# The burden of neurological disorders across the states of India: the Global Burden of Disease Study 1990–2019

**DOI:** 10.1016/S2214-109X(21)00164-9

**Published:** 2021-07-14

**Authors:** India State-Level Disease Burden Initiative Neurological Disorders Collaborators, Gagandeep Singh, Meenakshi Sharma, G Anil Kumar, N Girish Rao, Kameshwar Prasad, Prashant Mathur, Jeyaraj D Pandian, Jaimie D Steinmetz, Atanu Biswas, Pramod K Pal, Sanjay Prakash, P N Sylaja, Emma Nichols, Tarun Dua, Harkiran Kaur, Suvarna Alladi, Vivek Agarwal, Sumit Aggarwal, Atul Ambekar, Bhavani S Bagepally, Tapas K Banerjee, Rose G Bender, Sadhana Bhagwat, Stuti Bhargava, Rohit Bhatia, Joy K Chakma, Neerja Chowdhary, Subhojit Dey, M Ashworth Dirac, Valery L Feigin, Atreyi Ganguli, Mahaveer J Golechha, M Gourie-Devi, Vinay Goyal, Gaurav Gupta, Prakash C Gupta, Rajeev Gupta, Gopalkrishna Gururaj, Rajkumar Hemalatha, Panniyammakal Jeemon, Catherine O Johnson, Pradeep Joshi, Rajni Kant, Amal C Kataki, Dheeraj Khurana, Rinu P Krishnankutty, Hmwe H Kyu, Stephen S Lim, Rakesh Lodha, Rui Ma, Rajesh Malhotra, Ridhima Malhotra, Matthews Mathai, Ravi Mehrotra, Usha K Misra, Parul Mutreja, Mohsen Naghavi, Nitish Naik, Minh Nguyen, Anamika Pandey, Priya Parmar, Arokiasamy Perianayagam, Dorairaj Prabhakaran, Goura K Rath, Nickolas Reinig, Gregory A Roth, Rajesh Sagar, Mari J Sankar, K S Shaji, R S Sharma, Shweta Sharma, Ravinder Singh, M V Padma Srivastava, Benjamin A Stark, Nikhil Tandon, J S Thakur, Akhil S ThekkePurakkal, Sanjeev V Thomas, Manjari Tripathi, Avina Vongpradith, Han Y Wunrow, Denis Xavier, D K Shukla, K Srinath Reddy, Samiran Panda, Rakhi Dandona, Christopher J L Murray, Theo Vos, R S Dhaliwal, Lalit Dandona

## Abstract

**Background:**

A systematic understanding of the burden of neurological disorders at the subnational level is not readily available for India. We present a comprehensive analysis of the disease burden and trends of neurological disorders at the state level in India.

**Methods:**

Using all accessible data from multiple sources, we estimated the prevalence or incidence and disability-adjusted life-years (DALYs) for neurological disorders from 1990 to 2019 for all states of India as part of the Global Burden of Diseases, Injuries, and Risk Factors Study 2019. We assessed the contribution of each neurological disorder to deaths and DALYs in India in 2019, their trends in prevalence or incidence and DALY rates over time, and heterogeneity between the states of India. We also assessed the Pearson correlation coefficient between Socio-demographic Index (SDI) of the states and the prevalence or incidence and DALY rates of each neurological disorder. Additionally, we estimated the contribution of known risk factors to DALYs from neurological disorders. We calculated 95% uncertainty intervals (UIs) for the mean estimates.

**Findings:**

The contribution of non-communicable neurological disorders to total DALYs in India doubled from 4·0% (95% UI 3·2–5·0) in 1990 to 8·2% (6·6–10·2) in 2019, and the contribution of injury-related neurological disorders increased from 0·2% (0·2–0·3) to 0·6% (0·5–0·7). Conversely, the contribution of communicable neurological disorders decreased from 4·1% (3·5–4·8) to 1·1% (0·9–1·5) during the same period. In 2019, the largest contributors to the total neurological disorder DALYs in India were stroke (37·9% [29·9–46·1]), headache disorders (17·5% [3·6–32·5]), epilepsy (11·3% [9·0–14·3]), cerebral palsy (5·7% [4·2–7·7]), and encephalitis (5·3% [3·7–8·9]). The crude DALY rate of several neurological disorders had considerable heterogeneity between the states in 2019, with the highest variation for tetanus (93·2 times), meningitis (8·3 times), and stroke (5·5 times). SDI of the states had a moderate significant negative correlation with communicable neurological disorder DALY rate and a moderate significant positive correlation with injury-related neurological disorder DALY rate in 2019. For most of the non-communicable neurological disorders, there was an increase in prevalence or incidence from 1990 to 2019. Substantial decreases were evident in the incidence and DALY rates of communicable neurological disorders during the same period. Migraine and multiple sclerosis were more prevalent among females than males and traumatic brain injuries were more common among males than females in 2019. Communicable diseases contributed to the majority of total neurological disorder DALYs in children younger than 5 years, and non-communicable neurological disorders were the highest contributor in all other age groups. In 2019, the leading risk factors contributing to DALYs due to non-communicable neurological disorders in India included high systolic blood pressure, air pollution, dietary risks, high fasting plasma glucose, and high body-mass index. For communicable disorders, the identified risk factors with modest contributions to DALYs were low birthweight and short gestation and air pollution.

**Interpretation:**

The increasing contribution of non-communicable and injury-related neurological disorders to the overall disease burden in India, and the substantial state-level variation in the burden of many neurological disorders highlight the need for state-specific health system responses to address the gaps in neurology services related to awareness, early identification, treatment, and rehabilitation.

**Funding:**

Bill & Melinda Gates Foundation; and Indian Council of Medical Research, Department of Health Research, Ministry of Health and Family Welfare, Government of India.

## Introduction

With an ageing population globally, the burden of neurological disorders is rapidly increasing, posing a challenge to the sustainability of health systems, including in low-income and middle-income countries.^1^, ^2^, ^3^, ^4^, ^5^, ^6^, ^7^, ^8^, ^9^ The burden of neurological disorders is also expected to increase in India due to the rapid demographic and epidemiological transition occurring in the country.^10^ Evidence regarding the incidence, prevalence, and disease burden associated with neurological disorders in India is scarce. Only a few local studies have reported disease burden for some neurological disorders.^11^, ^12^, ^13^, ^14^, ^15^, ^16^, ^17^, ^18^ We have previously reported the state-level distributions of stroke and brain and nervous system cancers.^19^, ^20^ However, a recent and comprehensive understanding of the magnitude and trends of all neurological disorders is not available, which is required for evidence-based preventive strategies, health-care planning, priority setting, and resource allocation. In 2020, the 73rd World Health Assembly resolution on global actions on epilepsy and other neurological disorders called for a globally integrated strategy underpinned by preventive, diagnostic, therapeutic, and rehabilitative plans to address neurological conditions more effectively in low-income and middle-income countries.^21^

**Research in context**

**Evidence before this study**

We searched PubMed for published papers on neurological disorders in India, Google for reports in the public domain, and references in these papers and reports, using the search terms “Alzheimer's disease”, “central nervous system neoplasms”, “cerebral palsy”, “DALYs”, “dementia”, “encephalitis”, “epidemiology”, “epilepsy“, “headache disorders“, “incidence”, “India“, “meningitis”, “morbidity“, “motor neuron diseases”, “multiple sclerosis“, “nervous system disease“, “neurological disorders”, “Parkinson's disease”, “prevalence“, “spinal cord injuries”, “stroke”, “tetanus“, “traumatic brain injuries”, and “trends”, on Feb 2, 2021, without language or publication date restrictions. We found several studies describing the incidence, prevalence, and disease burden of various neurological disorders in different parts of India. However, no systematic compilation was available on trends in the incidence, prevalence, and disability-adjusted life-years (DALYs) for the major neurological disorders across the states of India over a long period, which is needed to inform neurological health policies and programmes in the country.

**Added value of this study**

To our knowledge, this study is the first to provide estimates on the prevalence or incidence and DALY rates of most neurological disorders for every state of India from 1990 to 2019, on the basis of all accessible data sources by use of the standardised Global Burden of Disease, Injuries, and Risk Factors Study methodology. The findings in this report highlight that the contribution of non-communicable and injury-related neurological disorders to total DALYs more than doubled between 1990 and 2019 in India. The increasing burden of non-communicable neurological disorders was mainly attributable to the increase in the proportion of older age groups in the population. This analysis found significant correlation of the Socio-demographic Index of states with communicable and injury-related neurological disorder DALYs. We also present data on the age patterns in the prevalence or incidence of each neurological disorder in India and the sex differentials, as well as the age distribution of DALYs from each neurological disorder. Additionally, this study reports data on the leading risk factors contributing to DALYs due to specific neurological disorders in India in 2019.

**Implications of all the available evidence**

This comprehensive assessment of neurological disorders in all states of India from 1990 to 2019 highlights a pattern of increasing burden of non-communicable and injury-related neurological disorders, which suggests the need to steer the health system to address these disorders more effectively. The state-specific trends in the burden of neurological disorders provided in this report could be useful as a reference in the future planning of approaches to contain the increasing burden of these disorders in all parts of the country.

The population of India comprises about 18% of the world population, with many of its states having populations equivalent to those of entire countries.^22^ Considerable differences exist between the states in terms of socioeconomic status, culture, ethnicity, genetics, and health systems. Therefore, analysing the state-level burden of neurological disorders is important, to enable policy makers to frame health-care policies and programmes that are suitable for individual states. In this report, we present a detailed account of the prevalence, incidence, deaths, and disability-adjusted life-years (DALYs) of neurological disorders for the states of India from 1990 to 2019, and the risk factors associated with these disorders. We present modelled estimates based on all accessible data sources.

## Methods

### Overview

The analysis and findings of neurological disorders presented in this report were produced by the India State-Level Disease Burden Initiative as part of the Global Burden of Diseases, Injuries, and Risk Factors Study (GBD) 2019. The work of this initiative is approved by the Health Ministry Screening Committee at the Indian Council of Medical Research, and by the ethics committee of the Public Health Foundation of India. A comprehensive description of the metrics, data sources, and statistical modelling for neurological disorders in GBD 2019 has been provided elsewhere.^23^, ^24^, ^25^ The current report includes non-communicable neurological disorders (stroke, headache disorders, epilepsy, cerebral palsy, Alzheimer's disease and other dementias, brain and CNS cancer, Parkinson's disease, multiple sclerosis, motor neuron diseases, and other neurological disorders with available International Classification of Diseases (ICD; 9th and 10th revision) codes [appendix pp 7–12]), communicable neurological disorders (encephalitis, meningitis, and tetanus), and injury-related neurological disorders (traumatic brain injuries and spinal cord injuries). The GBD 2019 methods relevant for this report are summarised here and described in detail in the appendix (pp 3–98).

### Estimation of prevalence, incidence, and years lived with disability (YLDs)

The definitions of neurological disorders and their subtypes in this study are based on various standard clinical diagnostic criteria, details of which are provided in the appendix (pp 4–7). The prevalence or incidence of neurological disorders was estimated with DisMod-MR version 2.1, a Bayesian disease modelling meta-regression computational tool that is the standard GBD modelling approach for describing non-fatal health outcomes by location, age, sex, and year. This approach involved identification of all available data sources that could be accessed, estimation of cause-specific prevalence or incidence, severity distribution for sequelae, quantification of the magnitude of health loss by use of disability weights, adjustment for comorbidity, and computation of YLDs for each location, age, sex, and year.^23^ YLDs were estimated as the product of the prevalence estimate and disability weights for health states of each mutually exclusive sequela with adjusted comorbidities. The major data inputs for estimating prevalence and incidence of neurological disorders in India included registries and population-based studies (appendix pp 99–119).

### Estimation of deaths, years of life lost (YLLs), and DALYs

The major data inputs for the estimates of death due to neurological disorders in India included Sample Registration System cause of death data and other verbal autopsy studies, and Medical Certification of Cause of Death data (appendix pp 99–119). All available and accessible data (including those for covariates) were used to develop a set of plausible models and, eventually, the best ensemble predictive model to produce estimates of deaths and YLLs due to premature mortality by location, age, sex, and year.^23^, ^24^, ^25^ YLLs were computed from observed deaths and reference standard life expectancy at the age of death, which was obtained from the GBD standard life table.^24^ For Alzheimer's disease and other dementias, and Parkinson's disease, customised modelling approaches were used as shown in the appendix (pp 46–47 and 51–53) because of the challenges in accurate assignment of these causes in the death data sources.^23^ Cerebral palsy prevalence was estimated indirectly by aggregating all sequelae of neonatal, congenital, and infectious causes with mention of moderate or severe motor impairment.^26^ DALYs, a summary measure of total health loss, were computed by adding YLLs and YLDs for each cause under neurological disorders.^23^ For cerebral palsy, traumatic brain injuries, and spinal cord injuries, DALYs were estimated only as YLDs.

### Estimation of risk factor exposure and attributable disease burden

The GBD comparative risk assessment framework was used to estimate exposure of risk factors related to neurological disorders and their attributable disease burden.^25^ The estimation of attributable disease burden included ascertainment of the relative risk of disease outcomes for risk exposure–disease outcome pairs with sufficient evidence of a causal relation in global literature (ie, randomised control trials, prospective cohorts, and case-control studies; appendix pp 99–119). Exposure data for risk factors with a categorical or continuous distribution were collated from all available data sources, including survey and other data, adjusted by use of age-sex splitting, and strengthened by the incorporation of covariates for modelling. The modelling approach integrated multiple data inputs and borrowed information across age, time, and location to produce the best possible estimates of risk exposure by location, age, sex, and year (appendix pp 59–98). For each risk factor, the theoretical minimum risk exposure level was established as the lowest level of risk exposure below which its relationship with a disease outcome was not supported by the available evidence. Estimates of mean risk factor exposure, strengthened by covariates, were used to calculate summary exposure values for each risk, a metric ranging from 0% to 100% to describe the risk-weighted exposure for a population or risk-weighted prevalence of exposure.^25^

Estimates of DALYs attributable to each risk factor were calculated by sex. A detailed description of estimation of exposure and attributable disease burden estimation for the major risk factors associated with neurological disorders, including the GBD exposure definitions and statistical modelling, is provided in the appendix (pp 59–98) and has been previously published.^23^, ^25^

GBD uses covariates that have a known association with the outcome of interest as explanatory variables to arrive at the best possible estimate of the outcome of interest when data for the outcome are scarce but data for covariates are available.^23^, ^24^, ^25^ This approach was part of the estimation process for the findings presented in this Article.

### Analysis presented in this paper

Findings are reported for 31 geographical units in India: 28 states, the Union Territory of Delhi, the Union Territory of Jammu & Kashmir combined with the Union Territory of Ladakh, and the other small union territories combined (Andaman and Nicobar Islands, Chandigarh, Dadra and Nagar Haveli, Daman and Diu, Lakshadweep, and Puducherry). The states of Chhattisgarh, Uttarakhand, and Jharkhand were created from existing larger states in 2000, and the state of Telangana was created from Andhra Pradesh in 2014. For estimation of trends from 1990 to 2019, data from districts that now constitute the four new states were disaggregated from their parent states and classed under the new states.

We report overall, age-specific, and sex-specific prevalence or incidence and DALY rates for the year 2019 for each neurological disorder across all states of India. We report deaths from each neurological disorder in India in 2019. Prevalence is reported for all non-communicable neurological disorders, except stroke, and incidence is reported for stroke, communicable neurological disorders, and injury-related neurological disorders, on the basis of the metric that is most commonly used clinically for each disease. The same metrics were also used in the previously published GBD global neurological disorders paper.^1^ Prevalence and incidence are not directly comparable. We assessed the Pearson correlation coefficient between the continuous distribution of Socio-demographic Index (SDI) of each state in 2019, and the state-level crude and age-standardised DALY rates of non-communicable, communicable, and injury-related neurological disorders. We also present age-specific DALYs for each neurological disorder in India in 2019. Additionally, we assessed the correlation between SDI of the states and the prevalence or incidence and DALY rates of each neurological disorder. SDI is a composite indicator of development status, which ranges from 0 to 1, and is a geometric mean of the values of the indices of lag-distributed per capita income, mean education in people aged 15 years or older, and total fertility rate in people younger than 25 years in the state.^24^

We assessed the percentage change between 1990 and 2019 for prevalence or incidence and DALY rates of the neurological disorders. In addition, we present the DALYs for specific neurological disorders that were attributable to the major risk factors in 2019.

We present both crude and age-standardised estimates as relevant. Crude estimates indicate the actual situation in each state and are thus useful for policy makers. Conversely, age-standardised estimates allow comparisons over time and across states adjusting for the differences in the population age distribution. GBD uses a global reference population age structure for age standardisation.^24^ Mean estimates are reported with 95% uncertainty intervals (UIs) wherever relevant. These intervals are based on 1000 runs of the models for each quantity of interest, with the 2·5th and 97·5th percentiles considered as the 95% UI (appendix p 98).^23^, ^24^, ^25^ 95% UIs and p values were used to interpret statistical significance.

### Role of the funding source

Some of the contributors to this study work with the Indian Council of Medical Research. The other funder of the study, the Bill & Melinda Gates Foundation, had no role in study design, data collection, data analysis, data interpretation, or writing of the report.

## Results

### Overview

The contribution of non-communicable neurological disorders to total DALYs from all causes in India doubled from 4·0% (95% UI 3·2–5·0) in 1990 to 8·2% (6·6–10·2) in 2019, whereas the contribution of communicable neurological disorders decreased from 4·1% (3·5–4·8) in 1990 to 1·1% (0·9–1·5) in 2019. Injury-related neurological disorders contributed to 0·6% (0·5–0·7) of total DALYs in 2019, compared with 0·2% (0·2–0·3) in 1990. The proportion of total DALYs due to all neurological disorders (combining communicable, non-communicable, and injury-related disorders) changed marginally in India, from 8·3% (7·3–9·5) in 1990 to 9·9% (8·2–11·9) in 2019.

**Figure 1:**
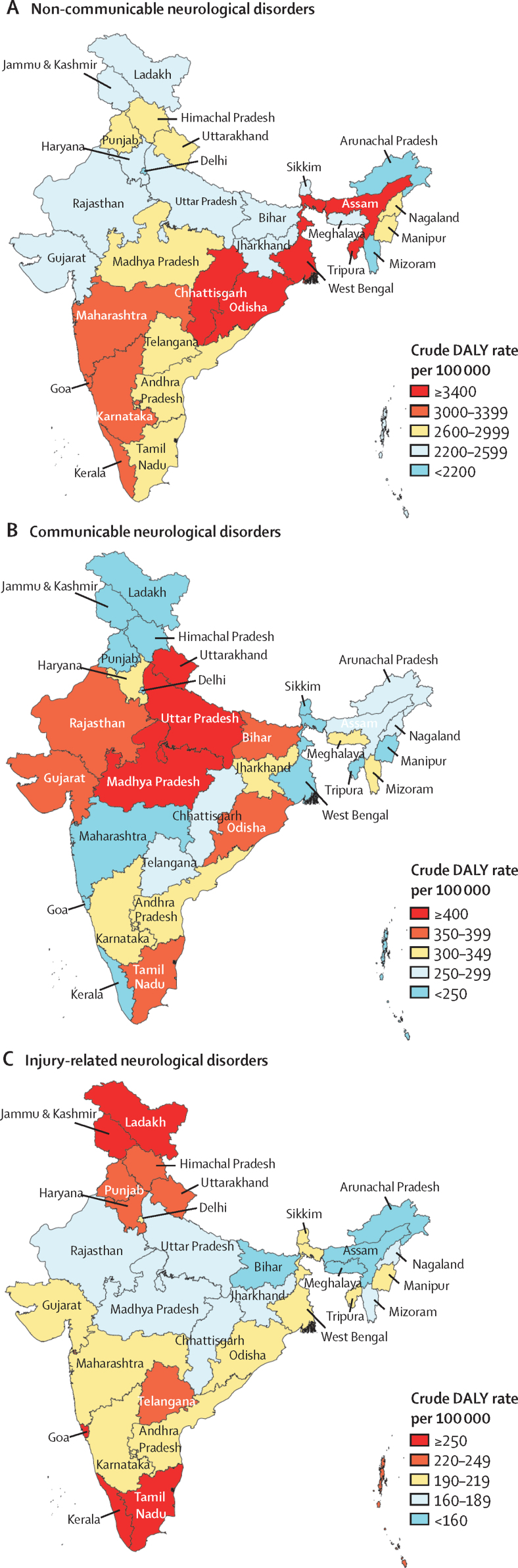
Crude DALY rates of non-communicable, communicable, and injury-related neurological disorders in the states of India, 2019 DALY=disability-adjusted life-year.

In 2019, of the total DALYs due to all neurological disorders in India, the contribution of non-communicable neurological disorders was 82·8% (78·5–86·2), that of communicable neurological disorders was 11·2% (8·4–15·0), and that of injury-related neurological disorders was 6·0% (4·6–7·7; table 1). Regarding correlation between SDI of the states and the crude and age-standardised DALY rates, there was a moderate significant negative correlation for communicable neurological disorders, a moderate significant positive correlation for injury-related neurological disorders, and no significant correlation for non-communicable neurological disorders (appendix p 121). The crude DALY rate of non-communicable neurological disorders had a 2·1 times variation between the states, with the highest rates in the eastern states of Chhattisgarh, West Bengal, and Odisha, and the northeast states of Tripura and Assam (figure 1, appendix p 122). The crude DALY rate of communicable neurological disorders had a 4·6 times variation between the states, with the highest rates in the northern states of Uttar Pradesh, Madhya Pradesh, and Uttarakhand (figure 1, appendix p 122). The crude DALY rate of injury-related neurological disorders had a 2·0 times variation between the states, with the highest rates in the southern states of Tamil Nadu and Kerala, followed by Goa in the west, and Jammu & Kashmir and Ladakh in the north (figure 1, appendix p 122). In 2019, the leading contributors to total DALYs from neurological disorders in India were stroke (37·9% [95% UI 29·9–46·1]), headache disorders (17·5% [3·6–32·5]), and epilepsy (11·3% [9·0–14·3]), followed by cerebral palsy (5·7% [4·2–7·7]), and encephalitis (5·3% [3·7–8·9]); table 1). In 2019, the predominant contributor to total deaths caused by neurological disorders in India was stroke (68·0% [95% UI 54·6–75·3]), followed by Alzheimer's and other dementias (12·0% [3·2–29·1]) and encephalitis (5·0% [3·7–8·1]; appendix p 123).

**Table 1:** Contribution of neurological disorders to total neurological disorder disability-adjusted life-years in India, 2019

		**Both sexes**	**Males**	**Females**
**Non-communicable disorders**		**82·8% (78·5–86·2)**	**81·9% (78·0–85·0)**	**83·6% (78·5–87·5)**
Stroke		37·9% (29·9–46·1)	39·5% (31·6–47·6)	36·2% (27·0–45·6)
Headache disorders		17·5% (3·6–32·5)	14·2% (2·9–27·1)	21·0% (4·5–38·0)
	Migraine	16·0% (2·5–31·1)	12·8% (2·0–25·6)	19·2% (3·0–36·4)
	Tension-type headache	1·6% (0·5–5·8)	1·5% (0·4–6·1)	1·7% (0·5–5·6)
Epilepsy		11·3% (9·0–14·3)	12·1% (9·7–15·1)	10·5% (8·0–13·7)
	Idiopathic epilepsy	6·4% (4·8–8·0)	6·8% (5·4–8·5)	5·9% (4·2–7·7)
	Secondary epilepsy	5·0% (3·6–6·7)	5·3% (3·8–7·1)	4·6% (3·3–6·4)
Cerebral palsy		5·7% (4·2–7·7)	5·9% (4·3–7·9)	5·5% (3·9–7·6)
Alzheimer's disease and other dementias		4·6% (1·9–10·4)	4·0% (1·6–9·4)	5·2% (2·1–11·7)
Brain and CNS cancer		2·2% (1·7–2·8)	2·5% (1·7–3·3)	1·9% (1·4–2·6)
Parkinson's disease		1·8% (1·4–2·2)	2·0% (1·6–2·4)	1·6% (1·2–2·0)
Multiple sclerosis		0·2% (0·2–0·3)	0·2% (0·2–0·3)	0·3% (0·2–0·4)
Motor neuron diseases		0·1% (0·1–0·2)	0·1% (0·1–0·2)	0·1% (0·1–0·2)
Other neurological disorders*		1·3% (0·9–1·7)	1·3% (0·9–1·8)	1·3% (0·9–1·7)
**Communicable disorders**		**11·2% (8·4–15·0)**	**10·7% (8·2–14·6)**	**11·8% (8·5–16·5)**
Encephalitis		5·3% (3·7–8·9)	5·0% (3·6–9·2)	5·6% (3·8–9·3)
Meningitis		4·8% (3·7–6·1)	4·5% (3·5–5·7)	5·1% (3·7–6·6)
Tetanus		1·1% (0·7–1·8)	1·2% (0·6–2·1)	1·1% (0·6–1·8)
**Injuries**		**6·0% (4·6–7·7)**	**7·4% (5·7–9·5)**	**4·6% (3·4–6·2)**
Traumatic brain injuries		4·1% (3·0–5·4)	5·1% (3·8–6·8)	3·0% (2·1–4·1)
Spinal cord injuries		1·9% (1·5–2·5)	2·2% (1·7–2·8)	1·6% (1·2–2·2)

Data in parentheses are 95% uncertainty intervals.

* Other non-communicable neurological disorders include a list of uncommon diseases, for which the International Classification of Diseases codes are shown in the appendix (pp 7–12).

## Stroke

**Figure 2:**
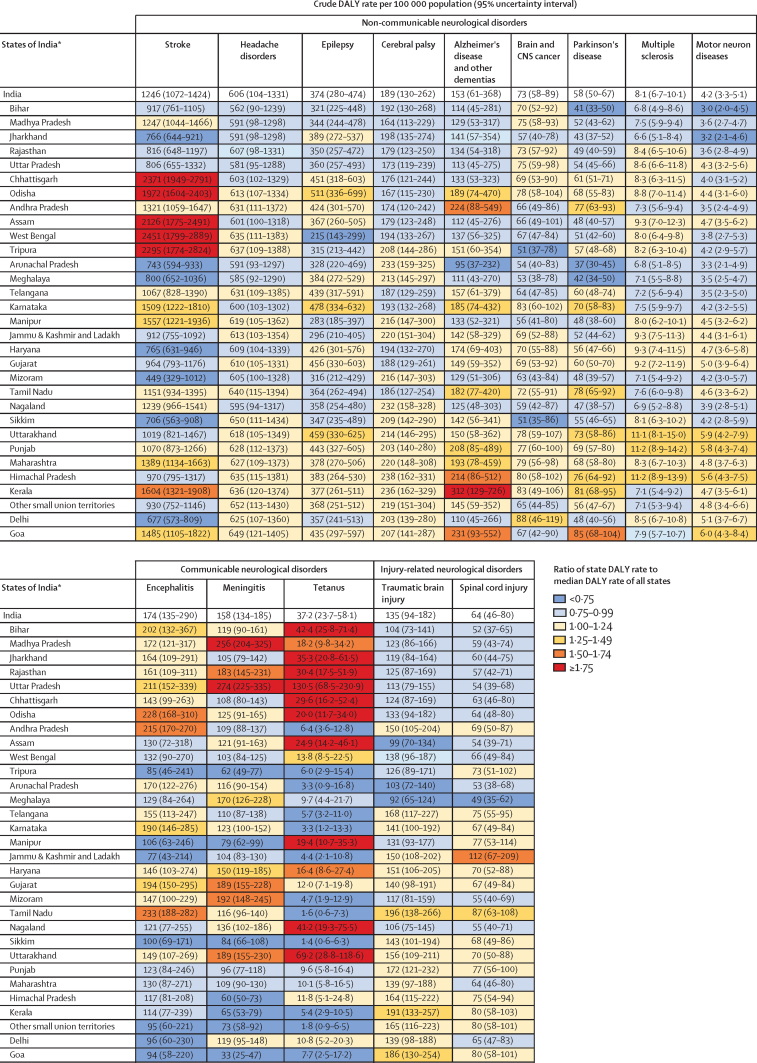
Crude DALY rates of neurological disorders in the states of India, 2019 DALY=disability-adjusted life-year. *States are listed in increasing order of Socio-demographic Index in 2019 (appendix p 120).

**Figure 3:**
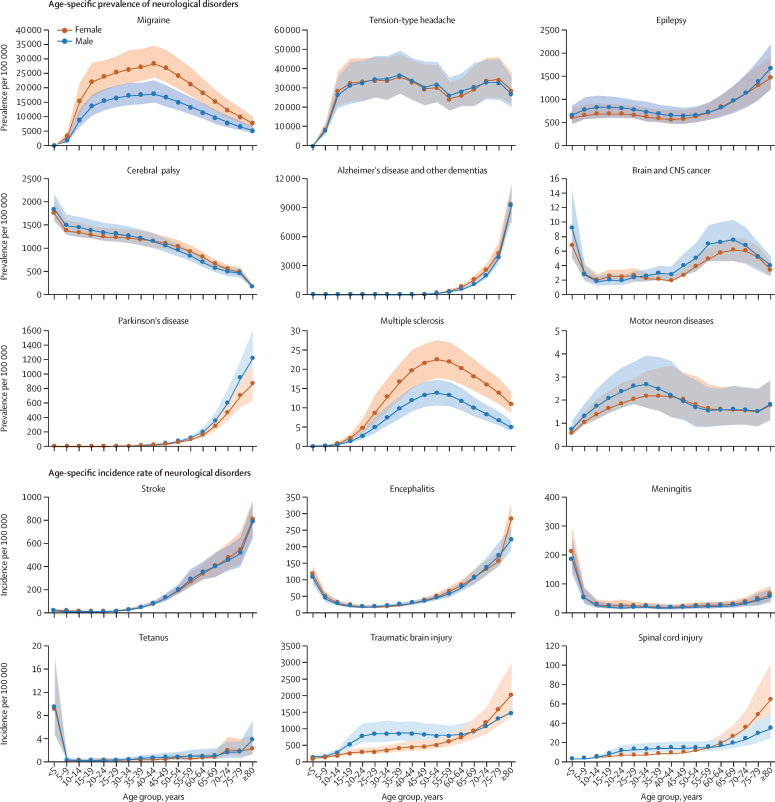
Age-specific prevalence or incidence rate of neurological disorders in India, 2019 Shaded areas show 95% uncertainty intervals. Prevalence or incidence rate are reported based on the metric that is most commonly used clinically for each disease.

In 2019, the estimated number of incident cases of stroke in India was 1·29 million (95% UI 1·15–1·45), and number of deaths due to stroke was 699 000 (95% UI 594 000–807 000; table 2, appendix p 123). The crude DALY rate of stroke had a 5·5 times variation between the states in 2019, with the highest rate in West Bengal, followed by Chhattisgarh and Tripura (figure 2). The crude and age-standardised incidence rates and crude DALY rate of stroke were not significantly correlated with SDI of the states, but age-standardised DALY rate had a weak significant negative correlation (appendix p 125). The crude incidence rate of stroke increased in India from 1990 to 2019, but the age-standardised incidence and DALY rates decreased. The increase in crude DALY rate was not significant over this period (table 3). Based on 2019 estimates, the incidence rate of stroke increased with age in both men and women (figure 3, appendix p 127).

**Table 2:** Number of prevalent or incident cases of neurological disorders in India, 2019

		**Both sexes**	**Males**	**Females**
**Non-communicable disorders; prevalent cases**				
Headache disorders		487 579 100 (448 555 100–527 120 900)	234 254 500 (214 459 000–254 437 600)	253 324 500 (233 111 600–273 361 700)
	Migraine	213 890 200 (185 723 700–246 241 400)	85 415 400 (73 737 300–99 504 200)	128 474 800 (111 438 800–147 423 900)
	Tension-type headache	374 453 700 (329 045 800–421 227 500)	191 610 700 (168 266 500–216 048 300)	182 843 000 (160 758 900–206 902 800)
Epilepsy		10 090 000 (8 395 500–11 858 300)	5 485 200 (4 558 900–6 417 500)	4 604 800 (3 825 600–5 398 600)
	Idiopathic epilepsy	4 008 700 (2 835 000–5 248 700)	2 212 600 (1 555 600–2 902 600)	1 796 100 (1 270 200–2 339 900)
	Secondary epilepsy	6 081 300 (5 277 900–6 888 300)	3 272 600 (2 827 100–3 725 600)	2 808 700 (2 427 500–3 196 800)
Cerebral palsy		16 821 600 (14 662 700–19 414 800)	8 814 800 (7 655 400–10 203 000)	8 006 800 (7 015 600–9 196 500)
Alzheimer's disease and other dementias		3 692 600 (3 132 600–4 249 300)	1 573 800 (1 334 100–1 818 300)	2 118 800 (1 805 100–2 437 700)
Brain and CNS cancer		49 300 (38 200–60 500)	26 900 (17 600–36 200)	22 400 (17 100–28 100)
Parkinson's disease		770 800 (635 100–919 400)	421 800 (348 300–502 100)	349 000 (287 200–416 500)
Multiple sclerosis		106 600 (83 800–130 300)	40 000 (31 100–49 700)	66 600 (52 700–81 700)
Motor neuron diseases		25 000 (19 900–31 500)	13 700 (10 900–17 300)	11 300 (8900–14 300)
Other neurological disorders*		9300 (6300–12 800)	5500 (3800–7800)	3800 (2500–5200)
**Non-communicable disorders; incident cases**				
Stroke		1 291 200 (1 150 700–1 453 000)	634 300 (564 600–716 700)	656 900 (586 700–738 700)
**Communicable disorders; incident cases**				
Encephalitis		610 200 (549 900–668 900)	303 000 (273 100–333 100)	307 200 (277 200–336 200)
Meningitis		552 100 (454 900–654 500)	266 000 (219 200–317 500)	286 000 (235 600–339 100)
Tetanus		16 600 (10 800–26 200)	9000 (5000–15 300)	7600 (4700–11 900)
**Injuries; incident cases**				
Traumatic brain injuries		7 464 800 (6 383 800–8 667 200)	4 673 100 (3 987 400–5 492 900)	2 791 700 (2 368 100–3 273 300)
Spinal cord injuries		134 900 (103 800–173 800)	74 000 (55 700–97 200)	60 900 (46 100–79 500)

Data in parentheses are 95% uncertainty intervals. Prevalent or incident cases are reported based on the metric that is most commonly used clinically for each disease.

* Other non-communicable neurological disorders include a list of uncommon diseases, for which the International Classification of Diseases codes are shown in the appendix (pp 7–12).

**Table 3:** Percentage change in prevalence or incidence and DALY rates of neurological disorders in India, 1990 to 2019

	**Crude estimate (prevalence or incidence rate)**	**Age-standardised estimate (prevalence or incidence rate)**	**Crude DALY rate**	**Age-standardised DALY rate**
**Non-communicable disorders; prevalence and DALY rate**				
Headache disorders	11·6% (9·5 to 13·5)	−0·1% (−1·0 to 0·9)	13·7% (9·0 to 23·4)	0·9% (−2·7 to 4·6)
Epilepsy	43·9% (20·5 to 73·0)	37·0% (16·8 to 61·9)	−10·2% (−27·2 to 17·3)	−7·2% (−23·0 to 18·9)
Cerebral palsy	49·2% (37·7 to 61·4)	53·6% (41·1 to 67·2)	99·3% (83·3 to 117·4)	119·6% (101·1 to 139·2)
Alzheimer's disease and other dementias	113·9% (105·7 to 123·6)	4·3% (2·9 to 5·9)	151·2% (122·0 to 183·6)	14·8% (3·5 to 27·4)
Brain and CNS cancer	8·3% (−39·5 to 58·9)	17·9% (−30·2 to 62·8)	−10·9% (−48·0 to 30·9)	−3·8% (−41·1 to 31·8)
Parkinson's disease	105·9% (97·2 to 115·0)	19·3% (17·4 to 21·2)	85·2% (51·0 to 120·1)	−3·5% (−21·1 to 14·6)
Multiple sclerosis	44·2% (40·7 to 47·8)	15·5% (13·9 to 17·2)	42·4% (11·5 to 91·9)	10·4% (−12·5 to 50·5)
Motor neuron diseases	17·3% (13·6 to 21·5)	11·1% (9·4 to 13·2)	67·3% (35·9 to 103·6)	46·3% (15·9 to 81·0)
Other neurological disorders*	10·9% (1·7 to 20·6)	2·1% (−3·5 to 7·3)	5·2% (−16·9 to 31·1)	14·6% (−8·1 to 40·8)
**Non-communicable disorders; incidence and DALY rate**				
Stroke	30·1% (26·5 to 33·7)	−8·8% (−10·0 to −7·5)	2·7% (−13·2 to 20·8)	−33·4% (−44·0 to −21·2)
**Communicable disorders; incidence and DALY rate**				
Encephalitis	−35·4% (−38·7 to −31·9)	−30·9% (−32·2 to −29·7)	−73·3% (−81·3 to −53·0)	−65·8% (−75·2 to −41·7)
Meningitis	−62·8% (−65·1 to −60·1)	−52·0% (−53·6 to −50·2)	−81·8% (−85·4 to −77·8)	−75·6% (−80·4 to −70·3)
Tetanus	−95·9% (−97·3 to −93·6)	−93·8% (−95·8 to −90·4)	−96·5% (−97·8 to −94·4)	−94·4% (−96·5 to −90·8)
**Injuries; incidence and DALY rate**				
Traumatic brain injuries	24·1% (17·8 to 30·0)	11·0% (7·3 to 14·3)	55·0% (52·4 to 57·7)	22·9% (21·2 to 24·6)
Spinal cord injuries	16·5% (2·5 to 28·7)	2·1% (−11·3 to 10·4)	28·2% (23·2 to 33·0)	7·9% (4·1 to 11·6)

Data in parentheses are 95% uncertainty intervals. DALY=disability-adjusted life-year.

* Other non-communicable neurological disorders include a list of uncommon diseases, for which the International Classification of Diseases codes are shown in the appendix (pp 7–12).

## Headache disorders

Headache disorders (comprising migraine and tension-type headache) were the most prevalent neurological disorder in India in 2019, affecting 488 million (95% UI 449–527) people (table 2). Between the two types of headache disorders, the DALY contribution of migraine to total neurological disorder DALYs was much higher than tension-type headache (16·0% [95% UI 2·5–31·1] *vs* 1·6% [0·5–5·8]). Variations in the crude DALY rate of headache disorders were minimal (1·2 times variation) between the states in 2019 (figure 2). The crude prevalence and DALY rate of headache disorders had a strong significant positive correlation with SDI of the states, but the age-standardised rates had no significant correlation (appendix p 125). The crude prevalence and DALY rate of headache disorders increased in India from 1990 to 2019, but no significant change occurred in the age-standardised prevalence or DALY rate (table 3). In 2019, prevalence of migraine was higher in females aged 35–59 years than in males of the same age. Prevalence increased with age and peaked at around age 40–44 years, followed by a gradual decrease in both females and males (figure 3, appendix p 126).

## Epilepsy

In 2019, the estimated number of people with epilepsy was 10·1 million (95% UI 8·40–11·9) in India, which included 4·01 million (2·84–5·25) people with idiopathic epilepsy and 6·08 million (5·28–6·89) people with secondary epilepsy (table 2). Idiopathic epilepsy contributed to 6·4% (95% UI 4·8–8·0) of the total neurological disorder DALYs and secondary epilepsy to 5·0% (3·6–6·7; table 1). An estimated 32 700 (95% UI 26 800–39 200) deaths due to epilepsy occurred in India in 2019 (appendix p 123). The crude DALY rate of epilepsy had a 2·4 times variation between the states in 2019. Odisha had the highest DALY rate due to epilepsy, followed by Karnataka and Uttarakhand (figure 2). The crude and age-standardised prevalence and age-standardised DALY rate of epilepsy had a moderate significant positive correlation with SDI of the states, but crude DALY rate had no significant correlation (appendix p 125). The crude and age-standardised prevalence of epilepsy increased in India from 1990 to 2019, but the DALY rates did not change significantly during this period (table 3). In 2019, the prevalence of epilepsy notably increased from age 55 years in both sexes (figure 3, appendix p 126).

## Cerebral palsy

An estimated 16·8 million (95% UI 14·7–19·4) people had cerebral palsy in India in 2019 (table 2). The crude DALY rate of cerebral palsy varied modestly between the states in 2019, with a 1·5 times variation (figure 2). Crude prevalence and DALY rate of cerebral palsy had a moderate significant positive correlation with SDI of the states and age-standardised rates had a strong significant positive correlation (appendix p 125). The crude and age-standardised prevalence and DALY rates increased substantially in India from 1990 to 2019 (table 3). In 2019, the prevalence of cerebral palsy was highest in children younger than 5 years in both sexes, and prevalence decreased with increasing age (figure 3, appendix p 126).

## Alzheimer's disease or other dementias

In India in 2019, an estimated 3·69 million (95% UI 3·13–4·25) people had Alzheimer's disease or other dementias, and 129 000 (95% UI 31 200–360 000) deaths occurred due to these diseases (table 2, appendix p 123). The crude DALY rate of Alzheimer's disease and other dementias had a 3·3 times variation between the states in 2019, with the highest rates in the states of Kerala, Goa, Andhra Pradesh, and Himachal Pradesh (figure 2). The crude prevalence and DALY rate of Alzheimer's disease and other dementias had a moderate significant positive correlation with SDI of the states, but the age-standardised rates had no significant correlation (appendix p 125). Both crude prevalence and DALY rate increased substantially in India from 1990 to 2019, but after age-standardisation the increases in prevalence and DALY rate were relatively smaller (table 3). In 2019, the prevalence of Alzheimer's disease and other dementias increased rapidly in the older age groups, particularly in those older than 60 years, both in males and females (figure 3, appendix p 126).

## Brain and CNS cancer

In 2019, the estimated number of people with brain and CNS cancer in India was 49 300 (95% UI 38 200–60 500), and number of deaths due to brain and CNS cancer was 23 700 (95% UI 18 600–28 900; table 2, appendix p 123). The crude DALY rate of brain and CNS cancer had a 1·7 times variation between states, with the highest rate in Delhi, followed by Karnataka, and Kerala (figure 2). The crude and age-standardised prevalence of brain and CNS cancer had a strong significant positive correlation with SDI of the states, but crude and age-standardised DALY rates had no significant correlation (appendix p 125). The crude and age-standardised prevalence and DALY rates had no significant change from 1990 to 2019 (table 3). In 2019, the prevalence of brain and CNS cancer had a sustained increase with age in females and males older than 44 years, with prevalence peaking at age 65–69 years in both sexes (figure 3, appendix p 126).

## Parkinson's disease

In India in 2019, an estimated 771 000 (95% UI 635 000–919 000) people had Parkinson's disease, and an estimated 45 300 (95% UI 38 600–52 800) deaths were due to Parkinson's disease (table 2, appendix p 123). The crude DALY rate of Parkinson's disease had a 2·3 times variation between the states in 2019, with the highest rate in Goa (figure 2). The crude and age-standardised prevalence and crude DALY rate of Parkinson's disease had a moderate significant positive correlation with SDI of the states, but age-standardised DALY rate had no significant correlation (appendix p 125). Both crude and age-standardised prevalence of Parkinson's disease increased in India from 1990 to 2019, with a greater increase in crude prevalence. The crude DALY rate of Parkinson's disease increased substantially during the same period, but the age-standardised rate did not change significantly (table 3). In 2019, Parkinson's disease was rare in young age groups. Prevalence increased notably in the older age groups, particularly in those older than 50 years, both in males and females (figure 3, appendix p 126).

## Multiple sclerosis

In 2019, the estimated number of people with multiple sclerosis in India was 106 600 (95% UI 83 800–130 300), and number of deaths due to multiple sclerosis was 2310 (95% UI 1860–2930; table 2, appendix p 123). The crude DALY rate of multiple sclerosis had a 1·7 times variation between the states, with the highest rates in Uttarakhand, Punjab, and Himachal Pradesh (figure 2). The crude and age-standardised prevalence and DALY rates were not significantly correlated with SDI of the states (appendix p 125). Notable increases occurred in the crude prevalence and DALY rate of multiple sclerosis from 1990 to 2019, with a smaller increase in age-standardised prevalence, and no significant change in age-standardised DALY rate during the same period (table 3). The prevalence of multiple sclerosis was higher in females than males, and increased after adolescence, peaking at age 50–54 years in females and males (figure 3, appendix p 126).

## Motor neuron diseases

In India in 2019, the estimated number of people with motor neuron diseases was 25 000 (95% UI 19 900–31 500) and the estimated number of deaths due to motor neuron diseases was 1600 (95% UI 1220–1990; table 2, appendix p 123). There was a 1·7 times variation between the states in the crude DALY rate of motor neuron diseases, with the highest rates in the states of Goa, Uttarakhand, Punjab, and Himachal Pradesh (figure 2). The crude and age-standardised prevalence and crude DALY rate of motor neuron diseases had a strong significant positive correlation with SDI of the states, and age-standardised DALY rate had a moderate positive correlation (appendix p 125). The crude and age-standardised prevalence and DALY rates of motor neuron diseases increased from 1990 to 2019, with the most notable increases in DALY rates (table 3). The prevalence of motor neuron diseases had a gradual increasing trend from childhood up to age 30–34 years, although the 95% UIs overlapped for most of the age groups. Prevalence was not significantly different between males and females (figure 3, appendix 126).

## Encephalitis

In 2019, the estimated number of incident cases of encephalitis in India was 610 000 (95% UI 550 000–669 000), and an estimated 51 900 deaths (95% UI 40 400–85 000) were due to encephalitis in the same year (table 2, appendix p 123). The crude DALY rate of encephalitis had a 3·0 times variation between the states in 2019, with the highest rate in Tamil Nadu, followed by Odisha and Andhra Pradesh (figure 2). The crude and age-standardised DALY rates of encephalitis had a moderate significant negative correlation with SDI of the states, but crude and age-standardised incidence rates had no significant correlation (appendix p 125). The crude and age-standardised incidence and DALY rates of encephalitis decreased substantially in India from 1990 to 2019, with the most notable decreases in DALY rates (table 3). The incidence rate of encephalitis was higher in children younger than 5 years and adults older than 60 years than in other age groups (figure 3, appendix p 127).

## Meningitis

An estimated 552 000 (95% UI 455 000–655 000) new cases of meningitis, and 34 700 (95% UI 29 700–40 000) deaths due to meningitis, occurred in India in 2019 (table 2, appendix p 123). The crude DALY rate of meningitis had a 8·3 times variation between the states, with the highest rates in Uttar Pradesh and Madhya Pradesh (figure 2). The crude incidence and DALY rates of meningitis had a moderate significant negative correlation with SDI of the states, age-standardised DALY rate had a weak negative correlation, and age-standardised incidence rate had no significant correlation (appendix p 125). The crude and age-standardised incidence and DALY rates of meningitis decreased substantially in India from 1990 to 2019 (table 3). In females and males, the incidence rate of meningitis was greatest in children younger than 5 years, decreasing with older age and remaining low at age 10–69 years. Incidence rate had a moderate increase from age 70 years and older in both sexes (figure 3, appendix p 127).

## Tetanus

In India in 2019, 16 600 (95% UI 10 800–26 200) new cases of tetanus and 7330 (95% UI 4920–11 000) deaths due to tetanus were estimated (table 2, appendix p 123). The crude DALY rate of tetanus had a 93·2 times variation between the states, with the highest DALY rates in Uttar Pradesh, Uttarakhand, and Bihar (figure 2). The crude DALY rate of tetanus had a weak significant negative correlation with SDI of the states, but crude and age-standardised incidence rates and age-standardised DALY rate were not significantly correlated with SDI of the states (appendix p 125). The crude and age-standardised incidence and DALY rates of tetanus decreased substantially (all by >90%) in India from 1990 to 2019 (table 3). The incidence of tetanus was highest in children younger than 5 years in both boys and girls, and incidence also increased in individuals older than 70 years in both sexes (figure 3, appendix p 127).

## Traumatic brain injuries

An estimated 7·46 million (95% UI 6·38–8·67) traumatic brain injuries occurred in India in 2019 (table 2). The crude DALY rate of traumatic brain injuries had a 2·1 times variation between the states in 2019, with the highest rates in Tamil Nadu, Kerala, and Goa (figure 2). The crude and age-standardised incidence rates and age-standardised DALY rate of traumatic brain injuries had a moderate significant positive correlation with SDI of the states, and crude DALY rate had a strong positive correlation (appendix p 125). The crude and age-standardised incidence and DALY rates increased in India from 1990 to 2019, with the most notable increase in crude DALY rate (table 3). The incidence rate of traumatic brain injuries increased with age in both sexes, and was significantly higher in males than in females aged 15–39 years (figure 3, appendix p 127).

## Spinal cord injuries

An estimated 135 000 (95% UI 104 000–174 000) new cases of spinal cord injuries occurred in India in 2019 (table 2). The crude DALY rate of spinal cord injuries had a 2·3 times variation between the states in 2019, with the highest rate in Jammu & Kashmir and Ladakh, followed by Tamil Nadu (figure 2). The crude incidence and DALY rates of spinal cord injuries had a moderate significant positive correlation with SDI of the states. Age-standardised incidence rate had a weak significant positive correlation and age-standardised DALY rate had no significant correlation with SDI of the states (appendix p 125). The crude incidence rate of spinal cord injuries increased in India from 1990 to 2019, but the age-standardised rate did not change significantly; both the crude and age-standardised DALY rates of spinal cord injuries increased in India from 1990 to 2019, although the increase was less for age-standardised rate (table 3). The incidence rate of spinal cord injuries had an increasing trend with age in females and males (figure 3, appendix p 127).

## Age-specific DALYs

**Figure 4:**
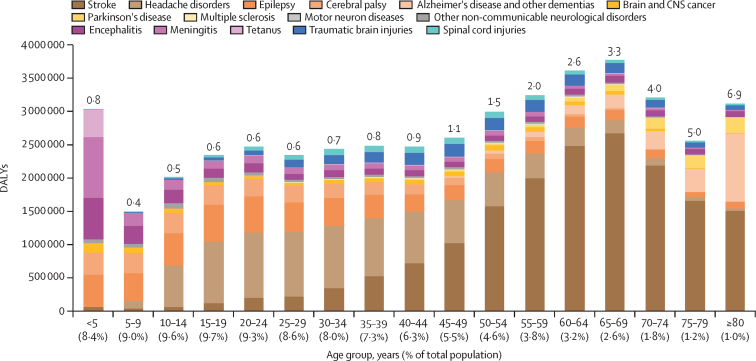
DALYs of neurological disorders by age group in India, 2019 The number on the top of each bar is the ratio of percentage of neurological disorder DALYs to percentage of the total population for that age group. DALY=disability-adjusted life-year.

The DALY to population ratio had an increase with age, reaching 6·9 in people aged 80 years and older (figure 4). However, there was less variation in the proportion of total neurological disorder DALYs across the age groups, because of a proportionately young population. In children younger than 5 years, communicable diseases contributed to the highest proportion of total neurological disorder DALYs (63·7%), whereas the contribution of non-communicable neurological disorders was highest in all other age groups, and increased with age, reaching 93·6% in the 80 years and older age group (appendix p 129). The proportional contribution of injury-related neurological disorders to neurological disorder DALYs was highest in the 30–49 years age group (10·3%).

## Risk factors

The risk factors to which the burden of specific neurological disorders could be attributed in GBD 2019 are shown in table 4. In 2019, among the risk factors that contributed to DALYs due to stroke in India, the factors with the highest contribution to DALYs were high systolic blood pressure (55·3% [95% UI 47·1 to 62·0]), air pollution (42·2% [39·3 to 44·9]), dietary risks (28·1% [19·9 to 37·7]), high fasting plasma glucose (26·9% [18·4 to 37·6]), high body-mass index (BMI; 22·3% [13·6 to 31·1]), and smoking and secondhand smoke (15·9% [14·1 to 17·5]; table 4). DALYs due to Alzheimer's disease and other dementias could be attributed to smoking (11·7% [7·1 to 16·0]), high fasting plasma glucose (10·9% [2·6 to 23·0]), and high BMI (7·5% [2·7 to 14·8]). The proportional contribution of smoking to DALYs due to Alzheimer's disease and other dementias was larger in men (21·3% [13·3 to 29·0]) than in women (4·4% [2·4 to 6·6]). A small proportion of DALYs due to total idiopathic epilepsy was associated with alcohol use (6·8% [4·5 to 9·4]), with this proportion much higher in males (11·7% [7·9 to 15·9]) than in females (0·9% [0·5 to 1·5]). The major identified risk factor for multiple sclerosis in terms of attributable DALYs was smoking (6·7% [4·7 to 8·9]), and this proportion was notably higher in males (13·3% [9·8 to 17·0]) than in females (2·0% [1·3 to 2·7]). Smoking had an inverse relation with Parkinson's disease, with an estimated 6·4% (3·4 to 9·6) reduction in DALYs attributable to smoking. A small proportion of DALYs due to meningitis could be attributed to low birthweight and short gestation (5·8% [4·5 to 7·5]). Low birthweight and short gestation also contributed to a small proportion of DALYs due to encephalitis (1·8% [1·4 to 2·2]). Air pollution was also a modest risk contributing to DALYs due to meningitis (1·5% [1·1 to 2·0]).

**Table 4:** Percentage of DALYs attributable to risk factors for neurological disorders in India, 2019

	**Both sexes**	**Males**	**Females**
**Stroke**			
High systolic blood pressure	55·3% (47·1 to 62·0)	54·8% (46·5 to 61·5)	56·0% (47·7 to 63·0)
Air pollution	42·2% (39·3 to 44·9)	42·5% (39·5 to 45·3)	41·8% (39·0 to 45·0)
Dietary risks*	28·1% (19·9 to 37·7)	29·4% (20·5 to 39·3)	26·7% (19·0 to 36·0)
High fasting plasma glucose	26·9% (18·4 to 37·6)	28·9% (19·5 to 40·1)	24·7% (16·5 to 35·0)
High body-mass index	22·3% (13·6 to 31·1)	21·0% (12·5 to 30·0)	23·7% (14·8 to 33·0)
Smoking and secondhand smoke	15·9% (14·1 to 17·5)	22·4% (20·4 to 24·4)	8·4% (6·9 to 10·0)
Kidney dysfunction	8·9% (7·5 to 10·5)	9·2% (7·7 to 10·9)	8·6% (7·3 to 10·0)
Lead exposure	8·7% (6·0 to 11·4)	9·1% (6·4 to 12·0)	8·3% (5·7 to 11·0)
High LDL cholesterol	6·1% (3·6 to 10·2)	5·9% (3·4 to 10·0)	6·4% (3·7 to 11·0)
Alcohol use	4·4% (2·7 to 6·3)	7·8% (4·8 to 10·9)	0·6% (0·1 to 1·2)
Non-optimal temperature	4·3% (1·7 to 6·6)	4·4% (1·7 to 6·7)	4·2% (1·7 to 7·0)
Low physical activity	1·0% (0·2 to 2·8)	0·8% (0·1 to 2·5)	1·4% (0·3 to 3·4)
**Idiopathic epilepsy**			
Alcohol use	6·8% (4·5 to 9·4)	11·7% (7·9 to 15·9)	0·9% (0·5 to 1·5)
**Alzheimer's disease and other dementias**			
Smoking	11·7% (7·1 to 16·0)	21·3% (13·3 to 29·0)	4·4% (2·4 to 6·6)
High fasting plasma glucose	10·9% (2·6 to 23·0)	11·3% (2·1 to 25·5)	10·6% (2·0 to 24·1)
High body-mass index	7·5% (2·7 to 14·8)	6·4% (1·5 to 14·6)	8·4% (1·8 to 17·8)
**Multiple sclerosis**			
Smoking	6·7% (4·7 to 8·9)	13·3% (9·8 to 17·0)	2·0% (1·3 to 2·7)
**Parkinson's disease**			
Smoking	−6·4% (−9·6 to −3·4)	−10·1% (−15·2 to −5·4)	−1·5% (−2·3 to −0·8)
**Meningitis**			
Low birthweight and short gestation	5·8% (4·5 to 7·5)	6·2% (4·3 to 8·5)	5·5% (3·9 to 7·8)
Air pollution	1·5% (1·1 to 2·0)	1·6% (1·1 to 2·2)	1·4% (1·0 to 2·1)
**Encephalitis**			
Low birthweight and short gestation	1·8% (1·4 to 2·2)	1·7% (1·2 to 2·3)	1·8% (1·3 to 2·5)

Data in parentheses are 95% uncertainty intervals. Risk factor exposures were as defined previously;^25^ estimations of risk exposure are detailed in the appendix (pp 59–98). The cumulative effect of the risk factors would be less than the sum of their individual contribution because the risk factors overlap. DALY=disability-adjusted life-year.

* Dietary risks include diets low in fruit, high in sodium, low in vegetables, low in fibre, low in whole grains, and high in red meat.

## Discussion

The findings in this report provide a systematic understanding of the burden of neurological disorders in the states of India from 1990 to 2019. The proportional contribution of non-communicable neurological disorders and injury-related neurological disorders to total DALYs more than doubled in India during this period, whereas the contribution of communicable neurological disorders in 2019 reduced to a quarter of that in 1990. With increasing SDI of the states, the DALY rate of communicable neurological disorders decreased and the DALY rate of injury-related neurological disorders increased in 2019. Among all neurological disorders in India in 2019, stroke, headache disorders, and epilepsy contributed to the greatest disease burden in terms of DALYs.

In 2019, the degree of heterogeneity varied among the states of India with regard to the burden of individual neurological disorders. Regarding non-communicable diseases, SDI of the states had a strong or moderate positive correlation with the crude DALY rate of headache disorders, Alzheimer's disease and other dementias, Parkinson's disease and motor neuron diseases, which was either absent or reduced in magnitude for age-standardised DALY rate, indicating that the increased burden of these disorders in developed states was related to ageing of the population. Both the crude and age-standardised DALY rates of cerebral palsy, which comprised only of morbidity (YLDs), were positively correlated with SDI of the states. As cerebral palsy is associated to a large degree with birth-related problems, improved birth care in high SDI states might lead to improved survival of babies with cerebral palsy, leading to a higher associated morbidity than in low SDI states. Conversely, improved birth care in high SDI states might result in less birth asphyxia, trauma, and infection in the newborn babies, leading to fewer cases of cerebral palsy than in low SDI states. However, with the available data we are not able to examine this trend further, but we anticipate that the estimation of cerebral palsy rates will improve in future GBD cycles. By contrast, both the crude and age-standardised DALY rates of encephalitis and meningitis were negatively correlated with SDI of the states, which is likely to be related to improved health care in developed states. The increase in age-standardised prevalence for most non-communicable neurological disorders, and in incidence of stroke, from 1990 to 2019 was less than the increase in crude prevalence or incidence, indicating the influence of ageing on this increase (ie, an increase in the proportion of older age groups in the population that have higher prevalence or incidence of these diseases^22^). Conversely, the age-standardised DALY rates of non-communicable neurological disorders did not increase significantly during this period, except for Alzheimer's disease and other dementias and motor neuron diseases, indicating the influence of improving health care over time.

The higher prevalence of migraine and multiple sclerosis among females than males in India is consistent with the reported global trends.^1^ However, our estimates did not show a significant difference between males and females in the prevalence of Alzheimer's disease and other dementias or Parkinson's disease in India, which is different from the reported global trends that show a higher prevalence in males.^1^, ^4^, ^5^ This discrepancy could be related to scarce population-level data on these disorders in India, and further data are needed to examine this. The incidence rate of traumatic brain and spinal cord injuries was higher among males than females in young adults, but higher among females than males in older adults, which is consistent with the higher incidence rate of road traffic injuries in young males and the higher incidence rate of falls in older females globally.

Except for stroke, only small proportions of neurological disorder DALYs were attributable to known risk factors, implying that more research is needed to understand association with risk factors. The leading risk factors for stroke were high systolic blood pressure and air pollution, followed by dietary risks, high fasting plasma glucose, high BMI, and smoking and secondhand smoke. Smoking was a risk factor for Alzheimer's disease and other dementias and multiple sclerosis, but showed a protective effect for Parkinson's disease, which is consistent with global trends. These effects of smoking were more prominent in males than females, which is consistent with higher smoking rates among males in India. Alcohol use was a risk factor for stroke and idiopathic epilepsy, which was also more prominent in males due to higher alcohol use among males in India.

The Indian Government launched the National Programme for Prevention and Control of Cancer, Diabetes, Cardiovascular Diseases and Stroke in 2010. Part of this programme is aimed at prevention, control, and treatment aspects of two non-communicable neurological disorders: stroke and brain and CNS cancer.^27^ The National Programme for Health Care of the Elderly in India, launched in 2010, addresses various health-related problems among older people.^28^ However, similar policy responses are lacking for the other neurological disorders in India. Other non-communicable neurological disorders could be integrated within the pre-existing vertical programmes. Despite several calls for a national programme for epilepsy in the past, no policies are in place to address the urgency of increasing epilepsy prevalence in the country. The publicly funded health system does provide treatment and care options for epilepsy under the Rashtriya Bal Swasthya Karyakram and Ayushman Bharat—Pradhan Mantri Jan Arogya Yojana government initiatives,^29^, ^30^ but more efforts are required to make epilepsy services widely available. Headache disorders, especially migraine, contributed substantially to the total DALYs in India in 2019, yet they are not recognised as a public health problem and are neglected in the process of defining standards of care. The burden of neurodegenerative disorders such as Alzheimer's disease and other dementias and Parkinson's disease has been increasing and is expected to rise further with increases in the proportion of older age groups in India.^22^ However, Alzheimer's disease and other dementias and Parkinson's disease are among the disorders without adequate attention in Indian health policies and programmes. The National Programme for Health Care of the Elderly was launched with an aim of providing comprehensive health services to older people at all levels of health care.^28^ However, this programme is still in a nascent stage of implementation in several states.^31^ The increasing burden of multiple sclerosis and motor neuron diseases has also not received attention from policy makers in India.

In the past 15 years, several immunisation campaigns related to communicable neurological disorders have been initiated in India. An important achievement has been the successful increase in coverage of tetanus vaccination under the Janani Surakhya Yojana and Janani Shishu Suraksha Karyakaram government initiatives (launched in 2005 and 2011, respectively), contributing towards a marked decrease in maternal and neonatal tetanus in India.^32^ Mission Indradhanush was launched by the Indian Government in 2014 to further improve immunisation coverage among children and pregnant women.^33^ This programme covers vaccination against *haemophilus influenzae* type b, which causes meningitis, and vaccination against Japanese encephalitis. In addition, pneumococcal conjugate vaccine is being introduced to the programme in a phased manner to provide coverage against *Streptococcus pneumoniae,* which also causes meningitis.^33^ Such increases in the availability of these vaccines is likely to reduce the burden of communicable neurological disorders.

The National Programme for Prevention and Management of Trauma and Burn Injuries in India, launched in 2014, aims to strengthen trauma care facilities and increase awareness about trauma care among the general population. Additionally, prevention of falls in the older population is one component of the National Programme for Health Care of the Elderly in India.^28^ However both of these programmes are in their initial stages of implementation,^34^, ^35^ and more work will be needed to reduce the burden of road traffic injuries and fall-related brain and spinal cord injuries in India.

Several supply-related barriers limit the organisation of neurology care services in India, including shortages in the neurology workforce, inadequate funding, shortages and irregular supply of drugs, scarcity of basic amenities, and infrastructural problems such as shortages of beds, furniture, and equipment particularly in public health facilities.^36^, ^37^, ^38^, ^39^ The acute shortages in the neurology workforce is mainly due to the low number of trained neurologists in India, suggesting the need to increase the amount of training institutions.^37^ A number of demand-related factors also hinder care services, including low perceived need for care among the public, distance to health facilities, and high costs of treatment, care, and rehabilitation, which also cause underuse of existing neurology services.^37^, ^40^, ^41^ Consequently, the burden associated with neurological disorders is higher than it could be if neurological services were more accessible and demand-related factors were favourable.

Strengthening the coverage of neurology services in the health-care system, utilising standard treatment protocols, should be prioritised. However, the importance of innovative, multidisciplinary, community-based interventions should also be considered for their role in spreading education and awareness and improving the early identification, detection, and rehabilitation of neurological disorders. Health education campaigns aimed at primary promotion and prevention in workplaces, schools, and communities can improve knowledge and awareness about neurological disorders.^42^ Schools can also be places for early identification and detection of neurological disorders with onset during childhood and adolescence.^43^ Non-specialist or lay community health workers, school teachers, community leaders, and private and non-governmental actors can all be engaged in strengthening coverage of care, by providing awareness and facilitating the early identification and detection of people with neurological disorders.^42^, ^44^, ^45^, ^46^, ^47^, ^48^, ^49^ For example, a four-staged treatment delivery model for epilepsy in tribal areas of the Indian State of Jharkhand has shown that voluntary health workers from the community can be effectively trained to identify people with epilepsy and persuade them to seek treatment.^50^ Introducing home-based care interventions also has the potential to improve treatment adherence, reduce costs of care, and improve overall quality of life.^50^ With regard to caregiver burden, a study in Goa noted that provision of a skilled team to support caregivers led to substantial improvements in the mental health of caregivers and reduced their burden of caregiving.^51^

The general limitations of the GBD methods, including those for estimation of neurological disorders, have been discussed previously.^23^, ^24^, ^25^ A major limitation of this study is that population-level data on the prevalence and incidence of many neurological disorders are scarce across the states of India. This deficiency in the data might have led to unknown biases in the estimates and trends reported in this paper. Due to this limitation, estimation of the subtypes of stroke was challenging for the states of India, and therefore these estimates were not presented. The estimates of cerebral palsy were indirect as these were based on sequelae data, which could be improved by use of cause-specific estimates for cerebral palsy in future GBD cycles.^26^ Additionally, we did not estimate the underlying causes of secondary epilepsy. Another important limitation is that studies are scarce in India on the association of risk factors with neurological disorders. Therefore, only well established risk factors from global data were included in this analysis that met strict criteria of a risk–cause association. To address the limitations related to data scarcity for neurological disorders in India, the GBD approach used covariates and other techniques that borrow strength over space and time to arrive at the best possible estimates. Although the best possible estimates are presented on the basis of modelling of the available data, the paucity of population-level data on neurological disorders has to be addressed in India to improve the robustness of the estimates. Furthermore, secondary headache disorders and some other conditions such as rabies, neurocysticercosis, neural tube defects, and neonatal encephalopathy were not included in our estimates of neurological disorder burden, or in GBD 2019, and such omissions have previously been suggested to lead to an underestimation of the total burden of neurological disorders.^52^ The strengths of this study include the utilisation of all available data sources in India that could be accessed to estimate the trends and patterns of neurological disorders in all states since 1990; comparability across locations and time owing to the use of standardised GBD methodology; and the comprehensive inputs from a network of neurology experts across India.

In conclusion, the burden of non-communicable and injury-related neurological disorders is increasing in India. Further research is needed to fill the knowledge gaps regarding the distribution, outcomes, and determinants of neurological disorders across the country. Given the poor availability of proper neurology health services, lack of knowledge and awareness, and stigma attached to these disorders in the country, efforts are needed by the government and other stakeholders to improve neurology services, with regard to treatment, care and rehabilitation, and preventive approaches where possible. In view of the considerable inter-state heterogeneity in disease burden for many neurological disorders, future policies and programmes should take into account the trends and context of each individual state.

## Data sharing

The neurological disorders burden data used in these analyses are available online and from the authors on request.

## Declaration of interests

MS, PMa, SAg, BSB, StB, JKC, RH, RK, RMe, RSS, SS, RSi, DKS, SPa, RSD, and LD work with the Indian Council of Medical Research, which partly funded this research. All other authors declare no competing interests.
