# Supplementary material for: The burden of neurological disorders across the states of India: the Global Burden of Disease Study 1990–2019
Source: Lancet Glob Health. 2021 Jul 14;9(8):e1129–44. doi: 10.1016/S2214-109X(21)00164-9 (PMC8295043; doi:10.1016/S2214-109X(21)00164-9)
Supplement: Supplementary appendix [file mmc1.pdf]

THE LANCET
Global Health

Supplementary appendix

This appendix formed part of the original submission and has been peer reviewed. We post it as supplied by the authors.

Supplement to: India State-Level Disease Burden Initiative Neurological Disorders Collaborators. The burden of neurological disorders across the states of India: the Global Burden of Disease Study 1990–2019. *Lancet Glob Health* 2021; published online July 14. http://dx.doi.org/10.1016/S2214-109X(21)00164-9.

The burden of neurological disorders across the states of India: the Global Burden of Disease study 1990-2019

India State-Level Disease Burden Initiative Neurological Disorders Collaborators

Web Appendix

Correspondence to: Prof. Lalit Dandona lalit.dandona@icmr.gov.in; lalit.dandona@phfi.org

Table of contents

1. GBD 2019 neurological disorders burden estimation methods....3
2. GBD 2019 India data inputs for neurological disorders morbidity, deaths, risk factors, and covariates ...99
3. Socio-demographic Index of the states of India, 2019....119
4. Correlation between SDI of the states of India and the DALY rates of non-communicable, communicable, and injury-related neurological disorders, 2019 120
5. Crude DALY rates of non-communicable, communicable, and injury-related neurological disorders in the states of India, 2019 121
6. Number of deaths due to neurological disorders in India, 2019 122
7. Prevalence or incidence rate of neurological disorders in the states of India, 2019....123
8. Correlation between SDI of the states of India and the prevalence or incidence and DALY rates of neurological disorders, 2019....124
9. Age-specific prevalence of select neurological disorders by sex in India, 2019....125
10. Age-specific incidence rate of select neurological disorders by sex in India, 2019....126
11. DALYs of neurological disorders by age group in India, 2019 127
12. Percentage contribution of communicable, non-communicable, and injury-related disorders to total neurological DALYs in each age group in India, 2019....128

1. GBD 2019 neurological disorders burden estimation methods

The material presented here is adapted from the following sources:

GBD 2019 Diseases and Injuries Collaborators. Global burden of 369 diseases and injuries, 1990–2019: a systematic analysis for the Global Burden of Disease Study 2019. *Lancet* 2020; 396: 1135-39.

GBD 2019 Risk Factors Collaborators. Global burden of 87 risk factors in 204 countries and territories, 1990–2019: a systematic analysis for the Global Burden of Disease Study 2019. *Lancet* 2020; 396: 1223–49.

Cieza A, Causey K, Kamenov K, Hanson SW, Chatterji S, Vos T. Global estimates of the need for rehabilitation based on the Global Burden of Disease study 2019: a systematic analysis for the Global Burden of Disease Study 2019. *Lancet* 2020; 396: 2006–17.

A. GBD case definitions of neurological disorders

The GBD case definitions and diagnostic criteria for neurological disorders are presented below:

Stroke

Stroke is defined according to WHO criteria – rapidly developing clinical signs of focal (at times generalized) disturbance of cerebral function lasting more than 24 hours or leading to death with no apparent cause other than that of vascular origin. Data on transient ischaemic attack (TIA) are not included.

1. Acute stroke: Stroke cases are considered acute from the data of incidence of a first ever stroke through day 28 following the event.
2. Chronic stroke: Stroke cases are considered chronic beginning 28 days following the occurrence of an event. Chronic stroke includes the sequelae of an acute stroke and all recurrent stroke events.
3. Ischaemic stroke: an episode of neurological dysfunction caused by focal cerebral, spinal, or retinal infarction.
4. Intracerebral haemorrhage: a focal collection of blood within the brain parenchyma or ventricular system that is not caused by trauma.
5. Subarachnoid haemorrhage: bleeding into the subarachnoid space (the space between the arachnoid membrane and the pia mater of the brain or spinal cord)

Migraine

Migraine is a disabling primary headache disorder, typically characterised by recurrent moderate or severe unilateral pulsatile headaches. The two major types are migraine without aura and migraine with aura (transient neurological symptoms). GBD does not distinguish between migraine with and without aura as most epidemiological studies report on overall migraine only. The reference diagnostic criteria for migraine are from the International Classification of Headache Disorders (ICHD), which describe five criteria:

1. At least five attacks fulfilling criteria 2-5
2. Headache attacks lasting 4-72 hour (untreated or unsuccessfully treated) as criteria
3. Headache has at least two of the following four characteristics:
   - Unilateral location
   - Pulsating quality
   - Moderate or severe pain intensity
   - Aggravation by or causing avoidance of routine physical activity
4. During headache at least one of the following:
   - Nausea and/or vomiting
   - Photophobia and phono phobia
5. Not better accounted for by another ICHD-3 diagnosis

Definite migraine is headache that satisfies all the criteria outlined above, while probable migraine satisfies all criteria except one. Studies that have looked at the reasons for cases with probable headache not fulfilling criteria definite diagnosis have suggested that most often it is the duration criterion that were left unfilled.

Tension-type headache

Tension-type headache (TTH) is characterised by a dull, non-pulsatile, diffuse, band-like (or vice-like) pain of mild to moderate intensity in the head or neck. The reference diagnostic criteria for tension-type headache are from the ICHD-3, which describes five criteria:

1. At least 10 attacks fulfilling criteria 2-5
2. Lasing from 30 minutes to 7 days
3. At least two of the following four characteristics:
   - Bilateral location
   - Pressing or tightening (non-pulsating) quality
   - Mild or moderate intensity
   - Not aggravated by routine physical activity such as walking or climbing stairs
4. Both of the following:
   - No nausea or vomiting

- o No more than one of photophobia or phono phobia

5. Not better accounted for by another ICHD-3 diagnosis

Definite tension-type headache is headache that satisfies all criteria outlined above, while probable tension-type headache satisfies all criteria except one.

Epilepsy

Epilepsy is a condition characterised by recurrent (two or more) epileptic seizures, unprovoked by any immediate identified cause. A prevalent case of active epilepsy is defined as a person with epilepsy who has had at least one epileptic seizure in the previous five years, regardless of antiepileptic drug (AED) treatment. GBD defines severe epilepsy as having seizures one or more times per month, idiopathic epilepsy as epilepsy of genetic origin or without a definite structural, metabolic, infective, or immune cause, and secondary epilepsy as epilepsy due to structural, metabolic, infective, or immune cause.

Cerebral palsy

Cerebral palsy estimation is aggregation of all sequelae of neonatal, congenital and infectious disease with mention of moderate or severe motor impairment.

Alzheimer's disease & other dementias

Dementia is a progressive, degenerative, and chronic neurological disorder typified by memory impairment and other neurological dysfunctions. GBD used the Diagnostic and Statistical Manual of Mental Disorders (DSMIV) III, IV or V, or ICD case definitions as the reference. DSMIV definition is:

- Multiple cognitive deficits manifested by both memory impairment and one of the following: aphasia, apraxia, agnosia, disturbance in executive functioning
- Must cause significant impairment in occupational functioning and represent a significant decline
- Course is characterized by gradual onset and continuing cognitive decline
- Cognitive deficits are not due to other psychiatric conditions
- Deficits do not occur exclusively during the course of a delirium

A wide array of diagnostic and screening instruments exists, including Clinical Dementia Rating scale (CDR), Mini Mental State Examination (MMSE), and the Geriatric Mental State (GMS). For severity rating purposes, GBD used the CDR as the reference.

Brain and nervous system cancer

This cause includes malignant neoplasms of the brain and central nervous system, captured by ICD-10 codes C70–C70.1, C70.5, C70.6, and C70.9–C72.9. Some registries included mortality from benign neoplasms, as they can cause death through intracranial pressure.

Parkinson's disease

Parkinson's disease is a chronic, degenerative and progressive neurological condition typified by the loss of motor mobility and control most notably tremors. The case definition for GBD is the presence of at least two of the four primary symptoms:

- Tremors/trembling
- Bradykinesia
- Stiffness of limbs and torso
- Posture instability

Multiple sclerosis

Multiple sclerosis is a chronic, degenerative, and progressive neurological condition typified by the damaging of the myelin sheaths. Mc Donald's criteria for diagnosis were considered the contemporary gold standard.

In GBD, diagnosis by McDonald's criteria, other published criteria (such as Poser, Schumacher, or McAllen criteria), and clinical neurological exam are all treated as reference.

Motor neuron diseases

Motor neuron diseases (MND) are a set of chronic, degenerative, and progressive neurological conditions typified by the destruction of motor neurons and the subsequent deterioration of voluntary muscle activity.

The most common MND is amyotrophic lateral sclerosis (ALS). The El Escorial Criteria are the gold standard diagnostic criteria.

Other non-communicable neurological disorders

Other non-communicable neurological disorders are defined as all non-communicable neurological disorders that are not Alzheimer's and & dementias, Parkinson's disease, epilepsy, cerebral palsy, multiple sclerosis, motor neuron disease, or headaches. The majority of the unspecified non-communicable neurological disorders are muscular dystrophy and Huntington's disease, but there are a number of other, rare neurological disorders included as well.

Encephalitis

Encephalitis is a disease caused by an acute inflammation of the brain. Symptoms of encephalitis can include flu-like symptoms like headache, fever, drowsiness, and fatigue, and at times, seizures, hallucinations, or stroke.

Meningitis

Meningitis is a disease caused by inflammation of the meninges, the protective membrane surrounding the brain and spinal cord, and is typically caused by an infection in the cerebrospinal fluid. Symptoms include headache, fever, stiff neck, and sometimes seizures.

Tetanus

Tetanus is a serious bacterial disease caused by the bacterium Clostridium tetani.

Traumatic brain injury

Traumatic brain injury is defined by an external force applied to the head causing injury to the brain manifest by different levels of clinical severity. Traumatic brain injury can be differentiated into mild, moderate, and severe based on Glasgow Coma Scale and neurological imaging.

Spinal cord injury

Spinal cord injury is defined as an external injury to the vertebral column which leads to mechanical compression or distortion of the spinal cord leading to diminished or lost function below the spinal cord level of the injury. GBD separated spinal cord injury into lesions at the neck level and below the neck level given the different levels of disability that can be caused by lesions at different levels.

B. GBD neurological disorders list

B1. List of ICD codes mapped to the GBD neurological disorders list

The codes used by GBD 2019 from the 9th and 10th revisions of the International Statistical Classification of Diseases and Related Health Problems (ICD) for neurological disorders are listed below:

Cause	ICD 10	ICD 9
Stroke	G45-G46.8, I60-I62, I62.9-I64, I64.1, I65-I69.998, Z82.3	430-439.6, V12.54, V17.1
Migraine	G43-G43.919	346-346.93
Tension-type headache	G44.2-G44.41	307.81, 339.1-339.3
Epilepsy	G40-G41.9, Z82.0	345-345.91
Alzheimer's disease & other dementias	F00-F03.91, F06.2, G30-G31.1, G31.8-G32.89	290-290.9, 294.0-294.9, 331-331.2, 331.6-331.7, 331.82, 331.89-331.9
Brain and nervous system cancer	C70-C70.1, C70.9-C72.9, Z85.841-Z85.848, Z86.011	191-191.9
Parkinson's disease	G20-G20.9	332-332.0
Multiple sclerosis	G35-G35.0	340-340.9
Motor neuron diseases	G12-G13	335-335.9
Encephalitis	A83-A85.2, A85.8-A86.0, B94.1, F07.1, G04 - G05.8, Z24.1	062-064.9, 310.89, 323-323.9, V05.0-V05.1
Meningitis	A39-A39.9, A87-A87.9, D86.81, G00-G03.9, G06-G09.9, Z20.811, Z22.31	036-036.9, 047-049.9, 054.72, 320-320.3, 320.5-322.9, 324-326.9, V01.84
Tetanus	A33-A35.0, Z23.5	037-037.9, 771.3, V03.7
Traumatic brain injury	F07.2, F07.8, F07.81, F07.89, F07.9, S06, S07, T90.2, T90.4	850, 852, 907
Spinal cord injury	S14, T91.3, S24, S34, T06.0, T06.1, T09.3, T09.4, P11.5	806.0, 806.1, 952.0, 806.2-806.9, 952.1, 952.2, 952.3, 952.4, 952.8, 952.9
Other non-communicable neurological disorders	G10, G21.2-G24, G24.1-G25.0, G25.2-G25.3, G25.5, G25.8-G26.0, G36-G37.9, G50-G54.1, G54.5-G62, G62.1-G65.2, G70-G72, G72.1-G73.7, G 81-G83.9, G89-G93.6, G93.8-G95.29, G95.8-G96, G96.1, G96.12-G96.9, G98-G99.8, M33-M33.99, M60-M60.19, M60.8-M60.9, M79.7	307.8-307.80, 307.89, 330-330.9, 331.3-331.5, 331.8, 331.83, 332.1-333.91, 333.93-338.4, 341-343.8, 344-344.9, 348-348.9, 350-353.0, 353.5-357.5, 357.7-359.23, 359.29-359.9, 710.3-710.4, 725-725.9, 728-728.85, 728.87-728.9, 775.2, 780.96

B2. List of disorders included in other non-communicable neurological disorders

ICD 10	Other non-communicable neurological disorders
G10	G10 Huntington disease
G21.2-G23	G21.2 Secondary parkinsonism due to other external agents
	G21.3 Postencephalitic parkinsonism
	G21.4 Vascular parkinsonism
	G21.8 Other secondary parkinsonism
	G21.9 Secondary parkinsonism, unspecified
	G22 Parkinsonism in diseases classified elsewhere
	G23 Other degenerative diseases of basal ganglia
G24-G26.0	G24 Dystonia
	G24.1 Idiopathic familial dystonia
	G24.2 Idiopathic nonfamilial dystonia
	G24.3 Spasmodic torticollis
	G24.4 Idiopathic orofacial dystonia
	G24.5 Blepharospasm
	G24.8 Other dystonia
	G24.9 Dystonia, unspecified
	G25.0 Essential tremor (Other extrapyramidal and movement disorders)
	G25.2 Other specified forms of tremor
	G25.3 Myoclonus
	G25.5 Other chorea
	G25.8 Other specified extrapyramidal and movement disorders
	G25.9 Extrapyramidal and movement disorder, unspecified
	G26.0 Extrapyramidal and movement disorders in diseases classified elsewhere

G36-G37.9	G36 Other acute disseminated demyelination
	G36.0 Neuromyelitis optica [Devic]
	G36.1 Acute and subacute haemorrhagic leukoencephalitis [Hurst]
	G36.8 Other specified acute disseminated demyelination
	G36.9 Acute disseminated demyelination, unspecified
	G37 Other demyelinating diseases of central nervous system
	G37.0 Diffuse sclerosis
	G37.1 Central demyelination of corpus callosum
	G37.2 Central pontine myelinolysis
	G37.3 Acute transverse myelitis in demyelinating disease of central nervous system
	G37.4 Subacute necrotizing myelitis
	G37.5 Concentric sclerosis [Baló]
	G37.8 Other specified demyelinating diseases of central nervous system
	G37.9 Demyelinating disease of central nervous system, unspecified
G50-G53.8	G50 Disorders of trigeminal nerve
	G50.0 Trigeminal neuralgia
	G50.1 Atypical facial pain
	G50.8 Other disorders of trigeminal nerve
	G50.9 Disorder of trigeminal nerve, unspecified
	G51 Facial nerve disorders
	G51.0 Bell palsy
	G51.1 Geniculate ganglionitis
	G51.2 Melkersson syndrome
	G51.3 Clonic hemifacial spasm
	G51.4 Facial myokymia
	G51.8 Other disorders of facial nerve
	G51.9 Disorder of facial nerve, unspecified
	G52 Disorders of other cranial nerves
	G52.0 Disorders of olfactory nerve
	G52.1 Disorders of glossopharyngeal nerve
	G52.2 Disorders of vagus nerve
	G52.3 Disorders of hypoglossal nerve
	G52.7 Disorders of multiple cranial nerves
	G52.8 Disorders of other specified cranial nerves
	G52.9 Cranial nerve disorder, unspecified
	G53 Cranial nerve disorders in diseases classified elsewhere
	G53.0 Postzoster neuralgia
	G53.1 Multiple cranial nerve palsies in infectious and parasitic diseases classified elsewhere
	G53.2 Multiple cranial nerve palsies in sarcoidosis
	G53.3 Multiple cranial nerve palsies in neoplastic disease
	G53.8 Other cranial nerve disorders in other diseases classified elsewhere
G54-G55.8	G54 Nerve root and plexus disorders
	G54.0 Brachial plexus disorders
	G54.1 Lumbosacral plexus disorders
	G54.5 Neuralgic amyotrophy
	G54.6 Phantom limb syndrome with pain
	G54.7 Phantom limb syndrome without pain
	G54.8 Other nerve root and plexus disorders
	G54.9 Nerve root and plexus disorder, unspecified
	G55 Nerve root and plexus compressions in diseases classified elsewhere
	G55.1 Nerve root and plexus compressions in intervertebral disc disorders
	G55.2 Nerve root and plexus compressions in spondylosis
	G55.3 Nerve root and plexus compressions in other dorsopathies
	G55.8 Nerve root and plexus compressions in other diseases classified elsewhere
G56-G65.2	G56 Mononeuropathies of upper limb
	G56.0 Carpal tunnel syndrome
	G56.1 Other lesions of median nerve
	G56.2 Lesion of ulnar nerve

	G56.3 Lesion of radial nerve
	G56.4 Causalgia
	G56.8 Other mononeuropathies of upper limb
	G56.9 Mononeuropathy of upper limb, unspecified
	G57 Mononeuropathies of lower limb
	G57.0 Lesion of sciatic nerve
	G57.1 Meralgia paraesthetica
	G57.2 Lesion of femoral nerve
	G57.3 Lesion of lateral popliteal nerve
	G57.4 Lesion of medial popliteal nerve
	G57.5 Tarsal tunnel syndrome
	G57.6 Lesion of plantar nerve
	G57.8 Other mononeuropathies of lower limb
	G57.9 Mononeuropathy of lower limb, unspecified
	G58 Other mononeuropathies
	G58.0 Intercostal neuropathy
	G58.7 Mononeuritis multiplex
	G58.8 Other specified mononeuropathies
	G58.9 Mononeuropathy, unspecified
	G59 Mononeuropathy in diseases classified elsewhere
	G60 Hereditary and idiopathic neuropathy
	G60.0 Hereditary motor and sensory neuropathy
	G60.1 Refsum disease
	G60.2 Neuropathy in association with hereditary ataxia
	G60.3 Idiopathic progressive neuropathy
	G60.8 Other hereditary and idiopathic neuropathies
	G60.9 Hereditary and idiopathic neuropathy, unspecified
	G61 Inflammatory polyneuropathy
	G61.0 Guillain-Barré syndrome
	G61.1 Serum neuropathy
	G61.8 Other inflammatory polyneuropathies
	G61.9 Inflammatory polyneuropathy, unspecified
	G62.1 Alcoholic polyneuropathy
	G62.2 Polyneuropathy due to other toxic agents
	G62.8 Other specified polyneuropathies
	G62.9 Polyneuropathy, unspecified
	G63 Polyneuropathy in diseases classified elsewhere
	G63.0 Polyneuropathy in infectious and parasitic diseases classified elsewhere
	G63.1 Polyneuropathy in neoplastic disease
	G63.2 Diabetic polyneuropathy
	G63.3 Polyneuropathy in other endocrine and metabolic diseases
	G63.4 Polyneuropathy in nutritional deficiency
	G63.5 Polyneuropathy in systemic connective tissue disorders
	G63.6 Polyneuropathy in other musculoskeletal disorders
	G63.8 Polyneuropathy in other diseases classified elsewhere
	G64 Other disorders of peripheral nervous system
	G65 Sequelae of inflammatory and toxic polyneuropathies
	G65.0 Sequelae of Guillain-Barré syndrome
	G65.1 Sequelae of other inflammatory polyneuropathy
	G65.2 Sequelae of toxic polyneuropathy
G70-G73.7	G70 Myasthenia gravis and other myoneural disorders
	G70 Myasthenia gravis and other myoneural disorders
	G70.0 Myasthenia gravis
	G70.1 Toxic myoneural disorders
	G70.2 Congenital and developmental myasthenia
	G70.8 Other specified myoneural disorders
	G70.9 Myoneural disorder, unspecified
	G71 Primary disorders of muscles

	G71.0 Muscular dystrophy
	G71.1 Myotonic disorders
	G71.2 Congenital myopathies
	G71.3 Mitochondrial myopathy, not elsewhere classified
	G71.8 Other primary disorders of muscles
	G71.9 Primary disorder of muscle, unspecified
	G72 Other myopathies
	G72.0 Drug-induced myopathy
	G72.1 Alcoholic myopathy
	G72.2 Myopathy due to other toxic agents
	G72.3 Periodic paralysis
	G72.4 Inflammatory myopathy, not elsewhere classified
	G72.8 Other specified myopathies
	G72.9 Myopathy, unspecified
	G72.1 Alcoholic myopathy
	G72.2 Myopathy due to other toxic agents
	G72.3 Periodic paralysis
	G72.4 Inflammatory myopathy, not elsewhere classified
	G72.8 Other specified myopathies
	G72.9 Myopathy, unspecified
	G73 Disorders of myoneural junction and muscle in diseases classified elsewhere
	G73.0 Myasthenic syndromes in endocrine diseases
	G73.1 Lambert-Eaton syndrome
	G73.2 Other myasthenic syndromes in neoplastic disease
	G73.3 Myasthenic syndromes in other diseases classified elsewhere
	G73.4 Myopathy in infectious and parasitic diseases classified elsewhere
	G73.5 Myopathy in endocrine diseases
	G73.6 Myopathy in metabolic diseases
	G73.7 Myopathy in other diseases classified elsewhere
G81-G83.9	G81 Hemiplegia
	G81.0 Flaccid hemiplegia
	G81.1 Spastic hemiplegia
	G81.9 Hemiplegia, unspecified
	G82 Paraplegia and tetraplegia
	G82.0 Flaccid paraplegia
	G82.1 Spastic paraplegia
	G82.2 Paraplegia, unspecified
	G82.3 Flaccid tetraplegia
	G82.4 Spastic tetraplegia
	G82.5 Tetraplegia, unspecified
	G83 Other paralytic syndromes
	G83.0 Diplegia of upper limbs
	G83.1 Monoplegia of lower limb
	G83.2 Monoplegia of upper limb
	G83.3 Monoplegia, unspecified
	G83.4 Cauda equina syndrome
	G83.8 Other specified paralytic syndromes
	G83.9 Paralytic syndrome, unspecified
G89-G89.4	G89 Pain, not elsewhere classified
	G89.0 Central pain syndrome
	G89.1 Acute pain, not elsewhere classified
	G89.11 Acute pain due to trauma
	G89.12 Acute post-thoracotomy pain
	G89.18 Other acute postprocedural pain
	G89.2 Chronic pain, not elsewhere classified
	G89.21 Chronic pain due to trauma
	G89.22 Chronic post-thoracotomy pain
	G89.28 Other chronic postprocedural pain

	G89.29 Other chronic pain
	G89.3 Neoplasm related pain (acute) (chronic)
	G89.4 Chronic pain syndrome
G90-G90.9	G90 Disorders of autonomic nervous system
	G90.0 Idiopathic peripheral autonomic neuropathy
	G90.1 Familial dysautonomia [Riley-Day]
	G90.2 Horner syndrome
	G90.3 Multi-system degeneration
	G90.4 Autonomic dysreflexia
	G90.8 Other disorders of autonomic nervous system
	G90.9 Disorder of autonomic nervous system, unspecified
G91-G91.9	G91 Communicating hydrocephalus
	G91.1 Obstructive hydrocephalus
	G91.2 Normal-pressure hydrocephalus
	G91.3 Post-traumatic hydrocephalus, unspecified
	G91.8 Other hydrocephalus
	G91.9 Hydrocephalus, unspecified
G92	G92 Toxic encephalopathy
G93-G93.9	G93 Other disorders of brain
	G93.0 Cerebral cysts
	G93.1 Anoxic brain damage, not elsewhere classified
	G93.2 Benign intracranial hypertension
	G93.3 Postviral fatigue syndrome
	G93.4 Encephalopathy, unspecified
	G93.5 Compression of brain
	G93.6 Cerebral oedema
	G93.8 Other specified disorders of brain
	G93.9 Disorder of brain, unspecified
G94-G94.8	G94 Other disorders of brain in diseases classified elsewhere
	G94.0* Hydrocephalus in infectious and parasitic diseases classified elsewhere (A00-B99+)
	G94.1* Hydrocephalus in neoplastic disease (C00-D48+)
	G94.2* Hydrocephalus in other diseases classified elsewhere
	G94.8* Other specified disorders of brain in diseases classified elsewhere
G95-G95.9	G95 Other diseases of spinal cord
	G95.0 Syringomyelia and syringobulbia
	G95.1Vascular myelopathies
	G95.2 Cord compression, unspecified
	G95.8 Other specified diseases of spinal cord
	G95.9 Disease of spinal cord, unspecified
G96-G96.9	G96 Other disorders of central nervous system
	G96.0 Cerebrospinal fluid leak
	G96.1 Disorders of meninges, not elsewhere classified
	G96.12 Meningeal adhesions
	G96.8 Other specified disorders of central nervous system
	G96.9 Disorder of central nervous system, unspecified
G98	G98 Other disorders of nervous system, not elsewhere classified
G99-G99.8	G99.0* Autonomic neuropathy in endocrine and metabolic diseases
	G99.1* Other disorders of autonomic nervous system in other diseases classified elsewhere
	G99.2* Myelopathy in diseases classified elsewhere
	G99.8* Other specified disorders of nervous system in diseases classified elsewhere
M33-M33.9	M33 Dermatopolymyositis
	M33.0 Juvenile dermatomyositis
	M33.1 Other dermatomyositis
	M33.2 Polymyositis
	M33.9 Dermatopolymyositis, unspecified
M60-M60.9	M60 Myositis
	M60.0 Infective myositis
	M60.1 Interstitial myositis

	M60.2 Foreign body granuloma of soft tissue, not elsewhere classified
	M60.8 Other myositis
	M60.9 Myositis, unspecified
M79.7	M79.7 Fibromyalgia

C. GBD data and analysis framework

The overview of data inputs and analysis framework for GBD is shown in the following flowchart:

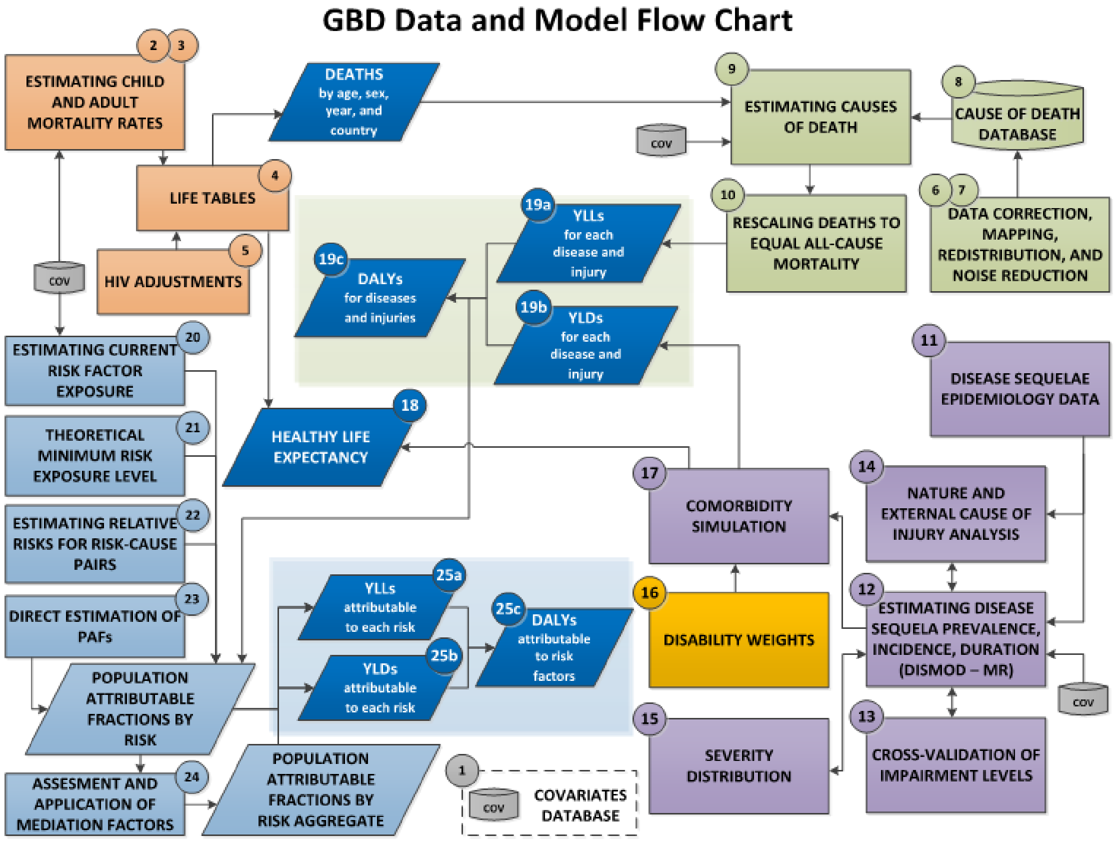

YLLs is years of life lost. YLDs is years lived with disability. DALYs is disability-adjusted life-years. PAFs is population attributable fractions. Rectangular boxes represent analytical steps, cylinders represent databases, and parallelograms represent intermediate and final results.

The flowchart above illustrates the flow of the key components of the GBD estimation process, including:

1. Incorporation of appropriate covariates (step 1)
2. All-cause mortality estimation (steps 2-5): the data come from sources such as censuses, surveys and vital registrations. The all-cause mortality estimation process (steps 2-4) can be divided into four distinct but interconnected areas: child mortality and adult mortality between ages 15 and 60, estimation of a complete set of age-specific death rates, estimation of HIV mortality and final estimates of age-specific mortality including HIV and fatal discontinuities (also known as mortality shocks) (step 5).
3. Causes of death estimation (steps 6-9): cause of death data are derived from vital registrations, verbal autopsy studies, mortality surveillance and, for selected causes, police records, crime reports and data collection systems for deaths due to conflict and natural disasters (step 7). Extensive data corrections and redistributions of ill-defined causes are made to correct for measurement bias between data sources. Cause of death ensemble modelling (CODEm), an ensemble model, is a systematized approach to analysing cause of death data for all but a few causes (step 9). CODEm explores a wide range of modelling approaches and varying predictive covariates to find an ensemble of best-performing models based on statistical tests. To do so, 30% of the data were withheld from each model and the model fit is evaluated by how well it covers the data that were left out. By repeating this process many times over the best performing models are selected. As all results in GBD are estimated 1,000 times over to propagate all sources of uncertainty, among the 1,000 runs we end up with an ensemble of up to 100 or more different types of models and covariates that are selected among the 1,000 runs.
4. Rescaling deaths to equal all-cause mortality (step 10): as all these estimates were made separately for each disease and injury, the sum of these could exceed or fall below the all-cause mortality estimated from the demographic analyses of steps 2 to 5. Therefore, all deaths by age, sex, geography, year and cause to match the all-cause death estimates (this process was called CoD Correct).

5. Estimation of disease sequelae prevalence, incidence, and duration (steps 11-12): population surveys, cohort studies, administrative records of hospitalisations and other health service encounters, disease registries, notifications, surveillance systems are the main data sources for non-fatal estimation (step 11). Extensive corrections of data to deal with measurement bias arising from study design or case definitions are applied. DisMod-MR 2.1 is the main analytical tool for non-fatal estimation (step 12). It is a Bayesian meta-regression software program that uses a lognormal model. The meta-regression component allows corrections for known sources of measurement error. Its core function is to make estimates of prevalence and incidence of disease that are consistent with data on mortality risk and remission (defined in GBD as the 'cure rate'). For a select number of causes that do not fit well in the three states model (alive without disease, prevalent case of disease and death) of DisMod-MR 2.1, we use alternative modelling strategies.
6. Cross-validation of impairment levels (step 13): for a number of impairments in GBD terminology, such as anaemia, heart failure, hearing and vision loss, we first estimate the total levels of prevalence and incidence and then ensured that all sequelae of diseases that lead to this impairment add up to the total.
7. Analysis of the nature and external cause of injury is done separately (step 14). Assignment of severity distributions for the main disabling conditions (step 15): In GBD, terminology sequelae are the disabling consequences for which we make estimates. All sequelae are defined to be mutually exclusive and collectively exhaustive. Many diseases have sequelae with a gradation by severity such as mild, moderate and severe dementia. Often the epidemiological data on severity distribution is sparse. Therefore, we first model the epidemiology of all cases of disease and then apply a severity distribution from the sparser data.
8. Assignment of disability weights for health states (step 16): each sequela is matched with a health state or combination of health states for which we have a disability quantifies the relative severity.
9. Disability weights were derived from population and internet surveys of over 60,000 respondents answering pair-wise comparison question of random combinations of health states. Each pair of health states was described with brief lay descriptions highlighting the main symptoms and impairments. Respondents were asked to nominate the 'healthier' of each presented pair. Analytical methods exist to formalise the intuition that if the majority of respondents nominate one health state in a pair as the healthier these lie farther apart on a severity scale than pairs assigned similar proportions as the healthier. In order to anchor estimates on a 0-1 scale of severity, a subset of respondents was asked additional population health equivalence questions on a selection of health states. These questions ask for a choice of the greater amount of health produce by two health programs; one that prevented sudden death in 1,000 persons and another that prevented the onset of a GBD health state for the rest of 2,000, 5,000 or 10,000 persons' lives.
10. Simulation of comorbidity (step 17): the last step of non-fatal estimation is a microsimulation ('COMO') to deal with comorbidity. For every age, sex, geography and year, 40,000 hypothetical persons were generated who have none, one or more of the GBD sequelae. In those with multiple sequelae their combined level of disability is estimated multiplicatively. That means we assume the disability from having two health states is less than the sum of the corresponding disability weights. This avoids assigning disability greater than one to any individual, which would indicate that person is worse off than being dead.
11. Estimation of healthy life expectancy (step 18): health life expectancy is estimated from the life tables generated in step 4 and the all-cause YLD rates from step 19b.
12. Computation of YLLs, YLDs, and DALYs from diseases and injuries with uncertainty (steps 19a-19c): YLLs (step 19a) are estimated as the product of counts of death by ages, sex, geography, year and cause and a normative life expectancy at the age of the death. The GBD standard life expectancy used as this norm is a compilation of the lowest observed mortality rates by age in all mortality data collections of populations greater than 5 million. The standard life table reflects a life expectancy at birth of 86.59 years. YLDs are the output from COMO (step 19b). DALYs are the simple addition of YLLs and YLDs (step 19c).
13. Risk factor estimation (steps 20-24): GBD 2019 also makes estimates for individual and combined risk factors. This involves estimation of risk factor exposure (step 20); the formulation of a minimum level of exposure to each risk that is associated with the least amount of health loss (step 21); derivation of relative risks of disease outcomes for each pair of a risk factor and a disease or injury for which there is judged to be sufficient evidence of a causal relationship (step 22); and the estimation of population attributable fractions of disease caused by each risk factor. For a few risk-outcome pairs it is hard to define exposure and a corresponding risk while directly observed proportions of disease are available, such as for the proportion of HIV/AIDS due to unsafe sex or injecting drug use (step 23). For combinations of risks how much of the risk is mediated through other risks (step 24) was assessed. For instance, all of the effect of high salt intake is mediated through elevated blood pressure and part of the risk of increased body mass index is through elevated blood pressure, cholesterol or fasting plasma glucose.
14. Computation of YLLs, YLDs, and DALYs attributable to risk factors (steps 25a-25c): YLLs, YLDs and DALYs attributable to each risk factor are generated by multiplying population attributable fractions with disease estimates (steps 25a-c).

D. Neurological disorders morbidity estimation

The major data inputs used for estimating prevalence or incidence of neurological disorders in India are population-based published studies. Neurological disorders morbidity was modelled using the DisMod-MR 2.1 platform. Morbidity estimation for the neurological disorders presented in this paper are described below.

D1. Stroke

The steps in the estimation of non-fatal stroke burden are shown in the following flowchart:

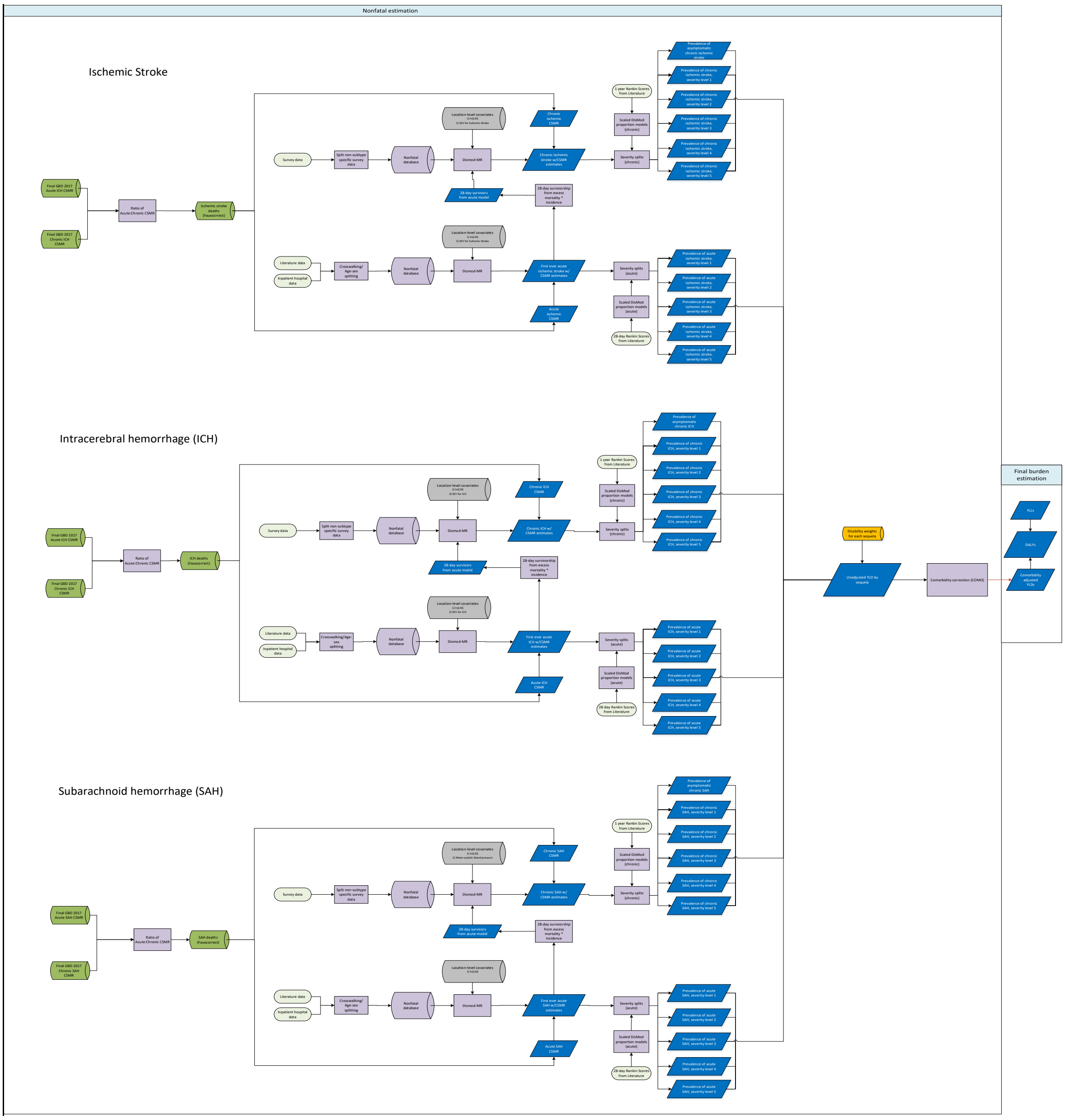

Data

GBD included inpatient hospital data, adjusted for readmission and primary to any diagnosis using correction factors estimated from US claims data. In addition, unpublished stroke registry data were included for acute ischaemic stroke, acute intracerebral haemorrhage, and acute subarachnoid haemorrhage. Survey data were also included for chronic stroke. These surveys were identified based on expert opinion and review of major survey series focused on world health that included questions regarding self-reported history of stroke. Unspecified strokes (ICD-10 I64) were split into ischaemic stroke, intracerebral haemorrhage, and subarachnoid

haemorrhage according to the proportions of subtype specific coded strokes in the original data. ICD-10 I62 was also split into intracerebral haemorrhage, and subarachnoid haemorrhage using the same approach.

As the data sources available are diverse, GBD adjusted these data to the preferred or reference case definition. For this, incidence and excess mortality data that did not meet the reference case definitions were cross-walked using MR-BRT, a bayesian meta-regression tool. Data points for first and recurrent strokes combined were adjusted using data for first strokes only as reference. For ischaemic stroke and intracerebral haemorrhage, data points that reported all stroke subtypes combined were adjusted using studies with subtype-specific information as reference. Data which included only persons who survived to hospital admission were adjusted using data on both fatal and nonfatal strokes as reference. Additionally, subtype-specific, inpatient clinical informatics data were adjusted using subtype-specific literature estimates as a reference. The formula for computing adjustment factors is given in equation below. A standardized age variable (age scaled) and a sex variable were also included to the cross-walking procedure to adjust for the possibly of bias.

$$Estimated\ Reference\ Def = invlogit(logit(Alternative\ Def) - Beta_{Alternative\ Def} - Beta_{Sex} * Sex - Beta_{Age_{scaled}} * Age\ Scaled)$$

Severity splits and disability weights

The tables below illustrate the severity level, lay description, and disability weights for stroke. A review of literature identified the epidemiologic studies which reported the degree of disability at 28 days (for acute stroke) or one year (for chronic stroke) using the modified Rankin scale (mRS) and the Mini-Mental State Examination (MMSE) or the Montreal Cognitive Assessment (MoCA). The mRS assesses functional capabilities, while the MMSE and MoCA tests provide evaluations of cognitive functioning. These measures were then mapped to the existing GBD categories as indicated below.

Severity distribution, details on the severity levels for acute stroke and the associated disability weights with that severity

Severity level	Lay description	Modified Rankin score	Cognitive status	Disability weights (95% CI)
Stroke, mild	Has some difficulty in moving around and some weakness in one hand, but is able to walk without help.	1	N/A	0.019 (0.01–0.032)
Stroke, moderate	Has some difficulty in moving around, and in using the hands for lifting and holding things, dressing, and grooming.	2, 3	MoCA>=24 or MMSE>=26	0.07 (0.046–0.099)
Stroke, moderate plus cognition problems	Has some difficulty in moving around, in using the hands for lifting and holding things, dressing and grooming, and in speaking. The person is often forgetful and confused.	2, 3	MoCA<24 or MMSE<26	0.316 (0.206–0.437)
Stroke, severe	Is confined to bed or a wheelchair, has difficulty speaking, and depends on others for feeding, toileting, and dressing.	4, 5	MoCA>=24 or MMSE>=26	0.552 (0.377–0.707)
Stroke, severe plus cognition problems	Is confined to bed or a wheelchair, depends on others for feeding, toileting, and dressing, and has difficulty speaking, thinking clearly, and remembering things.		MoCA<24 or MMSE<26	0.588 (0.411–0.744)

Severity distribution, details on the severity levels for chronic stroke and the associated disability weights with that severity

Severity level	Lay description	Modified Rankin score	Cognitive status	Disability weights (95% CI)
Stroke, asymptomatic		0	N/A	N/A
Stroke, long-term consequences, mild	Has some difficulty in moving around and some weakness in one hand, but is able to walk without help.	1	N/A	0.019 (0.01–0.032)
Stroke, long-term consequences,	Has some difficulty in moving around, and in using the hands for lifting and holding things,	2, 3	MoCA>=24 or	0.07 (0.046–0.099)

moderate	dressing, and grooming.		MMSE>=26	
Stroke, long-term consequences, moderate plus cognition problems	Has some difficulty in moving around, in using the hands for lifting and holding things, dressing and grooming, and in speaking. The person is often forgetful and confused.	2, 3	MoCA<24 or MMSE<26	0.316 (0.206–0.437)
Stroke, long-term consequences, severe	Is confined to bed or a wheelchair, has difficulty speaking, and depends on others for feeding, toileting, and dressing.	4, 5	MoCA>=24 or MMSE>=26	0.552 (0.377–0.707)
Stroke, long-term consequences, severe plus cognition problems	Is confined to bed or a wheelchair, depends on others for feeding, toileting, and dressing, and has difficulty speaking, thinking clearly, and remembering things.	4, 5	MoCA<24 or MMSE<26	0.588 (0.411–0.744)

DisMod-MR 2.1, a Bayesian meta-regression tool, was used to model the six severity levels, with an independent proportion model for each. Reports which grouped mRS scores differently than the mapping (eg, 0-2) were adjusted in DisMod by estimating the association between these alternate groupings and the preferred mappings. These statistical associations were used to adjust data points to the referent category as necessary. The six models were scaled such that the sum of the proportions for all levels equalled.

Modelling strategy

The general approach employed for all of the components of the stroke modelling process is detailed elsewhere (Lancet 2020; 396: 1204–22). Data points were adjusted from alternative to reference case definitions using estimates from statistical models generated by MR-BRT for the acute models. The summary exposure values (SEV), which are the relative risk-weighted prevalence of exposure, were included as covariates for the ischaemic stroke or intracerebral haemorrhage models as appropriate, and a covariate for country income was used as a country-level covariate for both models. Subarachnoid haemorrhage did not include an SEV covariate, but did include a covariate for country income for excess mortality. GBD used the ratio of acute:chronic cause-specific mortality estimated by the final DisMod model estimates to divide stroke deaths into acute and chronic stroke deaths, using the global average for the proportion of acute chronic stroke mortality. The acute and chronic models were then run using the same incidence, prevalence, and case fatality data as well as the custom cause-specific mortality rates as input data.

The first-ever acute subtype-specific models were run with CSMR as derived from FauxCorrect and epidemiological data as described above using Dismod-MR. GBD then calculated the rate of surviving until 28 days after an acute event for all three subtypes using the modelled estimates of excess mortality and incidence from the acute stroke models. Twenty-eight-day survivorship data was uploaded into the chronic subtype-specific with CSMR models. These chronic models also use CSMR as derived from FauxCorrect and epidemiological data as described above. Models were evaluated based on expert opinion, comparison with previous iterations, and model fit. Models were evaluated based on expert opinion, comparison with previous iterations, and model fit. The covariates used by cause in the estimation process, as well as the beta and exponentiated beta values are reported elsewhere (Lancet 2020; 396: 1204–22).

D2. Headache disorders

The steps in the estimation of non-fatal headache disorders burden are shown in the following flowchart:

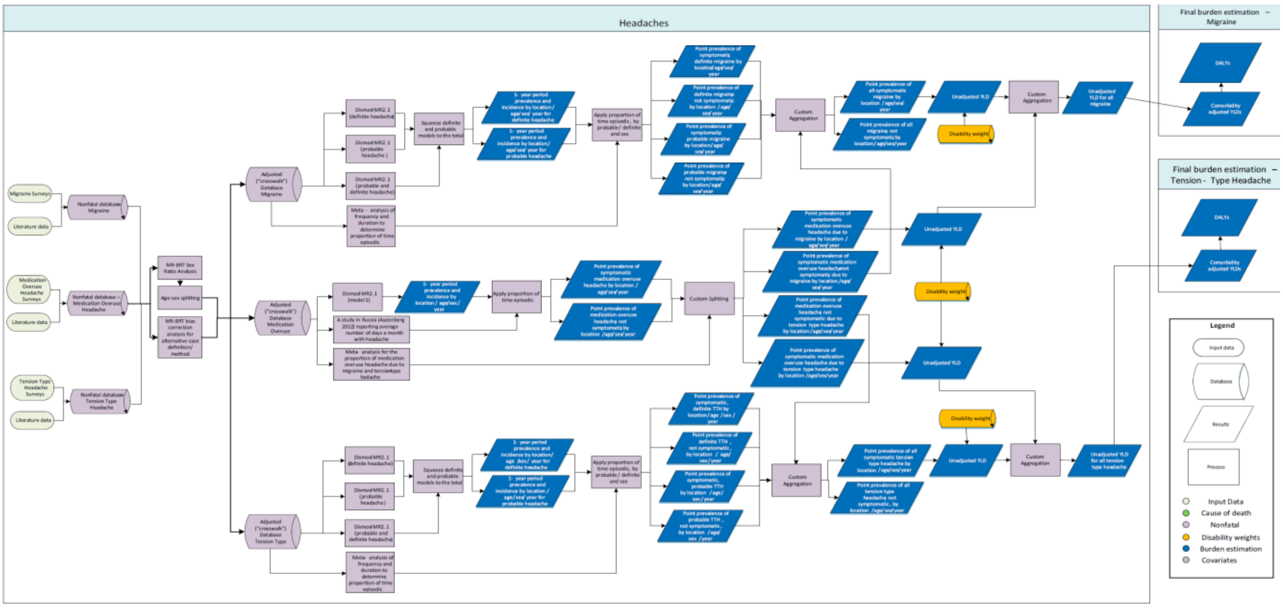

Data

Data from population-based representative surveys that reported the prevalence of migraine or tension-type headache available from a systematic review were used.

Age and sex splitting

Reported estimates of prevalence were split by age and sex where possible. First, if studies reported prevalence for broad age groups by sex, and also by specific age groups but for both sexes combined, age-specific estimates were split by sex using the reported sex ratio and bounds of uncertainty. Second, prevalence data for both sexes that could not be split using a within-study ratio were split using a sex ratio derived from a meta-analysis of existing sex-specific data using MR-BRT. Finally, after the application of bias adjustments, where studies reported estimates across age groups spanning 25 years or more, these were split into five-year age groups using the prevalence age pattern estimated by the best DisMod-MR 2.1 for each headache type.

Data adjustment

A list of binary adjustment criteria which are a modified version of quality indicators of epidemiological studies on headache as shown in the table below were used.[1]

**Study covariate**	**Notation**	
	**Less desirable (1)**	**Reference (zero)**
Other than one-year recall period	Point prevalence	One-year prevalence
Not representative	Selected population	General population or community-based sample from whole country OR general population or community-based sample from defined region within a country, or school-based (for children)
Low-quality sampling method	Not stated OR no (or failed) attempt to secure representativeness	Total defined population, or random sample corrected for population demographics OR random sample uncorrected for population demographics
Poor response	Not stated, or <70%	70–100%
Low-quality survey method and type of interviewer	Not stated OR self-administered (unsupervised) questionnaire OR telephone or face-to-face interview by untrained or unspecified interviewer(s)	Face-to-face interview with headache expert or trained interviewer
Low-quality validation of diagnostic instrument	Instrument not specified or not validated OR validated, but sensitivity and/or specificity <70% OR validated only in screen-positive	Validated in target population or similar, and sensitivity and specificity ≥70%, or all diagnoses made in face-to-face or telephone interviews by headache expert

	sub-sample, or in clinic or unspecified sample, but sensitivity and specificity ≥70%	
Low-quality diagnostic criteria	Not stated OR stated, other than ICHD OR ICHD (or reasonable modification)	ICHD (or reasonable modification)

The mean and standard error for the coefficients were calculated using the MR-BRT adjustment method. All study covariates were initially evaluated independently for each type of headache. However, covariate values varied not only in magnitude but in direction across these headache types. Because GBD assumes that the same study covariate should adjust data at least in the same direction for these headache types, the final study covariates were evaluated taking all available headache data into account.

Modelling strategy

Migraine

Separate DisMod models were run for estimation of definite migraine, probable migraine, and the total migraine category and upper bound on remission was set 0.1 across all models. Afterwards, the results were scaled of probable and definite headache to the total headache envelope to ensure consistency.

Because some data sources, especially earlier data from before International Classification of Headache Disorders (ICHD) became the standard (the initial criteria were published in 1988), largely report on definite migraine, studies were adjusted that reported only on definite migraine to the total migraine category in order to better inform that model. All data that reported on both definite and total migraine were used in regression models by sex in order to derive an age and sex specific adjustment.

Tension-type headache

DisMod models were run for definite tension-type headache, probable tension-type headache, and the total tension-type headache category, setting an upper bound on remission of 0.5 across all models. Afterwards, the results of probable and definite headache to the total headache envelope to ensure consistency.

Because some data sources, especially earlier data from before ICHD became the standard largely report on definite tension-type headache, studies were adjusted that reported only on definite tension-type headache to the total tension-type headache category in order to better inform that model. Initially, all data that reported on both definite and total tension-type headache were used in regression models by sex in order to derive an age- and sex-specific adjustment. These sex-specific models resulted in an implausible age pattern for females such that the age-pattern of the age-split data points was the inverse of the original data. Consequently, a regression model was used to derive an age-specific adjustment that was applied to both sexes.

Reference

1. Steiner, T.J., Stovner, L.J., Al Jumah, M. et al. Improving quality in population surveys of headache prevalence, burden and cost: key methodological considerations. *J Headache Pain* 2013; 14: 87.

D3. Epilepsy

The steps in the estimation of non-fatal epilepsy burden are shown in the following flowchart:

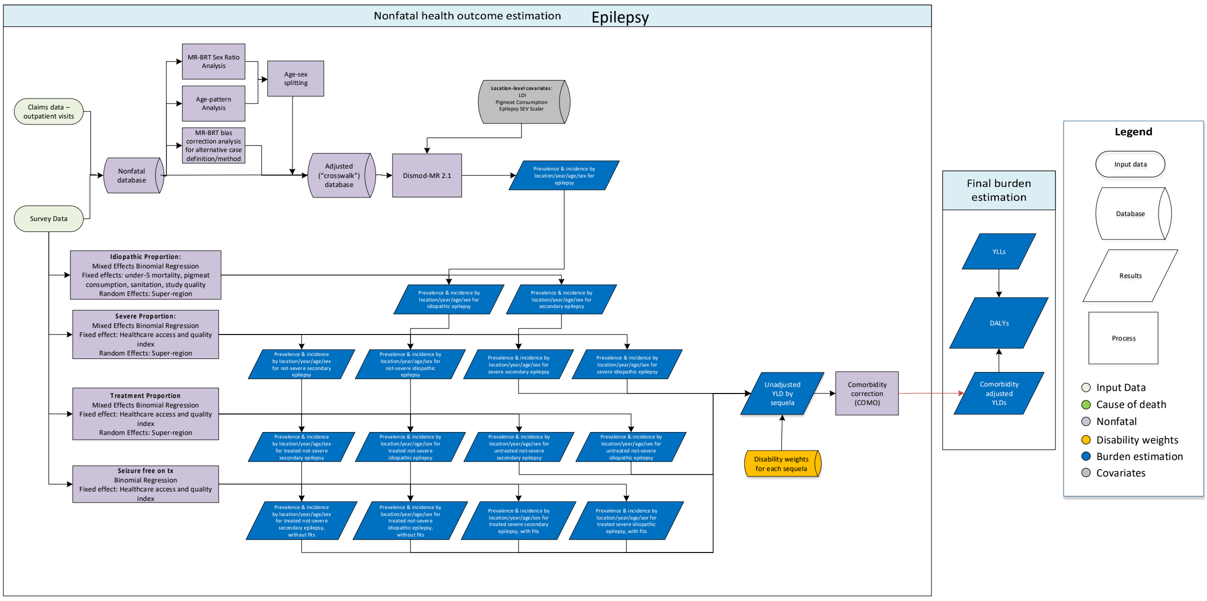

Data

Population-based studies were included if they reported on prevalence, incidence, remission rate, and excess mortality rate, relative risk of mortality, standardised mortality ratio, or with condition mortality rate. Studies were excluded if there is no clearly defined sample (e.g. among clinic attenders or patient organisation members with non-specific or non-representative catchment area).

Modelling strategy

The prevalence of epilepsy was modelled in two steps: first, an epilepsy impairment envelope was created and second, the envelope was split into primary (or idiopathic) and secondary epilepsies. Each of these were subdivided into severe (on average one or more fits per month) and 'non-severe' epilepsies. No severe cases were subdivided into 'treated' and 'un-treated' epilepsy. Treated cases were then subdivided into treated cases with fits (one and 11 fits on average in the preceding year) and treated cases without fits (no fits reported in the preceding year).

In the first step, DisMod-MR 2.1 was used for the epilepsy impairment envelope to model a consistent fit between incidence, prevalence, remission, and standardised mortality ratio data. The table below illustrates the covariates, parameters, beta and exponentiated beta values for epilepsy.

Study covariate	Parameter	Beta	Exponentiated beta
Log-transformed age-standardised SEV scalar for epilepsy	Prevalence	0.76	2.14 (2.12-2.21)
LDI (I$ per capita)	Excess mortality rate	-0.55	0.58 (0.37-0.90)

In the second step, mixed-effects generalized linear models (binomial family) were used to predict the proportion of idiopathic epilepsy, the proportion of severe epilepsy, the proportion of treated epilepsy and the proportion of epilepsy that is treated without fits.

Because not all of the data on the proportion of idiopathic epilepsy use optimal case finding methods (using CT scans or MRIs in addition to EEGs in order to diagnose secondary epilepsy), first an initial linear regression model was run with a covariate on study quality. Then the beta from this model was used to crosswalk studies with non-optimal case finding methods to those with adequate methods. The adjusted data are then used in the regression for the proportion of epilepsy that is idiopathic, with a fixed effect on SDI as well as a random effect on GBD super-region.

Similar models were used to predict the proportion of severe epilepsy and treatment gap based on the reported proportions extracted from the systematic review. To predict the proportion of severe epilepsy and the treatment gap, mixed-effects models was used with a fixed effect on the log of Healthcare Access and Quality (HAQ) Index and a random effect on GBD super-region. The generalized linear model (binomial family) was used to generate predictions for the proportion of treated epilepsy that is seizure-free with a fixed effect on the log of HAQ Index.

A fixed effect on epilepsy cause-specific mortality, under-5 mortality rate, sanitation, and pig meat consumption as well as random effects on region and country was tested in different models, but they did not improve the models. Country-specific 1,000 draws of estimates were generated for each year between 1980 and 2019 for each of the models.

Severity splits and disability weights

The table below illustrates the severity levels, descriptions, and disability weights associated with epilepsy. These are calculated using regressions from literature (i.e. frequency of seizures).

**Severity level**	**Lay description**	**Disability weights (95% CI)**
Severe (seizures >= once per month)	This person has sudden seizures one or more times each month, with violent muscle contractions and stiffness, loss of consciousness, and loss of urine or bowel control. Between seizures the person has memory loss and difficulty concentrating.	0.552 (0.375-0.71)
less severe (seizures < once per month)	This person has sudden seizures two to five times a year, with violent muscle contractions and stiffness, loss of consciousness, and loss of urine or bowel control.	0.263 (0.173 0.367)
Treated without fits	This person has a chronic disease that requires medication every day and causes some worry but minimal interference with daily activities.	0.049 (0.031 0.072)

D4. Alzheimer's disease & other dementias

The steps in the estimation of non-fatal Alzheimer’s disease & other dementias burden are shown in the following flowchart:

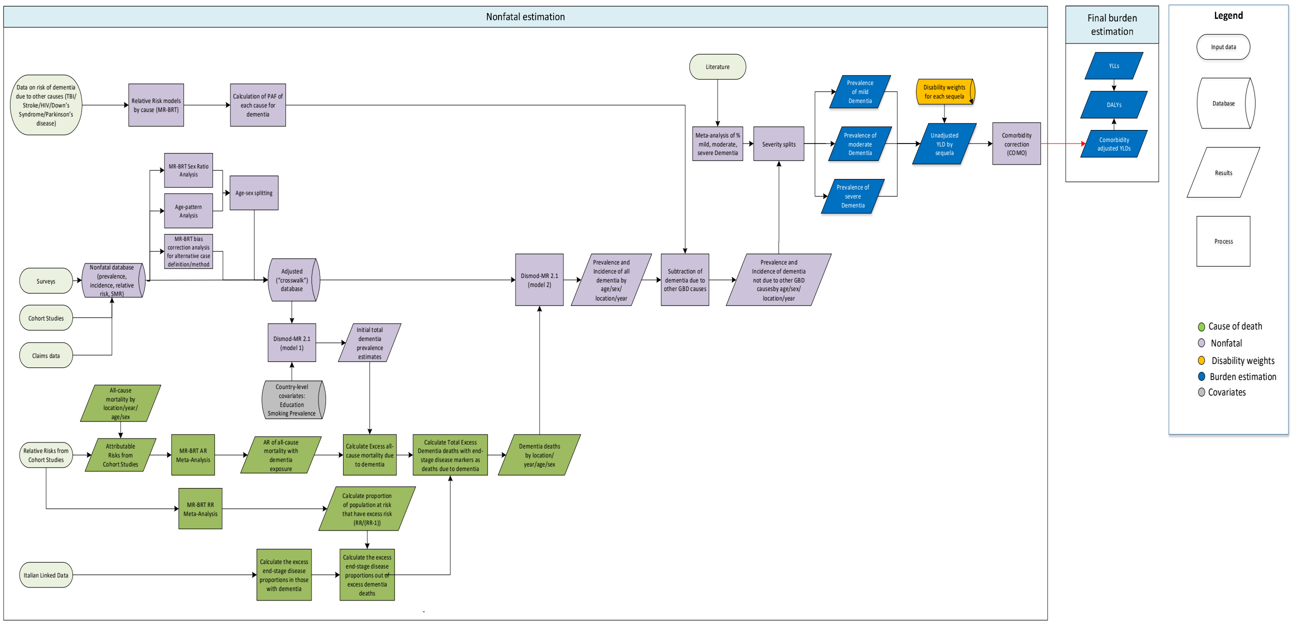

Unlike most causes in the GBD, dementia mortality and morbidity estimates are modelled jointly. This is because of marked discrepancies between prevalence data and cause of death data. Specifically, prevalence data suggest little to no variation over time, whereas age-standardised mortality rates in vital registrations in high-income countries have increased multiple times over this same period. Additionally, prevalence variation between countries is much smaller than the variation in death rates assigned to dementia in vital registration. These discrepancies were attribute to changing coding practices rather than epidemiological change.

Data

To inform estimates of burden due to dementia, mortality data from relative risk studies and linked hospital to mortality data, as well as prevalence data from surveys and administrative data were used.

Item Response Theory for prevalence prediction

The prevalence models for dementia are data sparse, and there are not many surveys done in low-income settings. However, there are a larger body of surveys that collect data on cognitive tests and functional limitations which are the two main components of a DSM or ICD diagnosis. Predictions of dementia prevalence using information from these questions would allow for expanded data coverage and additional information in locations where there are currently no data guiding estimates.

Generating these predictions requires calibrating a model to samples that have information about both functional limitations, cognition and adjudicated dementia diagnoses. However, making comparisons across surveys can be difficult, as each survey asks a different set of questions about cognition and limitations, although there is some overlap. This overlap allows for the use of item response theory methods for the harmonization of these scales. Once the scales are harmonized the subsamples can be utilized to create a model for the prediction of prevalence.

Excluding incidence

Incidence data were excluded, because in locations with high quality cohort data on prevalence and incidence, the two are not compatible (incidence data implies a higher prevalence than what is reported). Because dementia has a slow, insidious onset and prevalence is easier to measure, GBD trust prevalence data more and rely on this, excluding incidence data from DisMod.

Severity splits

A systematic review was conducted to collect information on the proportion of individuals in each dementia severity class out of the population of all individuals with dementia. The Clinical Dementia Rating (CDR) scale was taken as the reference definition for severity classification, along with a doctor-given diagnosis according to DSM III, IV, V or ICD case definitions as our reference definition for dementia.

However, as a neurodegenerative disorder with a wide range of categories in which symptoms manifest, there are an abundance of classification tools which discern between severity levels along different criteria. GBD accepted severities classified by:

- Clinical dementia rating sum-of-boxes (CSR-SB)
- Blessed test of information, memory, and concentration (BIMC)
- Global deterioration scale (GDS)
- Geriatric Mental State Examination (GMS)
- CAMDEX
- DSM-III-R
- Karasawa's

GBD excluded any studies which classified dementia severity according to scales that only evaluated cognitive function and memory, excluding activities of daily living (ADLs). The most prominent such scale is Mini Mental State Examination (MMSE). The table below illustrates the severity levels, descriptions, and disability weights associated with Alzheimer's disease & other dementias.

Severity level	Lay description
Mild	The person has some trouble remembering recent events and finds it hard to concentrate and make decisions and plans. They may have slight to moderate difficulty engaging in community affairs, complicated hobbies, and intellectual interests.
Moderate	The person retains highly learned material, but has severe memory problems, is disoriented with respect to time and sometimes place. They are severely impaired in their ability to handle problems and make social judgements. They require assistance with daily activities, and only retain simple chores and hobbies.
Severe	The person has complete memory loss, no longer recognizes close family members, and requires help with all daily activities, including personal care.

Modelling strategy

The prevalence data was sex split, cross-walked and age split. Studies with age and sex detail separately were split into age- and sex-specific data points. Data specified as "both" sex data were split into male- and female-specific data points using MR-BRT to get a model ratio of female/male prevalence and then using the following equations:

Male prevalence:

$$prev_{male} = prev_{both} * \frac{pop_{both}}{(pop_{male} + ratio * pop_{female})}$$

Female prevalence:

$$prev_{female} = ratio * prev_{male}$$

Data points were split where the age range was greater than 25 years using the global age pattern. Dementia studies are heterogeneous. Even with a smaller number of definitions (DSM/ICD), there are a large number of different ways to diagnose dementia. Most use a two-step procedure, where screening is first done using a cognitive test to then fully evaluate those that fall below a certain pre-defined threshold. Differences in methods were controlled by crosswalking alternative case definitions to reference. Study covariates are based on broad categories determined after going through the diagnostic heterogeneity and there are some added for specific criteria that GBD know are biased. Crosswalking was carried out using a logit difference network meta-regression analysis.

Two country-level covariates were included in the initial DisMod model. Age-standardised education was used as a proxy for general brain health/use that may be protective of dementia – specifically Alzheimer's disease. Smoking prevalence (age-standardised, both sexes) was also used as a covariate to guide estimates, as the literature has shown a positive relationship between smoking and dementia.

Two DisMod models were run with prevalence inputs – the first uses adjusted prevalence data (DisMod Model 1 in flowchart), which accounts for dementia caused by other diseases. The second uses unadjusted dementia (DisMod Model 2 in flowchart) which accounts for all dementia regardless of cause (this is the dementia impairment envelope).

The cause-specific mortality results from final fatal estimates were pulled into a final DisMod model (Model 2), with the same settings as the previous models. To prevent double counting of prevalent cases, both under dementia and under other causes that can lead to dementia, GBD adjusted the dementia prevalence to exclude cases caused by these other conditions, which include stroke, Parkinson's disease, traumatic brain injury and Down's Syndrome. For this GBD used data from the Aging, Demographics and Memory study (ADAMS) and new systematic reviews, to estimate the relative risk of getting dementia for each condition included in the ADAMS dataset (stroke, Parkinson's disease, TBI). GBD first fit logistic regression models predicting the outcome of dementia given each exposure, with an additional covariate on age.

GBD then used these models to predict the probability of dementia given each exposure at various ages and divided the probability of having dementia by the probability of not having dementia at each age to calculate relative risks. After calculating age specific relative risks, these data and estimates of dementia prevalence from DisMod-MR 2.1 model was used to calculate the population attributable fractions (PAFs) for each cause and age using the formula:

$$PAF = \frac{prevalence * (RR - 1)}{prevalence * (RR - 1) + 1}$$

Finally, the PAF was multiplied by the total prevalence to get the amount of dementia prevalence that can be attributed to each cause and subtracted this from the total prevalence to get the prevalence of dementia that is not due to other GBD causes.

D5. Brain and central nervous system cancer

The steps in the estimation of non-fatal brain and central nervous system (CNS) cancer burden is shown in the following flowchart:

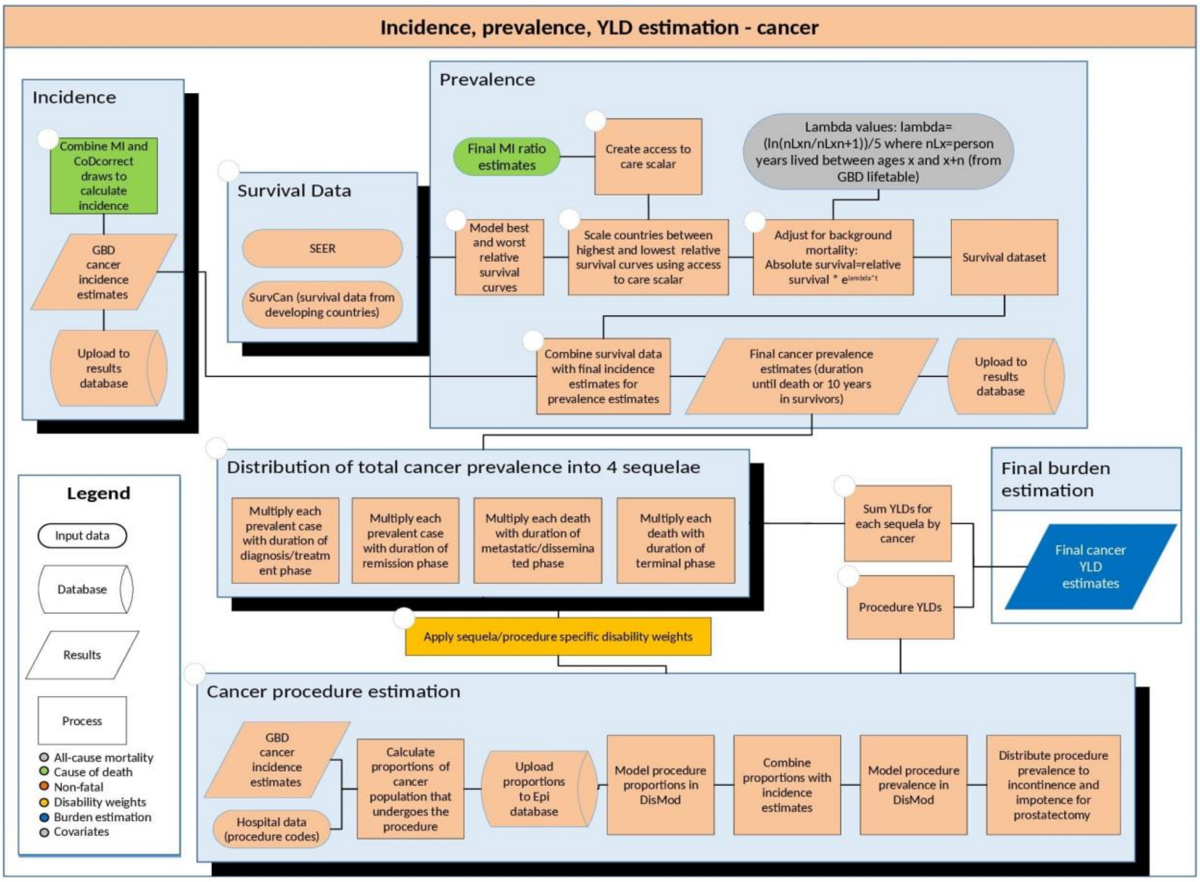

To estimate disability for each cancer, total prevalence is split into four sequelae: 1. diagnosis and primary therapy; 2. controlled phase; 3. metastatic phase; and 4. terminal phase. The diagnosis and primary therapy phase is defined as the time from the onset of symptoms to the end of treatment. The controlled phase is defined as the time between finishing primary treatment and the earliest of either: cure (defined as recurrence- and progression-free survival after ten years); death from another cause; or progression to the metastatic phase. The metastatic phase is defined as the time period of intensive treatment for metastatic disease, as determined for each cancer by SEER (Surveillance, Epidemiology, and End Results Program) averages. The terminal phase is defined as the one-month period prior to death. Each of these four sequelae has a separate disability weights, which are the same across cancers.

Data

Cancer incidence is directly estimated from cancer mortality using mortality to incidence ratios (MIRs). Cancer incidence was sought from individual cancer registries and from Cancer Incidence in Five Continents (CI5).[1-10] Data were excluded if they were not representative of the population (e.g., hospital-based registries).

Modelling strategy

Estimation of cancer mortality and MIR estimation has been described in detail elsewhere (Lancet 2020; 396: 1204–22). The final GBD cancer mortality estimates are transformed to incidence estimates by using MIRs (which are modeled separately). To summarize the MIR estimation process: incidence and mortality data from cancer registries were matched by cancer, age, sex, year, and location to generate M/I ratios. These MIR data were used to fit cause-specific fixed effect logistic regression models with covariates for sex, categorical age, and the HAQ index:

$$\text{logit}\left(MI\ ratio_{c,a,s,t}\right) = \alpha + \beta_1 HAQI_{c,t} + \sum_{a}^{A} \beta_2 I_a + \beta_3 I_s + \epsilon_{c,a,s,t}$$

c: country, a: age group, t: time (years); s: sex
HAQI: Healthcare Access and Quality Index
I: indicator variable
"ϵ" c,a,s,t: error term

These models were then used to obtain MIR estimates for all combinations of GBD age, sex, year, cause, and location. Data points were outliered manually if they clearly influenced the model in an unrealistic way. For example, a data point was marked as an outlier if it created a single-year, single age group spike in model predictions that was inconsistent with the trend suggested by surrounding data points. Results from the final linear model were used as input for space-time smoothing and a Gaussian Process Regression (ST-GPR).

Final MIR estimates at the 1,000-draw level were combined with final mortality estimates (also at the 1,000-draw level) to generate 1,000 draws of incidence estimates (which provides an estimated mean incidence with 95% uncertainty interval). It was assumed that uncertainty in the MIR is independent of uncertainty in the estimated mortality.

After transforming the final GBD cancer mortality estimates to incidence estimates (step 1 in the general cancer flowchart), incidence was combined with annual relative survival estimates from 1 to 10 years (step 7 in the flowchart). Using the survival estimation methods, MIRs generated the yearly cancer relative survival estimates utilizing age-specific curves. GBD used SEER*Stat to obtain mortality, incidence, and relative survival statistics from the 9 SEER registries reporting from 1980-2014 (step 2), by cancer type, sex, 5-year blocks (i.e., 1980-84, 1985-1989, etc.), and 5-year age groups (except combining 80+). For each cancer, GBD modelled 5-year relative survival with the SEER MIRs. These models were then applied to the GBD MIR estimates to predict an estimated 5-year survival for each age/sex/year/location (step 4). To prevent unrealistic values, predicted 5-year survival values were winsorized to be between 0% and 100% survival. To generate yearly survival estimates up to 10 years, SEER sex- and age-specific annual 1- through 10-year relative survival data were downloaded from patients diagnosed between 2001 and 2010. The proportion of the predicted GBD 5-year survival estimate to the SEER 5-year survival statistic was calculated as a scalar, and then used to generate yearly survival estimates by scaling the 1-10 year SEER curve to the GBD survival predictions under the proportional hazard assumption (step 5).

To transform relative to absolute survival (adjusting for background mortality), GBD 2019 lifetables were used (step 6 and 7 in the flowchart) to calculate lambda values: lambda= (ln(nLxn/nLxn+1))/5, where nLx=person years lived between ages x and x+n (from GBD lifetable). Absolute survival was then calculated using an exponential survival function (absolute survival = relative survival*elambda*t). Absolute survival is combined with incidence to estimate the prevalence at each year after diagnosis, which is then split into the four sequelae (step 8 in the flowchart).

For the purposes of calculating disability due to cancer, survivors beyond 10 years were considered cured. For the survivor population, prevalence was divided into two sequelae (1. diagnosis and primary therapy; 2. controlled phase). For the population that did not survive beyond 10 years, the yearly prevalence was divided into the four sequelae by assigning the fixed durations for each of the diagnosis and primary therapy phase, metastatic phase, and terminal phase, and assigning the remaining prevalence to the controlled phase (step 8 in the flowchart).

GBD assumed that for the population surviving up to 10 years, only the prevalence population being in remission experiences additional disability due to procedures. To estimate the prevalence of the cancer population in remission during the first 10 years after diagnosis with and without procedure-related disability, GBD multiplied the prevalence of the population in the remission phase with the proportion of the population undergoing a procedure. This step allowed to estimate disability during the remission phase for both the population experiencing disability due to the remission phase alone, as well as the population experiencing disability from the remission phase and the additional procedure-related disability.

Lastly, the procedure sequelae prevalence and general sequelae prevalence were multiplied with their respective disability weights to obtain the number of YLDs (steps 11 and 12 in the flowchart). The sum of these YLDs is the final YLD estimate associated with each cancer.

The following table provides the lay description and disability weights associated with brain and nervous system cancer.

Health state	Lay description	Disability weights (95% CI)
Cancer, diagnosis and primary therapy	This person has pain, nausea, fatigue, weight loss and high anxiety.	0.288 (0.193 - 0.399)
Cancer, controlled phase	This person has a chronic disease that requires medication every day and causes some worry but minimal interference with daily activities.	0.049 (0.031- 0.072)
Cancer, metastatic	This person has severe pain, extreme fatigue, weight loss and high anxiety.	0.451 (0.307 - 0.600)
Terminal phase, with medication	This person has lost a lot of weight and regularly uses strong medication to avoid constant pain. The person has no appetite, feels nauseous, and needs to spend most of the day in bed.	0.540 (0.377 - 0.687)

References

1. Doll R, Payne P, Waterhouse J. Cancer incidence in five continents I. Geneva: Union Internationale Contre le Cancer, 1966.
2. Doll R, Muir C, Waterhouse J. Cancer incidence in five continents II. Geneva: Union Internationale Contre le Cancer, Geneva, 1970.
3. Waterhouse J, Muir C, Correa P, Powell J. Cancer incidence in five continents III. Lyon: IARC, 1976.
4. Waterhouse J, Muir C, Shanmugaratnam K, Powell J. Cancer incidence in five continents IV. Lyon: IARC, 1982.
5. Muir C, Mack T, Powell J, Whelan S. Cancer incidence in five continents V. Lyon: IARC, 1987.
6. Parkin D, Muir C, Whelan S, Gao Y, Ferlay J, Powell J. Cancer incidence in five continents VI. Lyon: IARC, 1992.
7. Parkin D, Whelan S, Ferlay J, Raymond L, Young J. Cancer incidence in five continents VII. Lyon: IARC, 1997.
8. Parkin D, Whelan S, Ferlay J, Teppo L, Thomas D. Cancer incidence in five continents VIII. Lyon: IARC, 2002.
9. Curado M, Edwards B, Shin H, et al. Cancer incidence in five continents IX. Lyon: IARC, 2007.
10. Forman D, Bray F, Brewster D, et al. Cancer incidence in five continents X. 2013.

D6. Parkinson's disease

The steps in the estimation of non-fatal Parkinson's disease burden are shown in the following flowchart:

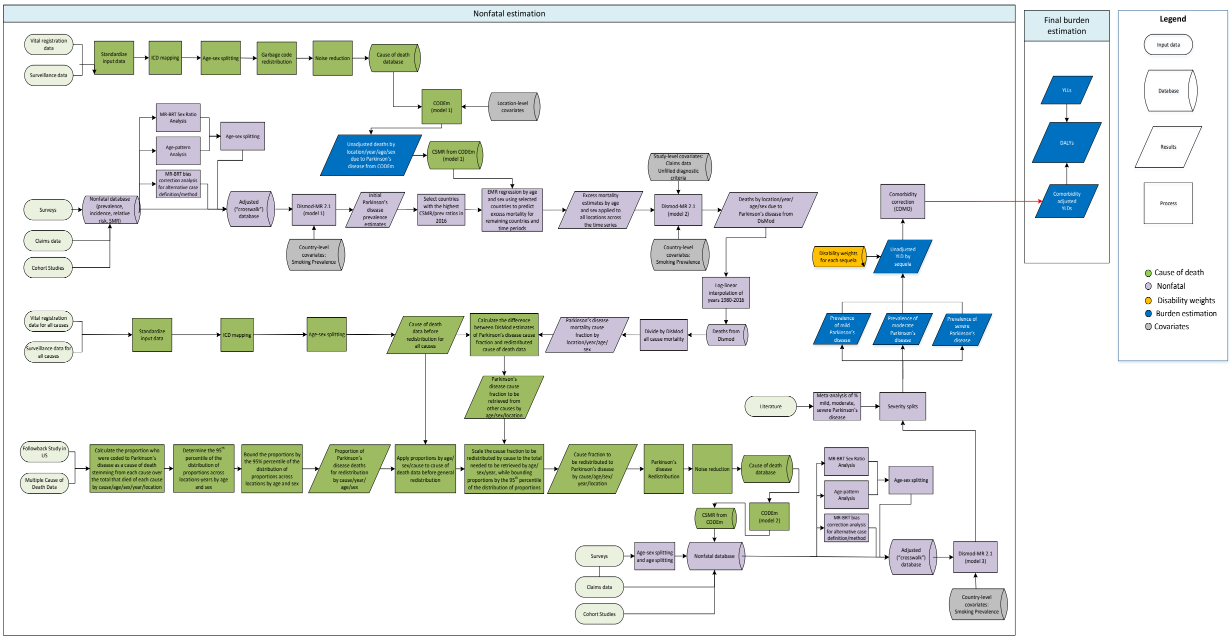

Data

To inform estimates of burden due to Parkinson's disease, GBD used mortality data from vital registration systems, as well as prevalence data from population-based representative surveys. Certain studies have been outliered on a case-by-case basis due to subsequent review and exclusion due to inappropriateness of the study design, or case ascertainment that conflict with existing gold-standard data, where possible.

Modelling strategy

Studies with age and sex detail separately were split into age- and sex-specific data points. Standard GBD sex splitting methods were used for studies with only "both" sex data points: GBD modelled the ratio of female/male prevalence in MR-BRT and then calculated male prevalence:

$$prev_{male} = prev_{both} * \frac{pop_{both}}{(pop_{male} + ratio * pop_{female})}$$

And then calculated female prevalence:

$$prev_{female} = ratio * prev_{male}$$

Summary of covariates used in the Parkinson's Disease DisMod-MR meta-regression model

Covariate	Type	Parameter	Exponentiated beta (95% Uncertainty interval)
Smoking prevalence (age-standardized)	Prevalence	-1.15	0.32 (0.28 – 0.36)
Healthcare access and quality index	Excess mortality rate	-0.025	0.98 (0.97 – 0.98)

Severity splits and disability weights

Hoehn and Yahr stages were used to determine severity. The cut-points were updated to more accurately correspond the lay descriptions of severities. Specifically, a Hoehn and Yahr stage 4 corresponds to a designation of severe, where before it was classified as moderate.

Severity	Stage
Mild	≤2.0
Moderate	2.5-3.5
Severe	≥4

The following table provides the lay description and disability weights associated with Parkinson's disease.

Severity level	Lay description	Disability weights (95% CI)
Mild	Has mild tremors and moves a little slowly, but is able to walk and do daily activities without assistance.	0.01 (0.005–0.019)
Moderate	Has moderate tremors and moves slowly, which causes some difficulty in walking and daily activities. The person has some trouble swallowing, talking, sleeping, and remembering things.	0.267 (0.181–0.372)
Severe	Has severe tremors and moves very slowly, which causes great difficulty in walking and daily activities. The person falls easily and has a lot of difficulty talking, swallowing, sleeping, and remembering things.	0.575 (0.396–0.73)

D7. Multiple sclerosis

The steps in the estimation of multiple sclerosis disease burden are shown in the following flowchart:

Multiple sclerosis

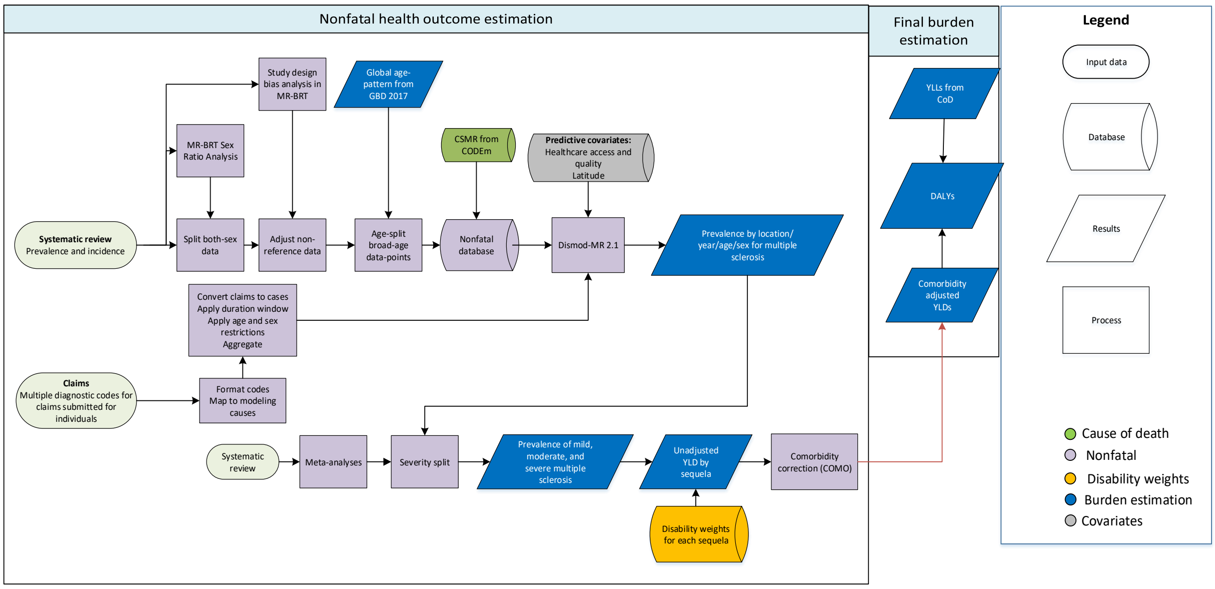

Data

The data underpinning estimates of burden due to multiple sclerosis are generally of two types. The first are representative, population-based, cross-sectional or longitudinal studies reported in peer-reviewed journals and identified via a search-string-based review, and described in previous reports. Estimates of epidemiologic measures (prevalence, incidence, etcetera) were manually extracted from these publications. The second type are claims data as obtained and processed by the GBD Clinical Informatics team.

For studies that reported epidemiologic measures (generally prevalence or incidence) by age for both sexes combined, and also by sex for all ages combined, GBD calculated the sex-ratio of cases in that study and applied it to the age-specific measures to estimate age-sex-specific measures.

To estimate sex-specific measures from studies that reported only for both sexes combined, GBD modeled the log sex ratio in MR-BRT using all sex-specific measurements from all other studies in the database and combined these with the GBD sex-specific population estimates for the relevant age-group. Estimate of prevalence were applied for calculating:

$$prev_{male} = prev_{both} * \frac{pop_{both}}{(pop_{male} + ratio * pop_{female})}$$

and then calculating female prevalence:

$$prev_{female} = ratio * prev_{male}$$

Also, equivalent equations were used for calculating incidence.

Modelling strategy

GBD used DisMod 2.1 as the main analytical tool for the multiple sclerosis estimation process. Inputs included prevalence and incidence data, as well as the cause-specific mortality rate (CSMR) estimated in the GBD causes of death analysis, and excess mortality rate (EMR) obtained by dividing CSMR by prevalence data-points. Prior settings included zero remission for all ages, no incidence or excess mortality for persons under 5 years old, and incidence limited to less than 0.000005 after the age of 60 years.

The table below illustrates the covariates, parameters, beta and exponentiated beta values for multiple sclerosis.

Covariate	Parameters	Beta coeff (95% CI)	Exponentiated
Absolute value of average latitude	Prevalence	0.041 (0.037 to 0.042)	1.04 (1.04 to 1.04)
Absolute value of average latitude	Incidence	0.041 (0.036 to 0.045)	1.04 (1.04 to 1.05)
Healthcare Access and Quality index	Excess mortality rate	-0.027 (-0.037 to -0.022)	0.97 (0.96 to 0.98)

Severity splits and disability weights

Kurtzke's Expanded Disability Status Scale (EDSS) was used to determine severity splits for multiple sclerosis. The EDSS scores corresponding to each severity are as follows:

- Asymptomatic: EDSS = 0
- Mild: 0 < EDSS ≤ 3.5
- Moderate: 3.5 < EDSS ≤ 6.5
- Severe: 6.5 < EDSS ≤ 9.5

The following table provides the lay description and disability weights associated with multiple sclerosis.

Severity level	Lay description	Disability weights (95% CI)
Asymptomatic	-	0 (0-0)
Mild	Has mild loss of feeling in one hand, is a little unsteady while walking, has slight loss of vision in one eye, and often needs to urinate urgently.	0.183 (0.124–0.253)
Moderate	Needs help walking, has difficulty with writing and arm coordination, has loss of vision in one eye and cannot control urinating.	0.463 (0.313–0.613)
Severe	Has slurred speech and difficulty swallowing. The person has weak arms and hands, very limited and stiff leg movement, has loss of vision in both eyes and cannot control urinating.	0.719 (0.534–0.858)

D8. Motor neuron diseases

The steps in the estimation of non-fatal motor neuron diseases burden are shown in the following flowchart:

Motor neuron diseases

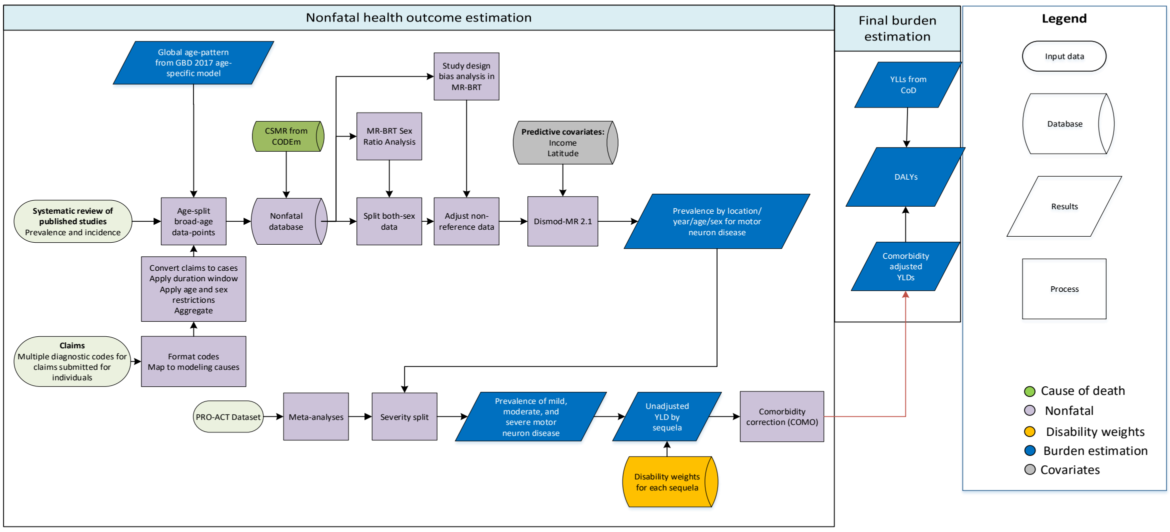

Data

Data were extracted from representative population-based study with well-defined sample, and reports on prevalence, incidence, remission, excess mortality, relative risk of mortality, standardised mortality ratio, or with-condition mortality rate for motor neuron diseases in aggregate or a specified motor neuron disease available from a systematic review.

All sex-specific data were used to estimate a pooled sex-ratio using MR-BRT. This ratio was combined with sex-specific population estimates for the year-age-location combinations corresponding to each data point reported for both sexes combined, to estimate sex-specific data-points prior to modeling. These were applied by calculating male prevalence:

$$prev_{male} = prev_{both} * \frac{pop_{both}}{(pop_{male} + ratio * pop_{female})}$$

and then calculating female prevalence:

$$prev_{female} = ratio * prev_{male}$$

Also, equivalent equations were used for calculating incidence.

Modelling strategy

GBD used DisMod 2.1 as the main analytical tool for motor neuron diseases estimation. Inputs included prevalence and incidence data, as well as the cause-specific mortality rate (CSMR) estimated in the GBD causes of death analysis, and excess mortality rate (EMR) obtained by dividing CSMR by prevalence data-points. Prior settings are limited to 0 remission at all ages and maximum incidence of 0.0004. GBD also constrain the super-region random effects for prevalence and incidence to -0.5 and 0.5 to account for spurious inflation of regional differences. The following covariates were employed to improve model predictions.

Covariate	Measure	Beta coefficient (95% CI)	Exponentiated
Absolute value of average latitude	Prevalence	0.032 (0.031 to 0.033)	1.03 (1.03 to 1.03)
LDI (I$ per capita)	Excess mortality rate	-0.5 (-0.5 to -0.5)	0.61 (0.61 to 0.61)

Severity splits and disability weights

The ALSFRS is an instrument for evaluating the functional status of patients with amyotrophic lateral sclerosis. It can be used to monitor functional changes in a patient over time. GBD subsequently mapped ALSFRS scores into GBD severities, and sequelae into different combinations of speech problems, chronic obstructive pulmonary disease, and motor impairment. After determining the severity status of each case for the three symptom umbrellas, GBD subsequently estimated the relative proportion of each combination of symptom class and their respective severities. Those without any symptoms (eg, no severity) were categorised as having worry about the diagnosis for disability estimation. The following table displays the various sequelae and their associated proportions.

**Sequela**	**Proportion (Mean)**	**Proportion (Lower)**	**Proportion (Upper)**
Mild motor impairment, mild respiratory problems and speech problems due to motor neuron disease	0.01779	0.01658	0.01909
Mild motor impairment, moderate respiratory problems and speech problems due to motor neuron disease	0.00270	0.00225	0.00324
Mild motor impairment, severe respiratory problems and speech problems due to motor neuron disease	0.00082	0.00059	0.00113
Mild motor impairment, and speech problems due to motor neuron disease	0.02052	0.01922	0.02190
Moderate motor impairment, mild respiratory problems and speech problems due to motor neuron disease	0.03377	0.03210	0.03552
Moderate motor impairment, moderate respiratory problems and speech problems due to motor neuron disease	0.00715	0.00640	0.00799
Moderate motor impairment, severe respiratory problems and speech problems due to motor neuron disease	0.00286	0.00240	0.00342
Moderate motor impairment, and speech problems due to motor neuron disease	0.03041	0.02883	0.03208
Severe motor impairment, mild respiratory problems and speech problems due to motor neuron disease	0.05242	0.05035	0.05457
Severe motor impairment, moderate respiratory problems and speech problems due to motor neuron disease	0.02247	0.02111	0.02392
Severe motor impairment, severe respiratory problems and speech problems due to motor neuron disease	0.01365	0.01259	0.01479
Severe motor impairment and speech problems due to motor neuron disease	0.04765	0.04567	0.04970
Mild respiratory problems and speech problems due to motor neuron disease	0.01157	0.01060	0.01263
Moderate respiratory problems and speech problems due to motor neuron disease	0.00142	0.00111	0.00182
Severe respiratory problems and speech problems due to motor neuron disease	0.00023	0.00013	0.00043
Speech problems due to motor neuron disease	0.02457	0.02315	0.02608
Mild motor impairment and mild respiratory problems due to motor neuron disease	0.02245	0.02109	0.02389
Mild motor impairment and moderate respiratory problems due to motor neuron disease	0.00275	0.00230	0.00329
Mild motor impairment and severe respiratory problems due to motor neuron disease	0.00068	0.00047	0.00097
Mild motor impairment due to motor neuron disease	0.10388	0.10103	0.10681
Moderate motor impairment and mild respiratory problems due to motor neuron disease	0.06744	0.06511	0.06985
Moderate motor impairment and moderate respiratory problems due to motor neuron disease	0.01302	0.01199	0.01413
Moderate motor impairment and severe respiratory problems due to motor neuron disease	0.00412	0.00356	0.00477
Moderate motor impairment due to motor neuron disease	0.20136	0.19760	0.20518
Severe motor impairment and mild respiratory problems due to motor neuron disease	0.06902	0.06666	0.07146
Severe motor impairment and moderate respiratory problems due to motor neuron disease	0.02000	0.01872	0.02137
Severe motor impairment and severe respiratory problems due to motor neuron disease	0.01062	0.00969	0.01163
Severe motor impairment due to motor neuron disease	0.15037	0.14702	0.15378
Mild respiratory problems due to motor neuron disease	0.00643	0.00571	0.00723
Moderate respiratory problems due to motor neuron disease	0.00044	0.00028	0.00069
Severe respiratory problems due to motor neuron disease	0.00005	0.00001	0.00017
Asymptomatic, but worry about diagnosis due to motor neuron disease	0.03738	0.03562	0.03921

To determine disability due to these sequelae, GBD used the standard multiplicative aggregation formula. The following table provides description and disability weights assigned to the sequelae as appropriate.

**Symptom group**	**Severity level**	**Lay description**	**Disability weights (95%)**
Respiratory problems	Asymptomatic		
Respiratory problems	Mild	Has cough and shortness of breath after heavy physical activity, but is able to walk long distances and climb stairs.	0.019 (0.011–0.033)
Respiratory problems	Moderate	Has cough, wheezing, and shortness of breath, even after light physical activity. The person feels tired and	0.225 (0.153–0.31)

		can walk only short distances or climb only a few stairs.	
Respiratory problems	Severe	Has cough, wheezing, and shortness of breath all the time. The person has great difficulty walking even short distances or climbing any stairs, feels tired when at rest, and is anxious.	0.408 (0.273–0.556)
Motor impairment	Asymptomatic		
Motor impairment	Mild	Has some difficulty in moving around but is able to walk without help.	0.01 (0.005–0.019)
Motor impairment	Moderate	Has some difficulty in moving around and difficulty in lifting and holding objects, dressing, and sitting upright, but is able to walk without help.	0.061 (0.04–0.089)
Motor impairment	Severe	Is unable to move around without help, and is not able to lift or hold objects, get dressed, or sit upright.	0.402 (0.268–0.545)
Speech problems	No		
Speech problems	Yes	Has difficulty speaking, and others find it difficult to understand.	0.051 (0.032–0.078)
Asymptomatic, but worry	Yes	Has a disease diagnosis that causes some worry but minimal interference with daily activities.	0.012 (0.006–0.023)

D10. Encephalitis

The steps in the estimation of non-fatal encephalitis burden are shown in the following flowchart:

Encephalitis

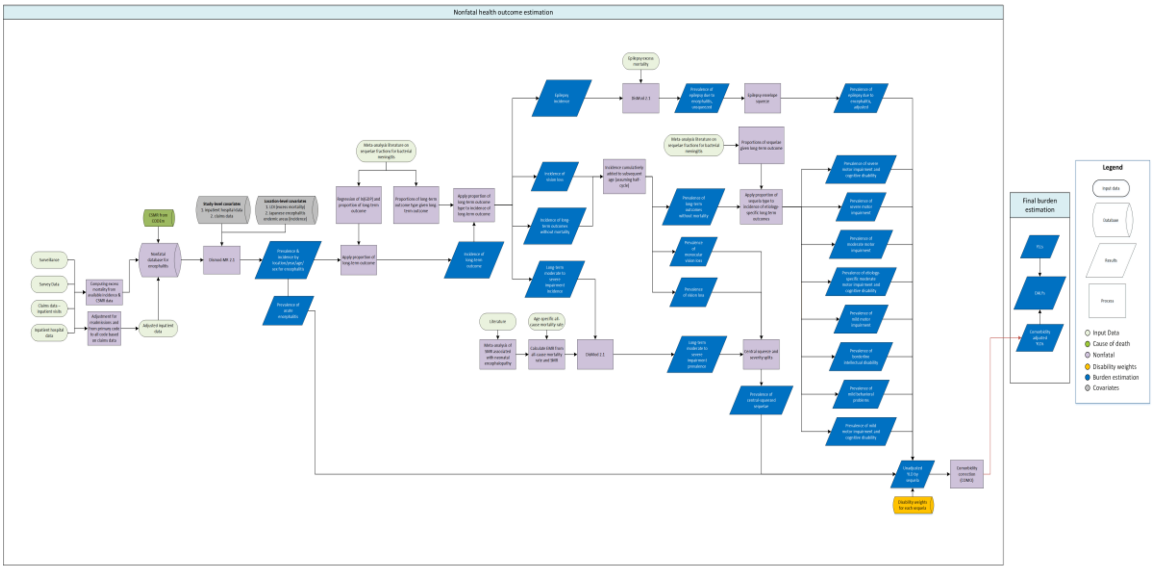

Data

Population representative surveys, studies and reports were included for the morbidity estimation of encephalitis.

Modelling strategy

Non-fatal outcomes were modelled using a combination of custom models and DisMod-MR 2.1. First, the overall incidence and prevalence of encephalitis were modelled to estimate the short-term morbidity due to acute infection. This DisMod model had a set duration (1/remission) of three weeks. Caps on excess mortality for ages 10-50 were imposed. The function in DisMod-MR 2.1 was used to pull in cause-specific mortality rate (CSMR) data from the CODEm and CODcorrect analyses and match with incidence data points for the same geography. The excess mortality rate was calculated to estimate priors by dividing CSMR by prevalence, calculated from remission and incidence. To help inform trends where data is lacking, a binary country-level covariate were applied at the subnational and country level. Incidence input data points were outliered with zero cases that were dragging down final estimates. In place of traditional DisMod-MR, Ordinary differential equations solver (ODE solver) was used to improve the efficiency of time and accuracy of estimations.

Country-level covariates, parameter, beta and exponentiated beta values for the model

Country-level covariate	Parameter	Beta	Exponentiated beta
Japanese Encephalitis endemic area	Incidence	0.052 (0.0041–0.097)	1.05 (1.00–1.10)
LDI (log transformed)	Excess mortality	-0.3 (-0.31 to -0.29)	0.74 (0.73–0.75)
DTP3 Coverage	Incidence	-1.56 (-1.68 to -1.45)	0.21 (0.19–0.23)

Sequelae splits

First the long-term sequelae were split among survivors of acute infection. The acute phase survivors were calculated by applying the excess mortality (calculated by the acute meningitis DisMod model) to the incidence of each aetiology (excess mortality was converted to case fatality rate by $e^{(-\text{excess mortality} \times 1/(\text{excess mortality} + \text{remission}))}$. The survivors were then subject to long-term sequelae by applying the post-discharge proportions of health consequences calculated by a meta-analysis by Edmond and colleagues.[1] The ratio of acute encephalitis survivors was calculated that result in a major long-term impairment, and the ratio of minor impairments to major impairments, based off a regression of log-transformed GDP and ratio values from Edmonds and colleagues.[1] The regression is shown below:

$$y = -0.33590 \ln(GDP) + 1.15230$$

A similar pattern of health outcomes was assumed for encephalitis infection survivors as with other bacterial meningitis survivors (except hearing loss, as no evidence was found for hearing loss as a consequence of encephalitis infection). These two ratios were used to calculate the proportions of survivors who contract a long-term minor impairment and those who contract a long-term major impairment. The proportion with major impairments were further split into specific major impairments, which were grouped into vision loss, moderate to severe cognitive impairments, and epilepsy.

The calculated incidence of long-term sequelae was then converted to prevalence by two different approaches. For the sequelae not associated with excess mortality, which were vision loss, intellectual disability, motor impairment, and behavioural problems, the incidence of each age was cumulatively added up to the subsequent age (assuming half-cycle) to construct prevalence at each age. If the sequela is associated with excess mortality (epilepsy and moderate-to-severe cognitive impairments), the calculated incidence was used as an input the ODE solver, together with the corresponding mortality parameters (excess mortality data from the epilepsy envelope DisMod model, and standardised mortality ratio data from a neonatal encephalopathy meta-analysis, converted to excess mortality using all-cause mortality estimates) to estimate the prevalence. Vision loss and epilepsy estimates were squeezed and severity split centrally.

Disability weights

The basis of the GBD disability weight survey assessments is lay descriptions of sequelae highlighting major functional consequences and symptoms. The lay descriptions and disability weights for sequelae associated with encephalitis are shown below.

Severity split	Lay description	Disability weights (95% CI)
Mild behavior problems	This person is hyperactive and has difficulty concentrating, remembering things, and completing tasks.	0.045 (0.028-0.066)
Moderate motor impairment	This person has some difficulty in moving around, and difficulty in lifting and holding objects, dressing and sitting upright, but is able to walk without help.	0.061 (0.04-0.089)
Moderate motor plus cognitive impairments	This person has some difficulty in moving around, holding objects, dressing and sitting upright, but can walk without help. This person has low intelligence and is slow in learning to speak and to do simple tasks.	0.203 (0.134-0.29)
Long-term mild motor impairment	This person has some difficulty in moving around but is able to walk without help.	0.01 (0.005-0.02)
Borderline intellectual disability	This person is slow in learning at school. As an adult, the person has some difficulty doing complex or unfamiliar tasks but otherwise functions independently.	0.011 (0.005-0.02)
Severe motor impairment	This person is unable to move around without help, and is not able to lift or hold objects, get dressed, or sit upright.	0.402 (0.268-0.545)
Epilepsy	(combined DW)	NA
Blindness	Is completely blind, which causes great difficulty in some daily activities, worry and anxiety, and great difficulty going outside the home without assistance.	0.187 (0.124-0.26)

Acute encephalitis	This person has a high fever and pain, and feels very weak, which causes great difficulty with daily activities.	0.133 (0.088-0.19)
Mild intellectual disability	This person has low intelligence and is slow in learning at school. As an adult, the person can live independently but often needs help to raise children<br>and can only work at simple supervised jobs.	0.043 (0.026-0.065)
Monocular distance vision loss	This person is blind in one eye and has difficulty judging distances.	0.017 (0.009-0.029)
Mild motor plus cognitive impairments	This person has some difficulty in moving around but is able to walk without help. The person is slow in learning at school. As an adult, the person has some difficulty doing complex or unfamiliar tasks but otherwise functions independently.	0.031 (0.018-0.05)
Severe motor plus cognitive impairments	This person cannot move around without help, and cannot lift or hold objects, get dressed or sit upright. The person also has very low intelligence, speaks few words, and needs constant supervision and help with all daily activities.	0.542 (0.37-0.702)

Reference

1. Edmond, K. et al. Global and regional risk of disabling sequelae from bacterial meningitis: a systematic review and meta-analysis. *Lancet Infectious Diseases* 2010; 10, 317–32.

D9. Meningitis

The steps in the estimation of non-fatal meningitis burden are shown in the following flowchart:

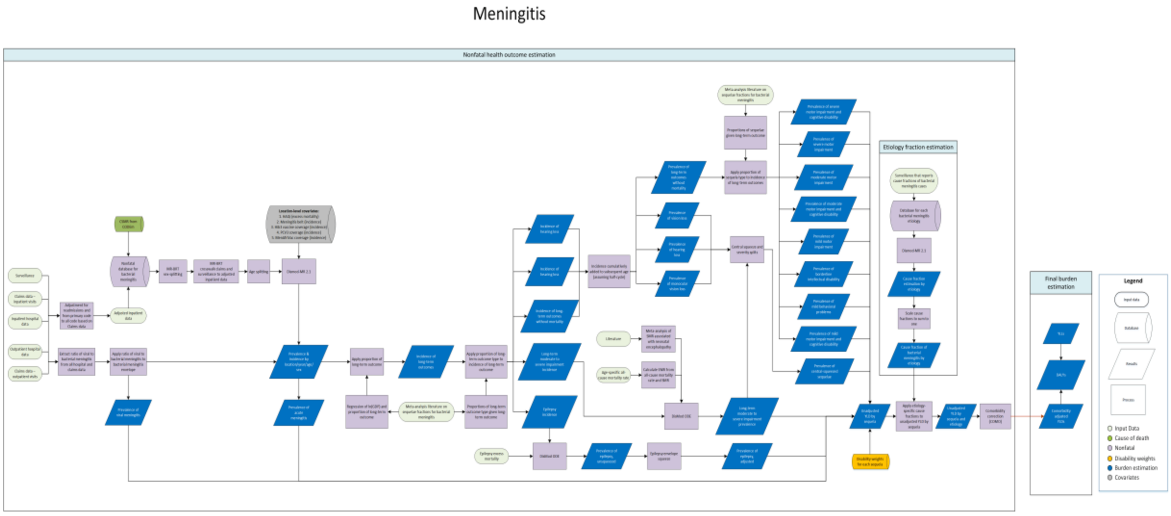

Data

Population representative surveys, studies and reports were included for the morbidity estimation of meningitis.

Modelling strategy

Non-fatal outcomes were modelled using a combination of custom models, DisMod-MR 2.1, and the use of an ordinary differential equations solver (ODE) for more timely and accurate estimates.

First, the overall incidence and prevalence of bacterial meningitis were modelled to estimate the short-term morbidity due to acute infection. This DisMod model had a set duration (1/remission) of four weeks with a range ±2 weeks. GBD also imposed caps on excess mortality for neonates and elders based on the highest excess mortality estimates from the previous GBD estimates. The function in DisMod- MR 2.1 was used to pull in cause-specific mortality rate (CSMR) data from the CODEm and CODcorrect analyses and match with prevalence data points for the same geography. Excess mortality rate was calculated to estimate priors by dividing CSMR by prevalence, calculated from remission and incidence. To help inform trends where data was lacking, a country-level covariate were applied for proportion of the population at the subnational and country levels. Incidence input data points were outlired with zero cases that were pulling down final estimates. Betas

and exponentiated values (which can be interpreted as an odd ratio) are shown in the tables below for study-level covariates and country-level covariates.

Country-level covariate	Parameter	Beta	Exponentiated beta
Hib3 vaccine coverage	Incidence	-0.61 (-0.66 to -0.57)	0.54 (0.52–0.57)
Meningitis belt	Incidence	1.93 (1.76–2.00)	6.88 (5.84–7.37)
MenAfriVac initiative	Incidence	-1.01 (-2 to -0.019)	0.36 (0.14–0.98)
LDI	Excess mortality	-0.00051 (-0.0013 to -0.000058)	1.00 (1.00–1.00)
Healthcare Access and Quality index	Excess mortality	-0.027 (-0.028 to - 0.027)	0.97 (0.97–0.97)

Incidence and prevalence of bacterial meningitis were split into four aetiologies (pneumococcal, meningococcal, H influenzae type B, and other bacterial meningitis) using four proportion models run in DisMod-MR 2.1. Results from these models were squeezed to sum to 1 at the draw level for each location, year, age, and sex. A Hib3 vaccine coverage covariate was applied to the H. influenzae type B proportion model, the proportion of the population living in the meningitis belt covariate and the proportion of the population living in areas covered by the MenAfriVac initiative (meningitis meningococcal type A) to the meningococcal meningitis proportion model, and a PCV3 coverage covariate to the pneumococcal meningitis model.

Data for viral meningitis was only available from hospital data, and not from population studies, so incidence and prevalence of viral meningitis were extrapolated from bacterial meningitis incidence by applying age- and sex-specific ratios between bacterial and viral cases from hospital data. In addition to short-term sequelae as a result of acute bacterial and viral meningitis, the long-term outcomes were modelled from bacterial meningitis infection.

Sequelae splits

GBD first split the long-term sequelae among survivors of acute infection. The acute-phase survivors was calculated by applying the excess mortality (calculated by the acute meningitis DisMod model) to the incidence of each aetiology (excess mortality was converted to case fatality rate by $e^{(\text{-excess mortality x 1/(excess mortality + remission)})}$). The survivors were then subject for long-term sequelae by applying the post-discharge proportions of health consequences calculated by a meta-analysis by Edmond and colleagues.[1] The ratio was calculated of acute meningitis survivors that experience major long-term impairments for all aetiologies, and the ratio of minor impairments to major impairments for pneumococcal meningitis versus all other aetiologies (because pneumococcal meningitis showed significantly higher risk of morbidity than other aetiologies). This ratio was based off a regression of log-transformed GDP and ratio values from Edmonds and colleagues.[1] The regression is shown below:

$$y = -0.33590 \ln(GDP) + 1.15230$$

These two ratios were used to calculate the proportions of survivors who contract a long-term minor impairment and those who contract a long-term major impairment. The proportion with major impairments were further split (again using pooled proportions from Edmond and colleagues[1]) into specific major impairments, which were grouped into vision loss, hearing loss, moderate-to-severe cognitive impairments, and epilepsy.

The calculated incidence of long-term sequelae was then converted to prevalence by two different approaches. For the sequelae not associated with excess mortality, which were vision loss, hearing loss, intellectual disability, motor impairment, and behavioural problems, the incidence of each age was cumulatively added up to the subsequent age (assuming half-cycle) to construct prevalence at each age. If the sequela is associated with excess mortality (epilepsy and moderate-to-severe cognitive impairments), the calculated incidence was used as in input to the ODE solver together with the corresponding mortality parameters (excess mortality data from the epilepsy envelope DisMod model, and standardised mortality ratio data from a neonatal encephalopathy meta-analysis, converted to excess mortality using all-cause mortality estimates) to estimate the prevalence. Vision loss, hearing loss, and epilepsy estimates were squeezed and severity split centrally.

Disability weights

The basis of the GBD disability weight survey assessments are lay descriptions of sequelae highlighting major functional consequences and symptoms. The lay descriptions and disability weights for sequelae associated with each aetiology are shown below.

Severity split	Lay description	Disability weights (95% CI)
Mild behaviour problems	This person is hyperactive and has difficulty concentrating, remembering things, and completing tasks.	0.045 (0.028-0.066)
Mild hearing loss	This person has great difficulty hearing and understanding another person talking in a noisy place (for example, on an urban street).	0.01 (0.004-0.019)
Mild hearing loss with ringing	This person is unable to hear and understand another person talking, even in a quiet place, is unable to take part in a phone conversation. Difficulties with communicating and relating to others cause emotional impact at times (for example worry or depression).	0.021 (0.012-0.036)
Moderate hearing loss	This person has great difficulty hearing and understanding another person talking in a noisy place (for example, on an urban street), and sometimes has annoying ringing in the ears.	0.027 (0.015-0.042)
Moderate hearing loss with ringing	This person is unable to hear and understand another person talking, even in a quiet place, is unable to take part in a phone conversation. Difficulties with communicating and relating to others often cause worry, depression, or loneliness.	0.074 (0.048-0.107)
Moderately severe hearing loss	Custom DW from hearing loss impairment envelope	
Severe hearing loss	This person is unable to hear and understand another person talking, even in a quiet place, and unable to take part in a phone conversation. Difficulties with communicating and relating to others cause emotional impact at times (for example worry or depression).	0.158 (0.105-0.227)
Profound hearing loss	This person is unable to hear and understand another person talking, even in a quiet place, is unable to take part in a phone conversation, and has great difficulty hearing anything in any other situation. Difficulties with communicating and relating to others often cause worry, depression, or loneliness.	0.204 (0.134-0.288)
Complete hearing loss	This person cannot hear at all in any situation, including even the loudest sounds, and cannot communicate verbally or use a phone. Difficulties with communicating and relating to others often cause worry, depression, or loneliness.	0.215 (0.144-0.307)
Severe hearing loss with ringing	This person is unable to hear and understand another person talking, even in a quiet place, is unable to take part in a phone conversation, and has annoying ringing in the ears for more than 5 minutes at a time, almost every day. Difficulties with communicating and relating to others cause emotional impact at times (for example worry or depression).	0.261 (0.175-0.36)
Profound hearing loss with ringing	This person is unable to hear and understand another person, even in a quiet place, is unable to take part in a phone conversation, has great difficulty hearing anything in any other situation, and has annoying ringing in the ears for more than 5 minutes at a time, several times a day. Difficulties with communicating and relating to others often cause worry, depression, or loneliness.	0.277 (0.182-0.387)
Complete hearing loss with ringing	This person cannot hear at all in any situation, including even the loudest sounds, and cannot communicate verbally or use a phone, and has very annoying ringing in the ears for more than half of the day. Difficulties with communicating and relating to others often cause worry, depression, or loneliness.	0.316 (0.212-0.435)
Moderate motor impairment	This person has some difficulty in moving around, and difficulty in lifting and holding objects, dressing and sitting upright, but is able to walk without help.	0.061 (0.04-0.089)
Moderate motor plus cognitive impairments	This person has some difficulty in moving around, holding objects, dressing and sitting upright, but can walk without help. This person has low intelligence and is slow in learning to speak and to do simple tasks.	0.203 (0.134-0.29)
Long-term mild motor impairment	This person has some difficulty in moving around but is able to walk without help.	0.01 (0.005-0.02)
Borderline intellectual disability	This person is slow in learning at school. As an adult, the person has some difficulty doing complex or unfamiliar tasks but otherwise functions independently.	0.011 (0.005-0.02)
Severe motor impairment	This person is unable to move around without help, and is not able to lift or hold objects, get dressed or sit upright.	0.402 (0.268-0.545)
Epilepsy	(combined DW)	NA
Blindness	Is completely blind, which causes great difficulty in some daily activities, worry and anxiety, and great difficulty going outside the home without assistance.	0.187 (0.124-0.26)
Severe acute episode of infectious disease	This person has a high fever and pain, and feels very weak, which causes great difficulty with daily activities.	0.133 (0.088-0.19)
Mild intellectual disability	This person has low intelligence and is slow in learning at school. As an adult, the person can live independently, but often needs help to raise children and can only work at simple supervised jobs.	0.043 (0.026-0.065)
Monocular distance vision loss	This person is blind in one eye and has difficulty judging distances.	0.017 (0.009-0.029)

Mild motor plus cognitive impairments	This person has some difficulty in moving around but is able to walk without help. The person is slow in learning at school. As an adult, the person has some difficulty doing complex or unfamiliar tasks but otherwise functions independently.	0.031 (0.018-0.05)
Severe motor plus cognitive impairments	This person cannot move around without help, and cannot lift or hold objects, get dressed or sit upright. The person also has very low intelligence, speaks few words, and needs constant supervision and help with all daily activities.	0.542 (0.37-0.702)

Reference

1. Edmond, K. et al. Global and regional risk of disabling sequelae from bacterial meningitis: a systematic review and meta-analysis. *Lancet Infectious Diseases* 2010; 10, 317–32.

D11. Tetanus

The steps in the estimation of non-fatal tetanus burden are shown in the following flowchart:

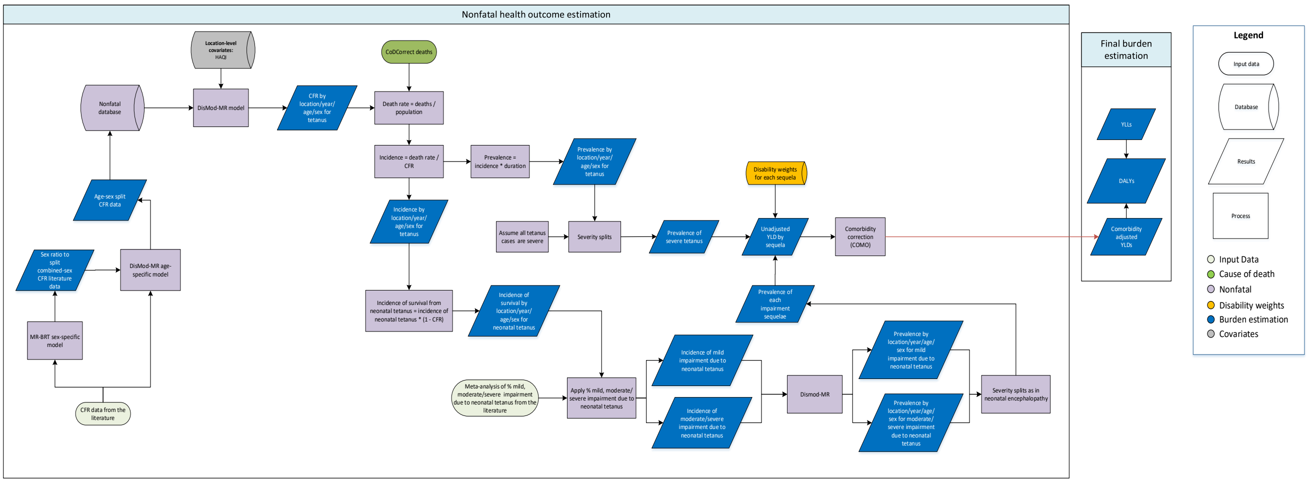

Data

The tetanus nonfatal model requires case fatality ratio (CFR) data obtained from systematic reviews of the literature, and the mortality rate outputs from the GBD 2019 tetanus mortality model.

All extracted tetanus CFR data that was not sex- and age-specific (i.e. the data that was reflective of both sexes combined and/or age ranges greater than a 20-year start and end difference) were split into sex and age-specific groups prior to use in modelling. The ratios used to make the sex splits were calculated using MR-BRT, the meta-regression Bayesian tool. The sex adjustment factor calculated for use in modeling was 0.96 (0.79 to 1.15).

Data input	Reference or alternative case definition	Beta Coefficient, Log (95% CI)	Adjustment factor*
Sex	N/A	-0.045 (-0.233 to 0.142)	0.96

*Adjustment factor is the transformed Beta coefficient in normal space, and can be interpreted as the factor by which the alternative case definition is adjusted to reflect what it would have been if measured as the reference.

Severity splits and disability weights

All of the tetanus cases estimated are assumed to be severe, acute infections. Table below presents our lay description of severe tetanus in addition to the disability weights applied. For neonatal tetanus impairments, the distribution matches the distribution of neonatal encephalopathy.

Severity level	Lay description	Disability weights (95% CI)
Severe	Has a high fever and pain, and feels very weak, which causes great difficulty with daily activities.	0.133 (0.088-0.19)

Modelling strategy

DisMod-MR was utilized to produce location-, year-, age-, and sex-specific tetanus CFR estimates from sex- and age-specific input data, following the age- and sex-splitting process. In the model, the Healthcare Access and Quality (HAQ) Index was used as a location-level covariate, enforcing a directional prior so location with increasing HAQ are predicted to have a reduced tetanus CFR. As a result, CFR model estimates now better reflect the expected relationship between HAQ and CFR across geographies and years, particularly in data-sparse locations. Additionally, DisMod model parameters were adjusted to decrease the influence of hierarchical priors in the DisMod geographic cascade. These adjustments allow the model to more closely track CFR data in locations where data is present and tend to result in broader uncertainty in CFR estimates for locations where no data is available.

Incidence rates were then calculated using estimates of tetanus CFR and GBD 2019 tetanus mortality estimates. Tetanus mortality rates were produced using CODEm separately for all combinations of children under one year of age and those ages one to eighty, data-rich and non-data-rich countries, and for males and females. Using these results, incidence was calculated as the quotient of mortality rate by CFR. From tetanus incidence and tetanus case duration sourced from a prior literature review, tetanus prevalence was computed. These calculations were completed at the draw level for each of 1000 draws, then summarized using the mean of draws and a 95% uncertainty interval (the 2.5th and 97.5th quantile of all draws).

Summary of covariates used in the tetanus CFR DisMod-MR meta-regression model

Covariate	Type	Parameter	Exponentiated beta (95% CI)
Healthcare Access and Quality Index	Country-level	Case fatality ratio	0.85 (0.75 — 0.97)

To estimate mild and moderate impairment due to neonatal tetanus, the incidence of survival was first computed:

$$Incidence\ of\ survival = incidence\ (1 * CRF)$$

To appropriately proportion impairments as either mild or moderate-to-severe, a systematic literature review of the proportion in cases was leveraged. These splits were applied to the incidence of survival to calculate the incidence of survival from neonatal tetanus with mild impairment and with moderate-to-severe impairment. These estimates were each then used as input data sets for separate DisMod-MR models, which in turn produced draw-level estimates of the prevalence of mild or moderate-to-severe impairment due to neonatal tetanus for all ages, sexes, years, and locations.

D12. Traumatic brain injury and spinal cord injury

The steps in the estimation of non-fatal injuries burden are shown in the following flowchart:

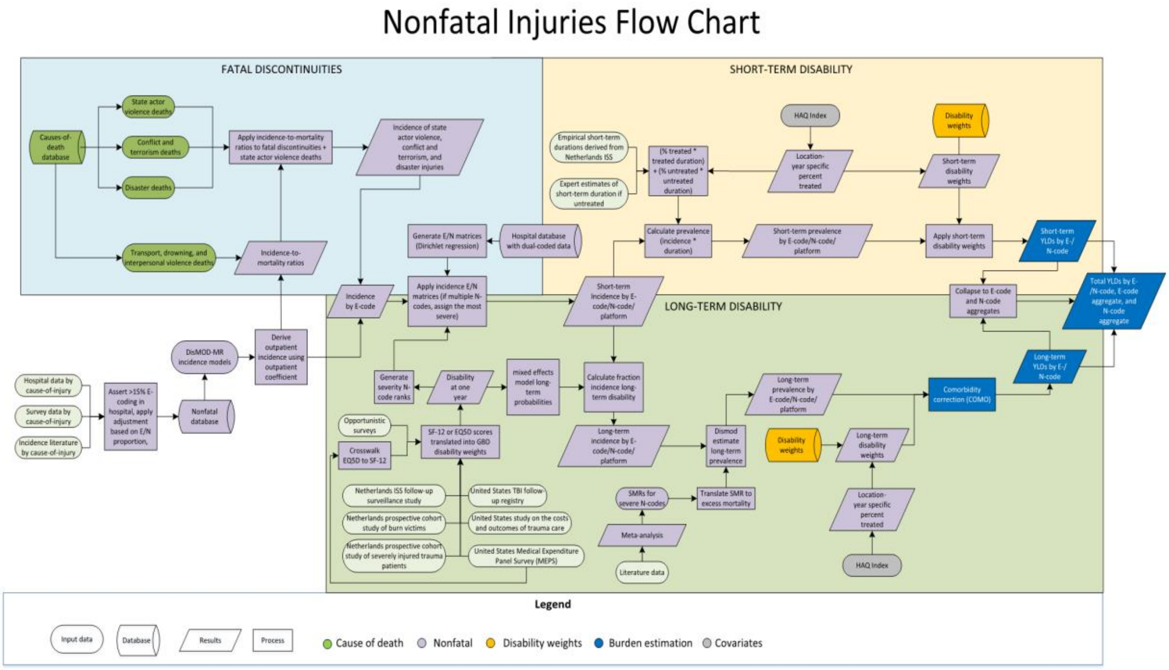

Data

Representative population-based surveys and studies reports incidence of injuries were included in the analysis. Consistent with modelling strategies for other causes in the GBD framework, injuries estimation also utilised hospital and clinic diagnosis codes for locations where these data were available.

Modelling strategy

Each external cause of injury is modelled in DisMod-MR 2.1. Some details on the modelling strategy are provided below, and more information on the overall injuries modelling process including injury-specific details is described in the methods appendix of the GBD 2019 publication on non-fatal outcomes (Lancet 2020; 396: 1204–22).

Cause-of-injury incidence

The majority of incidence data exist at the external cause-of-injury level. Incidence for cause-of-injury categories was modelled using DisMod-MR 2.1. Multiple datasets from hospital and emergency/outpatient departments, insurance claims, and surveys were fed into these incidence models. GBD separately estimated two categories of injury severity: inpatient and outpatient injuries.

Excess mortality modelling

Priors on excess mortality rate (EMR) were estimated in DisMod by matching prevalence data points with their corresponding CSMR values within the same age, sex, year, location (by dividing CSMR by prevalence). For short duration conditions like injuries (remission > 1), the corresponding prevalence was derived by running an initial model and then applying the same CSMR/prevalence method. However, for many causes, DisMod estimated a rather unrealistic pattern of EMR compared to an expected pattern of decreasing EMR with greater access to quality health care. Such unexpected patterns often signal inconsistencies between CSMR estimates and the measures of prevalence and/or incidence.

In effort to provide greater guidance to DisMod on the expected pattern of EMR, EMR data generated in the previous round were modeled using the MR-BRT approach by age and sex with a prior on Healthcare Access

and Quality (HAQ) Index having a negative coefficient. Results from MR-BRT were then predicted for each location year, sex and for ages 0, 10, 20 … 100. GBD included HAQ index as a country-level covariate to inform EMR with a mean and standard deviation produced from MR-BRT. However, even without this setting DisMod would tend to estimate a coefficient that was consistent with the MR-BRT analysis.

Adjusting data

The adjustment of data via study-level covariates was performed out of DisMod using adjustment coefficients derived from a network analysis on World Health Survey data on road injuries. First, ST-GPR was used to estimate the proportion of people who were able to receive care for their injuries using the ratio of invividuals who received inpatient or outpatient care to individuals who were injured overall. These proportions allowed us to adjust data to the definition "injuries that received inpatient or outpatient care." Then, MR-BRT was used to crosswalk "received care" incidence and outpatient incidence both to inpatient incidence, using inpatient versus outpatient incidence comparisons from the United States National Hospital Ambulatory Medical Care Survey.

Fatal discontinuities

Due to the sporadic nature of the incidence of injuries and a lack of time trend that results from fatal discontinuities, DisMod-MR 2.1 was not used to model incidence due to fatal discontinuities, including state actor violence, exposure to forces of nature (i.e., natural disaster), and conflict and terrorism. Instead, incidence-to-mortality ratios were averaged over super-region, year, and sex to limit the variability in the ratios applied to fatal discontinuities.

Nature-of-injury category hierarchy

Multiple injuries can occur in one individual. A nature-of-injuries severity hierarchy was developed to establish a one-to-one relationship between cause-of-injury and nature-of-injury category. This means that in the case of multiple injuries the nature-of-injury category that was likely to be responsible for the largest burden was selected. To construct the hierarchy, GBD used data from the pooled dataset of follow-up studies.[1–6] The output of the regression of logit-transformed disability weights on nature-of-injury category and individual characteristics of the follow-up studies were used to calculate the mean long-term disability attributable to each nature-of-injury category. The ranking of nature-of-injury categories by their long-term disability weights formed the basis of our severity hierarchy. Hierarchies were developed separately, for injuries warranting inpatient care and injuries warranting other health care.

Cause-nature matrices

Because injury disability is linked more to the nature of injury than to the cause of injury, matrices were generated to map the proportion of each cause-of-injury category that results in a particular nature-of-injury category. These matrices are based on a collection of dual-coded (i.e., both cause-of-injury and nature-of-injury coded) hospital and emergency department datasets. GBD applied our nature-of-injury severity hierarchy above to assert that every observation had one cause of injury and one nature of injury.

Dirichlet models were used to estimate all of the nature-of-injury category proportions for one cause of injury simultaneously. These models allow for consistent borrowing of information across age, sex, inpatient/outpatient, and high/low-income countries and assert that the nature-of-injury proportions within a cause-of-injury category must add up to 1. One cause-nature matrix was created for each combination of injury warranting hospital admission versus injury warranting other health care, high/low-income countries, male/female, and age category. Applying these matrices to our cause-of-injury incidence from DisMod-MR, cases of injury warranting hospital admission and incidence of injury warranting other health care by cause and nature of injury were produced.

Probability of permanent health loss

Disability due to injury was assumed to affect all cases in the short term with a proportion having long-term (permanent) outcomes. The probability of long-term outcomes was needed to estimate the incidence and subsequently the prevalence of cases with permanent health loss. In the conceptual model, individuals who suffer a non-fatal injury will, in the long-term, return to either full or partial health. If one-year post-injury patients return to a health status with more disability than their pre-injury health status, patients are assumed

to have permanent disability from their injury. The difference between the pre-injury health states and health status one year after injury is assumed to be their permanent level of injury-related disability. GBD assessed the probability of developing permanent health loss using the pooled dataset of follow-up studies[1–6] and the Medical Expenditure Panel Survey[7] that were also used to generate the nature-of-injury hierarchy. To assess the probability of permanent health loss, GBD estimated the effects using a logit-linear mixed effects regression:

$$Logit(DW)_{im} = \alpha + \beta(injuries_{im}) + \beta(never\ injured_i) + \beta(never\ injured_i * age_i) + \beta(fracture\ of\ pelvis_i) + \beta(fracture\ of\ pelvis_i * age_i) + \beta(poisoning_i * age_i) + \beta(moderate\ to\ severe\ TBI_i * age_i) + RE_c + RE_i$$

where dummies were included for all the nature-of-injury categories $(injuries_{im})$, with the reference category being no injury (from MEPS dataset). GBD also included a dummy for never injured prior to the current injury, age, interactions between age and never injured status, and interactions with long-term nature-of-injury categories (moderate/severe traumatic brain injuries) that were found to significantly vary with age. In notation, subscript m refers to patient-reported outcome measure, i refers to individual, and c refers to country. Random effects (RE) were included to control for variation between countries and individuals.

After predicting overall disability at one-year follow-up, we estimated a counterfactual by setting all observations to "no injury," the reference group for $\beta(injuries_{im})$ in the model. The disability attributable to the nature of injury at one year was assumed to be the difference between our counterfactual of no injury and predicted disability with injury. The probability of treated long-term outcomes was estimated via the ratio of this attributable disability relative to the long-term disability weight for that injury.

$$Probability\ of\ long - term\ disability = \frac{with\ injury\ disability_{im} - counterfactual\ disability_{im}}{DW_m}$$

GBD developed estimates of the probability of permanent health loss by nature-of-injury category, injury severity level (injuries warranting inpatient admission and injuries warranting other health care), and age. Moderate-severe TBI and spinal cord lesions only have inpatient injury long-term probabilities.

Disability associated with treated and untreated cases

For many nature-of-injury categories, GBD has a separate disability weight for treated and for untreated cases. To estimate the percent treated for injuries in a given location-year, the HAQ Index was used with the same strategy described for the probability of permanent health loss.

Duration of short-term health loss

To determine the duration for treated cases of short-term injury, we analysed patient responses from two Dutch Injury Surveillance System follow-up studies conducted from 2001–2003 and 2007–2009.[8] These studies collected data at 2.5, 5, 9, and 24 months post-injury to determine whether injury patients were still experiencing problems due to their injury. If not, the patients were asked how many days they had experienced problems. The injury patients that still reported having problems one year after the injury were assumed to be captured in our analysis of permanent disability. The duration for treated cases of short-term injury was estimated for injuries warranting inpatient admission and injuries warranting other health care separately. The estimates were supplemented by expert-driven estimates of short-term duration for nature-of-injury categories that did not appear in the Dutch dataset and untreated injuries.

Calculation of prevalence from incidence data – short-term injury

For short-term injury outcomes, which were assumed to be less than one year in duration, the prevalence for each cause-of-injury/nature-of-injury/severity-level grouping was approximated by the incidence for that grouping multiplied by the associated nature-of-injury/severity-level-specific duration.

Calculation of prevalence from incidence data – permanent health loss

For permanent health loss, GBD assumed no remission and thus integrated incidence over time to arrive at prevalence estimates. DisMod ODE (i.e., the "engine" of DisMod-MR 2.1) was used to carry out this integration for each combination of cause of injury and nature of injury by country, year, and sex. For this step we used random effects meta-analysis to pool data on standardised mortality ratios derived from literature reviews.

References

1. Chinese Center for Disease Control and Prevention (CCDC). China Zhuhai Study 2006-2007 - China CDC.
2. Consumer Safety Institute (Netherlands). Netherlands Injury Surveillance System 2002.
3. Consumer Safety Institute (Netherlands). Netherlands Injury Surveillance System 2008.
4. Mackenzie EJ, Rivara FP, Jurkovich GJ, et al. The National Study on Costs and Outcomes of Trauma. *J Trauma* 2007; 63: S54-67; discussion S81-86.
5. CDC, Medical University of South Carolina, South Carolina Department of Disabilities and Special Needs, South Carolina Department of Health and Environmental Control. South Carolina Traumatic Brain Injury Follow-up Registry 1999-2013. USA.
6. Van Loey NE, van Beeck EF, Faber BW, van de Schoot R, Bremer M. Health-Related Quality of Life After Burns: A Prospective Multicentre Cohort Study With 18 Months Follow-Up. *J Trauma* 2011; 72: 513-520.
7. Agency for Healthcare Research and Quality. United States Medical Expenditure Panel Survey. Rockville, United States: Agency for Healthcare Research and Quality.
8. Polinder S, van Beeck EF, Essink-Bot ML, Toet H, Looman CW, Mulder S, Meerding WJ. Functional outcome at 2.5, 5, 9, and 24 months after injury in the Netherlands. *J Trauma* 2007; 62: 133-41.

D13. Other non-communicable neurological disorders

In addition to the non-communicable neurological disorders described above, there are many diverse types of non-communicable neurological disorders with a range of severities and associated sequelae. Because these non-communicable neurological disorders are diverse in their underlying causes and risk factors as well as in their associated health outcomes, modelling them together in a DisMod-MR model would not produce reliable estimates of prevalence or excess mortality. Instead, GBD calculated the YLDs caused by neurological disorders directly using a YLD/YLL ratio.

GBD calculated the ratio of YLDs to YLLs across the specified neurological disorders for which non-fatal outcomes were modelled, using YLL estimates from the GBD 2019 cause of death (CoD) analysis. This YLD/YLL ratio then multiplied by the YLL estimates for other non-communicable neurological disorders from the GBD 2019 CoD analysis, providing with an estimate of the YLDs associated with other non-communicable neurological disorders.

E. Estimation of deaths from neurological disorders

The major data inputs used in GBD for estimating deaths from neurological disorders in India were Sample Registration System cause of death data, Medical Certification of Cause of Death data, and other verbal autopsy studies.

Deaths for most neurological disorders were generated using the cause of death ensemble modelling (CODEm). CODEm is the framework used to model most cause-specific death rates in the GBD. It relies on four key components. First, all available data are identified and gathered to be used in the modelling process. Though the data may vary in quality, they all contain some signal of the true epidemiological process. Second, a diverse set of plausible models are developed to capture well-documented associations in the estimates. Using a wide variety of individual models to create an ensemble predictive model has been shown to outperform techniques using only a single model both in cause of death estimation and in more general prediction applications. Third, the out-of-sample predictive validity is assessed for all individual models, which are then ranked for use in the ensemble modelling stage. Finally, differently weighted combinations of individual models are evaluated to select the ensemble model with the highest out-of-sample predictive validity.

As many factors covary with a particular cause of death, a large range of plausible statistical models are developed for each neurological disorder. For the CODEm framework, four families of statistical models are developed using covariates. These are mixed effects linear models of the natural log of the death rate, mixed effects linear models of the logit of the cause fraction, spatiotemporal Gaussian process regression (ST-GPR) models of the log of the death rate, and ST-GPR of the logit of the cause fraction. All plausible relationships between covariates and relevant cause are identified, and all possible permutations of selected covariates are tested in linear models where the logit cause fraction or log death rate is the response variable. Because all permutations of covariates, multicollinearity are tested between covariates may produce implausible signs on coefficients or unstable coefficients. All models where the sign on the coefficient is in the direction expected based on the literature and where the coefficient is statistically significant at $p < 0.05$ are retained. For both cause fractions and death rates covariate selection is performed and then create both mixed effects only and ST models for each set of covariates. Sum of the sampling variance, non-sampling variance, and garbage code redistribution variance for each data point are included in CODEm.

The performance of all component models and ensembles is evaluated using out-of-sample predictive validity tests. Thirty percent of the data are excluded from the initial model fits, and half of that (15% of total) is used to evaluate and rank component models and then build ensembles. Data are held out from the analysis using the pattern of missingness for each cause in the cause of death database. Out-of-sample predictive validity testing is repeated until stable model results have been obtained. The out-of-sample performance tests include the root mean squared error of the log of the cause-specific death rate, the direction of the trend in the prediction compared to the data, and the validity of the 95% uncertainty interval. For every model, the in-sample root mean squared error of the log death rates (RMSE) and the out-of-sample performance in the 15% of data not used in the model building process. After component models are ranked on their out-of-sample predictive validity they are weighted based on their ranking and each component model contributes a portion to the final estimate. How much each sub model contributes is a function of its relative ranking as well as the value of psi chosen, which dictates that distribution of rankings.

Using the second half of the holdout data (15% of total), the differently weighted ensembles and different values of psi are tested using the same predictive validity metrics as the component models. For every model, the in-sample RMSE and the out-of-sample performance in the 15% of data not used in the model building process. The ensemble with the best average trend and RMSE is chosen as the final ensemble weighting scheme.

After a model weighting scheme has been chosen, each model contributes a number of draws proportional to its weight such that 1,000 draws are created. The mean of the draws is used as the final estimate for the CODEm process and 95% UI are created from the 0.025 and 0.975 quantiles of the draws. The final assessment of ensemble model performance is the validity of the UIs; ideally, the 95% UI for a model would capture 95% of the data out-of-sample. Higher coverage suggests that UIs are too large and lower than 95% suggest UIs are too narrow.

For certain causes of death, such as Alzheimer's diseases and other dementias and Parkinson's disease, mortality rates reported in vital registration systems are impossible to reconcile with the observed trends in disease prevalence and excess mortality. To address this bias in cause of death (CoD) data, customised modelling approaches are used to first identify the proportion of all deaths that should be assigned to these causes and then determine the GBD causes and garbage groups to which these deaths are being incorrectly

assigned. For Parkinson's disease, excess mortality is first estimated from prevalence and CoD data in countries with the highest ratio of cause-specific mortality to prevalence. Then, using DisMod-MR 2, cause-specific mortality rates estimated from available prevalence surveys, as well as the estimates of excess mortality rate, are applied across countries and over time. This value is divided by the all-cause mortality rate to determine the fraction of overall mortality to attribute to each under-coded cause. For Alzheimer's diseases and other dementias, relative risk data from cohort studies were used to calculate total number of excess deaths due to dementia, and end-stage disease proportions from linked hospital to death records to subset these deaths to the proportion of excess deaths with end-stage conditions, which are attributed to dementia.

The detailed description for estimating deaths for each neurological disorder presented in this paper are described below.

E1. Stroke

The approach for estimating deaths from stroke is shown in the following flowchart:

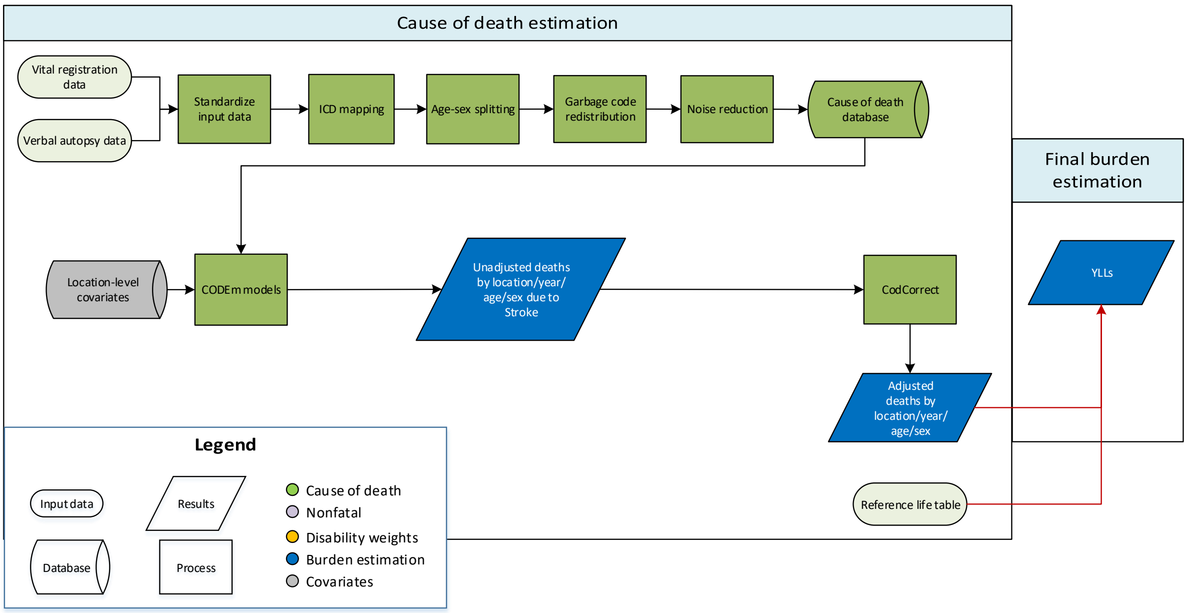

Data

Verbal autopsy and vital registration data were used to model cerebrovascular disease (stroke). GBD reassigned deaths from verbal autopsy reports for cerebrovascular disease to the parent cardiovascular disease for both sexes for those under 20 years of age. GBD outliered non-representative subnational verbal autopsy data points. GBD also outliered ICD8, ICD9BTL, and tabulated ICD10 data points which were inconsistent with the rest of the data and created implausible time trends. Data points from sources which were implausibly low in all age groups and data points that were causing the regional estimates to be improbably high were outliered.

Modelling strategy

A standard CODEm approach was used to model deaths from stroke. The covariates included in the ensemble modelling process are listed in the table below.

Covariate	Transformation	Level	Direction
Summary exposure variable, stroke	None	1	1
Cholesterol (total, mean per capita)	None	1	1
Smoking prevalence	None	1	1
Systolic blood pressure (mmHg)	None	1	1
Mean BMI	None	2	1
Elevation over 1,500 m (proportion)	None	2	-1
Fasting plasma glucose	None	2	1
Outdoor pollution ($PM_{2.5}$)	None	2	1
Indoor air pollution	None	2	1
Healthcare Access and Quality Index	None	2	-1
Lag distributed income per capita (I$)	Log	3	-1

Summary exposure value, omega-3	None	3	1
Summary exposure value, fruits	None	3	1
Summary exposure value, vegetables	None	3	1
Summary exposure value, nuts and seeds	None	3	1
Pulses/legumes (kcal/capita, unadjusted)	None	3	-1
Summary exposure value, PUFA adjusted (percent)	None	3	1
Alcohol (litres per capita)	None	3	1
Trans fatty acid	None	3	1

E2. Epilepsy

The approach for estimating deaths from idiopathic epilepsy is shown in the following flowchart:

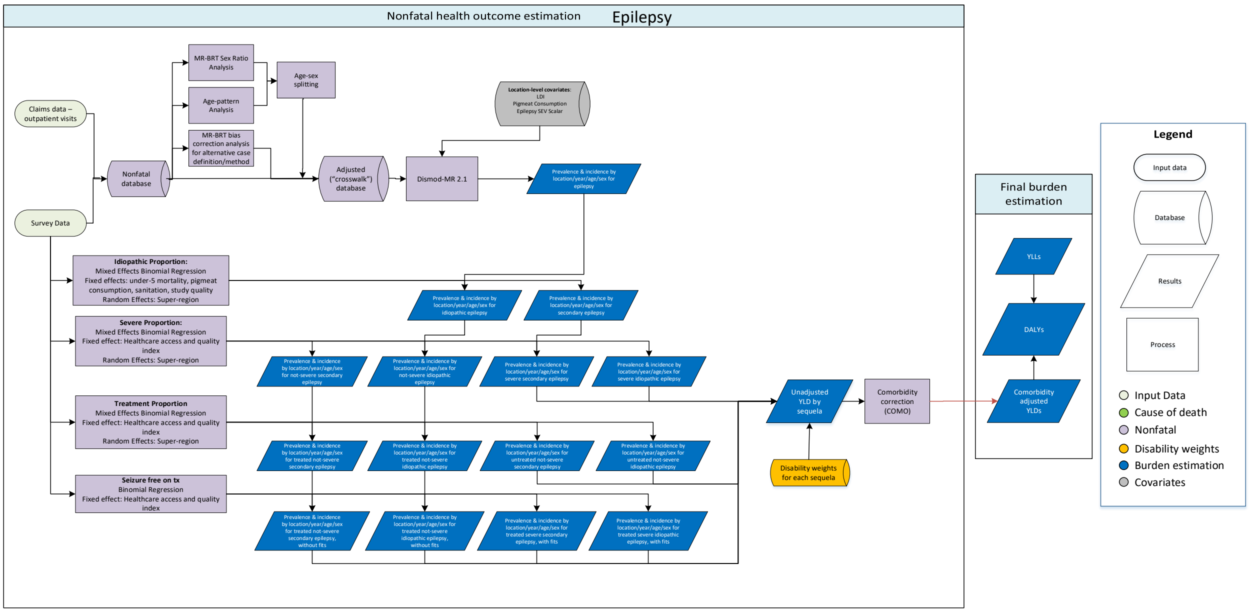

Data

For estimating deaths from epilepsy vital registration and verbal autopsy data were included. Data points were excluded if they were implausibly high or low relative to global or regional patterns, substantially inconsistent with established age or temporal patterns, or significantly inconsistent with other data sources from the same locations or locations with similar characteristics (ie, Socio-demographic index).

Modelling strategy

The standard CODEm modelling approach was applied to estimate deaths due to epilepsy. Separate models were used for estimating deaths for male and female, and the age range for both models was 28 days to 95+ years. Unadjusted death estimates were adjusted using CoDCorrect to produce final estimates of YLLs.

Table: Covariates used in modelling deaths from epilepsy

Level	Covariate	Direction
1	Pig meat consumption (kcal per capita)	+
	Pigs (per capita)	+
	SEV scalar: epilepsy	+
	Mean systolic blood pressure (mmhg)	+
2	Healthcare access and quality index	-
	Mean body-mass index	+
	Mean serum total cholesterol (mmol/L)	+
3	Cumulative cigarettes (10 years)	+
	Cumulative cigarettes (5 years)	+
	education (years per capita)	-
	log LDI (per capita)	-
	Socio-demographic Index	-

E3. Alzheimer's disease and other dementias

The approach for estimating deaths from Alzheimer's disease & other dementias are shown in the following flowchart:

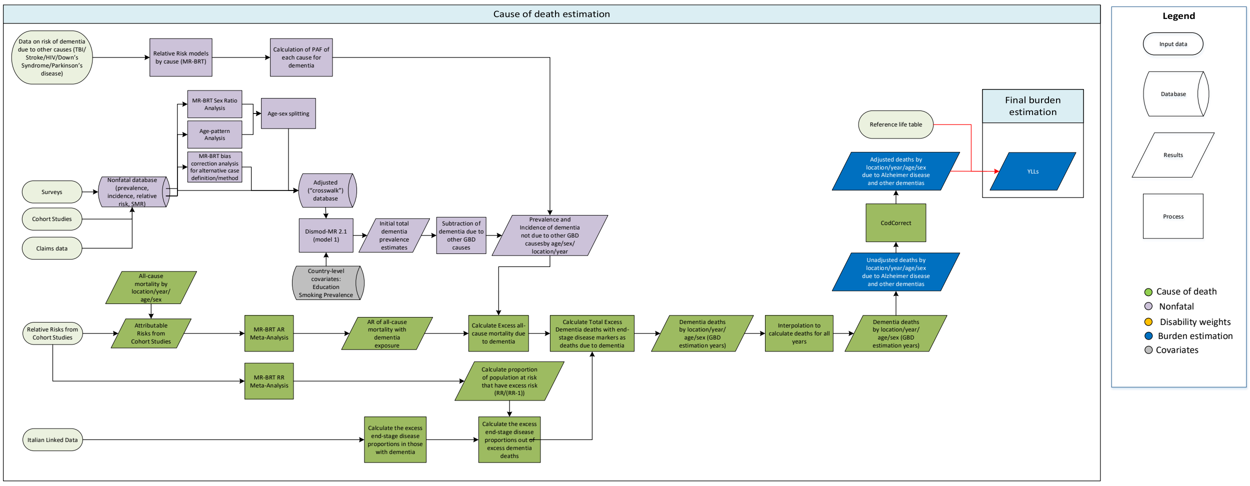

Data

For estimating deaths due to Alzheimer's disease & other dementias, death data from vital registration systems and prevalence data from population-based studies were used. Studies that reported prevalence, incidence, remission rate, excess mortality rate, relative risk of mortality, standardised mortality ratio, or with condition mortality rate were included. Studies with non-representative samples or no clearly defined sample were excluded from the analysis.

Modelling Strategy

Relative risk data

First, using relative risk data extracted from studies identified by systematic review, the attributable risk was calculated and the GBD estimate of all-cause mortality rate for a given study location and time, using the following formula:

$$Attributable\ Risk = (Relative\ Risk - 1) * All\text{-}Cause\ Mortality$$

Meta-analysis was conducted on the attributable risk data, using covariates for age, sex, exposure category (all dementia, Alzheimer's disease, cognitive impairment), whether the study was conducted in a clinical sample, and categories indicating different types of variables that were controlled for in the component studies (educational attainment, cardiovascular disease comorbidities, smoking and alcohol consumption, and daily activities or residence in a nursing home). Relative risks were estimated using a second Bayesian bias-reduction meta-regression model and the same studies identified through systematic review.

Meta-regression results were used to calculate the total number of excess deaths due to dementia as the product of prevalence estimates (post-adjustment for dementia caused by other GBD diseases) and estimates of attributable risk.

Linked data

The excess deaths calculated through the multiplication of attributable risk and prevalence represent the total number of excess deaths due to having dementia, which likely includes deaths due to other conditions, such as cardiovascular diseases, that are more common in those with dementia as compared to the general population due to common underlying risk factors such as blood pressure, smoking, and lower educational attainment. In order to subset this total number of excess dementia deaths to calculate the number of deaths that were caused by dementia, GBD completed an analysis of linked clinical and mortality data. Death records linked to inpatient records were used, covering all deaths from 2003 to 2017 in the Emilia-Romagna region of Italy. Using these data, GBD looked for markers of severe, end-stage disease in the clinical records up to one year before death.

To select these markers, for each ICD code that appeared in the data, GBD calculated the difference in the proportion of individuals who died with dementia and had a record of each code in the year before death and the proportion of individuals who died without dementia and had a record of the same code in the year before death. GBD reviewed the 150 codes with the highest difference and selected codes that indicated end-stage disease, excluding codes for conditions such as cardiovascular disease. Codes for decubitus ulcer, malnutrition, sepsis, pneumonia, urinary tract infections, falling from bed, senility, dehydration, sodium imbalance, muscular wasting, bronchitis, dysphagia, hip fracture, and bedridden status were used as indicators of severe disease.

In order to determine the proportion of excess deaths that were caused by dementia, GBD calculated the proportion of dementia deaths that had clinical markers of end-stage disease in the year before death, above and beyond the occurrence of end-stage disease markers in those who died without dementia. The subtraction of the proportions with end-stage disease markers in those without dementia from the proportions in those with dementia represents the proportion of individuals who are assumed to have died with severe, end-stage dementia out of total deaths in those with dementia.

Calculation of deaths due to dementia

In order to apply these estimates to the total excess deaths these proportions were adjusted to calculate the proportion of individuals who died with severe, end-stage dementia out of excess dementia deaths using the formula:

$$\frac{Died\ with\ Severe\ Disease}{Excess\ Dementia\ Deaths} = \frac{Died\ with\ Severe\ Disease}{Total\ Dementia\ Deaths} * \frac{Relative\ Risk}{Relative\ Risk - 1}$$

The number of deaths due to dementia were calculated as the product of total excess dementia deaths and the proportion of those who died with severe disease out of excess dementia deaths. These final estimates of deaths due to dementia were then used to adjust data on causes of death from all other causes in vital registration systems.

Interpolation for all years

Finally, log-linear interpolation was used to interpolate these results (limited to 1990, 1995, 2000, 2005, 2010, 2015, 2017, 2019) to create estimates for the entire time series from 1980 to 2019. Socio-demographic Index was used as a covariate to extrapolate back to the year 1980.

E4. Brain and nervous system cancer

The approach for estimating deaths from brain and nervous system cancer are shown in the following flowchart:

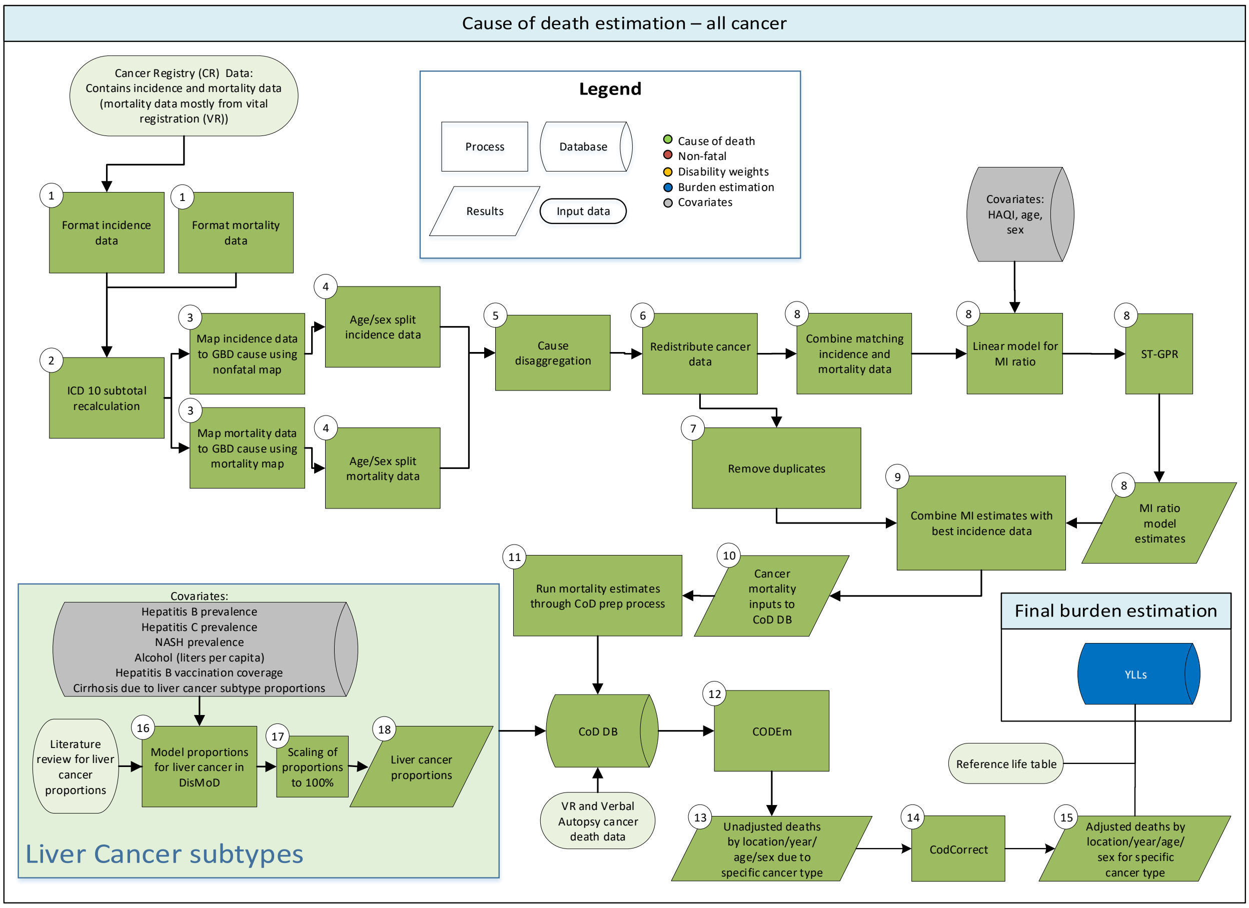

Abbreviations: ICD: International classification of diseases; DB: database, ST-GPR: Space-time smoothing, Gaussian process regression, COD: Causes of death

Data

The cause of death (COD) database contains multiple sources of cancer mortality data. These sources include vital registration, verbal autopsy, and cancer registry data. The cancer registry mortality estimates uploaded into the COD database stem from cancer registry incidence data that have been transformed to mortality estimates through the use of mortality-to-incidence ratios (MIR).

Only population-based cancer registries were included, and only those that included all cancers (no specialty registries), data for all age groups (except for paediatric cancer registries), and data for both sexes. Pathology-based cancer registries were included if they had a defined population. Hospital-based cancer registries were excluded.

Cancer registry data were excluded from either the final incidence data input or the MI model input if a more detailed source (eg, providing more detailed age or diagnostic groups) was available for the same population. Preference was given to registries with national coverage over those with only local coverage, except those from countries where the GBD study provides subnational estimates. Data were excluded if the coverage population was unknown.

Bias of categories of input data

Cancer registry data can be biased in multiple ways. A high proportion of ill-defined cancer cases in the registry data requires redistribution of these cases to other cancers, which introduces a potential for bias. Changes between coding systems can lead to artificial differences in disease estimates; however, this bias was adjusted by mapping the different coding systems to the GBD causes. Underreporting of cancers that require advanced diagnostic techniques can be an issue in cancer registries from low-income countries. On the other hand, misclassification of metastatic sites as primary cancer can lead to overestimation of cancer sites that are common sites for metastases, like the brain. Since many cancer registries are located in urban areas, the

representativeness of the registry for the general population can also be problematic. The accuracy of mortality data reported in cancer registries usually depends on the quality of the vital registration system. If the vital registration system is incomplete or of poor quality, the mortality-to-incidence ratio can be biased to lower ratios.

Modelling strategy

Cancer registry data went through multiple processing steps before integration with the COD database. First, the original data were transformed into standardised files, which included standardisation of format, categorisation, and registry names (#1 in flowchart).

Second, some cancer registries report individual codes as well as aggregated totals (eg, C18, C19, and C20 are reported individually, but the aggregated group of C18-C20 [colorectal cancer] is also reported in the registry data). The data-processing step "subtotal recalculation" verifies these totals and subtracts the values of any individual codes from the aggregates.

In the third step of the flowchart, cancer registry incidence data and cancer registry mortality data were mapped to GBD causes. A different map was used for incidence data and for mortality data because of the assumption that there are no deaths for certain cancers. One of the example is benign or in situ neoplasms. Benign or in situ neoplasms found in the cancer registry incidence dataset were simply dropped from that dataset. The same neoplasms reported in a cancer registry mortality dataset were mapped to the respective invasive cancer.

In the fourth data-processing step of the flowchart, cancer registry data were standardised to the GBD age groups. Age-specific incidence rates were generated using all datasets that include microdata, and datasets that report age groups up to 95+ years of age, while age-specific death rates were generated from the CoD data. Age-specific proportions were then generated by applying the age-specific rates to a given registry population that required age-splitting to produce the expected number of cases/deaths for that registry by age. The expected number of cases/deaths for each sex, age, and cancer were then normalised to 1, creating final, age-specific proportions. These proportions were then applied to the total number of cases/deaths by sex and cancer to get the age-specific number of cases/deaths.

In the rare case when the cancer registry only contained data for both sexes combined, the now-age-specific cases/deaths were split and reassigned to separate sexes using the same weights that are used for the age-splitting process. Starting from the expected number of deaths, proportions were generated by sex for each age (eg, if for ages 15 to 19 years old there are six expected deaths for males and four expected deaths for females, then 60% of the combined-sex deaths for ages 15-19 years would be assigned to males and the remaining 40% would be assigned to females).

In the fifth step of flowchart, data for cause entries that are aggregates of GBD causes were redistributed.

In the sixth step of the flowchart, unspecified codes ("garbage codes") were redistributed. Redistribution of cancer registry incidence and mortality data mirrored the process of the redistribution used in the cause of death database.

In the seventh step of the flowchart, duplicate or redundant sources were removed from the processed cancer registry dataset.

In the eighth step of the flowchart, the processed incidence and mortality data from cancer registries were matched by cancer, age, sex, year, and location to generate MI ratios. These MI ratios were used as input for a three-step modelling approach using ST-GPR, with Healthcare Access and Quality (HAQ) Index as a covariate in the linear step mixed effects model using a logit link function. Predictions were made without the random effects. The ST-GPR model has three main hyper-parameters that control for smoothing across time, age, and geography, which were adjusted for GBD 2019. The time adjustment parameter lambda (λ) aims to borrow strength from neighbouring time points (ie, the exposure in this year is highly correlated with exposure in the previous year but less so further back in time). Lambda was lowered from 2 to 0.05, reducing the weight of more distant years. The age adjustment parameter omega (ω) borrows strength from data in neighbouring age groups and was set to 0.5 (unchanged). The space adjustment parameter zeta (ξ) aims to borrow strength across the hierarchy of geographical locations. Zeta was lowered from 0.95 to 0.01, reducing the weight of more distant geographical data. For the remaining parameters in the Gaussian process regression, GBD lowered the amplitude from 2 to 1 (reducing fluctuation from the mean function) and reduced the scale value from 15 to 10 (reducing the time distance over which points are correlated).

For each cancer, MI ratios from locations in HAQ quintiles 1-4 were dropped if they were below the median of MI ratios from locations in HAQ quintile 5. GBD also dropped MI ratios from locations in HAQ quintiles 1-4 if the MI ratios were above the third quartile + 1.5 * IQR (inter-quartile range). GBD dropped all MIR that were based on less than 15 (this was 25 in 2017) cases to avoid noise due to small numbers. GBD also aggregated incidence and mortality to the youngest five-year age bin where SEER reported at least 50 cases from 1990 to 2015, to avoid unstable MIR predictions in young age groups on too few data points. The MIR in the minimum age-bin was used to backfill the MIR down to the lowest age group estimated for that cancer.

Since MI ratios can be above 1, especially in older age groups and cancers with low cure rates, GBD used the 95th percentile (by age group) of the cleaned dataset (detailed above) to cap the MIR input data. This "upper cap" was used to allow MIR over 1 but to constrain the MIR to a maximum level. To run the logit model, the input data were divided by the upper caps to get data from 0 to 1. Model predictions from ST-GPR were then rescaled back by multiplying them by the upper caps.

To constrain the MIRs at the lower end, GBD used the fifth percentile of the cancer and age-specific cleaned MIR input data to replace all model predictions with this lower cap.

Final MI ratios were matched with the cancer registry incidence dataset in the ninth step (#9 in the flowchart) to generate mortality estimates (Incidence * Mortality/Incidence = Mortality) (#10 in the flowchart). These mortality estimates are then smoothed by a Bayesian noise-reduction algorithm and uploaded into the COD database (#11 in the flowchart). Cancer-specific mortality modelling then followed the general CODEm process.

Covariates used in modelling deaths from brain and nervous system cancer

Level	Covariate	Direction
1	Litres of alcohol consumed per capita	+
	Log-transformed SEV scalar: Thyroid Cancer	+
2	Age- and sex-specific SEV for low vegetables	+
	Age- and sex-specific SEV for high red meat	+
	Tobacco (cigarettes per capita)	+
	Mean BMI	+
	Healthcare Access and Quality Index	−
3	Education (years per capita)	−
	Sanitation (proportion with access)	−
	Improved water source (proportion with access)	−
	Age- and sex-specific SEV for low fruits	+
	LDI (I$ per capita)	+
	Socio-demographic Index	+

E5. Parkinson's disease

The approach for estimating deaths from Parkinson's disease is shown in the following flowchart:

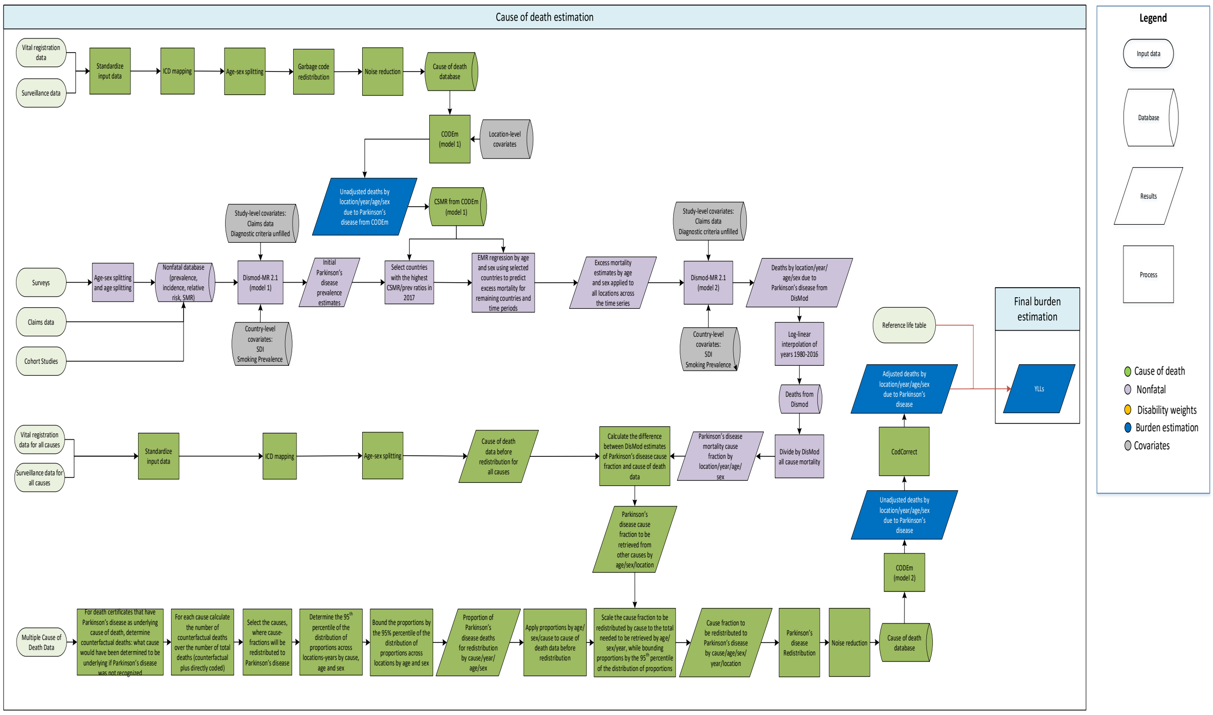

Data

Data used to estimate deaths due to Parkinson's disease included death data from vital registration systems and prevalence data from surveys and claims sources. A systematic review was conducted and studies that reported prevalence, incidence, remission rate, excess mortality rate, relative risk of mortality, standardized mortality ratio, or with-condition mortality rate were included. Studies with no clearly defined sample or that drew from specific clinic/patient organizations were excluded.

Modelling strategy

First, GBD ran a CODEm model for Parkinson's disease and extracted the death rates by age, sex, and geography. The covariates used in this intermediary model are displayed below; some have a direction of 0 because this model was run early in the GBD 2019 cycle. The final Parkinson's model has a negative or positive direction specified for all covariates.

Level	Covariate	Direction
1	Cumulative cigarette consumption (10 years)	-
2	Absolute latitude	+
	Cholesterol (total, mean per capita)	+
	Sanitation (proportion with access)	0
	Improved water source (proportion with access)	0
	Fruit consumption adjusted (g)	-
	Healthcare access and quality index	-
3	Education (years per capita)	-
	Socio-demographic index	+
	Lag distributed income	0

Second, GBD ran a DisMod-MR 2.1 model with all data on incidence, prevalence, and mortality risk (RR, SMR, or with-condition mortality rates) and a setting of zero remission and extracted prevalence by age, sex, and geography. Studies where the case definition of two of the four cardinal symptoms of Parkinson's disease was not filled were crosswalked to studies using the reference case definition. No random effects were used in the model in order to prevent spurious inflation of regional differences due to differences in measurement and measurement error.

Third, the seven countries (France, England, the United States, the Netherlands, Finland, Scotland, and Wales) with the highest cause-specific mortality rate to prevalence ratio in 2019 were selected. These countries also had an age-standardised prevalence rate greater than 0.0005, and a population greater than 1 million.

Fourth, GBD used a linear effects regression with dummies on age group and sex to predict excess mortality (i.e., the ratio of cause-specific mortality rate and prevalence) by age and sex.

Fifth, these estimates were added to a second DisMod-MR 2.1 model as pertaining to the full 1990–2019 estimation period. For the countries included in the regression, GBD allowed them to retain their original excess mortality rates (EMR) when the age-standardized EMR for a country was higher than the age-standardized EMR prediction generated from the regression. These countries retained their age- and sex-specific ratios and entered those also as pertaining to the full 1990–2019 estimation period. Smoking prevalence was used as a country-level covariate. Data for standardized mortality ratio, with-condition mortality rate, and relative risk were excluded as GBD wanted to estimate cause-specific mortality rates that were consistent with the level of excess mortality from the seven chosen countries in 2019.

Sixth, GBD took the predictions of cause-specific mortality by age, sex, geography, and year that DisMod-MR 2.1 calculated as being consistent with the data on incidence, prevalence, and the priors on excess mortality from step five. Because DisMod-MR 2.1 produces estimates in five-year intervals only, GBD expanded the time series by log-linear interpolation; values for 1980-1990 were generated using a regression on the entire time series with Socio-demographic index included as a predictor. GBD divided this cause-specific mortality by the all-cause mortality used in DisMod to calculate the Parkinson’s disease cause-fraction based on prevalence data and the excess mortality derived from countries most likely to code to Parkinson’s disease as a cause of death.

Seventh, GBD calculated the difference between this cause-fraction derived from DisMod and the cause-fraction derived from the cause of death data prep process before redistribution in order to get the amount of cause fraction that needed to be retrieved from other causes through the Parkinson’s disease redistribution process.

Eighth, in order to calculate where these Parkinson’s disease deaths should be retrieved from, multiple cause of death (MCOD) data was analysed. GBD only used data from the US, and asserted that the data from 2010-2015, during which the increases in coding to Parkinson’s disease as a cause of death levelled off, is the reference data.

Ninth, for deaths where Parkinson’s disease is the underlying cause of death in the years 2010-2015, GBD calculated what the underlying cause of death would have been in the counterfactual scenario in which Parkinson’s disease had not been recognized. In order to calculate this counterfactual, GBD examined the causes listed in part one of the chain of the death certificate. For each death certificate chain GBD looked across the entire dataset from 1980-2015 and determine what the distribution of underlying causes of death was in individuals with that particular death certificate chain. Then, the counterfactual deaths were assigned proportionally to the causes that are listed as underlying in these death certificates. If, over the time period, there were less than 1,000 death certificates that had exactly the same death certificate chain, then GBD included all death certificate chains that had those same causes, but which could additionally include other causes in the chain as well. To assign counterfactual deaths for these chains, GBD further subsetted the data to death certificate chains where any of the causes in the original death certificate chain were listed as underlying, determined the distribution of underlying causes of death among just this subset, and then assigned counterfactual deaths proportionally in the same manner.

Tenth, once GBD determined the counterfactual causes of death stemming from all Parkinson’s disease deaths from 2010-2015, the proportion of deaths were calculated by the cause that should be Parkinson’s disease deaths according to the reference data by taking the counterfactual deaths for each cause and dividing by the sum of the counterfactual deaths for that cause plus the directly coded deaths for that cause.

Eleventh, GBD applied the proportions to cause of death data in cause fraction space and scaled the cause fractions to the total mortality cause fraction to be retrieved based on the DisMod model. GBD set caps on the percent of deaths that were moved by age, sex and cause. The caps were determined by finding the 95th percentile of the percentages of deaths moved in each age-sex-cause category across all 5-star vital registration locations. The COD data is then processed using general redistribution strategies and noise reduction.

Finally, the data derived from this process was used in a final CODEm model, using the covariates as the original CODEm model. These covariates were adjusted for this model in GBD 2019 so that every covariate

had a specified directionality (see table below), and with some adjustments for level. These results were then adjusted through CodCorrect and become the final cause of death estimates for Parkinson's disease.

Level	Covariate	Direction
1	Cumulative cigarette consumption (10 years)	-
	Fruit consumption adjusted (g)	-
2	Absolute latitude	+
	Cholesterol (total, mean per capita)	+
	Sanitation (proportion with access)	+
	Improved water source (proportion with access)	+
	Healthcare access and quality index	-
3	Education (years per capita)	-
	Socio-demographic index	+
	Lag distributed income	+

E6. Multiple sclerosis

The approach for estimating deaths from multiple sclerosis is shown in the following flowchart:

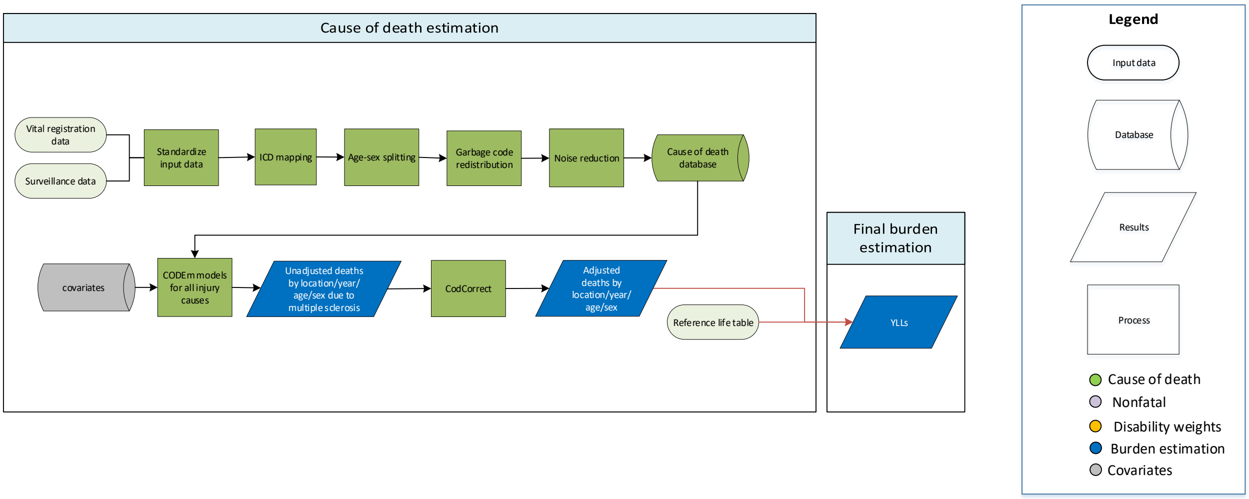

Data

Data used to estimate multiple sclerosis included vital registration and surveillance data from the cause of death database. GBD excluded data if they (1) were implausibly high or low, (2) substantially conflicted with established age or temporal patterns, or (3) substantially conflicted with other data sources conducted from the same locations or locations with similar characteristics (i.e., Socio-demographic Index). In particular, where data-processing could not resolve discrepancies between different coding systems for the same location over time, one system was selected as more reliable and the other was excluded.

Modelling strategy

The standard CODEm modelling approach was used to estimate deaths due to multiple sclerosis. Separate models were used for estimating deaths for male and female, and the age range for both models was 5-95+ years (differing from previous years where the age range was 20-95+ years). The linear floor was set to 0.0001. The full list of covariates used in GBD 2019 are displayed below. Unadjusted death estimates were adjusted using CoDCorrect to produce final estimates of YLLs.

Covariates used in modelling deaths from multiple sclerosis

Level	Covariate	Direction
1	Absolute value of average latitude	+
2	Mean serum total cholesterol (mmol/L)	+
	Health care access and quality index	-
3	Cumulative cigarettes (10 years)	+
	Cumulative cigarettes (5 years)	+
	Education (years per capita)	-
	Log-transformed LDI (per capita)	-
	Smoking prevalence	+
	Socio-demographic Index	+

E7. Motor neuron diseases

The approach for estimating deaths from motor neuron diseases are shown in the following flowchart:

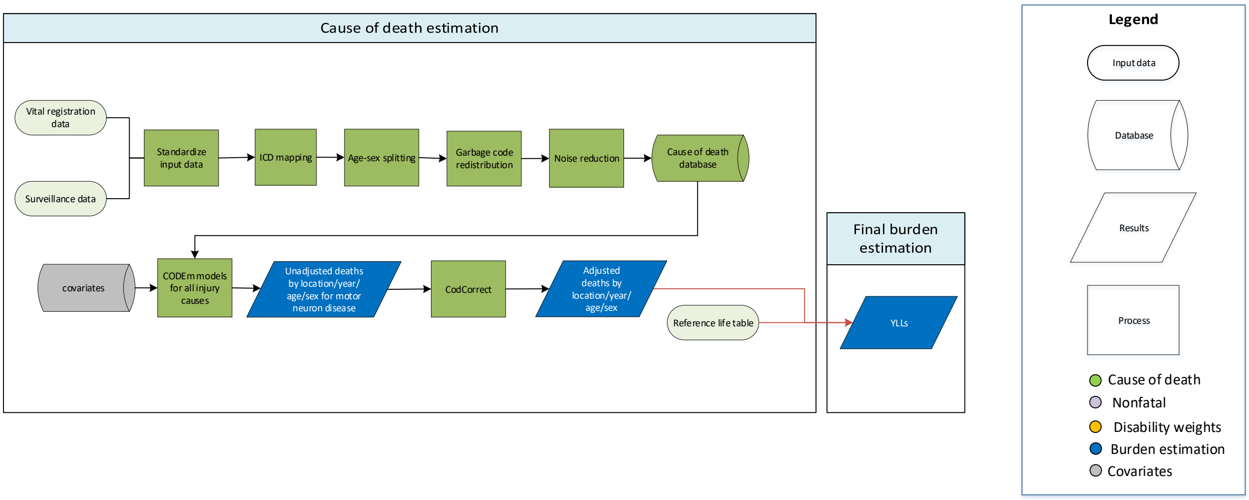

Data

Data used to estimate motor neuron diseases included vital registration and surveillance data from the cause of death database. GBD excluded data points that (1) were implausibly high or low, (2) substantially conflicted with established age or temporal patterns, or (3) substantially conflicted with other data sources from the same locations or locations with similar characteristics (i.e., Socio-demographic Index).

Modelling strategy

The standard CODEm modelling approach was used to estimate deaths due to multiple sclerosis. Separate models were used for estimating deaths for male and female, and the age range for both models was 0-days to 95+ years. Unadjusted death estimates were adjusted using CoDCorrect to produce final estimates of YLLs.

Covariates used in modelling deaths from motor neuron diseases

Level	Covariate	Direction
1	Mean total body mass index (kg/m^2)	-
	Mean serum total cholesterol (mmol/L)	-
	Absolute value of average latitude	+
	Mean diabetes fasting plasma glucose (mmol/L)	+
	Fruit consumption (grams per day adjusted)	-
	Socio-demographic Index	+
	Health care access and quality index	-
2	Population-weighted mean temperature	-
	Sanitation (proportion with access)	+
	Improved water source (proportion with access)	-
3	Education (years per capita)	+
	Log-transformed LDI (per capita)	+

E8. Encephalitis

The approach for estimating deaths from encephalitis is shown in the following flowchart:

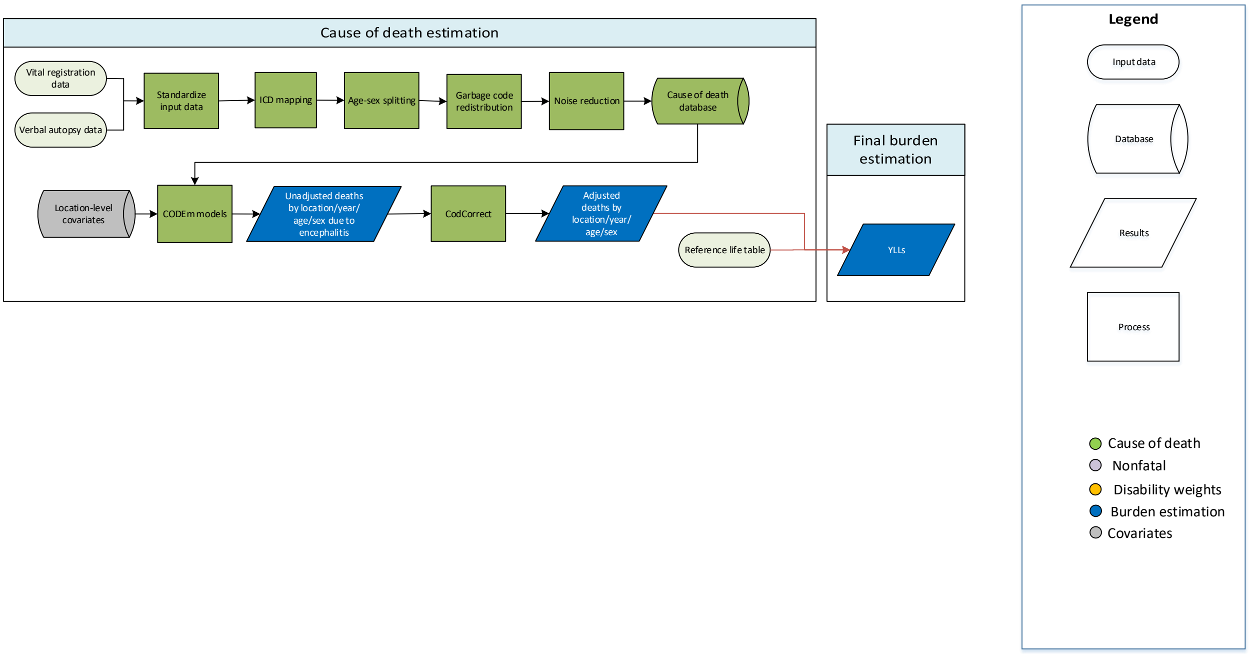

Data

Vital registration and verbal autopsy data were used to model the cause of death estimation of encephalitis. The data was outliered in instances where garbage code redistribution and noise reduction, in combination with small sample sizes, resulted in unreasonable cause fractions when compared to regional, super-regional, and global rates, and data that violated well-established time or age trends. Outliering methods were consistent across both vital registration and verbal autopsy data.

Modelling strategy

Deaths due to encephalitis were modelled with a standard CODEm model using the cause of death database and location-level covariates as inputs. Separate global and data-rich models were hybridised to acquire unadjusted results, which were adjusted using CodCorrect to reach final years of life lost due to encephalitis.

Covariates used in modelling deaths from encephalitis

Level	Covariate	Direction
1	Japanese encephalitis binary	+
	Age- and sex-specific summary exposure value for child underweight	+
2	Log-transformed lag distributed income	-
	Healthcare Access and Quality Index	-
	Maternal care and immunization	-
3	Squared proportion of in-facility deliveries	-
	Socio-demographic Index	-
	Logit-transformed sanitation (proportion with access)	-
	Logit-transformed water (proportion with access)	-
	Diphtheria-tetanus-pertussis third-dose vaccination coverage	-
	Maternal education (years per capita)	-

E9. Meningitis

The approach for estimating deaths from meningitis is shown in the following flowchart:

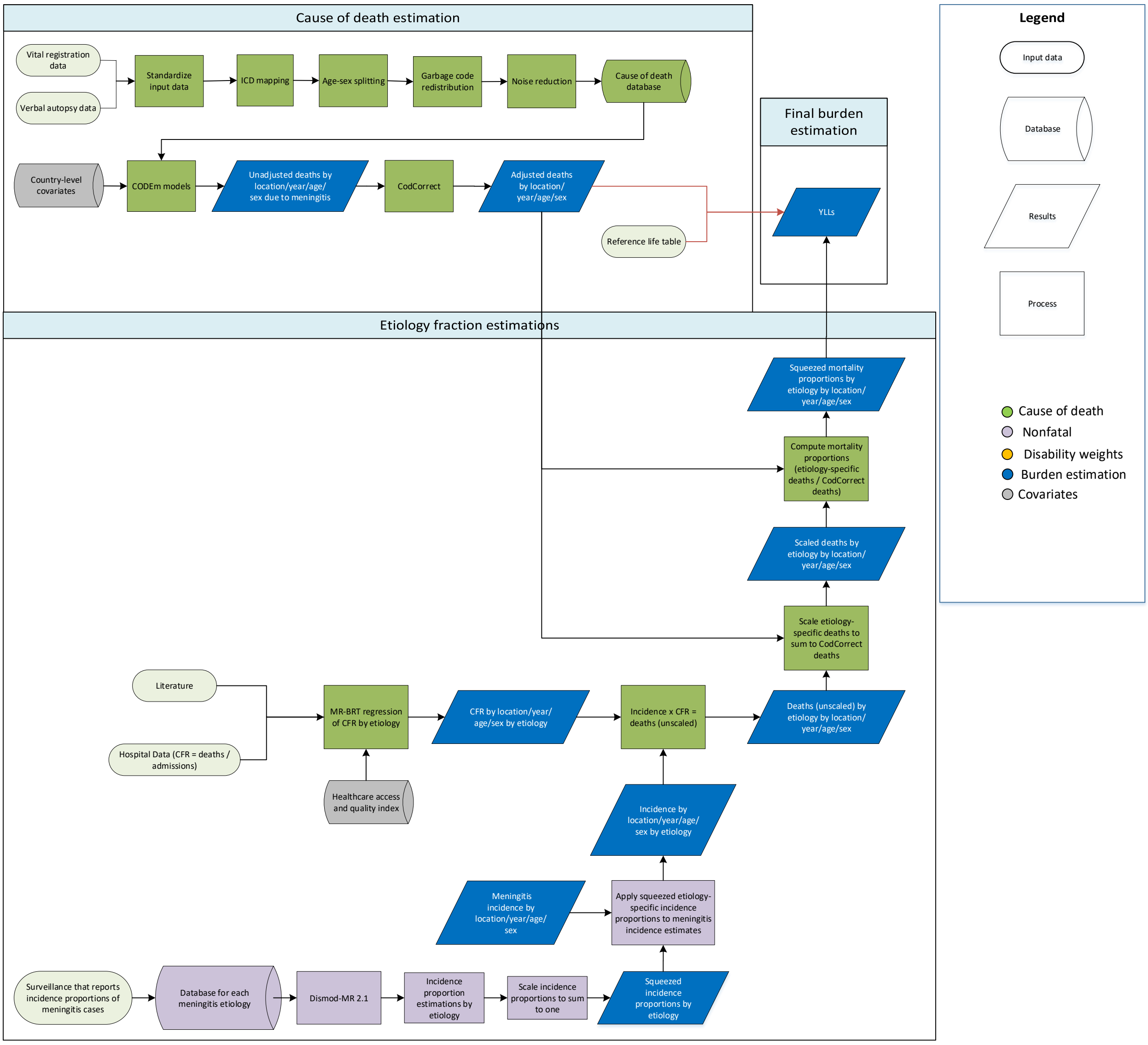

Data

Input data for the overall meningitis model came from the cause of death database, which includes vital registration and verbal autopsy data. The data was outliered in instances where garbage code redistribution and noise reduction, in combination with small sample sizes, resulted in unreasonable cause fractions when compared to regional, super-regional, and global rates, and data that violated well-established time or age trends. Outliering methods were consistent across both vital registration and verbal autopsy data.

Modelling strategy

Deaths due to meningitis were modelled with two CODEm models, separately for each sex and two age categories – under 5 and 5 years and above. The mortality trends differ substantially between children and adults, and there are a significant number of data sources that only have data for children under 5. The two models used the same covariates (with the exception of the covariate for underweight, which is age-specific) and otherwise standard CODEm parameters. The final sex-specific models for deaths due to all meningitis were a hybridised model of separate global and data-rich models for males and females.

Death estimates for each of the three aetiologies of bacterial meningitis – meningococcal, pneumococcal, *H. influenzae* type B – were derived from aetiology-specific incidence and case fatality rate estimates.

The aetiology-specific deaths were then squeezed to the total number meningitis deaths after CoDCorrect and meningococcal shocks deaths were included at the draw level.

Covariates used in modelling deaths from meningitis (0–4 years, 5–95+ years)

Level	Covariate Name	Direction
1	Meningitis belt (proportion of population in belt)	+
1	MenAfriVac coverage	-
1	H. influenzae type B proportion covered	-
1	PCV3 coverage proportion	-
2	Age- and sex-specific summary exposure value for child underweight	+
2	Logit-transformed water (proportion with access)	-
2	Maternal care and immunization	-
2	Healthcare Access and Quality Index	-
3	Log-transformed lag distributed income	-
3	Sanitation (proportion with access)	-
3	Maternal education (years per capita)	-
3	Socio-demographic Index	-

E10. Tetanus

The approach for estimating deaths from tetanus is shown in the following flowchart:

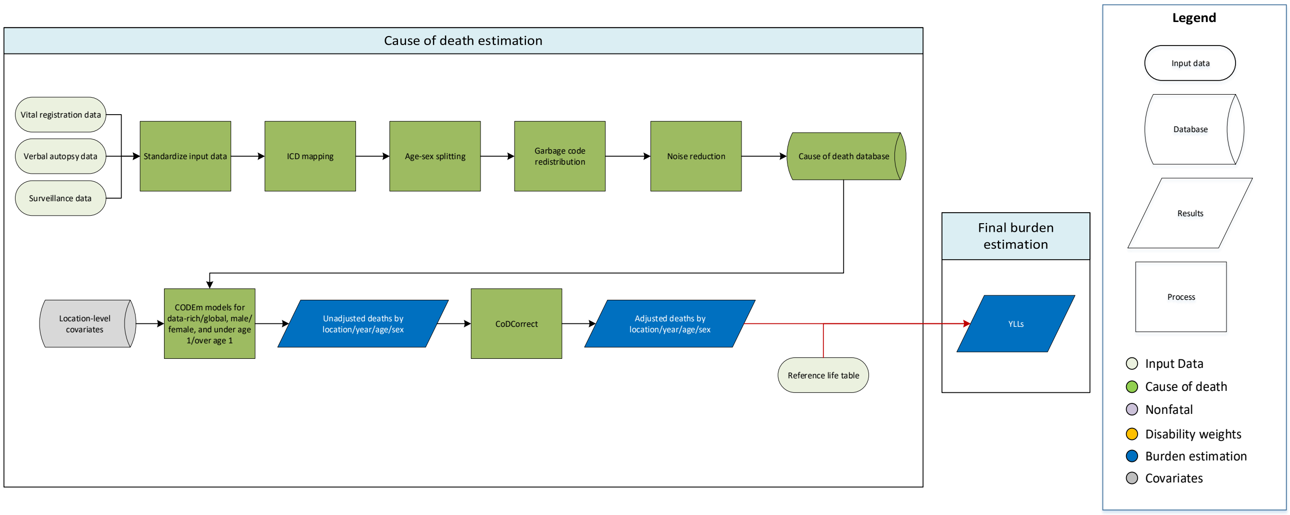

Data

Tetanus cause of death data included vital registration, verbal autopsy, and surveillance sources from all locations as available. Data was excluded if it was highly incongruent with other available data from the same location or locations of similar sociodemographic characteristics (i.e., Socio-demographic Index).

Modelling strategy

Cause of Death Ensemble modelling approach (CODEm) was used to compute age-, sex-, location-, and year-specific estimates. Given the relative rarity of tetanus mortality, it was modelled directly in count space. These models in count space had lower out-of-sample root mean squared error (RMSE) than rate-space models, and thus were frequently the top models selected in the ensemble.

Separate, sex-specific models were run for neonatal tetanus (under 1-year age groups) and all other tetanus (1 year to 95+ age groups). Models were also stratified by vital registration data quality, running both “data-rich” and global models for each age- and sex-specific group. Following model completion, the data-rich and global model outputs were combined to produce a single set of estimates for all locations by sex and age (under 1 and over 1 age groups). Tables below lists the covariates used in the under-1 models and the over-1 model.

Summary of covariates used in the under-1 tetanus cause of death model

Level	Covariate	Direction
1	Diphtheria-tetanus-pertussis third-dose vaccination coverage	-
	Tetanus toxoid coverage	-
2	In-facility deliveries (proportion)	-
	Skilled birth attendance (proportion)	-
	Healthcare Access and Quality Index	-
3	Lag-distributed income	-
	Socio-demographic Index	-
	Mean years of education per capita	-

Summary of covariates used in the over-1 tetanus cause of death model

Level	Covariate	Direction
1	Diphtheria-tetanus-pertussis third-dose vaccination coverage	-
2	Healthcare Access and Quality Index	-
3	Sanitation access (proportion)	-
	Lag-distributed income	-
	Socio-demographic Index	-
	Mean years of education per capita	-

F. Estimation of risk factors for neurological disorders

The analytical approach used in GBD 2019 for comparative risk assessment to estimate population attributable fractions for risk factors are shown in the following flowchart:

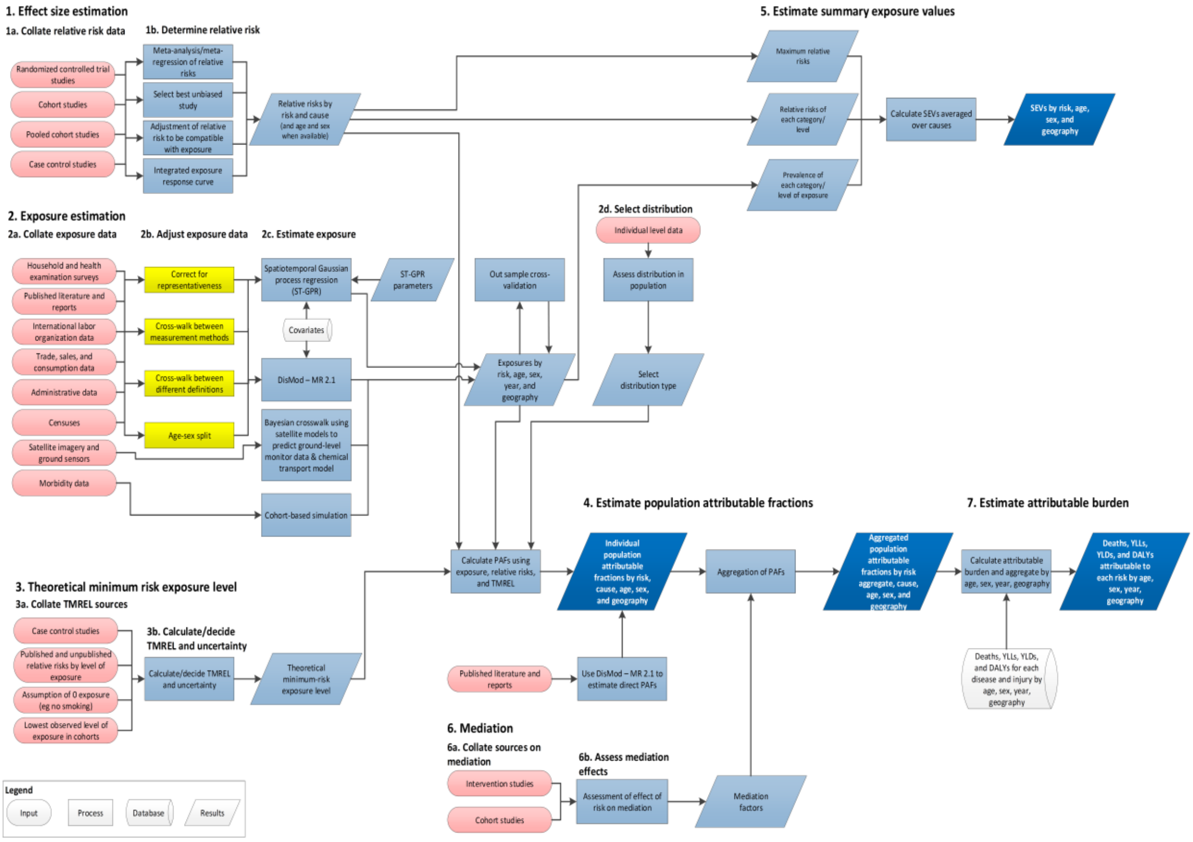

GBD is Global Burden of Disease. SEV is summary exposure value. TMREL is theoretical minimum-risk exposure level. PAF is population attributable fraction. YLL is years of life lost. YLD is years lived with disability. DALY is disability adjusted life-years. Ovals represent data inputs, rectangular boxes represent analytical steps, cylinders represent databases, and parallelograms represent intermediate and final results.

We describe details of the major risk factors related to neurological disorders, i.e. high systolic blood pressure, air pollution, dietary risks, high fasting plasma glucose, smoking, secondhand smoke, high body-mass index, impaired kidney function, other environmental risks, high low-density lipoprotein cholesterol level, non-optimal temperature, alcohol use, low physical activity, low birthweight and short gestation. Description of other risk factors can be found in the GBD 2019 risk factor paper (Lancet 2020; 396: 1223–49).

F1. High systolic blood pressure

In the GBD analysis, high systolic blood pressure (SBP) is associated with increased risk for stroke among the neurological disorders. For the purpose of attributing disease burden to high SBP, TMREL for SBP range from 110 to 115 mmHg based on pooled prospective cohort studies that showed risk of mortality increases for SBP above this level.[1,2] To include the uncertainty in the TMREL, a random draw was taken from the uniform distribution of the interval between 110 mmHg and 115 mmHg each time the population attributable burden was calculated.

The steps in the estimation of disease burden attributable to high systolic blood pressure are shown in the following flowchart:

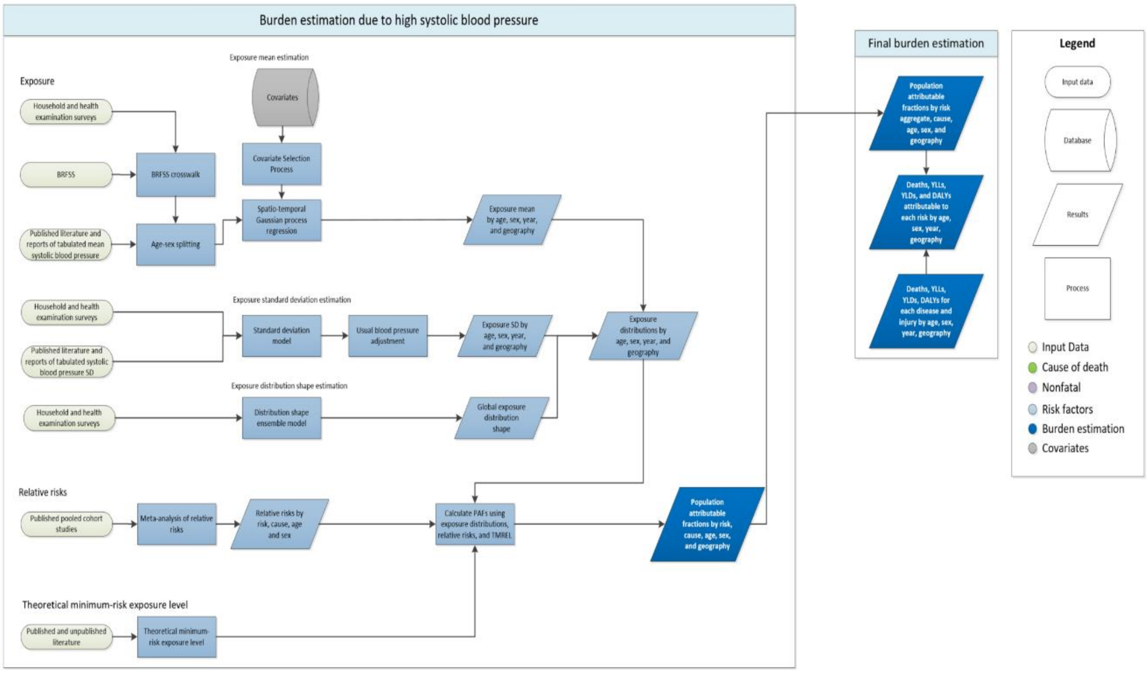

Data

Data on mean systolic blood pressure were used from literature and from household survey microdata and reports. Studies were included if they were population-based and directly measured systolic blood pressure using a sphygmomanometer. The data were assumed to be representative if the geography or the population were not selected because it was related to hypertension or hypertensive outcomes.

Data were utilised in the modelling process unless an assessment strongly suggested that the source was biased. A candidate source was excluded if the quality of study did not warrant a valid estimate because of selection (non- representative populations) or did not provide methodological details for evaluation. In a small number of cases, a data point was considered to be an outlier candidate if the level was widely inconsistent with data from other country.

Individual-level data on blood pressure estimates extracted from survey microdata were collapsed across demographic groupings to produce mean estimates in the standard GBD five-year age-sex groups. If microdata were unavailable, information from survey reports or from literature were extracted along with any available measure of uncertainty including standard error, uncertainty interval, and sample size. Standard deviations were also extracted. Where mean SBP was reported split out by groups other than age, sex, location, and year (e.g. by hypertensive status), a weighted mean was calculated.

Prior to modelling, data provided in age groups wider than the GBD 5-year age groups were processed using the approach outlined in Ng and colleagues.[3] Briefly, age-sex patterns were identified using sources of data with multiple age-sex groups, and these patterns were applied to estimate age-sex-specific levels of mean systolic blood pressure from aggregated results reported in published literature or survey reports. In order to incorporate uncertainty into this process and borrow strength across age groups when constructing the age-

sex pattern, a model with auto-regression on the change in mean SBP over age groups was used. Draws of the age-sex pattern were combined with draws of the input data needing to be split in order to calculate the new variance of age-sex split data points.

Modelling strategy

Exposure estimates were produced from 1980 to 2019 for each national and subnational location, sex, and for each five-year age group starting from 25+. A spatiotemporal Gaussian process regression (ST-GPR) framework was used to model the mean SBP at the location-, year-, age-, sex-level.

The first step of the ST-GPR framework requires the creation of a linear model for predicting SBP at the location-, year-, age-, sex-level. Covariates for this model were selected in two stages. First a list of variables with an expected causal relationship with SBP was created based on significant association found within high quality prospective cohort studies reported in the published scientific literature. The second stage in covariate selection was to test the predictive validity of every possible combination of covariates in the linear model, given the covariates selected above. This was done separately for each sex. Predictive validity was measured with out of sample root-mean-squared error. Ensemble with the lowest root-mean-squared error for each sex was then used in the ST-GPR model. The result of the ST-GPR model are estimates of the mean SBP for each age, sex, location, and year.

The standard deviation of SBP within a population was estimated for each national and subnational location, sex, and five-year age group starting from age 25 using the standard deviation mainly from person-level and some tabulated data sources. Tabulated data were only used to model standard deviation if it was sex-specific and five-year-age-group-specific and reported a population standard deviation of SBP. The SBP standard deviation function was estimated using a linear regression:

$$\log\left(\mathrm{SD}_{l,a,t,s}\right) = \beta_0 + \beta_1 \log(\mathrm{mean\_SBP}_{l,a,t,s}) + \beta_4 \mathrm{sex} + \sum_{k=2}^{16} \beta_k I_A$$

Where $\mathrm{mean\_SBP}_{l,a,t,s}$ is the location, age, time, and sex specific mean SBP estimate from ST-GPR, and I_A is a dummy variable for a fixed effect on a given 5-year age group.

To account for in-person variation in SBP, a 'usual blood pressure' adjustment was done. Measurements of a risk factor taken at a single time point may not accurately capture an individual's true long-term exposure to that risk. Blood pressure readings are highly variable over time due to measurement error as well as diurnal, seasonal, or biological variation. These sources of variation result in an overestimation of the variation in cross-sectional studies of the distribution of SBP. To adjust for this overestimation, a correction factor was applied to each location-, age-, time-, and sex-specific standard deviation. These correction factors were age-specific and represented the proportion of the variation in blood pressure within a population that would be observed if there were no within person variation across time. Four longitudinal surveys were used to estimate these factors: the China Health and Retirement Longitudinal Survey, the Indonesia Family Life Survey, the National Health and Nutrition Examination Survey I Epidemiological Follow-up Study, and the South Africa National Income Dynamics Survey.

For each survey, the following regression was created for each age group:

$$\mathrm{SBP}_{i,a} = \beta_0 + \beta_1 \mathrm{sex} + \beta_3 \mathrm{age} + +\upsilon_i$$

Where SBPi,a is the systolic blood pressure of an individual i at age a, sex is a dummy variable for the sex of an individual, age is a continuous variable for the age of an individual, and υ_i is a random intercept for each individual. Then, a blood pressure value $\widehat{\mathrm{SBP}}_{i,b}$ was predicted for each individual i for his/her age at baseline b. The correction factor cf for each age group within each survey was calculated as variation in these predicted blood pressures was divided by the variation in the observed blood pressures at baseline, $\mathrm{SBP}_{i,b}$:

$$\mathrm{cf} = \sqrt{\frac{\mathrm{var}\left(\widehat{\mathrm{SBP}}_b\right)}{\mathrm{var}(\mathrm{SBP}_b)}}$$

The average of the correction factors was taken over the three surveys to get one set of age-specific correction factors, which were then multiplied by the square of the modelled standard deviations to estimate

standard deviation of the ‘usual blood pressure’ of each age, sex, location, and year. Because of low sample sizes, the correction factors for the 75-79 age group were used for all terminal age groups.
The shape of the distribution of SBP was estimated using all available person-level microdata sources, which was a subset of the input data into the modelling process. Ensemble distribution was created from a weighted average of distribution families was fit for each individual microdata source, separately by sex. The weights for the distribution families for each individual source were then averaged and weighted to create a global ensemble distribution for each sex.

The estimates of RR for stroke were taken from the CALIBER study[4] and from two pooled epidemiological studies: the Asia Pacific Cohort Studies Collaboration[2] and the Prospective Studies Collaboration.[5] These epidemiological studies have shown that the RR for stroke associated with SBP declines with age, with the log (RR) having an approximately linear relationship with age and reaching a value of 1 between the ages of 100 and 120. RRs were reported per 10 mmHg increase in SBP above the TMREL value (115 mmHg), calculated as in the equation below:

$$RR(x) = RR_0^{\frac{(x-TMREL)}{10\,mmHg}}$$

Where $RR(x)$ is the RR at exposure level x and RR_0 is the increase in RR for each 10 mmHg above the TMREL. Dismod-MR 2.1 was used to pool effect sizes from included studies and generate dose response curve for the outcomes associated with high SBP. The tool enabled us to incorporate random effects across studies and include data with different age ranges. RRs were used universally for all countries and the meta-regression only helped to pool the three major sources and produce RRs with uncertainty and covariance across ages taking into account the uncertainty of the data points. Estimates of exposure to high SBP and relative risks for stroke were then used to calculate the population attributable fractions for stroke attributable to high SBP.

References

1. Singh GM, Danaei G, Farzadfar F, et al. The age-specific quantitative effects of metabolic risk factors on cardiovascular diseases and diabetes: a pooled analysis. *PloS One* 2013; 8: e65174.
2. Lawes CM, Rodgers A, Bennett DA, Parag V, Suh I, Ueshima H, MacMahon S; Asia Pacific Cohort Studies Collaboration. Blood pressure and cardiovascular disease in the Asia Pacific region. *J Hypertens* 2003; 21: 707–16.
3. Ng M, Fleming T, Robinson M, et al. Global, regional, and national prevalence of overweight and obesity in children and adults during 1980–2013: a systematic analysis for the Global Burden of Disease Study 2013. *Lancet* 2014; 384: 766–81.
4. Rapsomaniki E, Timmis A, George J, et al. Blood pressure and incidence of twelve cardiovascular diseases: lifetime risks, healthy life-years lost, and age-specific associations in 1·25 million people. *Lancet* 2014; 383: 1899–911.
5. Prospective Studies Collaboration. Age-specific relevance of usual blood pressure to vascular mortality: a meta-analysis of individual data for one million adults in 61 prospective studies. *Lancet* 2002; 360: 1903–13.

F.2 Air pollution

Air pollution in GBD consists mainly of ambient air pollution and household air pollution. The exposure to these and the disease burden caused by them are estimated separately in GBD.

F2.1. Ambient particulate matter pollution

Exposure to ambient particulate matter pollution is defined as the population-weighted annual average mass concentration of particles with an aerodynamic diameter less than 2.5 micrometers ($PM_{2.5}$) in a cubic meter of air at a spatial resolution of 0.1° x 0.1° over the globe, which is approximately 11 x 11 km at the equator. This measurement is reported in $\mu g/m^3$. These estimates were based on multiple satellite-based aerosol optical depth data globally combined with a chemical transport model, and calibration of these with $PM_{2.5}$ data from the ground-level monitoring stations.

In the GBD analysis, exposure to ambient particulate matter pollution is associated with increased risk for stroke and meningitis among the neurological disorders. For the purpose of attributing disease burden to ambient air pollution, the TMREL for ambient air pollution was defined as population-weighted mean between 2.4 and 5.9 $\mu g/m^3$, bounded by the minimum and fifth percentiles of exposure distributions from outdoor air pollution cohort studies. The uniform distribution represents the uncertainty regarding adverse effects of low-level exposure. To include the uncertainty in the TMREL, GBD took a random draw from the uniform distribution of the interval between 2.4 and 5.9 $\mu g/m^3$ each time the population attributable burden was calculated. TMREL was defined as a uniform distribution rather than a fixed value in order to represent the uncertainty regarding the level at which the scientific evidence was consistent with adverse effects of exposure. The specific OAP cohort studies selected for this averaging were based on the criteria that their fifth percentiles were less than that of the American Cancer Society Cancer Prevention II cohort's fifth percentile of 8.2 based on Turner et al.[1]

The steps in the estimation of disease burden attributable to ambient particulate matter pollution are shown in the following flowchart:

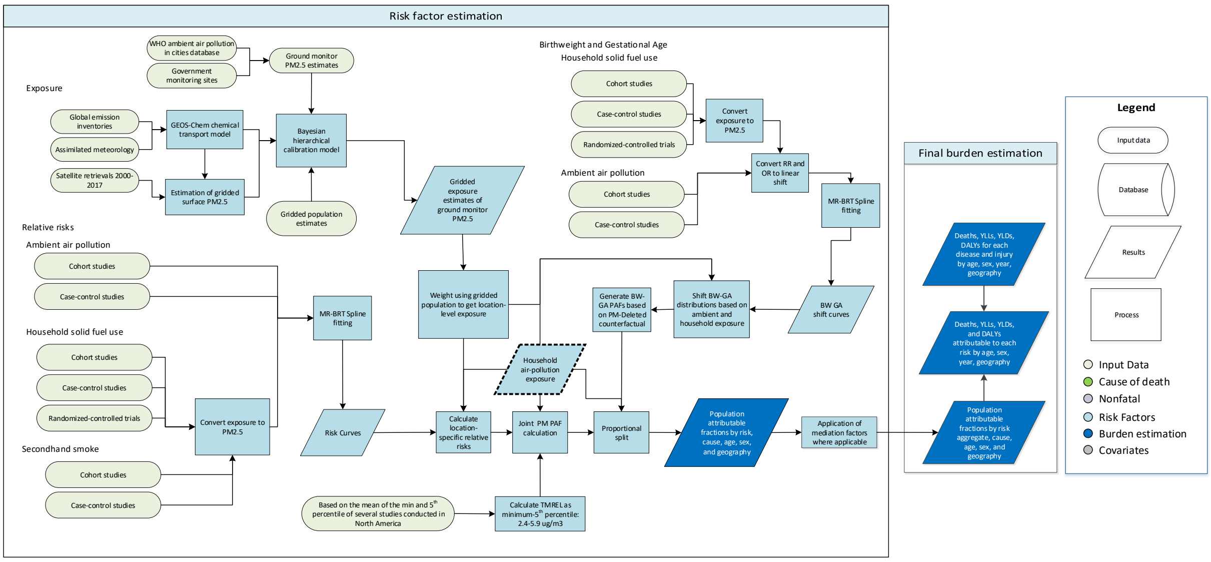

Data

The estimates of ambient $PM_{2.5}$ exposures in India were based on multiple satellite-based aerosol optical depth data combined with a chemical transport model, and calibration of these with $PM_{2.5}$ data from ground-level monitoring stations.

$PM_{2.5}$ ground measurements: Monitor-specific measurements as reported in the WHO Global Ambient Air Quality Database were used, resulting in measurements of concentrations of PM_{10} and $PM_{2.5}$ from over 10,000 ground monitors from 116 countries. For locations measuring only PM_{10}, $PM_{2.5}$ measurements were estimated from PM_{10}. This was performed using a locally derived conversion factor ($PM_{2.5}/PM_{10}$ ratio, for stations where measurements are available for the same year) that was estimated using population-weighted averages of location-specific conversion factors for the country or state. If country-level conversion factors were not available, the average of country-level conversion factors within a region were used. Additionally,

information related to the ground measurements was also included where available, including monitor geo-coordinates and monitor site type.

Satellite-based estimates: These estimates were available at 0.1×0.1° resolution (~11 x 11 km resolution at the equator) and combine aerosol optical depth retrievals from multiple satellites with the updated GEOS Chem chemical transport model and land use information. Updates to the GEOS-Chem simulation includes improved representation of mineral dust and secondary organic aerosol, as well as updated emission inventories. The resultant geophysical $PM_{2.5}$ estimates were highly consistent with ground monitors worldwide Additionally, data from MAIAC at 1 km resolution were also used to estimate $PM_{2.5}$ at the global scale.

Population data: A comprehensive set of population data on a high-resolution grid was obtained from the Gridded Population of the World (GPW) database. These data are provided on a 0.0083°× 0.0083° resolution. Aggregation to each 0.1°×0.1° grid cell was accomplished by summing the central 12 × 12 population cells.

Chemical transport model simulations: Estimates of the sum of particulate sulfate, nitrate, ammonium, and organic carbon and the compositional concentrations of mineral dust simulated using the GEOS Chem chemical transport model, and a measure combining elevation and the distance to the nearest urban land surface were available for 2000–2017 for each 0.1°×0.1° grid cell.[2,3]

Modelling strategy

An updated Data Integration Model for Air Quality (DIMAQ-2) was used for ambient air pollution modelling.[3,4] The coefficients in the calibration model were estimated for each country. Where data were insufficient within a country, information can be 'borrowed' from a higher aggregation (region) and, if enough information is still not available, from an even higher level (super-region). Individual country-level estimates were therefore based on a combination of information from the country, its region, and its super-region. This was implemented within a Bayesian hierarchical modelling (BHM) framework. BHMs provide an extremely useful and flexible framework in which to model complex relationships and dependencies in data. Uncertainty can also be propagated through the model, allowing uncertainty arising from different components, both data sources and models, to be incorporated within estimates of uncertainty associated with the final estimates. The results of the modelling comprise a posterior distribution for each grid cell, rather than just a single point estimate, allowing a variety of summaries to be calculated. The primary outputs here are the median and 95% credible intervals for each grid cell.

In DIMAQ-2 model within-country variation in calibration was included. The ground measurements were matched with other inputs (over time), and the (global-level) coefficients were allowed to vary over time, subject to smoothing that is induced by a first-order random walk process. In addition, the manner in which spatial variation can be incorporated within the model was developed: where there are sufficient data, the calibration equations can now vary (smoothly) both within and between countries, achieved by allowing the coefficients to follow (smooth) Gaussian processes. Where there are insufficient data within a country, information was borrowed from lower down the hierarchy and it was supplemented with information from the wider region to produce accurate equations.

Due to both the complexity of the models and the size of the data, notably the number of spatial predictions that were required, recently developed techniques that perform 'approximate' Bayesian inference based on Integrated Nested Laplace Approximations (INLA) were used.[4] Computation was performed using the R interface to the INLA computational engine. Also, samples from the Bayesian model were used to represent distributions of estimated concentrations in each grid-cell. Estimates and distributions representing uncertainty of concentrations for each grid was obtained by taking repeated (joint) samples from the posterior distributions of the parameters and calculating estimates based on a linear combination of those samples and the input variables.[5]

DIMAQ-2 was used to produce estimates of ambient $PM_{2.5}$ for 1990, 1995, and 2010–2019 by matching the gridded estimates with the corresponding coefficients from the calibration. As there is a lag in reporting ambient air pollution based quantities, the input variables were extrapolated, allowing estimates for 2018 and 2019 to be produced in the same way as other years and, crucially, allowing measures of uncertainty to be produced within the BHM framework rather than by using post-hoc approximations.

Estimates from the satellites and the GEOS-Chem chemical transport model in 2018 and 2019 were produced by extrapolating estimates from 2000–2017 using generalised additive models,[6] on a cell-by-cell basis, except in those grid cells that saw a >100% increase between 2016 and 2017, in which case only the 2000–2016 estimates were used for extrapolating, in order to avoid unrealistic and/or unjustified extrapolation of trends. Population estimates for 2018 and 2019 were obtained by interpolation.

Model development and comparison was performed using within- and out-of-sample assessment. In the evaluation, cross validation was performed using 25 combinations of training (80%) and validation (20%) datasets. Validation sets were obtained by taking a stratified random sample, using sampling probabilities based on the cross-tabulation of $PM_{2.5}$ categories (0-24.9, 25-49.9, 50 74.9, 75-99.9, 100+ $\mu g/m^3$) and super-regions, resulting in them having the same distribution of $PM_{2.5}$ concentrations and super-regions as the overall set of sites. The following metrics were calculated for each training/evaluation set combination: for model fit - R^2 and deviance information criteria (DIC, a measure of model fit for Bayesian models); for predictive accuracy-RMSE and population weighted root mean squared error.

All modelling was performed on the log-scale. The choice of which variables were included in the model was made based on their contribution to model fit and predictive ability. The list of variables and model structures that were considered in developing the model are below.

**Variable**	**Model structure**
Continuous explanatory variables	(SAT) Estimate of $PM_{2.5}$ (in μgm-3) from satellite remote sensing on the log scale.
	(POP) Estimate of population for the same year as SAT on the log-scale.
	(SNAOC) Estimate of the sum of sulphate, nitrate, ammonium and organic carbon simulated using the GEOS Chem chemical transport model.
	(DST) Estimate of compositional concentrations of mineral dust simulated using the GEOS Chem chemical transport model.
	(EDxDU) The log of the elevation difference between the elevation at the ground measurement location and the mean elevation within the GEOS Chem simulation grid cell multiplied by the inverse distance to the nearest urban land surface.
Discrete explanatory variables	(LOC) Binary variable indicating whether exact location of ground measurement is known.
	(TYPE) Binary variable indicating whether exact type of ground monitor is known.
	(CONV) Binary variable indicating whether ground measurement is $PM_{2.5}$ or converted from PM_{10}.
Random effects	Regional temporal (random walk) hierarchical random-effects on the intercept
	Regional hierarchical random-effects for the coefficient associated with SAT
	Regional hierarchical random-effects for the coefficient associated with POP
	Smoothed, spatially varying random-effects for the intercept
	Smoothed, spatially varying random-effects for the coefficient associated with SAT
	Super-region random effects were assumed to be independent and identically distributed.
Interactions	Interactions between the binary variables and the effects of SAT.

For stroke and meningitis, results were used from cohort and case-control studies of ambient $PM_{2.5}$ pollution, cohort studies, case-control studies, and randomised-controlled trials of household use of solid fuel for cooking, and cohort and case-control studies of secondhand smoke. Incidence and mortality due to stroke were extracted from all available studies and was included as a covariate in the model. There was no significant difference between estimates of incidence risk and mortality risk, so both types of risk estimates were included in the curve fitting and the same curve was used for both incidence and mortality. Active smoking data was not used in the risk curves because of the inclusion of more estimates at high $PM_{2.5}$ levels in the model available from the recent publications and additional studies of HAP.

For stroke, evidence suggests that the relative risk decreases with age.[7] To account for this in the model, unique risk curves were generated for stroke for every five-year age group from 25–29 years to 95 years and older. Because the risk data for every unique age group is not available, each study was adjusted based on the median age during follow-up to generate a full adjusted dataset for every curve. The median age of follow-up was calculated by taking the median (or mean) age at enrollment and adding one-half of median or mean follow-up time. If follow-up time was not available, 70% of total study period was taken based on the observed ratio of follow-up time to total study period for other studies. Using the median age during follow-up, each study was extrapolated to the full set of ages where the estimated data point for age was calculated.

MR-BRT splines were used to fit the risk data with a more flexible shape. The curve was fit beginning at zero exposure and incorporate the TMREL into the relative risk calculation process. The published relative risk over a range of exposure data was considered for fitting the risk curves. For OAP studies, the relative risk informs the curve from the fifth to the 95th percentile of observed exposure. When this is not available in the published study, the distribution was estimated from the provided information (mean and standard deviation, mean and IQR, etc.). The RR were scaled to this range.

For HAP studies, each study was allowed to inform the curve from the ExpOAP to ExpOAP+ExpHAP, where ExpOAP is the GBD 2017 estimate of the ambient exposure level in the study location and year, and ExpHAP is the GBD 2017 estimate of the excess exposure for those who use solid fuel for cooking in the study location and year. For SHS studies, an updated strategy of exposure estimation was used for accounting for outdoor exposure. Similar to the approach used for HAP, each study was allowed to inform the curve from the ExpOAP to ExpOAP+ExpSHS, where ExpOAP is the GBD 2017 estimate of the ambient exposure level in the study location and year, and ExpSHS is an estimate of the excess exposure for those who experience secondhand smoke.

Splines on the datasets were fitted including studies of OAP, HAP, and SHS using the following functional form, where X and XCF represent the range of exposure characterised by the effect size:

$$MRBRT(X) - MRBRT(X_{CF}) \sim Shift$$

For each of the risk-outcome pairs, various model settings and priors were tested in fitting the MR-BRT splines. The final models used third-order splines with two interior knots and a constraint on the right-most segment, forcing the fit to be linear rather than cubic. Ensemble approach was used to knot placement, wherein 100 different models were run with randomly placed knots and then combined by weighting based on a measure of fit that penalises excessive changes in the third derivative of the curve. Knots were free to be placed anywhere within the fifth and 95th percentile of the data, as long as a minimum width of 10% of that domain exists between them. Shape constraints were included so that the risk curves were concave down and monotonically increasing, the most biologically plausible shape for the $PM_{2.5}$ risk curve. On the non-linear segments, a Gaussian prior on the third derivative of mean 0 and variance 0.01 was used to prevent over-fitting; on the linear segment, a stronger prior of mean 0 and variance 1e-6 was used to ensure that the risk curves do not continue to increase beyond the range of the data.

For the proportion of the population not exposed to HAP the relative risk was:

$$RR_{OAP} = MRBRT(z = Exp_{OAP})/MRBRT(z = TMREL)$$

And for those exposed to HAP, the relative risk was

$$RR_{HAP} = MRBRT(z = Exp_{OAP} + Exp_{HAP})/MRBRT(z = TMREL)$$

A population level RR and PAF for all particulate matter exposure were then calculated.

$$RR_{PM} = RR_{OAP}(1 - P_{HAP}) + RR_{HAP}P_{HAP}$$

$$PAF_{PM} = \frac{RR_{PM} - 1}{RR_{PM}}$$

The grid-cell level particulate matter PAFs were population weighted to get a country level PAF, and finally split this PAF based on the average exposure to each OAP and HAP.

$PAF_{OAP} = \frac{Exp_{OAP}}{Exp_{OAP}+P_{HAP}*Exp_{HAP}} PAF_{PM}$, and $PAF_{HAP} = \frac{P_{HAP}*Exp_{HAP}}{Exp_{OAP}+P_{HAP}*Exp_{HAP}} PAF_{PM}$.

With this strategy, $PAF_{PM} = PAF_{HAP} + PAF_{OAP}$, and no burden is counted twice.

References

1. Turner MC, Jerrett M, Pope CA 3rd, Krewski D, Gapstur SM, Diver WR, Beckerman BS, Marshall JD, Su J, Crouse DL, Burnett RT. Long-term ozone exposure and mortality in a large prospective study. *Am J Respir Crit Care Med*. 2016; 193: 1134-42.
2. Hammer MS, van Donkelaar A, Martin R, Li C, Lyapustin A, Sayer AM, Hsu NY, Levy RC, Garay MJ, Kalashnikova OV, Kahn RA, Brauer M, Apte J, Henze DK, Zhang L, Zhang Q. Improved Global Estimates of Fine Particulate Matter Concentrations and Trends Derived from Updated Satellite Retrievals, Modeling Advances, and Additional Ground-Based Monitors. *AGUFM*. 2019; GH21B-1208.
3. Van Donkelaar A, Martin RV, Brauer M, Hsu NC, Kahn RA, Levy RC, Lyapustin A, Sayer AM, Winker DM Global Estimates of Fine Particulate Matter using a Combined Geophysical-Statistical Method with Information from Satellites, Models, and Monitors. *Environ. Sci. Technol*. 2016; 50: 3762–3772.
4. Rue H, Martino S, Chopin N. Approximate Bayesian inference for latent Gaussian models by using integrated nested Laplace approximations. Journal of the royal statistical society: Series b (statistical methodology). 2009; 71: 319-92.
5. Thomas ML, Shaddick G, Simpson D, de Hoogh K, Zidek JV. Spatio-temporal downscaling for continental–scale estimation of air pollution concentrations. arXiv preprint arXiv:1907.00093 (also been Submitted to the Journal of the Royal Statistical Society: Series C (Applied Statistics).
6. Wood SN. Generalized additive models: an introduction with R. Chapman and Hall/CRC. 2017. ISBN 9781498728331
7. Lind L, Sundström J, Ärnlöv J, Lampa E. Impact of Aging on the Strength of Cardiovascular Risk Factors: A Longitudinal Study Over 40 Years. *J Am Heart Assoc*. 2018; 7: e007061.

F2.2 Household air pollution

Exposure to HAP from solid fuels is defined as the proportion of households using solid cooking fuels. The definition of solid fuel in this analysis includes coal, wood, charcoal, dung, and agricultural residues.

In the GBD analysis, exposure to household air pollution is associated with increased risk for stroke and meningitis among the neurological disorders. For the purpose of attributing disease burden to household air pollution, TMREL was defined as a uniform distribution between 2.4 and 5.9 ug/m^3. To include the uncertainty in the TMREL, a random draw from the uniform distribution of the interval between 2.4 and 5.9 μg/m^3 each time the population attributable burden was calculated were taken.

The steps in the estimation of disease burden attributable to household air pollution are shown in the following flowchart:

Household Air Pollution from Solid Fuels

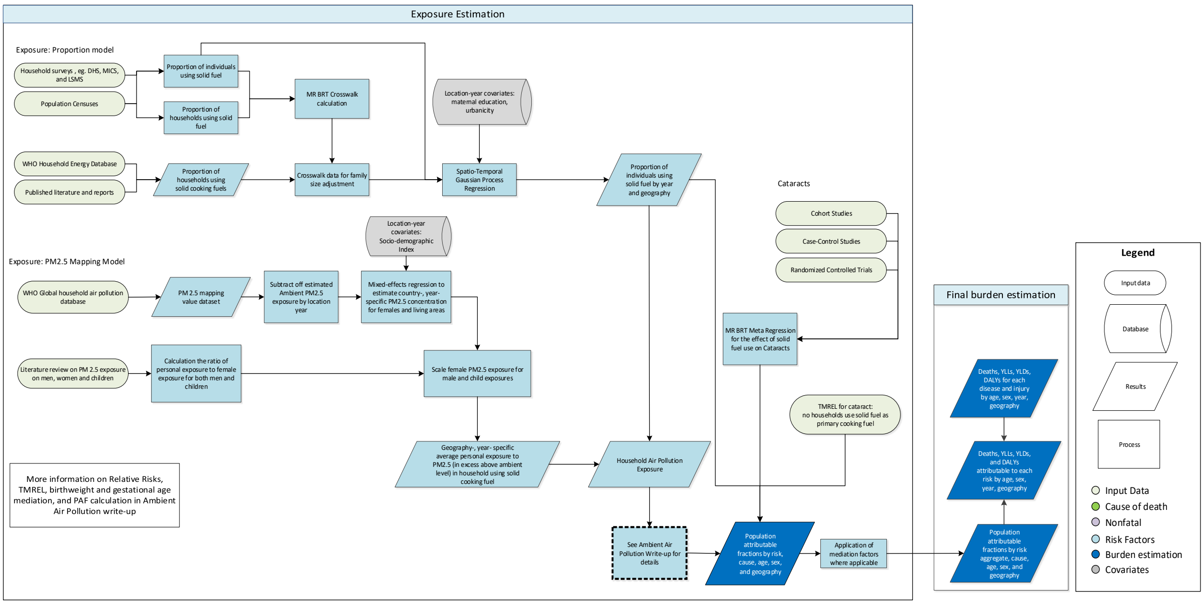

Data

There are many data sources on HAP from solid fuel use in India which include national health surveys such as the National Family Health Survey and the District Level Household Survey, nationwide surveys of the National Sample Survey Organisation, and the Census of India, as well as other published and unpublished epidemiological studies.

Globally, data were extracted from the standard multi-country survey series such as Demographic and Health Surveys, Living Standards Measurement Surveys, Multiple Indicator Cluster Surveys, and World Health Surveys, as well as census country-specific survey series. To fill the gaps of data in surveys and censuses, GBD also downloaded and updated HAP estimates from WHO Energy Database and extracted from literature through systematic review. Each nationally or sub-nationally representative data point provided an estimate for the percentage of households using solid cooking fuels. To crosswalk these estimates, whenever we had the available information, we extracted fuel use at both the individual and household levels. Estimates for the usage of solid fuels for non-cooking purpose were excluded, i.e. heating and primary fuels for lighting.

Modelling strategy

HAP was modelled at individual level using a three-step modelling strategy that uses linear regression, spatiotemporal regression and Gaussian Process Regression. The first step was a mixed-effect linear regression of logit-transformed proportion of households using solid cooking fuels as shown below. The linear model contains maternal education, proportion of population living in urban areas as covariates and has nested random effect by GBD region, and GBD super region respectively.

$$logit(proportion) \sim maternal\ education + urbanicity + (1|region) + (1|super - region)$$

The RRs for stroke and meningitis were jointly calculated with ambient $PM_{2.5}$ air pollution. In order to use the particulate matter risk curves curve, the exposure to particulate matter with diameter of less than 2.5 micrometres ($PM_{2.5}$) must be estimated. A mapping model relying on a database of 75 studies which measures $PM_{2.5}$ exposure in households using solid cooking fuel was utilised. Using socio-demographic index and study-level factors as covariates, the exposure was predicted for all location-years. Joint-estimation PAF approach are described above for ambient particulate matter was used to get a country level PAF and split this PAF based on the average exposure to each OAP and HAP.

References

1. Smith KR, Bruce N, Balakrishnan K, Adair-Rohani H, Balmes J, Chafe Z, et al. Millions dead: how do we know and what does it mean? methods used in the comparative risk assessment of household air pollution. *Annu Rev Public Health*. 2014; 35:185–206.
2. Shupler M, Balakrishnan K, Ghosh S, et al. Global household air pollution database: Kitchen concentrations and personal exposures of particulate matter and carbon monoxide. Data in Brief 2018; 21: 1292–5.
3. Shupler M, Godwin W, Frostad J, Gustafson P, Arku RE, Brauer M. Global estimation of exposure to fine particulate matter (PM2.5) from household air pollution. *Environment International*. 2018; 120: 354–63.
4. Tanchangya J, Geater AF. Use of traditional cooking fuels and the risk of young adult cataract in rural Bangladesh: a hospital-based case-control study. *BMC Ophthalmology*. 2011; 11.

F3. Dietary risks

Dietary risks in GBD comprise of ten components that are protective which include fruits, nuts and seeds, seafood omega-3 fatty acids, vegetables, fibre, whole grains, legumes, polyunsaturated fatty acids, calcium, and milk intake; and five components that are harmful which include sodium, trans-fatty acids, processed meat, sugar sweetened beverages, and red meat intake.

In the GBD analysis, exposure to harmful dietary risk is associated with stroke among neurological disoirders. For the purpose of attributing disease burden for harmful dietary risks other than sodium, TMREL was set to zero. For protective dietary risk factors, the level of intake associated with the lowest risk of mortality from each disease endpoint was calculated based on the 85th percentile of intake across all epidemiological studies included in the meta-analysis of the risk-outcome pair. Then the TMREL was calculated as the weighted average of these numbers using the global number of deaths from each outcome

as the weight. The definition of the dietary risks associated with stroke and their TMREL values are given in the following table.

Risk	Definition	TMREL range
Diet low in fruit	Average daily consumption (in grams per day) of fresh, frozen, cooked, canned, or dried fruit, excluding fruit juices and salted or pickled fruits	310-340 g/day
Diet low in vegetables	Average daily consumption (in grams per day) of fresh, frozen, cooked, canned, or dried vegetables and excluding legumes and salted or pickled vegetables, juices, nuts and seeds, and starchy vegetables such as potatoes or corn	280-320 g/day
Diet low in whole grains	Average daily consumption (in grams per day) of bran, germ, and endosperm in their natural proportion from breakfast cereals, bread, rice, pasta, biscuits, muffins, tortillas, pancakes, and other sources	140-160 g/day
Diet low in fibre	Average daily consumption (in grams per day) of fibre from all sources including fruits, vegetables, grains, legumes, and pulses	21-22 g/day
Diet high in red meat	Any intake (in grams per day) of red meat including beef, pork, lamb, and goat but excluding poultry, fish, eggs, and all processed meats	0 g/day
Diet high in sodium	Average 24-hour urinary sodium excretion (in grams per day)	1-5 g/day

To include the uncertainty in the TMREL, a random draw was taken from the uniform distribution of the interval between the ranges as given in the table above for each dietary risks, each time the population attributable burden was calculated.

The steps in the estimation of disease burden attributable to dietary risks are shown in the following flowchart:

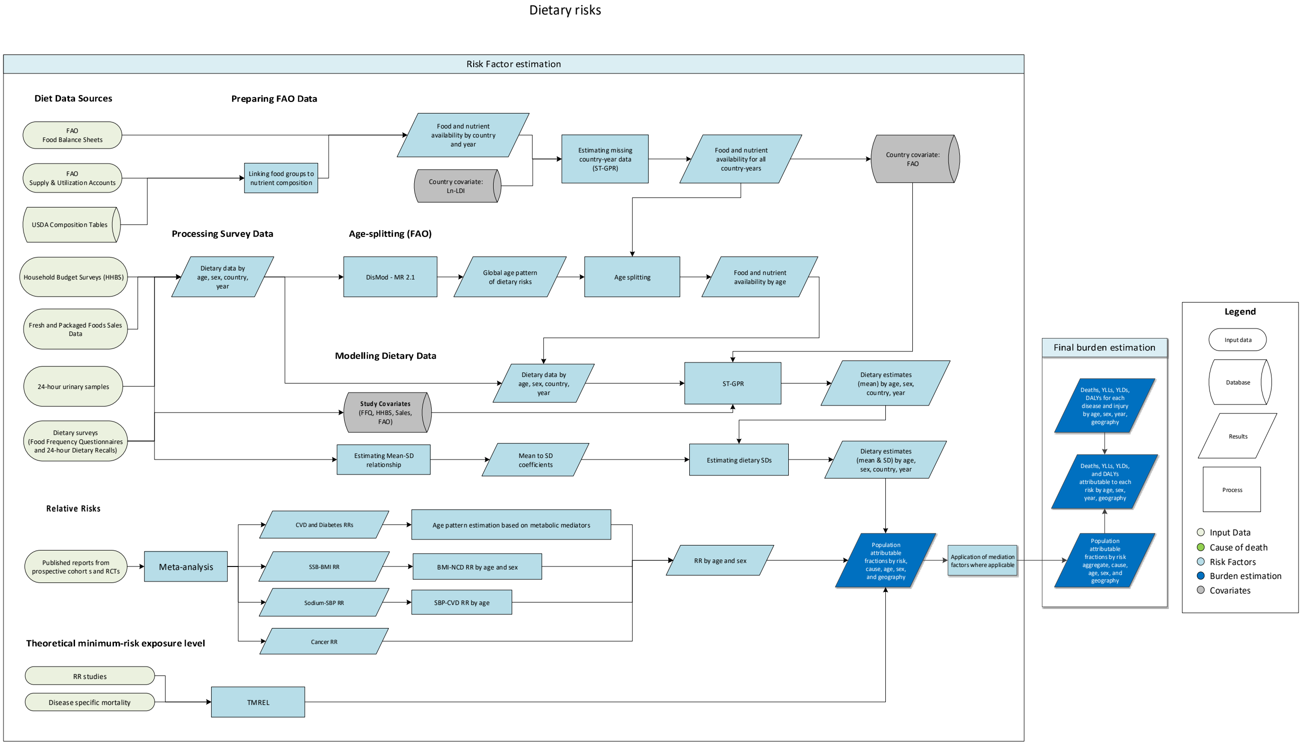

Data

The dietary data that were used in the models comes from multiple sources, including nationally and sub-nationally representative nutrition surveys, household budget surveys, accounts of national sales, and United Nations FAO Supply and Utilization Accounts (SUA). In addition to this, new dietary recall sources were included from a literature search of PubMed and new sources from the yearly known survey series updates. A new systematic review for sodium was also conducted. To more accurately characterise the national availability of various food groups, more disaggregated data on food commodities was used, that were include in FAO SUA and recreated the national availability of each food group based on the GBD definition of the food group.

Missing country-year data was modelled from FAO using a spatiotemporal Gaussian process regression and lag-distributed country income as the covariate. For nutrient availability, the data from Global Nutrient Database was used.[1] For each dietary factor, the global age pattern of consumption based on nutrition surveys (ie, 24-hour diet recall) was estimated and applied that age pattern to the all-age data (availability, sales and household budget surveys) before the data source bias adjustment.

For all dietary risks other than sodium, GBD considers a 24-hour dietary recall as the gold standard, and cross-walked other methods of assessment to the gold standard definition. For sodium, the 24-hour urinary sodium was considered as the gold standard. To estimate the 24-hour urinary sodium based on dietary sodium, a crosswalk adjustment was performed between these two types of data. The bias adjustment factors for non-gold standard data points were determined using MR-BRT (a network meta-regression).

Modelling strategy

ST-GPR framework was used to estimate the mean intake of each dietary factor by age, sex, country, and year. In this analysis, lag-distributed income was removed as a covariate from the models and country-level energy availability was added.

To characterise the distribution of each dietary factor at the population level, ensemble approach was used to separately fit 12 distributions for individual-level microdata to specific to each data source's sampled population. The respective goodness of fit of each family was assessed, and a weighting scheme was determined to optimise overall fit to the unique distribution of each risk factor. A global mean of the weights for each risk factor's data sources was created. Then the standard deviation of each population's consumption was determined through a linear regression that captured the relationship between the standard deviation and mean of intake in nationally representative nutrition surveys using 24-hour diet recalls:

$$ln\ (Standard\ deviation) = \beta_0 + \beta_1 \times ln\ (Mean_i)$$

Then the coefficients of this regression were applied to the outputs of ST-GPR model to calculate the standard deviation of intake by age, sex, year, and country. Within-person variation was also quantified the in consumption of each dietary component and adjusted the standard deviations accordingly.

The most recent epidemiological evidence assessing the relationship between each dietary risk factor and stroke was used in the relative risk analysis. Additionally, based on the most recent epidemiological evidence and the newly developed methods for characterising the risk curve, the dose-response curve of relative risks for all dietary risks was updated. The effect of diet high in sodium on stroke was estimated based on its effect on systolic blood pressure.

The effects of dietary risks on stroke is mediated through high systolic blood pressure, cholesterol, and fasting plasma glucose. To incorporate the age trend in the relative risks for stroke diseases due to metabolic risk factors, the median age-at-event across all cohorts was identified which was considered as the reference age group. The newly estimated risk curves were then assigned to this reference age group. The percentage change in relative risks was derived between each age group and the reference age group by averaging percentage changes in relative risks of all metabolic mediators. Estimates of exposure to dietary risks and relative risks for stroke were then used to calculate the population attributable fractions for stroke attributable to dietary risks.

Reference

1. Schmidhuber, Josef, et al. The Global Nutrient Database: Availability of Macronutrients and Micronutrients in 195 Countries from 1980 to 2013. *Lancet Planetary Health*. 2018, 2: 8.

F4. High fasting plasma glucose

In the GBD analysis, exposure to high fasting plasma glucose (FPG) is associated with the increased risk for stroke and Alzheimer's disease and other dementias among the neurological disorders. For the purpose of attributing disease burden to high FPG, the TMREL was estimated in the range of for FPG is 4.5-5.4 mmol/L. This was calculated by taking the person-year weighted average of the levels of fasting plasma glucose that were associated with the lowest risk of mortality in the pooled analyses of prospective cohort studies.[1]

The steps in the estimation of high fasting plasma glucose are shown in the following flowchart:

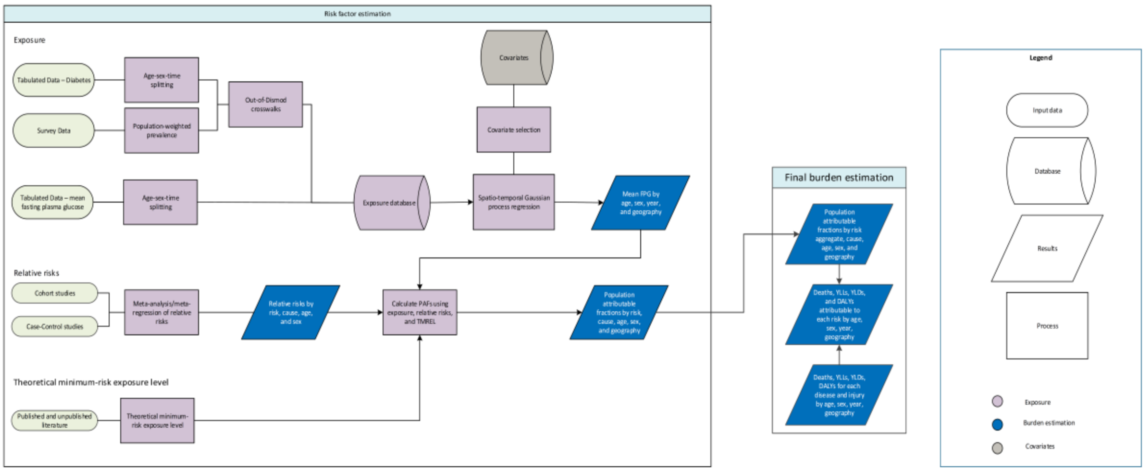

Data

The data inputs were derived from estimates of mean FPG in a representative population, individual-level data of fasting plasma glucose measured from surveys, and estimates of diabetes prevalence in a representative population. Data sources that did not report mean FPG or prevalence of diabetes were excluded from analysis. When a study reported both mean FPG and prevalence of diabetes, the mean FPG for exposure estimates were used. Where possible, individual-level data superseded any data described in a study. Individual-level data were aggregated to produce estimates for each 5-year age group, sex, location, and year of a survey.

Several processing steps were performed to the data in order to address sampling and measurement inconsistencies to ensure comparability across data.

1. Small sample size: Estimates in a sex and age group with a sample size <30 persons is considered a small sample size. In order to avoid small sample size problems that may bias estimates, data are collapsed into the next age group in the same study till the sample size reach at least 30 persons. The intent of collapsing the data is to preserve as much granularity between age groups as possible. If the entire study sample consists of <30 persons and did not include a population-weight, the study is excluded from the modelling process.
2. Crosswalks: Mean FPG from diabetes prevalence were predicted using an ensemble distribution. The distribution of FPG was characterized using individual-level data. Before predicting mean FPG from prevalence of diabetes, we ensured that the prevalence of diabetes was based on the reference case definition FPG >126 mg/dL (7 mmol/L) or on treatment.

Modelling strategy

Exposure estimates are produced for every year between 1980 to 2019 for each national and subnational location, sex, and for each 5-year age group starting from 25 years. A ST-GPR framework was used to model the mean fasting plasma glucose at the location-, year-, age-, and sex-level.

FPG is frequently tested or reported in surveys aiming at assessing the prevalence of diabetes mellitus. In these surveys, the case definition of diabetes may include both a glucose test and questions about treatment for diabetes. People with positive history of diabetes treatment may be excluded from the FPG test. Thus, the mean FPG in these surveys would not represent the mean FPG in the entire population. In this event, the prevalence of diabetes was estimated assuming a definition of FPG>126 mg/dL (7mmol/L), then

crosswalked it to our reference case definition, and then predicted mean FPG.

To inform our estimates in data-sparse countries, a range of covariates were systematically tested, and selected age specific prevalence of obesity as a covariate based on direction of the coefficient and significance level.

Mean FPG is estimated using a mixed-effects linear regression, run separately by sex:

$$\mathrm{logit}(\mathrm{FPG}_{c,a,t}) = \beta_0 + \beta_1 p_{\mathrm{overweight}_{c,a,t}} + \sum_{k=2}^{16} \beta_k I_{A[a]} + \alpha_s + \alpha_r + \alpha_c + \epsilon_{c,a,t}$$

where $p_{\mathrm{overweight}_{c,a,t}}$ is the prevalence of overweight, $I_{A[a]}$ is an indicator variable for a fixed effect on a given 5-year age group, and α_s α_r α_c are random effects at the super-region, region, and country level, respectively. The estimates were then propagated through the ST-GPR framework to obtain 1000 draws for each location, year, age, and sex.

Relative risks for stroke and Alzheimer's disease and other dementias were obtained from dose-response meta-analysis of prospective cohort studies. For stroke, age-specific RRs were estimated using DisMod-MR 2.1 with log (RR) as the dependent variable and median age at event as the independent variable with an intercept at age 110. Estimates of exposure to high FPG and relative risks for stroke and Alzheimer's disease and other dementias were then used to calculate the population attributable fractions for each of these disease attributable to high FPG.

Reference

1. Singh GM, Danaei G, Farzadfar F, et al. The age-specific quantitative effects of metabolic risk factors on cardiovascular diseases and diabetes: a pooled analysis. *PloS One* 2013; 8: e65174.

F5. High body-mass index

In the GBD analysis, exposure to high-body-mass index (BMI) is associated with increased risk for stroke and Alzheimer's disease and other dementias among the neurological disorders. For the purpose of attributing disease burden to high BMI, TMREL exposure level for BMI in adults (ages 20+) was estimated in the range of 20 to 25 kg/m^2 was determined based on the BMI level that was associated with the lowest risk of all-cause mortality in prospective cohort studies The risk-outcome pairs to attribute burden of stroke and Alzheimer's disease and other dementias to high BMI were defined based on the strength of available evidence supporting a causal effect of BMI in meta-analysis. To include the uncertainty in the TMREL, a random draw was taken from the uniform distribution of the interval between 20 and 25 kg/m^2 each time the population attributable burden was calculated.

The steps in the estimation of high body-mass index are shown in the following flowchart:

Adult (Ages 20+) High Body-Mass Index: Data and Model Flow Chart

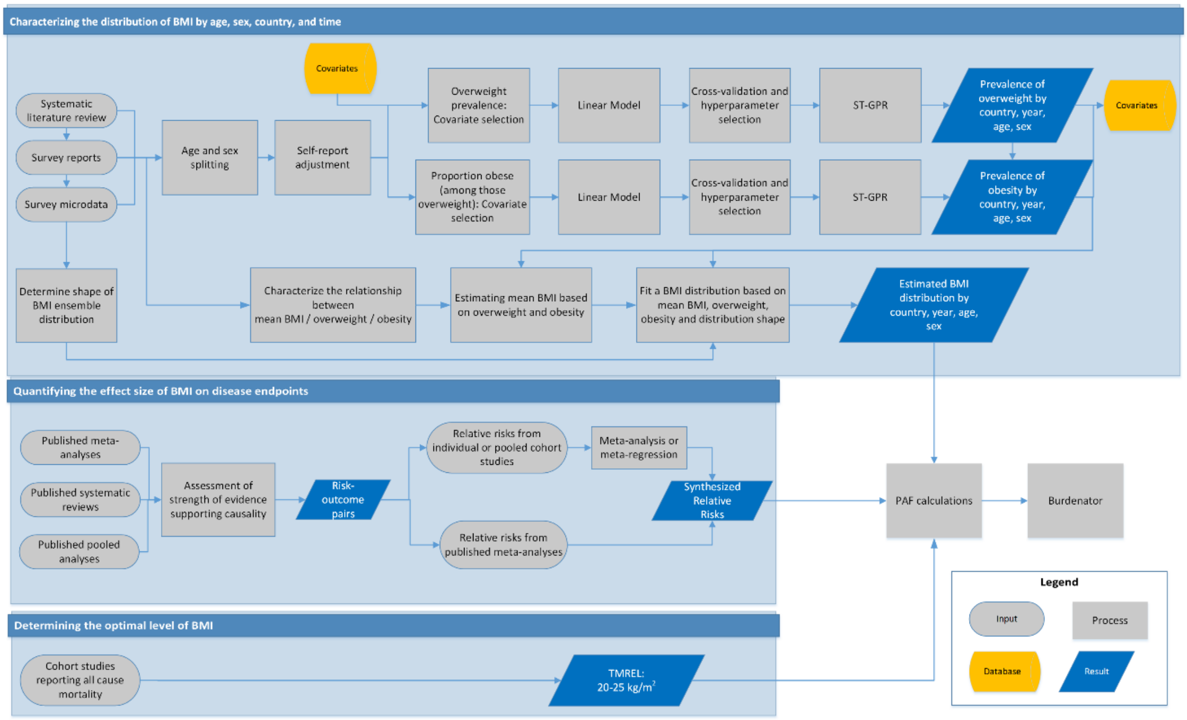

Data

A systematic review was conducted to identify studies providing nationally or sub-nationally representative estimates of overweight prevalence, obesity prevalence, or mean BMI. New data were also added from update of known survey series. Representative studies providing data on mean BMI or prevalence of overweight or obesity were included. For adults, studies were included if they defined overweight as BMI≥25 kg/m^2 and obesity as BMI≥30 kg/m^2, or if estimates using those cutoffs could be back-calculated from reported categories. Studies were excluded if using non-random samples (eg, case-control studies or convenience samples); conducted among specific subpopulations (eg, pregnant women, racial or ethnic minorities, immigrants, or individuals with specific diseases); using alternative methods to assess adiposity (eg, waist-circumference, skin-fold thickness, or hydro densitometry); having sample sizes of less than 20 per age-sex group; or provided inadequate information on any of the inclusion criteria.

Where individual-level survey data were available, mean BMI was computed using weight and height and then the BMI was to determine the prevalence of overweight and obesity. For individuals aged over 19 years, we considered them to be overweight if their BMI was greater than or equal to 25 kg/m^2, and obese if their BMI was greater than or equal to 30 kg/m^2. When only age in years was available, the cut-off for the midpoint of that year was used. Individuals who were obese were also considered to be overweight. At the individual level, observations with BMI<10 kg/m^2 and BMI>70 kg/m^2 were excluded as they were considered to be biologically implausible.

From report and literature, data were extracted on mean BMI, prevalence of overweight, and prevalence of obesity, measures of uncertainty for each, and sample size by the most granular age and sex groups available. Additionally, the same study-level covariates were extracted from microdata (measurement, urbanicity, and representativeness), as well as location and year.

Any report or literature data provided in age groups wider than the standard 5-year age groups or as both sexes combined were split by age-sex using the approach used by Ng and colleagues.[2] The split patterns were identified using sources with data on multiple age-sex groups and these patterns were applied to split aggregated report and literature data. Uncertainty in the age-sex split was propagated by multiplying the standard error of the data by the square root of the number of splits performed.

Both measured and self-reported data were used. Bias in self-report data were tested compared to measured data, which is considered to be the gold standard. For individuals ages 15 and above, self-reported was data adjusted for overweight prevalence and obesity prevalence using MR-BRT models. For both overweight and obesity, sex-specific MR-BRT models were fit on the logit difference between measured and self-reported with a fixed effect on super-region.

Modelling strategy

After adjusting for self-report bias and splitting aggregated data into 5-year age-sex groups, we used ST-GPR to estimate the prevalence of overweight and obesity. The linear model, which when added to the smoothed residuals forms the mean prior for GPR is as follows:

$$\text{logit(overweight)}_{c,a,t} = \beta_0 + \beta_1 \text{energy}_{c,t} + \beta_2 \text{SDI}_{c,t} + \beta_3 \text{vehicles}_{c,t} + \beta_4 \text{agriculture}_{c,t} + \sum_{k=5}^{21} \beta_k I_{A[a]} + \alpha_s + \alpha_r + \alpha_c$$

$$\text{logit(obesity/overweight)}_{c,a,t} = \beta_0 + \beta_1 \text{energy}_{c,t} + \beta_2 \text{SDI}_{c,t} + \beta_3 \text{vehicles}_{c,t} + \sum_{k=4}^{21} \beta_k I_{A[a]} + \alpha_s + \alpha_r + \alpha_c$$

Where energy is ten-year lag-distributed energy consumption per capita, SDI is a composite index of development including lag-distributed income per capita, education, and fertility, vehicle is the number of two or four-wheel vehicles per capita, and agriculture is the proportion of the population working in agriculture. $I_{A[a]}$ is a dummy variable indicating specific age group A that the prevalence point captures, and α_s, α_r, and α_c are super region, region, and country random intercepts, respectively. Random effects were used in model fitting but were not used in prediction.

All combinations of the following covariates were tested to see which performed best in terms of in-sample AIC for the overweight linear model and the obesity as a proportion of overweight linear model: ten-year lag distributed energy per capita, proportion of the population living in urban areas, SDI, lag-distributed income per capita, educational attainment (years) per capita, proportion of the population working in agriculture, grams of sugar adjusted for energy per capita, grams of sugar not adjusted for energy per capita, and the number of two or four-wheeled vehicles per capita. These candidate covariates were selected based on theory as well as reviewing covariates used in other publications. The final linear model was selected based on: 1) if the direction of covariates matched what is expected from theory, 2) all the included covariates were significant, and 3) minimizing in-sample AIC. The covariate selection process was performed using the dredge package in R.

The new version of ST-GPR incorporates information about data density into the process for smoothing over space and time. Estimates in areas or years with few observations have more weight on regional observations. To specify the distribution of time weights and space weights, values of lambda=0.2 and zeta=0.05 were used, respectively. GBD used a value of omega=1.0 for the distribution of age weights. The GPR scale parameter to set to 20, and used the default global cutoff setting for amplitude.

To estimate the mean BMI for adults in each country, age, sex, and time period 1980-2017, the following nested hierarchical mixed-effects model was used, which fit restricted maximum likelihood on data from sources containing estimates of all three indicators (prevalence of overweight, prevalence of obesity, and mean BMI), in order to characterise the relationship between overweight, obesity, and mean BMI:

$$\log(\text{BMI}_{c,a,s,t}) = \beta_0 + \beta_1 \text{ow}_{c,a,s,t} + \beta_2 \text{ob}_{c,a,s,t} + \beta_3 \text{sex} + \sum_{k=4}^{20} \beta_k I_{A[a]} + \alpha_s(1 + \text{ow}_{c,a,s,t} + \text{ob}_{c,a,s,t}) + \alpha_r(1 + \text{ow}_{c,a,s,t} + \text{ob}_{c,a,s,t}) + \alpha_c(1 + \text{ow}_{c,a,s,t} + \text{ob}_{c,a,s,t}) + \epsilon_{c,a,s,t}$$

where $ow_{c,a,s,t}$ is the prevalence of overweight in country c, age a, sex s, and year t, $ob_{c,a,s,t}$is the prevalence of obesity in country c, age a, sex s, and year t, sex is a fixed effect on sex, $I_{A[a]}$ is an indicator variable for age, and α_s, α_r and α are random effects at the super-region, region, and country, respectively.

1,000 draws of the regression coefficients were applied to the 1,000 draws of overweight prevalence and obesity prevalence produced through ST-GPR to estimate 1,000 draws of mean BMI for each country, year, age, and sex. This approach ensured that overweight prevalence, obesity prevalence, and mean BMI were correlated at the draw level and uncertainty was propagated.

Ensemble distribution approach was used to fit ensemble weights by source and sex, with source and sex specific weights averaged across all sources included to produce the final global weights. The ensemble weights were fit on measured microdata. One thousand draws of BMI distributions for each location, year, age group, and sex estimated were produced by fitting an ensemble distribution using 1,000 draws of estimated mean BMI, 1,000 draws of estimated standard deviation, and the ensemble weights. Estimated standard deviation was produced by optimizing a standard deviation to fit estimated overweight prevalence draws and estimated obesity prevalence draws.

Risk-outcome pairs were based on the strength of available evidence supporting a causal effect. The relative risk per 5-unit change in BMI for stroke and Alzheimer's disease and other dementias endpoint was obtained from meta-analyses, and where available, pooled analyses of prospective observational studies. In cases where a relative risk per 5-unit change in BMI was not available, the dose-response meta-analysis was conducted using two-step generalised least squares for time trends estimation methods. Estimates of exposure to high BMI and relative risks for stroke and Alzheimer's disease and other dementias were then used to calculate the population attributable fractions for each of these disease attributable to high BMI.

References

1. Angelantonio ED, Bhupathiraju SN, Wormser D, et al. Body-mass index and all-cause mortality: individual-participant-data meta-analysis of 239 prospective studies in four continents. *Lancet* 2016; 388: 776–86.
2. Ng M, Fleming T, Robinson M, et al. Global, regional, and national prevalence of overweight and obesity in children and adults during 1980–2013: a systematic analysis for the Global Burden of Disease Study 2013. *Lancet* 2014; 384: 766–81.

F6. Tobacco use

Tobacco use in GBD consists mainly of smoking, secondhand smoke and chewing tobacco. We describe below the exposure to smoking and secondhand smoke attributable to the neurological disorders.

F6.1 Smoking

In the GBD analysis, exposure to smoking is associated with increased risk for stroke, Alzheimer disease and other dementias, Parkinson disease (protective), and multiple sclerosis among the neurological disorders. For the purpose of attributing disease burden to smoking, the TMREL was all individuals who were lifelong non-smokers, above which there could be adverse health effects.

The steps in the estimation of disease burden attributable to smoking are shown in the following flowchart:

Current and former smoking prevalence

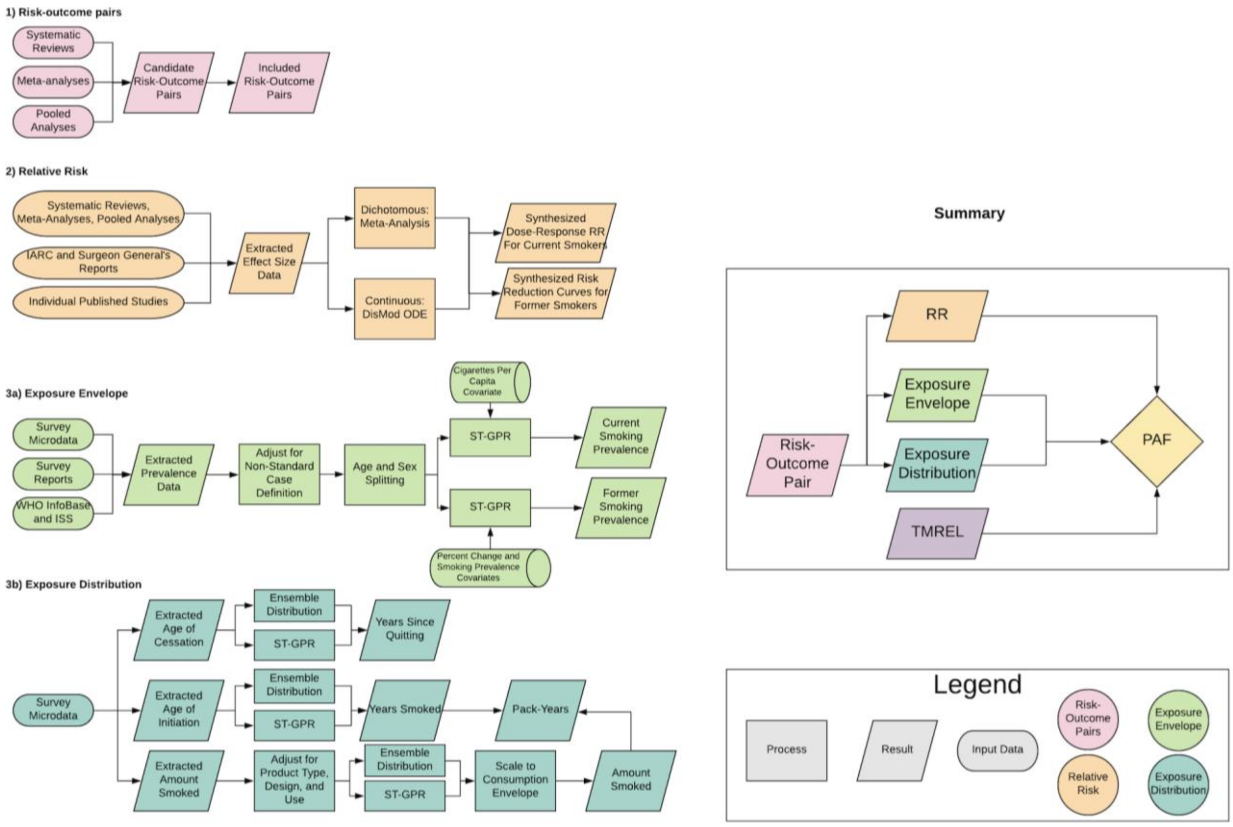

GBD estimates the prevalence of current smoking and the prevalence of former smoking using data from cross-sectional nationally representative household surveys. GBD defines current smokers as individuals who currently use any smoked tobacco product on a daily or occasional basis, and former smokers as individuals who quit using all smoked tobacco products for at least 6 months, where possible, or according to the definition used by the survey. Prior to modelling a complete time series for all demographic groups were made and adjustments for alternative case definitions as well as for data reported in non-standard age or sex groups. Current and former prevalence were modelled using ST-GPR.

Data

Primary data was extracted from individual-level microdata and survey report tabulations. Data on current, former, and/or ever smoked tobacco were extracted if surveys reported any combination of frequency of use (daily, occasional, and unspecified, which includes both daily and occasional smokers) and type of smoked tobacco used (all smoked tobacco, cigarettes, hookah, and other smoked tobacco products such as cigars or pipes resulting in 36 possible combinations. For microdata, relevant demographic information, including age, sex, location, and year, as well as survey metadata, including survey weights, primary sampling units, and strata were extracted. This information allowed us to tabulate individual-level data in the standard GBD

five-year age, sex groups and produce accurate estimates of uncertainty. For survey report tabulations, data at the most granular age-sex group provided were extracted.

Crosswalk: Case definitions for smoking prevalence is current smoking of any tobacco product and former smoking of any tobacco product. All other data points were adjusted to be consistent with either of these definitions. Some sources contained information on more than one case definition and these sources were used to develop the adjustment coefficient to transform alternative case definitions to our case definition. The adjustment coefficient was the beta value derived from a linear model with one predictor and no intercept.

Age and sex splitting: Data reported was split in broader age groups than the GBD 5-year age groups or as both sexes combined by adapting the method reported in Ng et al[1] to split using a sex-geography-time-specific reference age pattern. The data was separated into two sets: a training dataset, with data already falling into GBD sex-specific 5-year age groups, and a split dataset, which reported data in aggregated age or sex groups. ST-GPR was then used to estimate sex-geography-time-specific age patterns using data in the training dataset. The estimated age patterns were used to split each source in the split dataset.

ST-GPR model for estimating the age patterns for age-sex splitting used an age weight parameter value that minimizes the effect of any age smoothing. This parameter choice allowed the estimated age pattern to be driven by data, rather than being enforced by any smoothing parameters of the model. Because these age-sex split data points were to be incorporated in the final ST-GPR exposure model, a modelled age pattern for a given sex-location-year were not doubly enforce on a given aggregate data point.

Modelling strategy

Smoking prevalence modelling

ST-GPR was used to model current and former smoking prevalence. The mean function input to GPR is a complete time series of estimates generated from a mixed effects hierarchical linear model plus weighted residuals smoothed across time, space, and age. The linear model formula for current smoking, fit separately by sex using restricted maximum likelihood in R, is:

$$logit(p_{g,a,t}) = \beta_0 + \beta_1 CPC_{g,t} + \sum_{k=2}^{19} \beta_k I_{A[a]} + \alpha_s + \alpha_r + \alpha_g + \epsilon_{g,a,t}$$

Where $CPC_{g,t}$ is the tobacco consumption covariate by geography g and time t, $I_{A[a]}$ is a dummy variable indicating specific age group A that the prevalence point $P_{g,a,t}$ captures, and α_s, α_r, and α_g are super region, region, and geography random intercepts, respectively. Random effects were used in model fitting but not in prediction.

The linear model formula for former smoking is:

$$logit(p_{g,a,t}) = \beta_0 + \beta_1 PctChange_{A[a],g,t} + \beta_3 CSP_{A[a],g,t} + \sum_{k=3}^{20} \beta_k I_{A[a]} + \alpha_s + \alpha_r + \alpha_g + \epsilon_{g,a,t}$$

Where $PctChange_{A[a],gt}$ is the percent change in current smoking prevalence from the previous year, and $CSP_{A[a],gt}$ is the current smoking prevalence by specific age group A, geography$g$, and time t that point $P_{g,a,t}$ captures, both derived from the current smoking ST-GPR model.

Supply-side estimation

Raw data used for supply-side estimation were domestic supply (USDA Global Surveillance Database and UN FAO) and retail supply (Euromonitor) of tobacco. These data were age-sex split using daily smoking prevalence to generate number of cigarettes per smoker per day for a given location-age-sex-year. A point was included if it was (in cigarettes per smoker): under five (10–14 year olds); under 20 (males, 15–19 year olds); under 18 (females, 15–19 year olds); under 38/35 and over three (males/females, 20+ year olds). The mean tobacco per capita value over a 10-year window was calculated. A point was excluded if it was over 70% of that mean value away from the mean value. The 70% limit was chosen using histograms of these distances. Additionally, some manual outliering was performed to account for edge cases. Finally, data smoothing was performed by taking a three-year rolling mean over each location-year.

Imputation to fill in missing years was performed for all series to remove compositional bias from the final estimates. For this the log ratio of each pair of sources was modelled as a function of an intercept and nested

random effects on super-region, region, and location. The appropriate predicted ratio was multiplied by each source, and then the predictions were averaged to get the final imputed value.
Variance was calculated both across series (within a location-year) as well as across years (within a location-source). Additionally, if a location-year had one imputed point was, the variance was multiplied by 2. If a location-year had two imputed points, the variance was multiplied by 4. The average estimates in each location-year were the input to an ST-GPR model. For this, GBD used a simple mixed effects model, which was modelled in log space with nested location random effects. Subnational estimates were then further modelled by splitting the country-level estimates using current smoking prevalence.

Exposure among current and former smokers

The exposure among current smokers was estimated for two continuous indicators: cigarettes per smoker per day and pack-years. Pack-years incorporates aspects of both duration and amount. One pack-year represents the equivalent of smoking one pack of cigarettes (assuming a 20-cigarette pack) per day for one year. Since the pack-years indicator collapses duration and intensity into a single dimension, one pack-year of exposure can reflect smoking 40 cigarettes per day for six months or smoking 10 cigarettes per day for two years.

To produce these indicators, individual smoking histories were simulated based on distributions of age of initiation and amount smoked. The simulation was informed with cross-sectional survey data capturing these indicators, modelled at the mean level for all locations, years, ages, and sexes using ST-GPR. The estimates of cigarettes per smoker per day were rescaled to an envelope of cigarette consumption based on supply-side data. Pack-years of exposure were estimated by summing samples from age- and time-specific distributions of cigarettes per smoker for a birth cohort in order to capture both age trends and time trends and avoid the common assumption that the amount someone currently smokes is the amount they have smoked since they began smoking. All distributions were age-, sex-, and region-specific ensemble distributions, which were found to outperform any single distribution.

The exposure among former smokers was estimated using years since cessation. ST-GPR was utilised to model mean age of cessation using cross-sectional survey data capturing age of cessation. Using these estimates, ensemble distributions of years was generated since cessation for every location, year, age group, and sex.

Relative risk and population attributable fractions

Evidence supporting a causal relationship suggests that smoking is associated with stroke, Alzheimer disease and other dementias, Parkinson disease (protective), and multiple sclerosis among the neurological disorders. The effect sizes by cigarettes per smoker per day, pack-years, and years since quitting were synthesised from cohort and case-control studies to produce nonlinear dose-response curves using a Bayesian meta-regression model. For outcomes with significant differences in effect size by sex or age, sex- or age-specific risk curves were produced.

GBD estimates risk curves of former smokers compared to never smokers taking into account the rate of risk reduction among former smokers seen in the cohort and case-control studies, and the cumulative exposure among former smokers within each age, sex, location, and year group.

PAFs were estimated based on the following equation:

$$PAF = \frac{p(n) + p(f)\int \exp(x) * rr(x) + p(c)\int \exp(y) * rr(y) - 1}{p(n) + p(f)\int \exp(x) * rr(x) + p(c)\int \exp(y) * rr(y)}$$

Where $p(n)$ is the prevalence of never smokers, $p(f)$ is the prevalence of former smokers, $p(c)$ is the prevalence of current smokers, $\exp(x)$ is a distribution of years since quitting among former smokers, $rr(x)$ is the relative risk for years since quitting, $\exp(y)$ is a distribution of cigarettes per smoker per day or pack-years, and $rr\ (y)$ is the relative risk for cigarettes per smoker per day or pack-years. Cigarettes per smoker per day was used as the exposure definition for neurological outcomes associated with smoking.

Reference

1. Ng M, Freeman MK, Fleming TD, Robinson M, Dwyer-Lindgren L, Thomson B, et al. Smoking Prevalence and Cigarette Consumption in 187 Countries, 1980–2012. *JAMA* 2014; 311 :183–92.

F6.2 Secondhand smoke

GBD defines secondhand smoke exposure as current exposure to secondhand tobacco smoke at home, at work, or in other public places. Household composition was used as a proxy for non-occupational secondhand smoke exposure and make the assumption that all persons living with a daily smoker are exposed to tobacco smoke. Surveys were used to estimate the proportion of individuals exposed to secondhand smoke at work. GBD only considers non-smokers to be exposed to secondhand smoke. Non-smokers are defined as all persons who are not daily smokers. Ex-smokers and occasional smokers are considered non-smokers in this analysis.

In the GBD analysis, exposure to secondhand smoke is associated with increased risk for stroke among the neurological disorders. The TMREL for secondhand smoke is zero exposure among non-smokers, meaning that non-smokers would not live with any primary smokers.

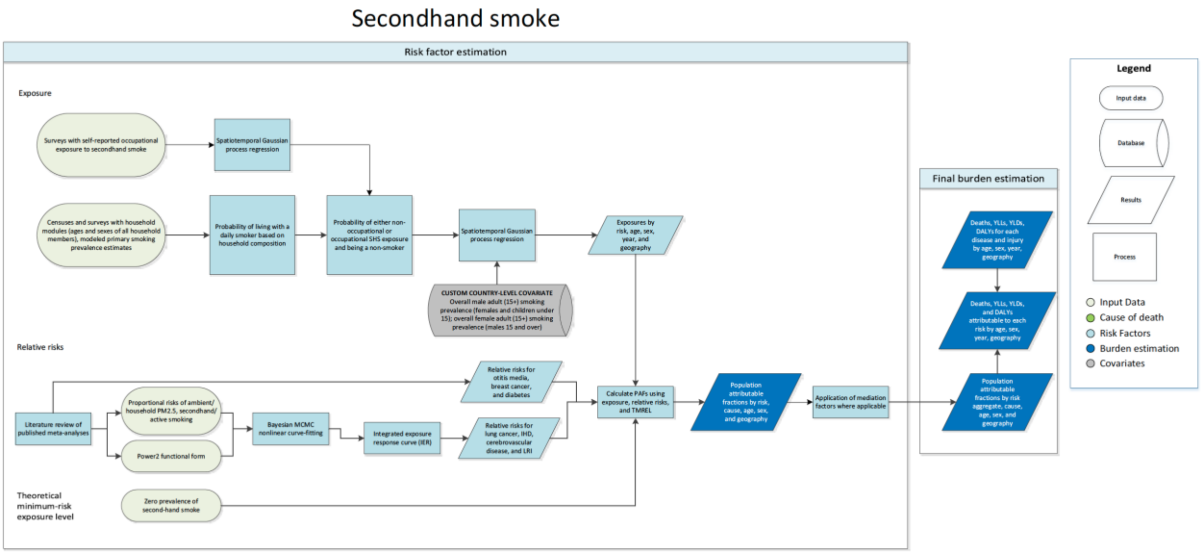

Data

To calculate the proportion of non-smokers who live with at least one smoker, GBD uses unit record data on household composition, which included the ages and sexes of all persons living in the same household. The data sources included representative major survey series with a household composition module, including the Demographic Health Surveys (DHS), the Multiple Indicator Cluster Surveys (MICS), and the Living Standards Measurement Surveys (LSMS); and national and subnational censuses, which included those captured in the IPUMS project and identified using the Global Health Data Exchange catalog.

To calculate the proportion of individuals exposed to secondhand smoke at work, by age and sex, we used cross-sectional surveys that ask respondents about self-reported occupational secondhand smoke exposure. Sources include the Global Adult Tobacco Surveys, Eurobarometer Surveys, and WHO STEPS Surveys.

Modelling strategy

The probability was estimated for each person is living with a smoker and is also a non-smoker themselves using set theory. First, household composition data were used at the individual level to capture the ages and sexes of each person in the household. Second, surveys were analysed with both household composition data and tobacco use questions and determined that the distribution of household size, mean age of the household members, and the age distribution were not significantly different between households with and without a self-reported smoker. Since, household composition did not vary between smokers and non-smokers, primary daily smoking prevalence model was used to calculate the probability that each household member is a daily smoker. Next, the probability of the union of sets on each individual household member was used to calculate the overall probability that at least one of the other household members was a daily smoker.

Occupational exposure was incorporated by modelling prevalence of current exposure to secondhand smoke at work, by age, sex, location, and year, using ST-GPR. In order to avoid double counting the probability was calculated for an individual that is exposed through either non-occupational exposure or occupational

exposure, given their age, sex, and household composition. This probability of exposure was then multiplied by the probability that the individual is not a smoker themselves (ie, 1 minus primary daily smoking prevalence for that person's location, year, age, and sex). These individual-level probabilities were then collapsed to produce average probabilities of exposure by location, year, age, and sex.

These probabilities were modelled in the ST-GPR framework, which generates exposure estimates from a mixed effects hierarchical linear model plus weighted residuals smoothed across time, space, and age. The linear model formula was fit separately by sex using restricted maximum likelihood in R. Sex-specific overall daily smoking prevalence for adults was used as a country-level covariate in the model. The overall male adult daily smoking prevalence was used as the covariate for females of all ages. The overall female adult daily smoking prevalence was used as the covariate for males.

All input data points from the probability calculation had a measure of uncertainty (variance and sample size) coming from the uncertainty of the primary smoking prevalence model and the sample size from the unit record data going into the modelling process. Geographical random effects were used in model fitting but were not used in prediction.

The burden of stroke associated with secondhand smoke was estimated for adults greater than or equal to 25 years. The country-specific relative risks were created using integrated exposure response curves (IER) for $PM_{2.5}$ air pollution. The estimates of exposure to secondhand smoke and relative risks for stroke were then used to calculate the population attributable fractions for stroke attributable to secondhand smoke.

F7. Kidney dysfunction

The kidney dysfunction risk factor exposure is divided into four categories of renal function defined by urinary albumin to creatinine ratio (ACR) and estimated glomerular filtration rate (eGFR):

- Albuminuria with preserved eGFR (ACR >30 mg/g & eGFR >=60 ml/min/1.73m2); this corresponds to stages 1 and 2 chronic kidney disease (CKD) in the Kidney Disease Improving Global Outcomes (KDIGO) classification
- CKD stage 3 (eGFR of 30-59 ml/min/1.73m2);
- CKD stage 4 (eGFR of 15-29 ml/min/1.73m2); and
- CKD stage 5 (eGFR <15ml/min/1.73m2, not (yet) on renal replacement therapy).

In the GBD analysis, the exposure to kidney dysfunction is associated with increased risk for stroke among the neurological disorders. For the purpose of attributing disease burden to kidney dysfunction, the TMREL is ACR 30 mg/g or less and eGFR greater than 60ml/min/1.73m^2. An ACR above 30 mg/g and eGFR below 60ml/min/1.73m2 have been demonstrated in the literature to be the thresholds at which increased cardiovascular events occur secondary to kidney dysfunction.[1-10]

The steps in the estimation of disease burden attributable to kidney dysfunction are shown in the following chart:

Kidney Dysfunction

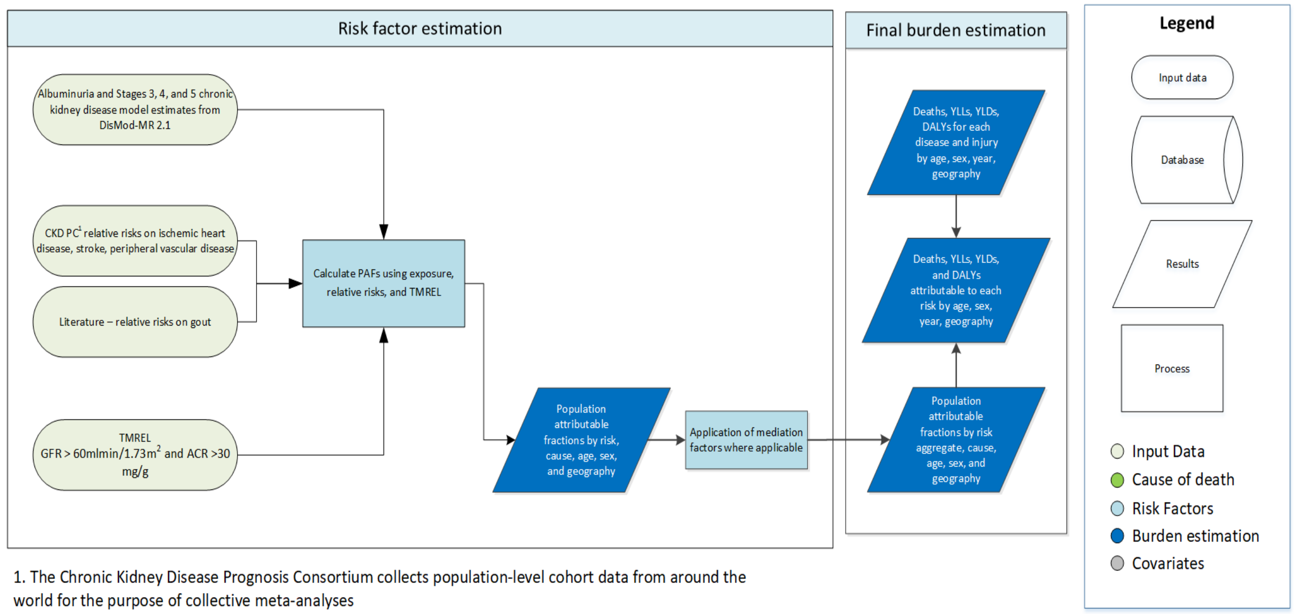

Data

Population based surveys and cohort studies were used to collect the data for kidney dysfunction. Studies were included if it was population representative, reporting albuminuria with preserved GFR (GFR< 60 ml/min/ 1.73m^2).

Modelling strategy

A two-stage pooled meta-analysis was used to calculate relative risks for stroke. The relative risk of stroke was first determined within each cohort, and then a pooled analysis of cohort-level relative risks was performed using a random effects meta-analysis approach. Uncertainty intervals largely overlapped for the relative risks of fatal and nonfatal cardiovascular events from kidney dysfunction. Thus relative risks from the combined analysis for fatal and nonfatal cardiovascular outcomes was used in the relative risk estimation.

Burden attributable to kidney dysfunction was estimated for stroke using a pooled cohort analysis of six cohort studies from the CKD-PC was explored. The data from cohort studies was ran through MR-BRT meta-regression to determine the relationship between age and stroke based on exposure to kidney dysfunction. Estimates were nested within cohorts. A three-degree spline was placed on age with decreasing monotonicity. Relative risk estimates for stroke above age 85 were set equal to the risk at age 85 to control for lack of data in older age groups.

$$PAF = \frac{\sum_{i=1}^{n} P_i \ (RR_i - 1)}{\sum_{i=1}^{n} P_i \, (RR_i - 1) + 1}$$

Some sensitivity analyses were done with and without controlling for blood pressure. This is because kidney dysfunction increases the risk of stroke directly, as well as through blood pressure. Generally, the relative risk of stroke was lower when controlling for blood pressure. This lower risk that controlled for hypertension was chosen for a more conservative estimate. The fatal and nonfatal burden for stroke attributable to the categorical exposure to impaired kidney function was calculated using the following equation:

PAF based on categorical exposure where RRi is the relative risk for exposure level i, Pi is the proportion of the population in that exposure category, and n is the number of exposure categories. Epidemiological evidence for estimating relative risk of stroke due to kidney dysfunction were obtained from the following studies:

Stroke	Chronic Kidney Disease Prognosis Consortium (CKD-PC). Chronic Kidney Disease Prognosis Consortium GBD 2016 Impaired Kidney Function Relative Risk Meta-Analysis.
	National Heart, Lung, and Blood Institute, National Institutes of Health (NIH). United States Atherosclerosis Risk in Communities Study. Bethesda, United States: National Heart, Lung, and Blood Institute, National Institutes of Health (NIH).
	International Diabetes Institute (IDI). Australia Diabetes, Obesity and Lifestyle Study 1999-2000. Melbourne, Australia: International Diabetes Institute (IDI)
	National Heart, Lung, and Blood Institute, National Institutes of Health, University of California, Los Angeles (UCLA), University of Minnesota. United States Multi-Ethnic Study of Atherosclerosis First Examination 2000-2002. Bethesda, United States: National Heart, Lung, and Blood Institute, National Institutes of Health Uppsala University. Sweden Uppsala Longitudinal Study of Adult Men.

References

1. Go AS, Chertow GM, Fan D, McCulloch CE, Hsu CY. Chronic kidney disease and the risks of death, cardiovascular events, and hospitalization. *N Engl J Med* 2004; 351 :1296-305.
2. Ninomiya T, Kiyohara Y, Kubo M, Tanizaki Y, Doi Y, Okubo K, et al. Chronic kidney disease and cardiovascular disease in a general Japanese population: the Hisayama Study. *Kidney international* 2005; 68: 228-36.
3. Shara NM, Wang H, Mete M, Al-Balha YR, Azalddin N, Lee ET, et al. Estimated GFR and incident cardiovascular disease events in American Indians: the Strong Heart Study. *AJKD* 2012; 60: 795-803.
4. Mann JF, Gerstein HC, Pogue J, Bosch J, Yusuf S. Renal insufficiency as a predictor of cardiovascular outcomes and the impact of ramipril: the HOPE randomized trial. *Annals of internal medicine* 2001; 134: 629-36.
5. Chronic Kidney Disease Prognosis C, Matsushita K, van der Velde M, Astor BC, Woodward M, Levey AS, et al. of estimated glomerular filtration rate and albuminuria with all-cause and cardiovascular mortality in general population cohorts: a collaborative meta-analysis. *Lancet* 2010; 375: 2073-81.
6. Association De Graauw J, Chonchol M, Poppert H, Etgen T, Sander D. Relationship between kidney function and risk of asymptomatic peripheral arterial disease in elderly subjects. Nephrology, dialysis, transplantation: official publication of the European Dialysis and Transplant Association-European Renal Association 2011; 26: 927-32.
7. Wattanakit K, Folsom AR, Selvin E, Coresh J, Hirsch AT, Weatherley BD. Kidney function and risk of peripheral arterial disease: results from the Atherosclerosis Risk in Communities (ARIC) Study. *JASN* 2007; 18: 629-36.
8. O'Hare AM, Vittinghoff E, Hsia J, Shlipak MG. Renal insufficiency and the risk of lower extremity peripheral arterial disease: results from the Heart and Estrogen/Progestin Replacement Study (HERS). *JASN* 2004; 15: 1046-51.
9. Manjunath G, Tighiouart H, Coresh J, Macleod B, Salem DN, Griffith JL, et al. Level of kidney function as a risk factor for cardiovascular outcomes in the elderly. *Kidney international* 2003; 63: 1121-9.
10. Manjunath G, Tighiouart H, Ibrahim H, MacLeod B, Salem DN, Griffith JL, et al. Level of kidney function as a risk factor for atherosclerotic cardiovascular outcomes in the community. *Journal of the American College of Cardiology* 2003; 41: 47-55.
11. Miettinen OS. Proportion of disease caused or prevented by a given exposure, trait or intervention. *American Journal of Epidemiology* 1974; 99: 325-32.

F8. Lead exposure

In the GBD analysis, chronic lead exposure, measured as micrograms of lead per gram of bone (µg/g), is associated with increased systolic blood pressure and stroke among the neurological disorders. For the purpose of attributing disease burden to lead exposure, the TMREL was estimated at 2.0 µg/dL. This level was based on ambient sources of lead that would be impossible to eliminate[1] and a review of the literature indicating no consistent statistically significant estimates of increased relative risks at lower levels of blood lead.

The steps in the estimation of lead exposure are shown in the following flowchart:

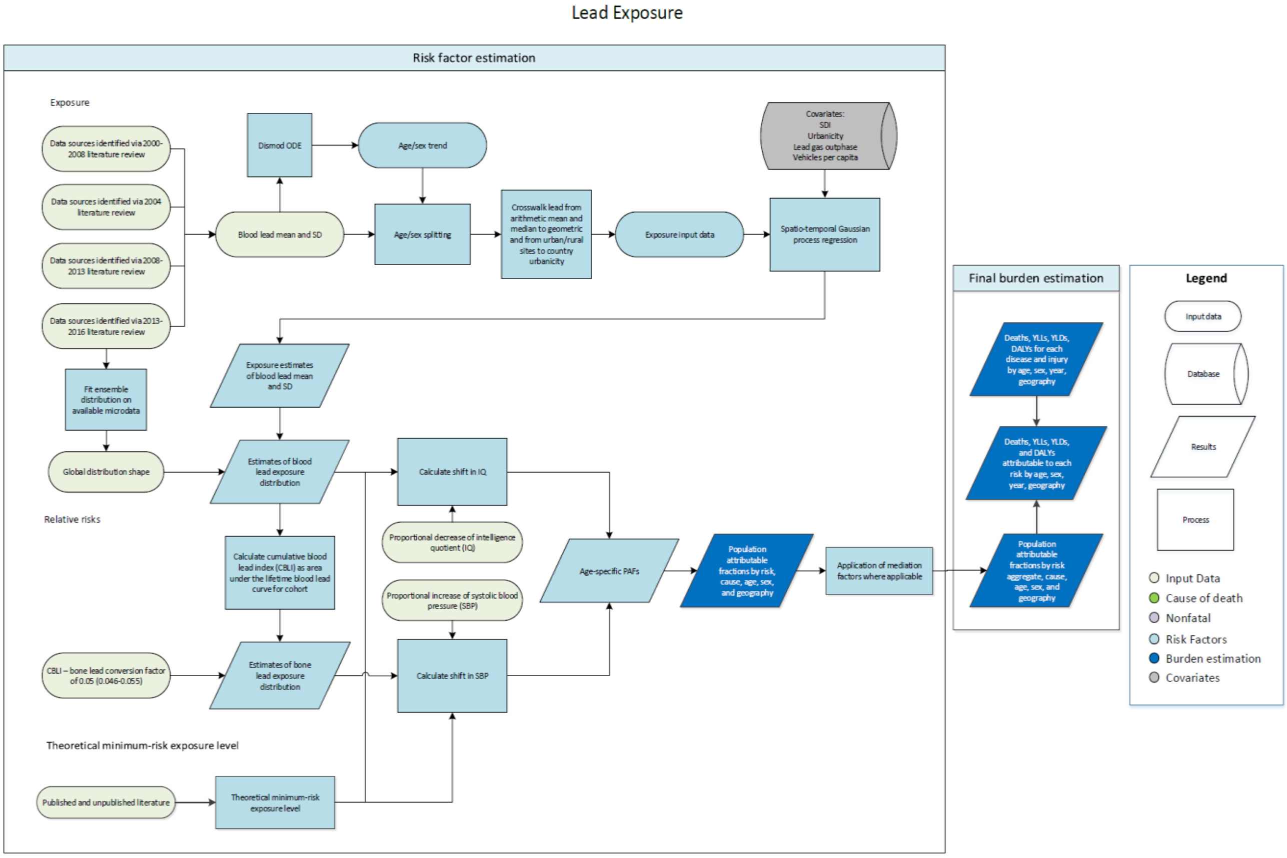

The input data for lead exposure is primarily extracted from literature reports of blood lead levels, in addition to a few blood lead surveys. Blood lead values are derived from studies that take blood samples and analyse them using various techniques to determine the level of lead present. The second pathway of burden, bone lead exposure, was estimated by calculating a cumulative blood lead index for cohorts using estimated blood lead exposure over their lifetime. The cumulative blood lead index is then used to estimate bone lead using a scalar defined in literature.[2]

MR-BRT was used to crosswalk data. Blood lead exposure data are reported in the literature as either an arithmetic mean, a geometric mean, or a median. To standardise the data, all values reported were adjusted as a geometric mean or median to reflect what they would have been had the study reported the arithmetic mean. Additionally, the data come from locations of varying urbanicity (proportion of individuals in a given location living in an urban area). Because the urbanicity of a location was expected to affect the estimates, data was adjusted so that they were equivalent to the average urbanicity of the country from which the data were collected.

Modelling strategy

Lead exposure was modelled using ST-GPR technique. In order to predict blood lead in country-years with insufficient data, covariates that have been produced across time and space relevant to this analysis were used. For blood lead exposure, the covariates determined to have predictive ability were the Socio-demographic Index (SDI), urbanicity, the combined number of two- and four-wheeled vehicles per capita, and a covariate indicating whether leaded gasoline had been phased out in a given country-year (smoothed over the first five years of phase-out to reflect its gradual implementation). ST-GPR was used to produce estimates of mean and standard deviation of blood lead for all age groups, for both sexes, and for all GBD locations from 1970 to 2019. The linear regression equation is shown below.

*Log(data) ~ sdi+urbancity + (leaded gas outphase*vehicles per capita) + (1|level_1)*

SDI = Socio-demographic Index
Urbanicity = proportion of population living in urban areas
Leaded gas outphase = whether or not a country has banned use of leaded gasoline Vehicles per capita = number of 2- and 4-wheeled vehicles per capita
(1|level_1) = super-region-level random effects

Ensemble modelling techniques were used to find an optimal global distribution by fitting a variety of distributions to the available blood lead microdata. This was a common update for all continuous risk factors. The ST-GPR mean and standard deviation estimates for blood lead were used with the global distribution shape to determine distributions for blood lead exposure. Eleven different probability distributions included were: exponential, gamma, inverse-gamma, mirrored gamma, log-logistic, Gumbel, mirrored Gumbel, Weibull, log-normal, normal, and beta. A little over 80% of the final distribution was log-logistic (35%), inverse-gamma (18%), log-normal (16%), or mirrored Gumbel (12%), with the seven other distributions comprising the remaining 20%.

To calculate blood lead over the lifetime of a given cohort, blood lead was assumed to grow linearly from 2.0 µg/dL in 1920 to the value for that cohort in 1970. Using the exposure distributions of blood lead over time and space, cohorts were constructed such that lifetime blood lead could be expressed as a curve over each year of life. The area under this curve was the cumulative blood lead index, which was used to estimate bone lead in a given year with the aforementioned scalar.

Bone lead level is paired with systolic blood pressure, and subsequently to stroke to which systolic blood pressure is paired. The bone lead relative risks were taken from a 2008 meta-analysis that showed a 0.26 mmHg increase in systolic blood pressure (SBP) per 10 µg/g increase in bone lead (95% CI: 0.02 to 0.50).[3] Because bone lead is associated with increases in SBP, the burden of stroke attributable to exposure to bone lead is mediated through SBP. As such, the relative risks for bone lead exposure are all the same as the relative risks that SBP has for stroke. Estimates of exposure to bone lead level and relative risks for stroke were then used to calculate the population attributable fractions for stroke attributable to bone lead level.

References

1. Pruss-Astun A, Fewtrell L, Landrigan PJ, Ayuso-Mateos JL. Lead Exposure. In: Ezzati M, Lopez AD, Rodgers A, Murray CJ, eds. Comparative quantifications of health risks: Global and regional burden of disease attrituable to selected major risk factors. Geneva, World Health Organization, 2004: 1496- 542
2. Hu H, Shih R, Rothenberg S, Schwartz BS. The epidemiology of lead toxicity in adults: measuring dose and consideration of other methodologic issues. *Environ Health Perspect* 2007; 115: 455-62.
3. Navas-Acien A, Schwartz BS, Rothenberg SJ, Hu H, Silbergeld EK, Guallar E. Bone lead levels and blood pressure endpoints: a meta-analysis. *Epidemiology* 2008; 19: 496-504.

F9. High low-density lipoprotein cholesterol level

In the GBD analysis, exposure to high low-density lipoprotein (LDL) cholesterol level is associated with the increased risk for stroke among the neurological disorders. For the purpose of attributing disease burden to high LDL cholesterol level, the TMREL with a uniform distribution between 0.7 and 1.3 mmol/L was used based on the evidence from meta-analysis of randomised trails and studies of PCSK-9 inhibitors that outcomes can be improved even at low levels of LDL-cholesterol, below 1.3 mmol/L[3].[1,2] To include the uncertainty in the TMREL, GBD took a random draw from the uniform distribution of the interval between 0.7 mmol/L and 1.3 mmol/L each time the population attributable burden was calculated.

The steps in the estimation of disease burden attributable to high LDL cholesterol level are shown in the following chart:

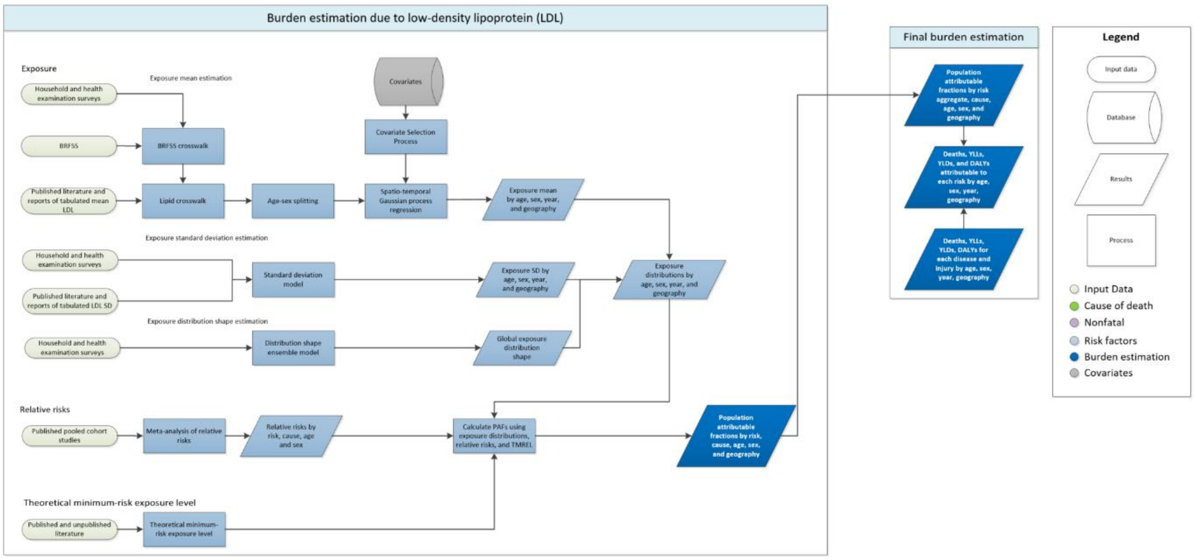

Data

Data on blood levels for low-density lipoprotein, total cholesterol, triglyceride, and high-density lipoprotein was extracted from literature and from household survey microdata and reports. The data was adjusted for total cholesterol, triglycerides, and high-density lipoprotein using the correction approach described in the Lipid Crosswalk section below. Studies were included if they were population-based and measured total LDL, total cholesterol (TC), high-density lipoprotein (HDL), and/or triglycerides (TG) were available from blood tests or if LDL was calculated using the Friedewald equation.

Assumption was made that the data was representative of the location if the geography or population chosen were not related to the diseases and if it was not an outlier compared to other data in the country or region. A candidate source was excluded if the quality of study did not warrant a valid estimate because of selection (non-representative populations) or if the study did not provide methodological details for evaluation. In a small number of cases, a data point was considered to be an outlier candidate if the level was implausibly low or high based on the data from other countries.

Where possible, individual-level data on LDL estimates were extracted from survey microdata and these were collapsed across demographic groupings to produce mean estimates in the standard GBD five-year age-sex groups. If microdata were unavailable, information from survey reports or from literature were extracted along with any available measure of uncertainty including standard error, uncertainty intervals, and sample size. Standard deviations were also extracted. Where LDL was reported split out by groups other than age, sex, location, and year (eg, by diabetes status), a weighted mean was calculated.

Lipid crosswalk: Total cholesterol consists of three major components: LDL, HDL, and TG. LDL is often calculated for an individual using the Friedewald equation, shown below:

$$LDL = TC - \left(HDL + \frac{TGL}{2.2}\right)$$

This relationship at the individual level was utilised to impute the mean LDL for a study population when only data on TC, HDL, and TGL were available. Because studies report different combinations of TC, HDL, and TGL, a single regression was constructed to utilise all available data to evaluate the relationship between each lipid and LDL at the population level. The following regression was used:

$$LDL = ind_{tc}\beta_1 TC - \left(ind_{hdl}\beta_2 HDL + ind_{tgl}\beta_3 TGL\right) + \sum \alpha_l I_l$$

Where ind_{tc}, $ind_{\text{hd}l}$, and $ind_{\text{tg}l}$ are indicator variables for whether data are available for a given lipid, I_l is an indicator variable a given set of available lipids l. α_l is a unique intercept for each set of available lipid combinations. For sources that only reported TC and HDL, $\alpha_{l\,=\text{TC, KDL}}$ should account for the missing lipid data, ie, TGL. The form of this regression allows us to estimate the betas for each lipid using all available data. As a sensitivity analysis, separate regressions for each set of available lipids was ran and found that the single regression method had much lower root-mean-squared error. A comparison of the observed versus predicted LDL for each set of available lipids. Almost no relationship was found between LDL and HDL or TGL when TC was not available, so only studies that reported TC were adjusted to LDL.

Age and sex splitting: Prior to modelling, data provided in age groups wider than the GBD five-year age groups were processed using the approach outlined in Ng and colleagues.[3] Briefly, age-sex patterns were identified using person-level microdata, and estimate age-sex-specific levels of total cholesterol from aggregated results reported in published literature or survey reports. In order to incorporate uncertainty into this process and borrow strength across age groups when constructing the age-sex pattern, a model with auto-regression on the change in mean LDL over age groups was used. Draws of the age-sex patterns were combined with draws of the input data needing to be split in order to calculate the new variance of age-sex-split data points.

Modelling strategy

Exposure estimates were produced from 1980 to 2019 for each national and subnational location, sex, and for each five-year age group starting from 25. ST-GPR framework was used to model the mean LDL at the location-, year-, age-, and sex-level.

The first step of the ST-GPR framework requires the creation of a linear model for predicting LDL at the location-, year-, age-, and sex-level. Covariates for this model were selected in two stages. First a list of variables with an expected causal relationship with LDL was created based on significant association found within high-quality prospective cohort studies reported in the published scientific literature. The second stage in covariate selection was to test the predictive validity of every possible combination of covariates in the linear model, given the covariates selected above. This was done separately for each sex. Predictive validity was measured with out of sample root-mean-squared error.

An ensemble model of the 50 models was used with the lowest root-mean-squared (RMSE) error for each sex. This allows us to utilise covariate information from many plausible linear mixed-effects models. The 50 models were each used to predict the mean LDL for every age, sex, location, and year, and the inverse-RMSE-weighted average of this set of 50 predictions was used as the linear prior. The results of the ensemble linear model were used for the first stage in an ST-GPR model. The result of the ST-GPR model are estimates of the mean LDL for each age, sex, location, and year.

The standard deviation of LDL within a population was estimated for each national and subnational location, sex, and five-year age group starting from age 25 using the standard deviation from person-level and some tabulated data sources. The shape of the distribution of LDL was estimated using all available person-level microdata sources, which was a subset of the input data into the modelling process. Briefly, an ensemble distribution created from a weighted average of distribution families was fit for each individual microdata source, separately by sex. The weights for the distribution families for each individual source were then averaged and weighted to create a global ensemble distribution for each sex.

Evidence suggests that the relative risks for LDL and TC are very similar[4] and there is a strong linear correlation between TC and LDL at the individual level, therefore relative risks reported for TC was used to approximate the relative risks for LDL. DisMod-MR 2.1 pooled the effect sizes from included studies and generate a dose-response curve for stroke associated with LDL. The tool enabled us to incorporate random effects across studies and include data with different age ranges. Relative risks (RRs) were used universally for all countries and produce RRs with uncertainty and covariance across ages, considering the uncertainty of the data points.

RRs for stroke were obtained from meta-regressions of pooled epidemiological studies: the Asia Pacific Cohort Studies Collaboration and the Prospective Studies Collaboration. RRs for stroke were modelled with log (RR) as the dependent variable and median age at event as the independent variable. Assumption that there is not a protective effect of LDL was made and therefore did not include an RR for ages 80+. Estimates of exposure to high LDL and relative risks for stroke was then used to calculate the population attributable fractions for stroke attributable to high LDL.

References

1. Boekholdt SM, Hovingh GK, Mora S, et al. Very Low Levels of Atherogenic Lipoproteins and the Risk for Cardiovascular EventsA Meta-Analysis of Statin Trials. *J Am Coll Cardiol* 2014; 64: 485–94.
2. Sabatine MS, Giugliano RP, Keech AC, et al. Evolocumab and Clinical Outcomes in Patients with Cardiovascular Disease. *N Engl J Med* 2017; 376: 1713-1722.
3. Ng M, Fleming T, Robinson M, et al. Global, regional, and national prevalence of overweight and obesity in children and adults during 1980–2013: a systematic analysis for the Global Burden of Disease Study 2013. *Lancet* 2014; 384: 766–81.
4. Wilson PF, D'Agostino RB, Levy D, Belanger AM, Silbershatz H, Kannel WB. Prediction of Coronary Heart Disease Using Risk Factor Categories. *Circulation* 1998; 97: 1837-1847.

F10. Alcohol use

Exposure is defined as the grams per day of pure alcohol consumed among individuals among current drinkers. Current drinkers is defined as the proportion of individuals who have consumed at least one alcoholic beverage (or some approximation) in a 12-month period. Additional indicators such as number of tourists within a location, their duration of stay and unrecorded alcohol stock were also used to adjust alcohol exposure estimates to account for different types of biases.

In the GBD analysis, alcohol use is associated with the increased risk for stroke among the neurological disorders. For attributing disease burden attributable to alcohol use, the TMREL was chosen as the exposure that minimises the risk of suffering burden from any given cause related to alcohol. The risk was weighted for a particular cause in GBD aggregation by the proportion of DALYs due to that cause.

The steps in the estimation of alcohol use are shown in the following chart:

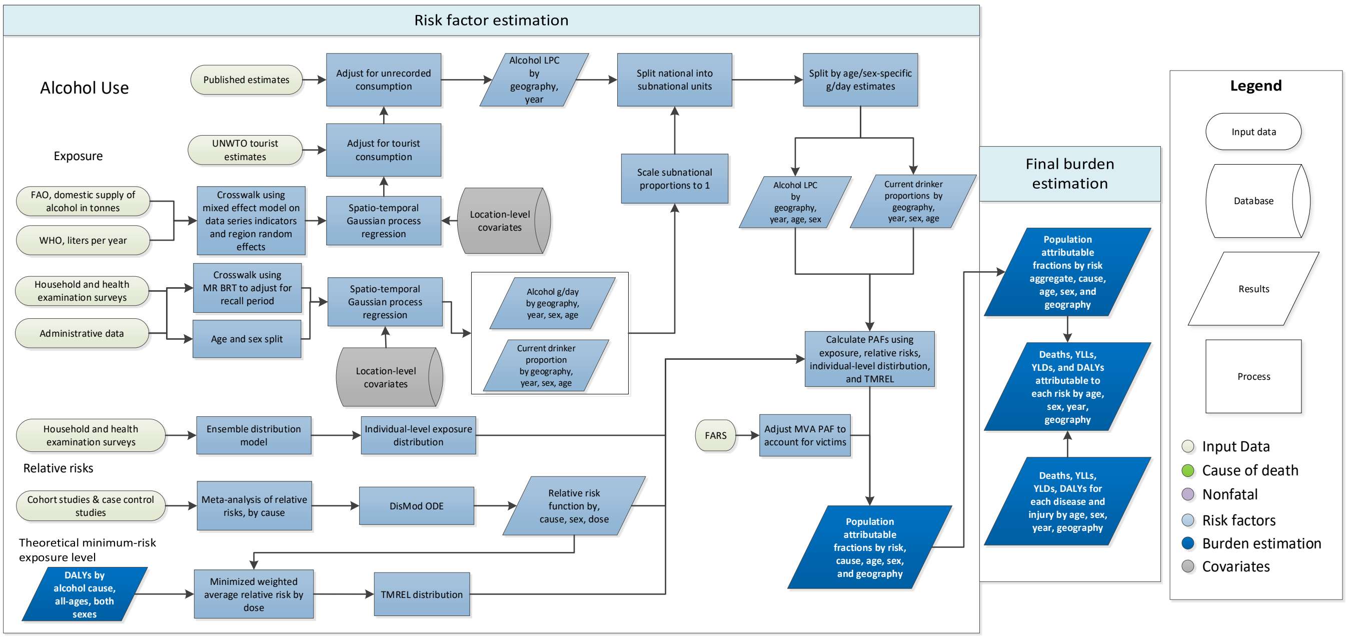

Data

A systematic review of the literature was performed to extract data on the primary indicators. The Global Health Exchange database was searched for population survey data containing participant-level information from which the required alcohol use indicators on current drinkers and alcohol consumption was formulated. Data sources were included if they captured a sample representative of the geographical location under study.

Estimates of current drinking prevalence were split by age and sex where necessary. First, studies that reported prevalence for both sexes were split using a region-specific sex ratio estimated using MR-BRT. Second, where studies reported estimates across non-GBD age groups, these were split into standard five-year age groups using the global age pattern estimated by ST-GPR. To allow for the inclusion of data that did not meet our reference definition for current drinking, two crosswalks were performed using MR-BRT. The first crosswalk converted estimates of one-month drinking prevalence to what they would be if data represented estimates of 12-month drinking prevalence. This crosswalk incorporated two binary covariates: male and age ≥ 50. The second crosswalk converted estimates of one-week drinking prevalence to 12-month drinking prevalence. This crosswalk incorporated age < 20 and male as covariates. The covariates utilised in both crosswalks were included as both x and z covariates. A uniform prior of 0 was set as the upper bound for the beta coefficients to enforce the logical constraint that one-month and one-week prevalence could not be greater than 12-month prevalence.

The raw data for supply-side estimation are domestic supply (WHO GISAH; FAO) and retail supply (Euromonitor) of litres of pure ethanol consumed. Domestic supply is calculated as the sum of production and imports, subtracting exports. The WHO and FAO sources were combined, so that FAO data were only used if there were no data available for that location-year from WHO. This was done because the WHO source takes into consideration FAO values when available. Since the WHO data are given in more granular alcohol types, the following adjustments were made:

$$LPC\ Pure\ Ethanol = 0.13 * \left(\frac{Wine}{0.973}\right)$$

$$LPC\ Pure\ Ethanol = 0.05 * \left(\frac{Beer}{0.989}\right)$$

$$LPC\ Pure\ Ethanol = 0.4 * \left(\frac{Spirits}{0.91}\right)$$

Three outliering strategies were used to omit implausible datapoints and data that created implausible model fluctuations. First, estimates from the current drinking model were used to calculate the grams of alcohol consumed per drinker per day. A point was outliered if the grams of pure ethanol per drinker per day for a given source-location-year is greater than 100 (approximately ten drinks). In the second round of outliering, the mean liters per capita value over a ten-year window was calculated. If a point is over 70% of that mean value away from the mean value, it was outliered. The 70% limit was chosen using histograms of these distances. Additionally, some manual outliering is performed to account for edge cases. Finally, data smoothing was performed by taking a three-year rolling mean over each location-year.

Next, an imputation to fill in missing years is performed for all series to remove compositional bias from our final estimates. Since the data from our main sources cover different time periods, by imputing a complete time series for each data series, the reduced probability that compositional bias of the sources is leading to biased final estimates. To impute the missing years for each series, the log ratio of each pair of sources as a function of an intercept and nested random effects on super- region, region, and location was modelled. The appropriate predicted ratio is multiplied by the source, which generates an estimated value for the missing source. For some locations where there was limited overlap between series, the predicted ratio did not make sense, and a regional ratio was used.

Finally, variance was calculated both across series (within a location-year) as well as across years (within a location-source). Additionally, if a location-year had one imputed point, the variance was multiplied by 2. If a location-year had two imputed points, the variance was multiplied by 4. The average estimates in each location-year were the input to an ST-GPR model. This uses a mixed-effects model modelled in log space with nested location random effects.

The data on the number of tourists and their duration of stay were obtained from the UNWTO.[1] A crosswalk across different tourist categories was applied, similar to the one used for the litres per capita data, to arrive at a consistent definition (ie, visitors to a country). Estimates on unrecorded alcohol stock were obtained from data available in WHO GISAH database,[2] consisting of 189 locations. For locations with no data available, the national or regional average was used.

Modelling strategy

While population-based surveys provide accurate estimates of the prevalence of current drinkers, they typically underestimate real alcohol consumption levels.[3-5] As a result, the litre per capita input was considered to be a

better estimate of overall volume of consumption. Per capita consumption, however, does not provide age- and sex-specific consumption estimates needed to compute alcohol-attributable burden of disease. Therefore, the age-sex pattern of consumption among drinkers were modelled from the population survey data and the overall volume of consumption from FAO, GISAH, and Euromonitor to determine the total amount of alcohol consumed within a location.

For data obtained through surveys, ST-GPR was used to construct estimates for each location/year/age/sex. ST-GPR was chosen due to its ability to leverage information across the nearby locations or time periods. The alcohol litres per capita (LPC) data, as well as the total number of tourists, was modeled using ST-GPR. Given the heterogeneous nature of the estimates on unrecorded consumption, 1,000 draws from the uniform distribution of the lowest and highest estimates were taken. This incorporated the diffuse uncertainty within the unrecorded estimates reported.

The alcohol LPC was adjusted for unrecorded consumption using the following equation:

$$Alcohol\ LPC = \frac{Alcohol\ LPC}{(1 - \%\ Unrecorded)}$$

The estimates for alcohol LPC were then adjusted for tourist consumption by adding in the per capita rate of consumption abroad and subtracting the per capita rate of tourist consumption domestically.

$$Alcohol\ LPC_d = Unadjusted\ Alcohol\ LPC_d + Alcohol\ LPC_{Domestic\ consumption\ abroad} - Alcohol\ LPC_{Tourist\ consumption\ domestically}$$

$$Alcohol\ LPC_i = \frac{\sum_l Tourist\ Population_l * Proportion\ of\ tourists_{i,l} * Unadjusted\ Alcohol\ LPC_l * \frac{Average\ length\ of\ stay_{i,l}}{365} *}{Population_d}$$

where:
l *is the set of all locations,* i *is either* Domestic consumption abroad *or* Tourist consumption domestically, *and* d *is a domestic location.*

After adjusting alcohol LPC by tourist consumption and unrecorded consumption for all location/years reported, sex-specific and age-specific estimates were generated by incorporating estimates modelled in ST-GPR for percentage of current drinkers within a location/year/sex/age, as well as consumption trends modelled in the ST-GPR grams per day model. First, the proportion of total consumption for a given location/year by age and sex was calculated, using the estimates of alcohol consumed per day, the population size, and the percentage of current drinkers. Then this proportion of total stock for a given location/year/sex/age was multiplied by the total stock for a given location/year to calculate the consumption in terms of litres per capita for a given location/year/sex/age. Then these estimates were converted to grams/per day. The following equations describe these calculations:

$$Proportion\ of\ total\ consumption_{l,y,s,a} = \frac{Alcohol\ \ g/day_{l,y,s,a} * Population_{l,y,s,a} * \%\ Current\ drinkers_{l,y,s,a}}{\sum_{s,a} Alcohol\ \ g/day_{l,y,s,a} * Population_{l,y,s,a} * \%\ Current\ drinkers_{l,y,s,a}}$$

$$Alcohol\ LPC_{l,y,s,a} = \frac{Alcohol\ LPC_{l,y} * Population_{l,y} * Proportion\ of\ total\ consumption_{l,y,s,a}}{\%\ Current\ drinkers_{l,y,s,a} * Population_{l,y,s,a}}$$

$$Alcohol\ g/day_{l,y,s,a} = Alcohol\ LPC_{l,y,s,a} * \frac{1000}{365}$$

where:
l is a location, y is a year, s is a sex, and a is an age group.

Then gamma distribution was used to estimate individual-level variation within location, year, sex, age drinking populations.[6,7] The parameters of the gamma distribution were chosen based on the mean and standard deviation of the 1,000 draws of alcohol g/day exposure for a given population. Standard deviation was calculated using the following formula.[8] Several alternative models were tested using the data which showed that this model performed best.

$$standard\ deviation = mean * (0.087 * female + 1.171)$$

The studies identified through the systematic review was used to calculate a dose-response for stroke, modelled using DisMod ODE. DisMod ODE estimates specific doses when categories overlap across studies, through an integration step. The results of the meta-regression were used to estimate a non-parametric curve for all doses between zero and 150 g/day and their corresponding relative risks. The relative risk for stroke was estimated by sex. PAF was defined as:

$$PAF(x) = \frac{P_A + \int_0^{150} P(x) * RR_C(x)\, dx - 1}{P_A + \int_0^{150} P(x) * RR_C(x)\, dx} \qquad P(x) = P_C * \Gamma(\boldsymbol{p})$$

where:
P_C *is the prevalence of current drinkers,* P_a *is the prevalence of abstainers,* $RR_c(x)$ *is the relative risk function for current drinkers, and* $\boldsymbol{p}$ *are parameters determined by the mean and sd of exposure*

The above equation for 1,000 draws of the exposure and relative risk models was performed. Then the estimated PAF draws were used to calculate DALYs, as per the other risk factors**.**

References

1. UN World Tourism Organization (UNWTO). UN World Tourism Organization Compendium of Tourism Statistics 2015 [Electronic]. Madrid, Spain: UN World Tourism Organization (UNWTO), 2016.
2. World Health Organization (WHO). WHO Global Health Observatory - Recorded adult per capita alcohol consumption, Total per country. Geneva, Switzerland: World Health Organization (WHO).
3. Fatal Accident Reporting System (FARS). National Highway Traffic Safety Administration, National Center for Statistics and Analysis Data Reporting and Information Division (NVS-424); 1985, 1990, 1995, 2000, 2005, 2010, 2015.
4. Chen, Li-Hui, Susan P. Baker, and Guohua Li.. Drinking history and risk of fatal injury: comparison among specific injury causes. *Accident Analysis & Prevention* 2005; 37: 245-251.
5. Bell N S, Amoroso PJ, Yore MM, Smith GS, Jones BH. Self-reported risk-taking behaviors and hospitalization for motor vehicle injury among active duty army personnel. *Am J Prev Med* 2000; 18: 85-95.
6. Taylor B, Irving HM, Kanteres F, Room R, Borges G, Cherpitel C, Greenfield T, Rehm J. The more you drink, the harder you fall: a systematic review and meta-analysis of how acute alcohol consumption and injury or collision risk increase together. *Drug Alcohol depend* 2010; 110: 108-116.
7. Vinson, Daniel C., Guilherme Borges, and Cheryl J. Cherpitel. The risk of intentional injury with acute and chronic alcohol exposures: a case-control and case-crossover study. *J Stud Alcohol* 2003; 64: 350-357.
8. Kehoe T, Gmel G, Shield KD, Gmel G, Rehm J. Determining the best population-level alcohol consumption model and its impact on estimates of alcohol-attributable harms. *Popul Health Metrics* 2012; 10:6.

F11. Non-optimal temperature

The exposure of non-optimal temperature is defined as the same day exposure to ambient temperature that is either warmer or colder than the temperature associated with the minimum mortality risk. In the GBD analysis, exposure of non-optimal temperature is associated with the increased risk for stroke among the neurological disorders. The TMREL for temperature is defined as the temperature that is associated with the lowest overall mortality attributable to the risk, in a given location and year. Given varying exposure-response curves for different mean annual temperature zones, as well as spatially and temporally varying cause compositions, TMRELs were estimated by year and location. High temperature (heat) exposure is defined as exposure to temperatures warmer than this TMREL and low temperature (cold) is defined as temperatures colder than this TMREL.

The steps in the estimation of non-optimal temperature are shown in the following chart:

Non-optimal temperature exposure

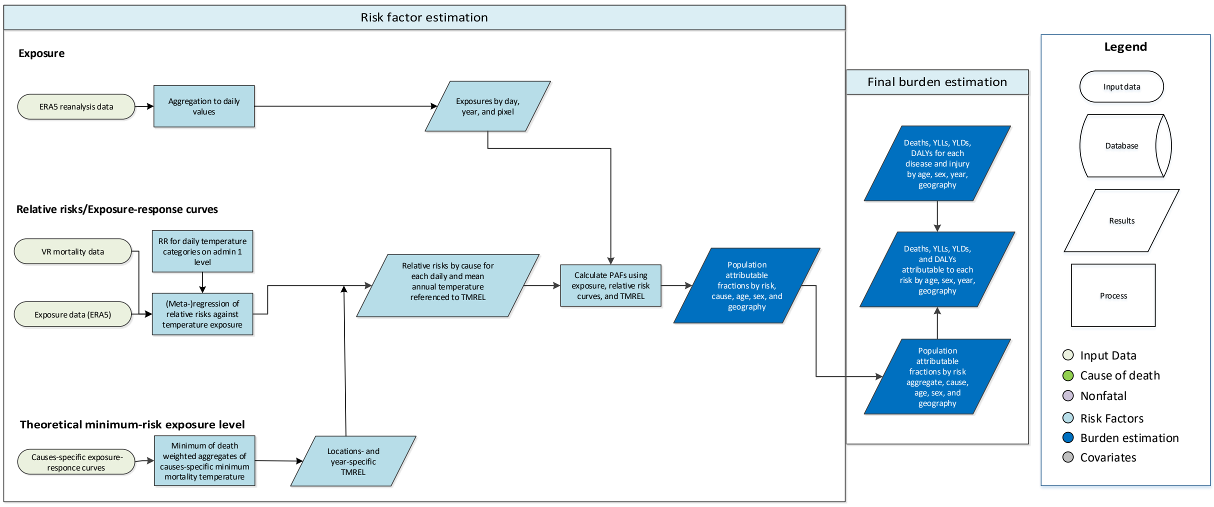

Data

Exposure estimates were derived from the ERA5 reanalysis dataset from the European Centre for Medium-Range Weather Forecasts (ECMWF). ECMWF produced ERA5 estimates using their Integrated Forecast System (IFS). Hourly values of surface temperature are available for a spatial resolution of 0.25°x0.25°. Uncertainty estimates for these temperature values, ie, the ensemble spread (standard deviation) is available for every 3 hours (00:00, 03:00, 06:00, 09:00, 12:00, 15:00, 18:00, 21:00) for a spatial resolution of 0.5°x0.5°. For this analysis, data were available from 1979 to 2019.[1,2] Daily averages of temperature were calculated and spread for each pixel and then assigned an uncertainty value to each daily temperature value. Based on the spread 1,000 draws of each daily temperature pixel were derived.

Multi-temporal, globally consistent, high-resolution human population data at 1 km x 1 km resolution from WorldPop for the years 2000, 2005, 2010 and 2020 were used for calculating population-weighted location means. The data was interpolated in-between the 5-year estimation bins to obtain annual data. Further, the data was extrapolated until 1990 by using the 2000-2005 growth rate for back-casting.

Deaths at the individual-level that included information regarding the cause (ie, ICD code), date, and the location at the second administrative level (admin2) or finer were collected from the GBD cause-of-death database for vital registration data sources. The GBD standard procedure for garbage code redistribution was adapted to redistribute daily mortality data rather than annual data and mapped ICD causes to GBD causes for level 3.

Modelling strategy

To estimate cause-specific mortality, based on average daily temperature and temperature zone (defined by mean annual temperature), a robust meta-regression framework, implemented through the MR-BRT (Bayesian, regularised, trimmed) tool was used. The tool allows 1) a meta-analytic framework that can handle heterogeneous data sources; 2) a robust approach to outlier detection and removal (trimming); 3) specification

of the functional dependence of outcome versus average daily temperature and temperature zone as a 2-dimensional surface through a spline interface.

For the purpose of modelling the relationship between mortality due to stroke and mean annual and daily temperature, a monotonicity in the direction of daily temperature was imposed. For all J-shaped curves that depicted an increase in mortality above and below a threshold, the curve was forced to monotonically decrease at the lower end of the temperature distribution and to monotonically increase at the upper end. For all external causes that displayed a monotonic increase over the entire temperature range, monotonicity was imposed only in the direction of warmer temperatures. When fitting the surface, 2 knots of degree 3 were placed in the direction of mean annual temperature. In the direction of daily mean temperature, 3 knots of degree 3 for J-shaped causes and 2 knots of degree 1 for external causes were placed that monotonically increase over temperature range.

The uncertainty was estimated using a two-step approach. First, the uncertainty of the mean surface was derived from the measurement error using the fit-retrofit error. Second, uncertainty from the random effects was added by sampling it separately from the cold and warm side.

The PAF was calculated for each temperature pixel and each day of the year (ie, pixel-day). Subsequently, population-weighted each pixel using the fraction of the population living in a given pixel relative to the GBD location. Depending on whether the daily mean temperature was below or above the TMREL, the effect was assigned to either low or high temperature. Daily population-weighted high and low temperature PAFs were then aggregated for the location and the year. Temperature effects can be either harmful or protective depending on whether the RR is above or below 1. For harmful temperature effects, ie, effects with a RR above 1, the following equation was used to derive PAFs: PAF=(RR-1)/RR; For temperature effects exhibiting a protective effect the equation was adapted by implementing the reverse RR: PAF=-((1/RR)-1)/(1/RR). The PAF associated with non-optimal temperature exposure is an aggregate of heat and cold effects in each location and year. The temperature attributable burden was estimated as the product of the total burden for stroke and the corresponding PAF for each location, year, age group, and sex.

References

1. Hersbach H, Bell B, Berrisford P, et al. Global reanalysis: goodbye Global reanalysis: goodbye ERA-Interim, hello. ECMWF Newsletter 2019; 159: 17-24. doi:10.21957/vf291hehd7.
2. Copernicus Climate Change Service (C3S) (2017): ERA5: Fifth generation of ECMWF atmospheric reanalyses of the global climate. Copernicus Climate Change Service Climate Data Store (CDS), September 2019.

F12. Low physical activity

Physical activity performed by adults greater than or equal to 25 years of age is measured, for durations of at least ten minutes at a time, across all domains of life (leisure/recreation, work/household and transport). Frequency, duration, and intensity of activity is used to calculate total metabolic equivalent minutes per week. MET (Metabolic Equivalent) is the ratio of the working metabolic rate to the resting metabolic rate. One MET is equivalent to 1 kcal/kg/hour and is equal to the energy cost of sitting quietly. A MET is also defined as the oxygen uptake in ml/kg/min with one MET equal to the oxygen cost of sitting quietly, around 3.5 ml/kl/min.

In the GBD analysis, low physical activity is associated with the increased risk for stroke among the neurological disorders. The TMREL for physical inactivity was defined in the range 3,000-4,500 MET-min per week, which is calculated as the exposure at which minimal deaths due to stroke occurred.[1]

The steps in the estimation of low physical activity are shown in the following chart:

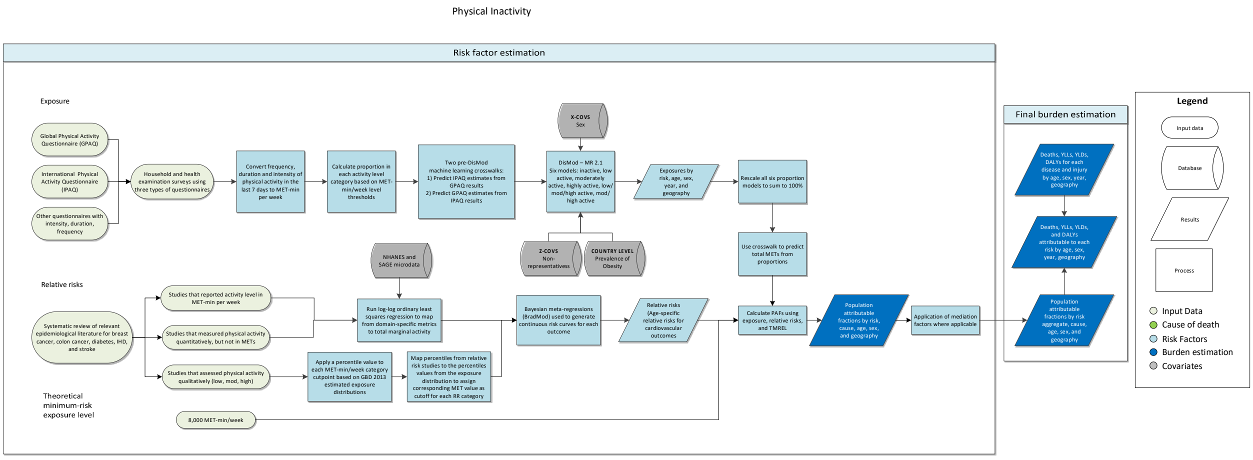

Data

Surveys of the general adult population were included that captured self-reported physical activity in all domains of life (leisure/recreation, work/household and transport), where random sampling was used. Data were primarily derived from two standardised questionnaires: The Global Physical Activity Questionnaire[2] (GPAQ) and the International Physical Activity Questionnaire[3] (IPAQ), the other survey instruments that asked about intensity, frequency and duration of physical activities performed across all activity domains were also included. Due to a lack of a consistent relationship on the individual level between activity performed in each domain and total activity, the studies that included only recreational/leisure activities were not included. Physical activity level is categorised by total MET-minutes per week using four categories based on rounded values closest to the quartiles of the global distribution of total MET-minutes/week. The lower limit for the Level 1 category (600 MET-min/week) is the recommended minimum amount of physical activity to get any health benefit. Four categories with higher thresholds were included rather than the GPAQ and IPAQ recommended three categories to better capture any additional protective effects from higher activity levels.

- Level 0: < 600 MET-min/week (inactive)
- Level 1: 600-3999 MET-min/week (low-active)
- Level 2: 4000-7,999 MET-min/week (moderately-active)
- Level 3: ≥ 8,000 MET-min/week (highly active)

Several surveys that focused specifically on leisure activity, were not included because they did not comprise all three domains (work, transport and leisure). In addition, surveys that did not report frequency, duration, and intensity of activity were also excluded.

Modelling strategy

A machine learning crosswalk was used to predict IPAQ estimates for GPAQ results and GPAQ estimates for IPAQ results, with original and estimated results then being combined to get one comprehensive IPAQ dataset and one comprehensive GPAQ dataset. The proportion of each country/year/age/sex subpopulation in each of the four activity levels was estimated using 12 separate Dismod models (one set of six for IPAQ and one for

GPAQ for IPAQ). Six categories of physical activity prevalence were used rather than four to accommodate the different MET minute/week cutoffs presented in tabulated data sources where individual unit record data was not available. Since the accepted threshold/definition for inactivity is consistently <600 MET-minutes/week, the vast majority of tabulated data was broken down into proportion inactive (model A) and proportion low, moderate or highly active (model B).

Model	Label	MET-min/week	Name of sequelae in online visualisation tool
A	Inactive	<600	Physical inactivity and low physical activity, inactive
B	Low/moderately/highly active	≥600	Physical inactivity and low physical activity, low/moderately/highly active
C	Low active	600-3999	Physical inactivity and low physical activity, low active
D	Moderately/highly active	>4000	Physical inactivity and low physical activity, moderately/highly active
E	Moderately active	4000-7999	Physical inactivity and low physical activity, moderately active
F	Highly active	≥8,000	Physical inactivity and low physical activity, highly active

These models have mesh points at 0 15 25 35 45 55 65 75 85 100, and a study-level fixed effect on integrand variance (Z-cov) for whether a study was nationally representative or not, to account for the heterogeneity introduced boy studies that are not generalizable to the entire population. They also have national level fixed effects on prevalence of obesity.

After DisMod, each of the 6 models specific to each data source were rescaled so that the proportions sum to one. For models A and B, the sum of the proportion in each category were rescaled to be equal to one. Next the sum of model C and D were rescaled to be equal to the rescaled value from model B. Then the sum of models E and F were rescaled to be equal to the rescaled value from model D. After these three rescales, the proportion for each of the four categories sums to 1. Scaled results for each data source are then hybridised to produce one set of results for the prevalence of the four categories of physical activity.

Total MET-minutes per week were estimated indirectly. Two specific machine learning algorithms (Random Forest & XGBoost) were used that were trained using data that could characterise the relationship between total MET-mins/week and each of the categorical prevalence of physical activity. This resulted in country-year-age-sex specific estimates of total physical activity in the form of MET-minutes per week. Utilising microdata on total MET-mins per week from individual-level surveys, the distribution of activity level was characterised at the population level. Then ensemble approach was used to distribution fitting, borrowing characteristics from individual distributions to tailor a unique distribution to fit the data using a weighting scheme. The standard deviation of each population's activity was characterized through a linear regression that captured the relationship between standard deviation and mean activity levels in nationally representative IPAQ surveys:

$$n\ (Standard\ deviation) = \beta_0\ +\ \beta_1\ \times\ ln\ (Mean_i) +\ \beta_2\ \times Age_i\ +\ \beta_3\ \times SR_i\ +\ \beta_4\ \times Fem_i$$

Where, Age_i is the youngest age in population i's age group, SR_i is the super region in which the population lives, and Femi is a Boolean value depicting whether the population is female.

The coefficients of this regression was then applied to the outputs of our estimate of total MET-minutes per week regression outputs to calculate the standard deviation by country, year, age, and sex.

To estimate the effect size of the change in physical activity level on stroke, dose-response meta-analysis of prospective cohort studies was used.[1] There is a well-documented attenuation of the risk for stroke due to metabolic risks factors throughout one's life. To incorporate this age trend in the relative risks, the median age-at-event across all cohorts were identified and considered as the reference age-group. Risk curves were then assigned to this reference age group. Finally, the percent change in relative risks between each age group and the reference age group was derived by averaging percentage changes in relative risks of all metabolic mediators. Estimates of exposure to low physical activity and relative risks for stroke was then used to calculate the population attributable fractions for stroke attributable to low physical activity.

References

1. Kyu HH, Bachman VF, Alexander LT, Mumford JE, Afshin A, Estep K, Veerman JL, Delwiche K, Iannarone ML, Moyer ML, Cercy K. Physical activity and risk of breast cancer, colon cancer, diabetes, ischemic heart disease, and ischemic stroke events: systematic review and dose- response meta-analysis

for the Global Burden of Disease Study 2013. *BMJ* 2016; 354: i3857.
2. IPAQ Research Committee. Guidelines for data processing and analysis of the International Physical Activity Questionnaire (IPAQ)–short and long forms. Retrieved September. 2005; 17: 2008.
3. World Health Organization. Global Physical Activity Questionnaire (GPAQ) Analysis Guide. 2011. Geneva, Switzerland: *WHO Google Scholar* 2013.

F13. Low birthweight and short gestation

In the GBD analysis, low birthweight and short gestation is associated with the increased risk for meningitis and encephalitis among the neurological disorders.

Low birthweight for gestation and short gestation for birthweight are separate risk factors, however the exposures and relative risks for both were estimated jointly through the low birthweight and short gestation parent risk factor. The meaning of "low birthweight" and "short gestation" in GBD have subtle definitional differences compared to other usages of "low birthweight" and "short gestation" in the literature. The term "low birthweight" has historically been used to refer to birthweight less than 2,500 grams. However, because the goal of the GBD risk factors analysis was to quantify the entirety of attributable burden due to each risk factor, the GBD definition of "low birthweight" therefore refers to all birthweight below the TMREL for birthweight. Likewise, new-borns were typically classified into gestational age categories of "extremely preterm" (<28 weeks of gestation), "very preterm" (28-<32 weeks of gestation), and "moderate to late preterm" (32-<37 weeks of gestation). "Short gestation" refers to gestational age below the gestational age TMREL. Exposures and relative risks for the GBD low birthweight and short gestation risk factors were categorised into different combinations of joint 500-gram birthweight and 2-week gestational age. The lowest risk overall 500-gram/2-week bin was the overall TMREL. The univariate TMRELs vary with gestational age and birthweight. The lowest risk gestational age varies by birthweight category and the lowest risk birthweight vary with gestational age category. The latter were used to quantify univariate attributable risk. Under this framework, all attributable burden under the joint TMREL were referred to jointly as burden of low birthweight and short gestation. All attributable burden to birthweights under the TMREL for each gestational age category were, on aggregate, "low birthweight", and all attributable burden to gestational ages under the TMREL for each birthweight category were, on aggregate, "short gestation." Each combination of 500-grams and 2-weeks was associated with a relative risk for mortality by neonatal period (early and late neonatal) and by the causes, and relative to the joint TMREL.

The steps in the estimation of low birthweight and short gestation are shown in the following flowchart:

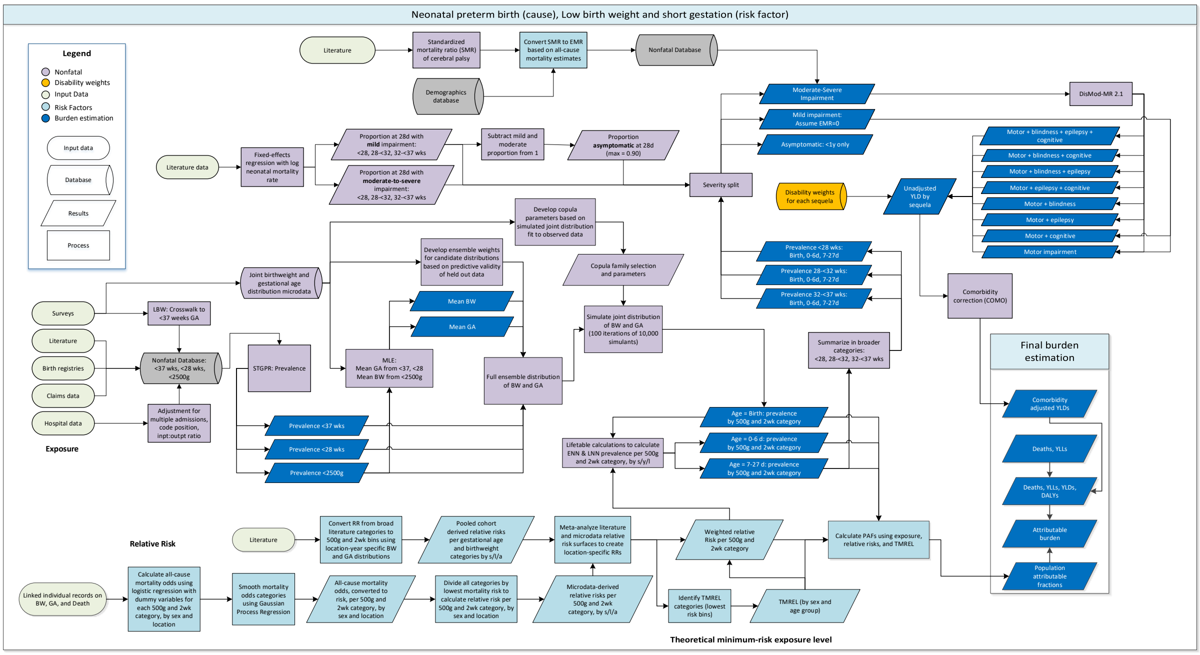

Data

Input data needed to model univariate gestational age and birthweight distributions at birth (Step A) are:

- Prevalence of preterm birth (<37 weeks), by location, year and sex
- Prevalence of preterm birth (<28 weeks), by location, year and sex
- Mean gestational age, by location, year and sex
- Gestational age microdata
- Prevalence of low birthweight (<2,500 grams), by location, year and sex
- Mean birthweight, by location, year and sex
- Birthweight microdata

To model the joint distribution and birthweight (Step B), joint microdata of gestational age and birthweight are also required. Additional inputs to modelling joint distributions from birth to 28 days (Step C) are all-cause mortality by location, year and sex, and joint birthweight and gestational age microdata linked to mortality outcomes. Prevalence of extremely preterm birth (<28 weeks) and preterm birth (<37 weeks) were modelled using vital registration, survey, and clinical data. For the preterm models, only inpatient and insurance claims data were included from clinical informatics datasets; outpatient data were excluded because they were more likely to capture repeated visits by the same child rather than unique visits. Prevalence of low birthweight (<2,500 grams) was modelled using only vital registration and survey data.

Several data processing steps were used prior to modelling. First, empirical age and sex ratios from previous GBD 2019 Decomposition 1 models was applied to disaggregate observations that did not entirely fit in one GBD age category or sex. Ratios were determined by dividing the result for a specific age and sex by the result for the aggregate age and sex specified in a given observation. It is our intention to update this splitting process annually. Second, to correct for the missingness in low birthweight data extracted from DHS surveys, birthweight was imputed using the Amelia II (Version 1.7.6) package in R. Birthweight was predicted using standard Amelia imputation methods from the following variables also in the DHS surveys: urbanicity, sex, birthweight recorded on card, birth order, maternal education, paternal education, child age, child weight, child height, mother's age at birth, mother's weight, shared toilet facility, and household water treated. Third, crosswalking was used to process data in the extremely preterm (<28 weeks) and preterm (<37 weeks) models. All preterm crosswalks were done using meta-regression-regularized, Bayesian, trimmed (MR-BRT). Insurance claims data in extremely preterm (<28 weeks) data were adjusted to vital registration data. Insurance claims data and inpatient data were also adjusted to vital registration in preterm (<37 weeks) conditions. The crosswalk for inpatient data had a spline on the prevalence of inpatient data. Once all claims and inpatient preterm (<37 weeks) data were adjusted, low birthweight data were crosswalked to post-claims and inpatient preterm (<37 weeks) data. If low birthweight data in countries that were 1) categorised as "data-rich" locations in cause-of-death modelling or had at least ten consecutive years of vital registration data recording gestational age and 2) had both preterm birth and low birthweight data, crosswalked low birthweight data were outliered so that the model was informed only by the gestational age data.

Modelling strategy

Step A: Estimating univariate birthweight and gestational age distributions at birth

Ensemble distribution models was constructed using mean of the distribution, variance of the distribution, and the weights of the distributions being ensemble. To model mean and variance for all location, year and sex for birthweight and gestational age, ST-GPR models was used to model prevalence of low birthweight, extremely preterm, and preterm birth for all location, year and sex at birth. To model mean birthweight for all location, year and sex, OLS linear regression was used to regress mean birthweight on log-transformed low birthweight prevalence. This model was then used to predict mean birthweight for all location year and sex, using the prevalence of low birthweight (<2500 grams) modelled for all location, year and sex in ST-GPR. Similarly, to model gestational age mean for all location, year and sex, OLS linear regression model was used to regress mean gestational age on log-transformed preterm prevalence. Mean gestational age for all location year and sex was predicted using the preterm birth (<37 weeks) estimated modelled in ST-GPR.

Global ensemble weights for gestational age were derived by using a 3 million sample of all available gestational age and birthweight microdata in Table 8 to select the ensemble weights. The two distribution families that received the highest weights were the Weibull (43%) and log-logistic (21%) distributions. Global ensemble weights for birthweight were derived using a 3 million sample of all available microdata, in addition to birthweight microdata available primarily through the DHS and MICS surveys. The four distribution families

that received the highest weights were the mirror gamma (31%), log-logistic (19%), normal (10%), and mirror gumbel (10%) distributions.

For each location, year and sex, given the mean and ensemble weights, the variance was optimised to minimise error on the prevalence of preterm birth (<37 weeks) for the gestational age distribution and prevalence of low birthweight (<2500 grams) for the birthweight distribution.

Step B: Estimating joint birthweight and gestational age distributions at births

In order to model the joint distribution of gestational age and birthweight from separate distributions, information was needed about the correlation between the two distributions. Distributions of gestational age and birthweight are not independent; the Spearman correlation for each country where joint microdata were available, pooling across all years of data available, ranged from 0.25 to 0.49. The overall Spearman correlation was 0.38, pooling across all countries in the dataset.

The joint distribution of birthweight and gestational age per location-year-sex was modelled using the global copula family and parameters selected and the location-year-sex gestational age and birthweight distributions. The joint distribution was simulated 100 times to capture uncertainty. Each simulation consisted of 10,000 simulated joint birthweight and gestational age data points. Each joint distribution was divided into 500g by 2-week bins to match the categorical bins of the relative risk surface. Birth prevalence was then calculated for each 500g by 2-week bin.

Step C: Estimating joint distributions from birth to the end of the neonatal period

Early neonatal and late neonatal prevalence was estimated using life table approaches for each 500-gram and 2-week bin. Using the all-cause early neonatal mortality rate for each location-year-sex, births per location-year-sex-bin, and the relative risks for each location-year-sex bin in the early neonatal period, the all-cause early neonatal mortality rate was calculated for each location-year-sex bin. The early neonatal mortality rate per bin was used to calculate the number of survivors at 7 days and prevalence in the early neonatal period. Using the same process, the all-cause late neonatal mortality rate for each location-year-sex was paired with the number of survivors at 7 days and late neonatal relative risks per bin to calculate late neonatal prevalence and survivors at 28 days.

Relative risks and population attributable fraction

The available data for deriving relative risk was only for all-cause mortality. The relative risk of all-cause mortality across all available sources and meningitis and encephalitis was analysed based on criteria of biologic plausibility. For each location, data were pooled across years, and the risk of all-cause mortality at the early neonatal period and late neonatal period at joint birthweight and gestational age combinations was calculated. To calculate relative risk at each 500-gram and 2-week combination, logistic regression was first used to calculate mortality odds for each joint 2-week gestational age and 500-gram birthweight category. Mortality odds were smoothed with Gaussian Process Regression, with the independent distributions of mortality odds by birthweight and mortality odds by gestational age serving as priors in the regression.

A pooled country analysis of mortality risk in the early neonatal period and late neonatal period by short gestational age category in developing countries were also converted into 500-gram and 2-week bin mortality odds surfaces. Location-specific relative risk surfaces, derived from location-specific estimates of with-condition mortality of preterm birth, were converted into 500-gram and 2-week bin mortality odds. The meta-analysed mortality odds surface for each location was smoothed using Gaussian Process Regression and then converted into mortality risk. To calculate mortality relative risks, the risk of each joint 2-week gestational age and 500-gram birthweight category were divided by the risk of mortality in the joint gestational age and birthweight category with the lowest mortality risk.

For each of the country-derived relative risk surfaces, the 500-gram and 2-week gestational age joint bin with the lowest risk was identified. This bin differed within each country dataset. To identify the universal 500-gram and 2-week gestational age category that would serve as the universal TMREL for our analysis, the bins were chosen that were identified to be the TMREL in each country dataset to contribute to the universal TMREL. Therefore, the joint categories that served as our universal TMREL for the low birthweight and short gestation risk factor were "38-40 weeks of gestation and 3,500-4,000 grams", "38-40 weeks of gestation and 4,000-4,500 grams", and "40-42 weeks of gestation and 4,000-4,500 grams". As the joint TMREL, all three categories were assigned to a relative risk equal to 1.

The total PAF for the low birthweight and short gestation joint risk factor was calculated by summing the PAF calculated from each 500-gram and 2-week category, with the lowest risk category among all the 500-gram and 2-week categories serving as the TMREL. The equation for calculating PAF for each 500-gram and 2-week category is:

$$PAF_{joasgt} = \frac{\sum_{x=1}^{u} RR_{joast}(x)P_{jasgt}(x) - RR_{joasg}(TMRE_{jas})}{\sum_{x=1}^{u} RR_{joas}(x)P_{jasgt}(x)}$$

To calculate the PAFs for the univariate risks ('short gestation for birthweight' and 'low birthweight for gestation'), relative risks are first weighted by global exposure in 2019, summed across one of the dimensions (gestational age or birthweight), and then rescaled by the maximum RR in the TMREL block (38-42 weeks of gestation and 3500-4500 grams). Any RR less than 1 was set to 1. Exposure was also summed across the same dimension, and the univariate PAF equalled the sum of the product of the weighted RRs and exposures.

Reference

1. Katz J, Lee AC, Kozuki N, Lawn JE, Cousens S, Blencowe H, CHERG Small-for-Gestational-Age-Preterm Birth Working Group, et al. Mortality risk in preterm and small-for-gestational-age infants in low-income and middle-income countries: a pooled country analysis. *Lancet* 2013; 382: 417–25.

G. Uncertainty intervals

Point estimates for each metric of interest were derived from the mean of the draws, while 95% uncertainty intervals (UIs) were derived from the 2.5th and 97.5th percentiles of the 1,000 draw level values. Uncertainty in the estimation is attributable to sample size variability within data sources, different availability of data by age, sex, year, or location, and cause-specific model specifications. The UIs were determined for components of cause-specific estimation based on 1,000 draws from the posterior distribution of cause specific mortality by age, sex, and location for each year included in the GBD 2019 analysis. Similarly, for non-fatal estimates if there was a change in disease estimates between locations or over time that was in the same direction in more than 950 of the 1,000 sample, it is reported as significant. With this approach, uncertainty could be quantified and propagated into the final quantities of interest.

2. GBD 2019 India data inputs for neurological disorders morbidity, deaths, risk factors, and covariates

Aarhus University, Addiction Switzerland Research Institute, Alcohol Research Group, Public Health Institute, Centre for Addiction and Mental Health, Centre for Alcohol Policy Research, Turning Point Alcohol and Drug Centre, Kettil Bruun Society for Social and Epidemiological Research on Alcohol, University of North Dakota. India - Karnataka Gender, Alcohol and Culture: An International Study (GENACIS) 2003.
Achutha Menon Centre for Health Science Studies, Sree Chitra Tirunal Institute for Medical Sciences and Technology, Kerala State Health Services Department. Prevention and control of non-communicable diseases in Kerala, 2016-17 [Data shared for analysis]
Action Aid International, Regional Medical Research Centre - Port Blair, Indian Council of Medical Research, National Institute of Mental Health and Neurosciences. Report on alcohol consumption prevalence and patterns in Andaman and Nicobar Islands 2007.
Ahankari A, Bapat S, Myles P, Fogarty A, Tata L. Factors Associated with preterm delivery and low birthweight: a study from rural Maharashtra, India. F1000Res. 2017; 6: 72.
Ahsan T, Shahid M, Mahmood T, Jabeen R, Jehangir U, Saleem M, Ahmed N, Shaheer A. Role of dexamethasone in acute bacterial meningitis in adults. J Pak Med Assoc. 2002; 52: 233-9.
Albanese E, Taylor C, Siervo M, Stewart R, Prince MJ, Acosta D. Dementia severity and weight loss: a comparison across eight cohorts. The 10/66 study. Alzheimers Dement. 2013; 9: 649-56.
Ali MK, Bhaskarapillai B, Shivashankar R, Mohan D, Fatmi ZA, Pradeepa R, Masood Kadir M, Mohan V, Tandon N, Narayan KM, Prabhakaran D, CARRS investigators. Socioeconomic status and cardiovascular risk in urban South Asia: The CARRS Study. Eur J Prev Cardiol. 2016; 23: 408-19.
All India Institute of Medical Sciences, Bangur Institute of Neurology, Indian Statistical Institute, National Neurosciences Centre. Kolkata study for epidemiology of neurological disorders 2003-2004.
Anand K, Kant S, Kumar G, Kapoor SK. "Development" is not essential to reduce infant mortality rate in India: experience from the Ballabgarh project. J Epidemiol Community Health. 2000; 54: 247-53.
Andresen PR, Ramachandran G, Pai P, Maynard A. Women's personal and indoor exposures to PM2.5 in Mysore, India: impact of domestic fuel usage. Atmos Environ. 2005; 39: 5500-8.
Anjana RM, Sudha V, Lakshmipriya N, Subhashini S, Pradeepa R, Geetha L, Ramya MB, Gayathri R, Deepa M, Unnikrishnan R, Sreekumaran NBV, Kurpad AV, Mohan V. Reliability and validity of a new physical activity questionnaire for India. Int J Behav Nutr Phys Act. 2015; 12: 2015.
Aung TW, Jain G, Sethuraman K, Baumgartner J, Reynolds C, Grieshop AP, Marshall JD, Brauer M. Health and climate-relevant pollutant concentrations from a carbon-finance approved cookstove intervention in rural India. Environ Sci Technol. 2016; 50: 7228-38.
Atilola O, Stevanovic D, Balhara YPS, Avicenna M, Kandemir H, Knez R, Petrov P, Franic T, Vostanis P. Role of personal and family factors in alcohol and substance use among adolescents: an international study with focus on developing countries. J Psychiatr Ment Health Nurs. 2014; 21: 609–17.
Awasthi S, Pande VK. Cause-specific mortality in under-fives in the urban slums of Lucknow, North India. J Trop Pediatr. 1998; 44: 358-61.
Badrinath SS, Sharma T, Biswas J, Srinivas V. A case control study of senile cataract in a hospital based population. Indian J Ophthalmol. 1996; 44: 213-7.
Balakrishnan K, Ghosh S, Ganguli B, Sambandam S, Bruce N, Barnes DF, Smith KR. State and national household concentrations of PM2.5 from solid cookfuel use: results from measurements and modeling in India for estimation of the global burden of disease. Environ Health. 2013; 12: 77.
Balagopal P, Kamalamma N, Patel TG, Misra R. A community-based participatory diabetes prevention and management intervention in rural India using community health workers. Diabetes Educ. 2012; 38: 822-34.
Banerjee A, Mondal NK, Das D, Ray MR. Neutrophilic inflammatory response and oxidative stress in premenopausal women chronically exposed to indoor air pollution from biomass burning. Inflammation. 2012; 35: 671-83.
Banerjee TK, Dutta S, Das S, Ghosal M, Ray BK, Biswas A, Hazra A, Chaudhuri A, Paul N, Das SK. Epidemiology of dementia and its burden in the city of Kolkata, India. Int J Geriatr Psychiatry. 2017; 32: 605-614.
Banerjee TK, Dutta S, Ray BK, Ghosal M, Hazra A, Chaudhuri A, Das SK. Epidemiology of epilepsy and its burden in Kolkata, India. Acta Neurol Scand. 2015; 132: 203-11.
Banerjee TK, Hazra A, Biswas A, Ray J, Roy T, Raut DK, Chaudhuri A, Das SK. Neurological disorders in children and adolescents. Indian J Pediatr. 2009; 76: 139-46.
Banerjee TK, Mukherjee CS, Dutt A, Shekhar A, Hazra A. Cognitive dysfunction in an urban Indian population – some observations. Neuroepidemiology. 2008; 31: 109-14.
Banerjee TK, Mukherjee CS, Sarkhel A. Stroke in the urban population of Calcutta – an epidemiological study. Neuroepidemiology. 2001; 20: 201-7.
Banerjee TK, Ray BK, Das SK, Hazra A, Ghosal MK, Chaudhuri A, Roy T, Raut DK. A longitudinal study of epilepsy in Kolkata, India. Epilepsia. 2010; 51: 2384-91.
Bang AT, Bang RA, Baitule SB, Reddy MH, Deshmukh MD. Effect of home-based neonatal care and management of sepsis on neonatal mortality: field trial in rural India. Lancet. 1999; 354: 1955-61.
Baqui AH, Darmstadt GL, Williams EK, Kumar V, Kiran TU, Panwar D, Srivastava VK, Ahuja R, Black RE, Santosham M. Rates, timing and causes of neonatal deaths in rural India: implications for neonatal health programmes. Bull World Health Organ. 2006; 84: 706-13.
Baruah J, Kusre G, Bora R. Pattern of Gross Congenital Malformations in a Tertiary Referral Hospital in Northeast India. Indian J Pediatr. 2015; 82: 917–22.
Basavanagowdappa H, Guruswamy M, Ravi MD, Mahesh PA. India - Mysore JSS Hospital Inpatient Data 2014. Mysore, Karnataka, India: JSS Hospital.
Basavanagowdappa H, Guruswamy M, Ravi MD, Mahesh PA. India - Mysore JSS Hospital Inpatient Data 2015. Mysore, Karnataka, India: JSS Hospital.
Basavanagowdappa H, Guruswamy M, Ravi MD, Mahesh PA. India - Mysore JSS Hospital Inpatient Data 2016. Mysore, Karnataka, India: JSS Hospital.

Basavanagowdappa H, Guruswamy M, Ravi MD, Mahesh PA. India - Mysore JSS Hospital Inpatient Data 2017. Mysore, Karnataka, India: JSS Hospital.

Beegom R. Diet, central obesity and prevalence of hypertension in the urban population of South India. Int J Cardiol. 1995; 51: 183-91.

Beegom R, Singh RB. Association of higher saturated fat intake with higher risk of hypertension in an urban population of Trivandrum in South India. Int J Cardiol. 1997; 58: 63-70.

Benara SK, Singh P. Validity of causes of infant death by verbal autopsy. Indian Pediatr. 1999; 66: 647-50.

Bhagyalaxmi A, Atul T, Shikha J. Prevalence of risk factors of non-communicable diseases in a District of Gujarat, India. J Health Popul Nutr. 2013; 31: 78-85.

Bhandari N, Bahl R, Taneja S, Martines J, Bhan MK. Pathways to infant mortality in urban slums of Delhi, India: implications for improving the quality of community- and hospital-based programmes. J Health Popul Nutr. 2002; 20: 148-55.

Bhandari N, Mazumder S, Taneja S, Sommerfelt H, Strand TA. Effect of implementation of Integrated Management of Neonatal and Childhood Illness (IMNCI) programme on neonatal and infant mortality: cluster randomised controlled trial. BMJ. 2012; 344: e1634.

Bhansali A, Dhandania VK, Deepa M, Anjana RM, Joshi SR, Joshi PP, Madhu SV, Rao PV, Subashini R, Sudha V, Unnikrishnan R, Das AK, Shukla DK, Kaur T, Mohan V, Pradeepa R. Prevalence of and risk factors for hypertension in urban and rural India: the ICMR-INDIAB study. J Hum Hypertens. 2015; 29: 204–9.

Bharati DR, Pal R, Kar S, Rekha R, Yamuna TV, Basu M. Prevalence and determinants of diabetes mellitus in Puducherry, South India. J Pharm Bioallied Sci. 2011; 3: 513-8.

Bharati S, Pal M, Bhattacharya BN, Bharati P. Prevalence and causes of chronic energy deficiency and obesity in Indian women. Hum Biol. 2007; 79: 395-412.

Bharucha NE, Bharucha EP, Bharucha AE, Bhise AV, Schoenberg BS. Prevalence of epilepsy in the Parsi community of Bombay. Epilepsia. 1988; 29: 111-5.

Bharucha NE, Bharucha EP, Bharucha AE, Bhise AV, Schoenberg BS. Prevalence of Parkinson's disease in the Parsi community of Bombay, India. Arch Neurol. 1988; 45: 1321-3.

Bharucha NE, Bharucha EP, Dastur HD, Schoenberg BS. Pilot survey of the prevalence of neurologic disorders in the Parsi community of Bombay. Am J Prev Med. 1987; 3: 293-9.

Bharucha NE, Bharucha EP, Wadia NH, Singhal BS, Bharucha AE, Bhise AV, Kurtzke JF, Schoenberg BS. Prevalence of multiple sclerosis in the Parsis of Bombay. Neurology. 1988; 38: 727-9.

Biswas M, Manna CK. Prevalence of hypertension and sociodemographic factors within the Scheduled Caste community of the District Nadia, West Bengal, India. High Blood Press Cardiovasc Prev. 2011; 18: 179–85.

Blencowe H, Cousens S, Jassir FB, Say L, Chou D, Mathers C, Hogan D, Shiekh S, Qureshi ZU, You D, Lawn JE; Lancet Stillbirth Epidemiology Investigator Group. National, regional, and worldwide estimates of stillbirth rates in 2015, with trends from 2000: a systematic analysis. Lancet Glob Health. 2016; 4: e98-108.

Bray F, Colombet M, Mery L, PiÃ±eros M, Znaor A, Zanetti R and Ferlay J, editors (2017). Cancer Incidence in Five Continents, Vol. XI (electronic version). Lyon: International Agency for Research on Cancer. Available from: https://ci5.iarc.fr/CI5-XI/Default.aspx

Broor S, Pandey RM, Ghosh M, Maitreyi RS, Lodha R, Singhal T, Kabra SK. Risk factors for severe acute lower respiratory tract infection in under-five children. Indian Pediatr. 2001; 38: 1361-9.

Brown I, Elliott P. Sodium Intakes Around The World. Paris: World Health Organization; 2007; 85.

Cancer Institute (WIA), Chennai, Health and Family Welfare Department, Government of Tamil Nadu. India Tamil Nadu Cancer Registry 2012.

Cancer Institute (WIA), Chennai, Health and Family Welfare Department, Government of Tamil Nadu. India Tamil Nadu Cancer Registry 2013.

Cancer Institute (WIA), Chennai, International Agency for Research on Cancer (IARC). India Dindigul Ambilikkai Cancer Registry 2012.

Cancer Institute (WIA), Chennai, International Agency for Research on Cancer (IARC). India Dindigul Ambilikkai Cancer Registry 2013.

Cardiological Society of India. India - Kerala Coronary Artery Disease Risk Factors Prevalence Study 2011. [Data shared for this analysis]

Carpio A, Bharucha N, Jallon P, Beghi E, Campostrini R, Zorzetto S, Mounkoro P. Mortality of epilepsy in developing countries. Epilepsia. 2005; 46: 28-32.

Central Board of Secondary Education, Department of School Education and Literacy, Ministry of Human Resource Development, Government of India, Centres for Disease Control and Prevention, World Health Organisation (WHO). India Global School-Based Student Health Survey 2007. Geneva, Switzerland: WHO; 2007.

Central Bureau of Health Intelligence (India). Ministry of Health and Family Welfare (India). India National Health Profile 2006. New Delhi, India: Central Bureau of Health Intelligence, India, 2007.

Central Bureau of Health Intelligence (India). Ministry of Health and Family Welfare (India). India National Health Profile 2011. New Delhi, India: Central Bureau of Health Intelligence, India, 2012.

Central Bureau of Health Intelligence (India). Ministry of Health and Family Welfare (India). India National Health Profile 2013. New Delhi, India: Central Bureau of Health Intelligence, India, 2014.

Central Bureau of Health Intelligence (India), Ministry of Health and Family Welfare (India). India National Health Profile 2016. New Delhi, India: Central Bureau of Health Intelligence (India), 2016.

Central Bureau of Health Intelligence (India), Ministry of Health and Family Welfare (India). India National Health Profile 2017. New Delhi, India: Central Bureau of Health Intelligence, India, 2017.

Central Pollution Control Board (CPCB), Ministry of Environment, Forest and Climate Change, Government of India. India - National Air Quality Monitoring Programme Data 2012. New Delhi, India: CPCB.

Central Pollution Control Board (CPCB), Ministry of Environment, Forest and Climate Change, Government of India. India - National Air Quality Monitoring Programme Data 2013. New Delhi, India: CPCB.

Central Pollution Control Board (CPCB), Ministry of Environment, Forest and Climate Change, Government of India. India - National Air Quality Monitoring Programme Particulate Matter Concentration Measurements 2010. New Delhi, India: CPCB.

Central Pollution Control Board (CPCB), Ministry of Environment, Forest and Climate Change, Government of India. India - National Air Quality Monitoring Programme Particulate Matter Concentration Measurements 2011. New Delhi, India: CPCB.
Central Pollution Control Board (CPCB), Ministry of Environment, Forest and Climate Change, Government of India. India - National Air Quality Monitoring Programme Particulate Matter Concentration Measurements 2012. New Delhi, India: CPCB.
Central Pollution Control Board (CPCB), Ministry of Environment, Forest and Climate Change, Government of India. India - National Air Quality Monitoring Programme Particulate Matter Concentration Measurements 2013. New Delhi, India: CPCB.
Central Pollution Control Board (CPCB), Ministry of Environment, Forest and Climate Change, Government of India. India - National Air Quality Monitoring Programme Particulate Matter Concentration Measurements 2014. New Delhi, India: CPCB.
Central Pollution Control Board (CPCB), Ministry of Environment, Forest and Climate Change, Government of India. India - National Air Quality Monitoring Programme Particulate Matter Concentration Measurements 2015. New Delhi, India: CPCB.
Central Pollution Control Board (CPCB), Ministry of Environment, Forest and Climate Change, Government of India. India - National Air Quality Monitoring Programme Particulate Matter Concentration Measurements 2016. New Delhi, India: CPCB.
Central Pollution Control Board (CPCB), Ministry of Environment, Forest and Climate Change, Government of India. India - National Air Quality Monitoring Programme Particulate Matter Concentration Measurements 2017. New Delhi, India: CPCB.
Center for Addiction Medicine, National Institute of Mental Health and Neurosciences, Government of India, World Health Organization Collaborative Programme. Report on unrecorded consumption of alcohol in Karnataka, India. Survey 2001-2002.
Center for Addiction Medicine, National Institute of Mental Health and Neurosciences (India), Indian Council of Medical Research (ICMR). India - Andaman and Nicobar Islands Alcohol Consumption Survey 2007.
Centre for Addiction Medicine, National Institute of Mental Health and Neurosciences, Government of India, World Health Organisation Collaborative Programme. Report on Patterns and Consequences of Alcohol Misuse in India: An Epidemiological Survey 2011-2012.
Centre for Chronic Disease Control, All India Institute of Medical Sciences, Madras Diabetes Research Foundation, Public Health Foundation of India, Emory University, Aga Khan University. Centre for Cardio-Metabolic Risk Reduction in South Asia Surveillance Baseline Survey 2010-2011. [Data shared for this analysis]
Centre for Chronic Disease Control, Indian Council of Medical Research. India Diet and Lifestyle Interventions for Hypertension RisR reduction through Anganwadi Workers and Accredited Social Health Activists Baseline Study Dataset 2013-2014. [Data shared for this analysis]
Centre for Chronic Disease Control. India Prevalence of Coronary Heart Disease and its Risk Factors in Residents of Urban and Rural Areas of NCR Survey Dataset 2010-2012. [Data shared for this analysis]
Centre for Chronic Disease Control. India Prevalence of Coronary Heart Disease and its Risk Factors in Residents of Urban and Rural Areas of NCR Survey Report 2010-2012. New Delhi, India: Centre for Chronic Disease Control.
Centre for Vaccine Development, University of Maryland, Centres for Disease Control and Prevention, Department of Medical Microbiology and Immunology, Göteborg University, International Vaccine Institute, National Institute of Cholera and Enteric Diseases, Perry Point Cooperative Studies Program Coordinating Centre, U.S. Department of Veterans Affairs, School of Medicine, University of Virginia, University of Chile. India - Kolkata Global Enteric Multicentre Study 2011-2013. Baltimore, United States: Centre for Vaccine Development, University of Maryland.
Chadha SL, Gopinath N, Shekhawat S. Urban-rural differences in the prevalence of coronary heart disease and its risk factors in Delhi. Bull World Health Organ. 1997; 75: 31-8.
Chandra V, Ganguli M, Pandav R, Johnston J, Belle S, DeKosky ST. Prevalence of Alzheimer's disease and other dementias in rural India: The Indo-US study. Neurology. 1998; 51: 1000-8.
Chavan BS, Arun P, Bhargava R, Singh GP. Prevalence of alcohol and drug dependence in rural and slum population of Chandigarh: A community survey. Indian J Psychiatry. 2007; 49: 44–8.
Chengappa C, Edwards R, Bajpai R, Shields KN, Smith KR. Impact of improved cookstoves on indoor air quality in the Bundelkhand region in India. Energy Sustain Dev. 2007; 11: 33-44.
Chidambaram N, Sethupathy S, Saravanan N, Mori M, Yamori Y, Garg AK, Chockalingam A. Relationship of sodium and magnesium intakes to hypertension proven by 24-hour urianalysis in a South Indian Population. J Clin Hypertens (Greenwich). 2014; 16: 581-586.
Choudhary A, Gulati S, Sagar R, Kabra M, Sapra S. Behavioral comorbidity in children and adolescents with epilepsy. J Clin Neurosci. 2014; 21: 1337-40.
Choudhary M, Grover K, Javed M. Nutritional profiles of urban and rural men of Punjab with regard to dietary fat intake. Ecol Food Nutr. 2014; 53: 436-52.
Christian Medical College - Vellore, MRC Epidemiology Resource Centre, University of Southampton. India - Vellore Birth Cohort Study 1998-2002. [Data shared for this analysis]
Christian Medical College - Vellore. India Prevalence of Risk Factors for Non-Communicable Diseases in Rural and Urban Tamil Nadu 2010-2012. [Data shared for this analysis]
Civil Hospital, Mansa (India), Department of Health and Family Welfare, Government of Punjab (India), Postgraduate Institute of Medical Education & Research (PGIMER) (Chandigarh), Tata Memorial Center (India). India Cancer Incidence and Mortality in Mansa District, Punjab State 2013. Mumbai, India: Tata Memorial Center (India).
Civil Hospital, Sangrur (India), Department of Health and Family Welfare, Government of Punjab (India), Postgraduate Institute of Medical Education & Research (PGIMER) (Chandigarh), Tata Memorial Center (India). India Cancer Incidence and Mortality in Sangrur District, Punjab State 2013. Mumbai, India: Tata Memorial Center (India).
Curado MP, Edwards B, Shin HR, Storm H, Ferlay J, Heanue M and Boyle P, eds (2007). Cancer Incidence in Five Continents, Vol. IX Periodic Data (electronic version). Lyon: International Agency for Research on Cancer. Available from: http://ci5.iarc.fr
Dalal PM, Malik S, Bhattacharjee M, Trivedi ND, Vairale J, Bhat P, Deshmukh S, Khandelwal K, Mathur VD. Population-based stroke survey in Mumbai, India: incidence and 28-day case fatality. Neuroepidemiology. 2008; 31: 254-61.
Daniel CR, Prabhakaran D, Kapur K, Graubard BI, Devasenapathy N, Ramakrishnan L, George PS, Shetty H, Ferrucci LM, Yurgalevitch S, Chatterjee N, Reddy KS, Rastogi T, Gupta PC, Mathew A, Sinha R. A cross-sectional investigation of regional patterns of diet and cardio-metabolic risk in India. Open Nutr J. 2011; 12.
Das S, S. Neuroepidemiology of major neurological disorders in rural Bengal. Neurol India. 1996; 44: 47-58.

Das SK, Misra AK, Ray BK, Hazra A, Ghosal MK, Chaudhuri A, Roy T, Banerjee TK, Raut DK. Epidemiology of Parkinson disease in the city of Kolkata, India: a community-based study. Neurology. 2010; 75: 1362-9.
Das SK, Banerjee TK, Biswas A, Roy T, Raut DK, Mukherjee CS, Chaudhuri A, Hazra A, Roy J. A Prospective Community-Based Study of Stroke in Kolkata, India. Stroke. 2007; 38: 906-10.
Das SK, Biswas A, Roy T, Banerjee TK, Mukherjee CS, Raut DK, Chaudhuri A. A random sample survey for prevalence of major neurological disorders in Kolkata. Indian J Med Res. 2006; 124: 163-72.
Deedwania PC, Gupta R, Sharma KK, Achari V, Gupta B, Maheshwari A, Gupta A. High prevalence of metabolic syndrome among urban subjects in India: a multisite study. Diabetes Metab Syndr. 2014; 8: 156-61.
Deepa M, Anjana RM, Manjula D, Narayan KMV, Mohan V. Convergence of prevalence rates of diabetes and cardiometabolic risk factors in middle and low income groups in urban India: 10-year follow-up of the Chennai Urban Population Study. J Diabetes Sci Technol. 2011; 5: 918–27.
Deepa M, Grace M, Binukumar B, Pradeepa R, Roopa S, Khan HM, et al. High burden of prediabetes and diabetes in three large cities in South Asia: The Centre for cArdio-metabolic Risk Reduction in South Asia (CARRS) Study. Diabetes Res Clin Pract. 2015 Nov; 110: 172–82.
Department of Health and Family Welfare, Government of Punjab, Postgraduate Institute of Medical Education & Research Chandigarh, University of Michigan. India - Punjab Non-communicable Disease Risk Factors Survey 2014-2015. [Data shared for this analysis]
Department of Mental Health and Substance Abuse, World Health Organisation (WHO). Alcohol, Gender, and Drinking Problems: Perspectives from Low and Middle Income Countries 1999-2003. Geneva, Switzerland: WHO; 2005.
Department of Pulmonary Medicine, Postgraduate Institute of Medical Education & Research. India Study on Epidemiology of Asthma, Respiratory Symptoms and Chronic Bronchitis (INSEARCH) 2007-2009. [Data shared for this analysis]
Department of Pulmonary Medicine, Postgraduate Institute of Medical Education & Research. India Study on Epidemiology of Asthma, Respiratory Symptoms and Chronic Bronchitis 2004-2005. [Data shared for this analysis]
Department of Women and Child Development, Ministry of Human Resource Development, Government of India, United Nations Children's Fund (UNICEF) - India Country Office. India Summary Report on the Multiple Indicator Cluster Survey 2000. New Delhi, India: UNICEF India Country Office; 2000.
Department of Women and Child Development, Ministry of Human Resource Development, Government of India. India Nutrition Profile Survey 1995-1996. New Delhi, India: Ministry of Human Resource Development.
Desai, Sonalde, Reeve Vanneman, National Council of Applied Economic Research, University of Michigan. India Human Development Survey II, 2011-12. Ann Arbor, Michigan: Inter-University Consortium for Political and Social Research.
Deshmukh V, Lahariya C, Krishnamurthy S, Das MK, Pandey RM, Arora NK. Taken to Health Care Provider or Not, Under-Five Children Die of Preventable Causes: Findings from Cross-Sectional Survey and Social Autopsy in Rural India. Indian J Community Med. 2016; 41: 108–19.
Dhamija RK, Dhamija SB. Prevalence of stroke in rural community – an overview of Indian experience. J Assoc Physicians India. 1998; 46: 351-4.
Directorate of Economics & Statistics and Office of Chief Registrar (Births & Deaths), Government of National Capital Territory of Delhi. Report on Medical Certification of Cause of Deaths in Delhi-2011. New Delhi, India: Directorate of Economics & Statistics and Office of Chief Registrar (Births & Deaths); 2012.
Directorate of Economics & Statistics and Office of Chief Registrar (Births & Deaths), Government of National Capital Territory of Delhi. Report on Medical Certification of Cause of Deaths in Delhi-2012. New Delhi, India: Directorate of Economics & Statistics and Office of Chief Registrar (Births & Deaths); 2014.
Directorate of Economics & Statistics and Office of Chief Registrar (Births & Deaths), Government of National Capital Territory of Delhi. Report on Medical Certification of Cause of Deaths in Delhi-2013. New Delhi, India: Directorate of Economics & Statistics and Office of Chief Registrar (Births & Deaths); 2014.
Dogra V, Khanna R, Jain A, Kumar AMV, Shewade HD, Majumdar SS. Neonatal mortality in India's rural poor: Findings of a household survey and verbal autopsy study in Rajasthan, Bihar and Odisha. J Trop Pediatr. 2015; 61: 210–4.
Dutta K, Shields KN, Edwards R, Smith KR. Impact of improved biomass cookstoves on indoor air quality near Pune, India. Energy Sustain Dev. 2007; 11: 19-32.
Dworetzky BA, Bromfield EB, Townsend MK, Kang JH. A prospective study of smoking, caffeine, and alcohol as risk factors for seizures or epilepsy in young adult women: data from the Nurses' Health Study II. Epilepsia 2010; 51: 198–205.
Dyson PA, Anthony D, Fenton B, Matthews DR, Stevens DE, Community Interventions for Health Collaboration. High rates of child hypertension associated with obesity: a community survey in China, India and Mexico. Paediatr Int Child Health. 2014; 34: 43–9.
Embassy of the United States - New Delhi, United States Environmental Protection Agency. United States Mission India NowCast Air Quality Data 2014. New Delhi, India: Embassy of the United States.
Esser MB, Gururaj G, Rao GN, Jernigan DH, Murthy P, Jayarajan D, Lakshmanan S, Benegal V, Collaborators Group on Epidemiological Study of Patterns and Consequences of Alcohol Misuse in India. Harms to Adults from Others' Heavy Drinking in Five Indian States. Alcohol Alcohol. 2016; 51: 177–85.
Euromonitor International. Euromonitor Passport - Cigarette Statistics 1999-2016. London, United Kingdom: Euromonitor International.
Euromonitor International. Euromonitor Passport - Alcoholic Drinks Statistics India. September 2020. London, United Kingdom: Euromonitor International.
Euromonitor International. Euromonitor Passport - Fresh Foods in India. London, United Kingdom: Euromonitor International.
European Centre for Medium-Range Weather Forecasts: European Centre for Medium-Range Weather Forecasts (ECMWF) Re-Analysis 5 (ERA5) model data. Centre for Environmental Data Analysis.
FAO Supply Utilization Accounts 1961-2013. Personal Correspondence with Dr. Josef Schmidhuber, 2016. [Data shared for this analysis]
Fall CHD, Sachdev HS, Osmond C, Lakshmy R, Biswas SD, Prabhakaran D, Tandon N, Ramji S, Reddy KS, Barker DJP, Bhargava SK. Adult metabolic syndrome and impaired glucose tolerance are associated with different patterns of BMI gain during infancy: Data from the New Delhi Birth Cohort. Diabetes Care. 2008; 31: 2349-56.
Feenstra RC, Inklaar R, Marcel P. Timmer MP. The Next Generation of the Penn World Table. American Economic Review. 2015; 105: 3150-82.

Ferlay J, Parkin DM, Curado MP, Bray F, Edwards B, Shin HR and Forman D. Cancer Incidence in Five Continents Time Trends, Volumes I to X and Volume XI: IARC CancerBase No. 9 [Internet]. Lyon, France: International Agency for Research on Cancer; 2010. Available from: http://ci5.iarc.fr

Food and Agriculture Organisation of the United Nations (FAO). FAOSTAT Commodity Balances - Crops Primary Equivalent. Rome, Italy: FAO.

Food and Agriculture Organisation of the United Nations (FAO). FAOSTAT Food Balance Sheets, October 2014. Rome, Italy: FAO.

Forman D, Bray F, Brewster DH, Gombe Mbalawa C, Kohler B, Piñeros M, Steliarova-Foucher E, Swaminathan R, Ferlay J, eds. Cancer Incidence in Five Continents, Vol. X. International Agency for Research on Cancer (IARC) Scientific Publications, No. 164. Lyon, France: IARC; 2014.

Gajalakshmi V, Peto R, Kanimozhi VC, Whitlock G, Veeramani D. Cohort Profile: The Chennai prospective study of mortality among 500,000 adults in Tamil Nadu, South India. Int J Epidemiol. 2007; 36: 1190-5.

Gallup. India World Poll 2009. https://www.gallup.com/analytics/318923/world-poll-public-datasets.aspx

Gallup. India World Poll 2011.https://www.gallup.com/analytics/318923/world-poll-public-datasets.aspx

Gallup. India World Poll 2012. https://www.gallup.com/analytics/318923/world-poll-public-datasets.aspx

Geetha L, Deepa M, Anjana RM, Mohan V. Prevalence and clinical profile of metabolic obesity and phenotypic obesity in Asian Indians. J Diabetes Sci Technol. 2011; 5: 439–46.

Gemmy Cheung CM, Li X, Cheng C-Y, Zheng Y, Mitchell P, Wang JJ, Jonas JB, Nangia V, Wong TY. Prevalence and risk factors for age-related macular degeneration in Indians: a comparative study in Singapore and India. Am J Ophthalmol. 2013; 155: 764–73.

George K, Prasad J, Singh D, Minz S, Albert DS, Muliyil J, Joseph KS, Jayaraman J, Kramer MS. Perinatal outcomes in a South Asian setting with high rates of low birthweight. BMC Pregnancy Childbirth. 2009; 5.

Ghosh A, Bala SK. Anthropometric, body composition, and blood pressure measures among rural elderly adults of Asian Indian origin: the Santiniketan aging study. J Nutr Gerontol Geriatr. 2011; 30: 305–13.

Ghosh R, Sharma AK. Determinants of tetanus and sepsis among the last neonatal deaths at household level in a peri-urban area of India. Postgrad Med J. 2011; 87: 257-63.

Ghosh S, Samanta A, Mukherjee S. Patterns of alcohol consumption among male adults at a slum in Kolkata, India. J Health Popul Nutr. 2012; 30: 73–81.

Global Burden of Disease Health Financing Collaborator Network, Institute for Health Metrics and Evaluation (IHME). Global Development Assistance for Health, Government, Prepaid Private, and Out-of-Pocket Health Spending 1995-2014. Seattle, United States: IHME; 2017.

Goel D, Agarwal A, Dhanai JS, Semval VD, Mehrotra V, Saxena V, Maithili B. Comprehensive rural epilepsy surveillance programme in Uttarakhand state of India. Neurol India. 2009; 57: 355–6.

Goel D, Dhanai JS, Agarwal A, Mehlotra V, Saxena V. Neurocysticercosis and its impact on crude prevalence rate of epilepsy in an Indian community. Neurol India. 2011; 59: 37-40.

Goswami AK, Gupta SK, Kalaivani M, Nongkynrih B, Pandav CS. Burden of Hypertension and Diabetes among Urban Population Aged ≥ 60 years in South Delhi: A Community Based Study. J Clin Diagn Res. 2016; 10: LC01-05.

Goudar SS, Goco N, Somannavar MS, Vernekar SS, Mallapur AA, Moore JL, Wallace DD, Sloan NL, Patel A, Hibberd PL, Koso-Thomas M, McClure EM, Goldenberg RL. Institutional deliveries and perinatal and neonatal mortality in Southern and Central India. Reprod Health. 2015; 12: S13.

Gourie-Devi M, Gururaj G, Satishchandra P, Subbakrishna DK. Neuro-epidemiological pilot survey of an urban population in a developing country. A study in Bangalore, south India. Neuroepidemiology. 1996; 15: 313-20.

Gourie-Devi M, Gururaj G, Satishchandra P, Subbakrishna DK. Prevalence of neurological disorders in Bangalore, India: a community-based study with a comparison between urban and rural areas. Neuroepidemiology. 2004; 23: 261-8.

Government of India, IPE Global Limited, Kantar Public. India National Annual Rural Sanitation Survey 2018-2019.

Government of India, Ministry of Health and Family Welfare (India), National Health Mission (India), Postgraduate Institute of Medical Education & Research (PGIMER) (Chandigarh). India - Haryana STEPS Noncommunicable Disease Risk Factors Survey 2016-2017.

Government of India, Ministry of Statistics and Programme Implementation (India). India National Sample Survey Round 71 2014. New Delhi, India: Ministry of Statistics and Programme Implementation (India).

Government of India. India Ground Monitor Station PM2.5 Data 2012.

Gupta A, Gupta R, Sarna M, Rastogi S, Gupta VP, Kothari K. Prevalence of diabetes, impaired fasting glucose and insulin resistance syndrome in an urban Indian population. Diabetes Res Clin Pract. 2003; 61: 69-76.

Gupta D, Boffetta P, Gaborieau V, Jindal SK. Risk factors of lung cancer in Chandigarh, India. Indian J Med Res. 2001; 113: 142-50.

Gupta PC, Subramoney S, Sreevidya S. Smokeless tobacco use, birthweight, and gestational age: population based, prospective cohort study of 1217 women in Mumbai, India. BMJ. 2004; 328: 1538.

Gupta R, Deedwania PC, Achari V, Asirvatham AJ, Bhansali A, Gupta A, Gupta B, Gupta S, Jali MV, Mahanta TG, Maheshwari A, Saboo B, Singh J. India Heart Watch Study 2005-2009. [Data shared for this analysis]

Gupta R, Misra A, Vikram NK, Kondal D, Gupta SS, Agrawal A, Pandey RM. Younger age of escalation of cardiovascular risk factors in Asian Indian subjects. BMC Cardiovasc Disord. 2009; 9:28.

Gupta R, Pandey RM, Misra A, Agrawal A, Misra P, Dey S, Rao S, Menon VU, Kamalamma N, Vasantha Devi KP, Revathi K, Vikram NK, Sharma V, Guptha S. High prevalence and low awareness, treatment and control of hypertension in Asian Indian women. J Hum Hypertens. 2012; 26: 585–93.

Gupta R, Sharma KK, Gupta BK, Gupta A, Saboo B, Maheshwari A, Mahanta T, Deedwania PC. Geographic epidemiology of cardiometabolic risk factors in middle class urban residents in India: cross sectional study. J Glob Health. 2015; 5: 010411.

Gupta R, Sharma AK, Gupta VP, Bhatnagar S, Rastogi S, Deedwania PC. Increased variance in blood pressure distribution and changing hypertension prevalence in an urban Indian population. J Hum Hypertens. 2003; 17: 535-40.

Gupta R. India - Jaipur Heart Watch Study Data on Blood Pressure, Cholesterol, BMI, and Fasting Blood Glucose 1993-2001. [Data shared for this analysis]
Gurukartick J, Dongre AR, Shah D. Social Determinants of Dementia and Caregivers' Perspectives in the Field Practice Villages of Rural Health Training Centre, Thiruvennainallur. Indian J Palliat Care. 2016; 22: 25-32.
Hackett RJ, Hackett L, Bhakta P. The prevalence and associated factors of epilepsy in children in Calicut District, Kerala, India. Acta Paediatr. 1997; 86: 1257-60.
Hara HS, Gupta A, Singh M, Raj R, Singh H, Pawar G, Hara PK, Singh J. Epilepsy in Punjab (India): A Population-Based Epidemiologic Study. Neuroepidemiology. 2015; 45: 273-81.
Healis-Sekhsaria Institute for Public Health, Madhya Pradesh Voluntary Health Association, Cancer Foundation of India, School of Preventive Oncology, University of Waterloo. International Tobacco Control Policy Evaluation Project: India Tobacco Control Survey 2010-2011. Navi Mumbai, India and Waterloo, Canada: Healis-Sekhsaria Institute for Public Health and University of Waterloo.
Health Department, Chandigarh Administration, Postgraduate Institute of Medical Education & Research (PGIMER) (Chandigarh), Tata Memorial Center (India). India Cancer Incidence and Mortality in Chandigarh Union Territory 2013. Chandigarh, India: Postgraduate Institute of Medical Education & Research (PGIMER) (Chandigarh).
Health Management Information System (HMIS), National Health Mission, Ministry of Health and Family Welfare, Government of India. India Performance Related to Immunisation 2007-2009. New Delhi, India: Ministry of Health and Family Welfare.
Health Management Information System (HMIS), National Health Mission, Ministry of Health and Family Welfare, Government of India. India Performance Related to Immunisation 2008-2010. New Delhi, India: Ministry of Health and Family Welfare.
Health Management Information System (HMIS), National Health Mission, Ministry of Health and Family Welfare, Government of India. India Performance Related to Immunisation 2009-2011. New Delhi, India: Ministry of Health and Family Welfare.
Health Management Information System (HMIS), National Health Mission, Ministry of Health and Family Welfare, Government of India. India Performance Related to Immunisation 2010-2012. New Delhi, India: Ministry of Health and Family Welfare.
Health Management Information System (HMIS), National Health Mission, Ministry of Health and Family Welfare, Government of India. India Performance Related to Immunisation 2011-2013. New Delhi, India: Ministry of Health and Family Welfare.
Health Management Information System (HMIS), National Health Mission, Ministry of Health and Family Welfare, Government of India. India Performance Related to Immunisation 2012-2014. New Delhi, India: Ministry of Health and Family Welfare.
Health Management Information System (HMIS), National Health Mission, Ministry of Health and Family Welfare, Government of India. India Performance Related to Immunisation 2013-2015. New Delhi, India: Ministry of Health and Family Welfare.
Health Management Information System (HMIS), National Health Mission, Ministry of Health and Family Welfare, Government of India. India Performance Related to Immunisation 2014-2016. New Delhi, India: Ministry of Health and Family Welfare.
Health Management Information System (HMIS), National Health Mission, Ministry of Health and Family Welfare, Government of India. India Performance Related to Immunisation 2015-2017. New Delhi, India: Ministry of Health and Family Welfare.
Huffman MD, Prabhakaran D, Osmond C, Fall CHD, Tandon N, Lakshmy R, Ramji S, Khalil A, Gera T, Prabhakaran P, Biswas SKD, Reddy KS, Bhargava SK, Sachdev HS, New Delhi Birth Cohort. Incidence of cardiovascular risk factors in an Indian urban cohort results from the New Delhi birth cohort. J Am Coll Cardiol. 2011; 57: 1765-74.
Indian Council of Medical Research (ICMR), Madras Diabetes Research Foundation. Indian Council of Medical Research India Diabetes Study (ICMR-INDIAB) - North East 2012-2015. [Data shared for this analysis]
Indian Council of Medical Research (ICMR), Madras Diabetes Research Foundation. Indian Council of Medical Research India Diabetes Study (ICMR-INDIAB) 2008-2010. [Data shared for this analysis]
Indian Council of Medical Research (ICMR), Madras Diabetes Research Foundation. Indian Council of Medical Research India Diabetes Study (ICMR-INDIAB) 2012-2013. [Data shared for this analysis]
Indian Council of Medical Research (ICMR). India Study on Causes of Death by Verbal Autopsy 2003. New Delhi, India: ICMR. [Data shared for this analysis]
Indian Council of Medical Research, World Health Organisation. India STEPS Noncommunicable Disease Risk Factors Survey 2003-2005. [Data shared for this analysis]
Indian Institute of Health Management Research, International Institute of Population Sciences, Ministry of Health and Family Welfare, Government of India, Performance Monitoring and Accountability 2020 (PMA2020) Project, Bill & Melinda Gates Institute for Population and Reproductive Health, Johns Hopkins Bloomberg School of Public Health. PMA 2016 Rajasthan Round 1 Survey Data as part of PMA 2020 Project. Baltimore, United States: PMA 2020.
Indian Institute of Tropical Metereology, Ministry of Earth Sciences (India). India System of Air Quality and Weather Forecasting And Research Air Quality Index 2015. Pune, India: Indian Institute of Tropical Metereology.
Indian Institute of Tropical Metereology, Ministry of Earth Sciences (India). India System of Air Quality and Weather Forecasting And Research Air Quality Index 2016. Pune, India: Indian Institute of Tropical Metereology.
Institute for Health Metrics and Evaluation (IHME). IHME GBD Cancer Incidence and Mortality Estimates. 1990-2019
Institute for Health Metrics and Evaluation (IHME). IHME GBD India Medical Certification of Cause of Death Estimates by State 2007.
Institute of Health Systems, World Health Organisation (WHO). WHO Multi-country Survey Study Report on Health and Health System Responsiveness, Andhra Pradesh 2000-2001.
Institute of Health Systems. India Cause of Death Dataset Version 1.3 1980-1998. Hyderabad, India: Institute of Health Systems; 2002.
Institute of Social Medicine and Health Policy, Shandong University, Shandong University School of Medicine, World Health Organization (WHO). China WHO Multi-country Survey Study on Health and Health System Responsiveness 2000-2001.
International Agency for Research on Cancer (IARC), International Association of Cancer Registries (IACR), World Health Organization (WHO). International Incidence of Childhood Cancer Volume III. Lyon, France: International Agency for Research on Cancer (IARC), 2017.
International Institute for Population Sciences (IIPS), Ministry of Health and Family Welfare, Government of India. Concurrent Evaluation of National Rural Health Mission 2009. Mumbai, India: IIPS

International Institute for Population Sciences (India), Population Council (India). India Youth Situation and Needs Study 2006-2008.

International Institute for Population Sciences, Ministry of Health and Family Welfare, Government of India. India District Level Household and Facility Survey Data (DLHS) 2002-2005. Mumbai, India: IIPS.

International Institute for Population Sciences (IIPS), Ministry of Health and Family Welfare, Government of India, Harvard T. H. Chan School of Public Health, RAND Corporation, Monash University, University of California Los Angeles, Indian Academy of Geriatrics, National AIDS Research Institute, Columbia University. Longitudinal Aging Study in India, Pilot Data 2010. Mumbai, India: IIPS.

International Institute for Population Sciences (IIPS), Ministry of Health and Family Welfare, Government of India, ICF. India National Family Health Survey (NFHS-4) 2015-2016: National and State-level Factsheets. Mumbai, India: IIPS.

International Institute for Population Sciences (IIPS), Ministry of Health and Family Welfare, Government of India, Macro International. India National Family Health Survey (NFHS-1) 1992-1993: National Report. Mumbai, India: IIPS.

International Institute for Population Sciences (IIPS), Ministry of Health and Family Welfare, Government of India, Macro International. India National Family Health Survey (NFHS-3) 2005-2006: National and State Reports. Mumbai, India: IIPS.

International Institute for Population Sciences (IIPS), Ministry of Health and Family Welfare, Government of India, Macro International. India National Family Health Survey Data (NFHS-1) 1992-1993. Mumbai, India: IIPS.

International Institute for Population Sciences (IIPS), Ministry of Health and Family Welfare, Government of India, Macro International. India National Family Health Survey Data (NFHS-3) 2005-2006. Mumbai, India: IIPS.

International Institute for Population Sciences (IIPS), Ministry of Health and Family Welfare, Government of India, ORC Macro. India National Family Health Survey (NFHS-2) 1998-1999: National and State Reports. Mumbai, India: IIPS.

International Institute for Population Sciences (IIPS), Ministry of Health and Family Welfare, Government of India, ORC Macro. India National Family Health Survey Data (NFHS-2) 1998-1999. Mumbai, India: IIPS.

International Institute for Population Sciences (IIPS), Ministry of Health and Family Welfare, Government of India. India District Level Household and Facility Survey (DLHS-3) 2007-2008: National and State Reports. Mumbai, India: IIPS.

International Institute for Population Sciences (IIPS), Ministry of Health and Family Welfare, Government of India. India District Level Household and Facility Survey Data (DLHS-3) 2007-2008. Mumbai, India: IIPS.

International Institute for Population Sciences (IIPS), Ministry of Health and Family Welfare, Government of India. India District Level Household and Facility Survey Data (DLHS-4) 2012-2013: State Reports. Mumbai, India: IIPS.

International Institute for Population Sciences (IIPS), Ministry of Health and Family Welfare, Government of India. India District Level Household Survey (DLHS-1) 1998-1999: National Report. Mumbai, India: IIPS.

International Institute for Population Sciences (IIPS), Ministry of Health and Family Welfare, Government of India. India District Level Household Survey Data (DLHS-1) 1998-1999. Mumbai, India: IIPS.

International Institute for Population Sciences, Ministry of Health and Family Welfare, Government of India. India District Level Household and Facility Survey Data (DLHS-4) 2012-2013. Mumbai, India: IIPS.

International Institute for Population Sciences, World Health Organisation (WHO). India WHO Study on Global Ageing and Adult Health 2007-2008.

International Institute for Population Sciences, World Health Organisation. India World Health Survey 2003.

International Monetary Fund (IMF). World Economic Outlook Database. Washington, D.C., United States of America: International Monetary Fund (IMF).

International Society of Nephrology (ISN), Tamil Nadu Kidney Research Foundation. India - Chennai International Society of Nephrology Kidney Disease Data Center Chronic Kidney Disease and Cardiovascular Risk Survey 2014-2015.

Intersalt: an international study of electrolyte excretion and blood pressure. Results for 24 hour urinary sodium and potassium excretion. Intersalt Cooperative Research Group. BMJ. 1988; 297: 319-328.

Invasive Bacterial Infections Surveillance (IBIS) Group of the International Clinical Epidemiology Network. Are Haemophilus influenzae infections a significant problem in India? A prospective study and review. Clin Infect Dis. 2002; 34: 949-57.

IPE Global Limited, Kantar Public. India National Annual Rural Sanitation Survey 2017-2018.

International Social Survey Programme (ISSP), GESIS - Leibniz Institute of Social Sciences. ISSP: Family and Changing Gender Roles IV, ZA5900 (Version 4.0.0). Mannheim, Germany: GESIS - Leibniz Institute of Social Sciences; 2012. https://dbk.gesis.org/dbksearch/sdesc2.asp?no=5900&db=e&doi=10.4232/1.12661

International Social Survey Programme (ISSP), GESIS - Leibniz Institute of Social Sciences. ISSP: National Identity III, ZA5950 Data file (Version 2.0.0). Mannheim, Germany: GESIS - Leibniz Institute of Social Sciences; 2013. https://dbk.gesis.org/dbksearch/sdesc2.asp?no=5950&db=e

International Social Survey Programme (ISSP), GESIS - Leibniz Institute of Social Sciences. ISSP: Citizenship II, ZA5900 Data file ZA6670 Data file (Version 4.0.0). Mannheim, Germany: GESIS - Leibniz Institute of Social Sciences; 2014. https://dbk.gesis.org/dbksearch/sdesc2.asp?no=6670&db=e&notabs=1

Jayaraman R, Varghese R, Kumar JL, Neeravi A, Shanmugasundaram D, Ralph R, Thomas K, Veeraraghavan B. Invasive pneumococcal disease in Indian adults: 11 years' experience. J Microbiol Immunol Infect. 2018; 1-7.

Johnson C, Mohan S, Rogers K, Shivashankar R, Thout SR, Gupta P, He FJ, MacGregor GA, Webster J, Krishnan A, Maulik PK, Reddy KS, Prabhakaran D, Neal B. Mean Dietary Salt Intake in Urban and Rural Areas in India: A Population Survey of 1395 Persons. J Am Heart Assoc. 2017; 6: e004547

Johnson C, Praveen D, Pope A, Raj TS, Pillai RN, Land MA, Neal B. Mean population salt consumption in India: a systematic review. J Hypertens. 2017; 35: 3-9.

Joseph N, Kumar GS, Nelliyanil M. Pattern of seizure cases in tertiary care hospitals in Karnataka state of India. Ann Indian Acad Neurol. 2013; 16: 347-51.

Joshi R, Cardona M, Iyengar S, Sukumar A, Raju CR, Raju KR, Raju K, Reddy KS, Lopez A, Neal B. Chronic diseases now a leading cause of death in rural India’s mortality data from the Andhra Pradesh Rural Health Initiative. Int J Epidemiol. 2006; 35: 1522-9.

Kalra S, Kalra B, Sharma A. Prevalence of type 1 diabetes mellitus in Karnal district, Haryana state, India. Diabetol Metab Syndr. 2010; 14.
Kalkonde Y, Deshmukh M, Kakarmath S, Puthran J, Agavane V, Sahane V, Bang A. A prospective study of causes of death in rural Gadchiroli, an underdeveloped district of India (2011-2013). J Glob Health Rep. 2019; 3: e2019009.
Kalkonde YV, Sahane V, Deshmukh MD, Nila S, Mandava P, Bang A. High Prevalence of Stroke in Rural Gadchiroli, India: A Community-Based Study. Neuroepidemiology. 2016; 46: 235-9.
Kamble P, Deshmukh PR, Garg N. Metabolic syndrome in adult population of rural Wardha, central India. Indian J Med Res. 2010; 132: 701–5.
Kameswaran C, Bhatia BD, Bhat BV, Oumachigui A. Perinatal mortality: a hospital based study. Indian Pediatr. 1993; 30: 997-1001.
Katyal R, Bansal R, Agrawal V, Goel K, Chaudhary V. Cross-sectional Study to Acknowledge the Independent Association of the Socio-demographic Determinants of Alcohol Use in an Urban Slum of North India. Int J Prev Med. 2014; 5: 749–57.
Kaur S, Sachdev HPS, Dwivedi SN, Lakshmy R, Kapil U. Prevalence of overweight and obesity amongst school children in Delhi, India. Asia Pac J Clin Nutr. 2008; 17: 592-6.
Kaushik SL, Parmar VR, Grover N, Kaushik R. Neonatal mortality rate: relationship to birthweight and gestational age. Indian J Pediatr. 1998; 65(3): 429-33.
Kayina KP, Sharma AK, Agrawal K. Implementation of ICD 10: a study on the doctors knowledge and coding practices in Delhi. Indian J Public Health. 2015; 59(1): 68-9.
KEM Hospital Research Center. India - Pimpale Cardiovascular Risk Factors Study Blood Pressure, Cholesterol, BMI, Blood Glucose, and Diabetes Incidence Measurements 1994-1999. [Data shared for this analysis]
Khandait DW, Ambadekar NN, Zodpey SP, Vasudeo ND. Maternal age as a risk factor for stillbirth. Indian J Public Health. 2000; 44: 28-30.
Kim S, Rifkin S, John SM, Jacob KS. Nature, prevalence and risk factors of alcohol use in an urban slum of Southern India. Natl Med J India. 2013; 26: 203–9.
King George's Medical University (India). India - Lucknow King George's Medical University Inpatient Data 2017.
Kodkany BS, Derman RJ, Honnungar NV, Tyagi NK, Goudar SS, Mastiholi SC, Moore JL, McClure EM, Sloan N, Goldenberg RL. Establishment of a Maternal Newborn Health Registry in the Belgaum District of Karnataka, India. Reprod Health. 2015; 12: S3.
Kokkat AJ, Verma AK. Prevalence of seizures and paralysis in a rural community. J Indian Med Assoc. 1998; 96: 43-5.
Kolappan C, Subramani R, Swaminathan S. Tuberculosis mortality in a rural population from South India. Indian J Tuberc. 2016; 63: 100–5.
Koul R, Motta A, Razdan S. Epidemiology of young strokes in rural Kashmir, India. Acta Neurol Scand. 1990; 82: 1-3.
Koul R, Razdan S, Motta A. Prevalence and pattern of epilepsy (Lath/Mirgi/Laran) in rural Kashmir, India. Epilepsia. 1988; 29: 116-22.
Krishnan MN, Zachariah G, Venugopal K, Mohanan PP, Harikrishnan S, Sanjay G, Jeyaseelan L, Thankappan KR. Prevalence of coronary artery disease and its risk factors in Kerala, South India: a community-based cross-sectional study. BMC Cardiovasc Disord. 2013; 16: 12.
Kulkarni GB, Rao GN, Gururaj G, Stovner LJ, Steiner TJ. Headache disorders and public ill-health in India: prevalence estimates in Karnataka State. J Headache Pain. 2015; 16: 67
Kumar P. Characterisation of indoor respirable dust in a locality of Delhi; India. Indoor Built Environ. 2001; 10: 95-102.
Kumar P, Krishna P, Reddy SC, Gurappa M, Aravind SR, Munichoodappa C. Incidence of type 1 diabetes mellitus and associated complications among children and young adults: results from Karnataka Diabetes Registry 1995-2008. J Indian Med Assoc. 2008; 106: 708–11.
Kumar R, Bhave A, Bhargava R, Agarwal GG. Prevalence and risk factors for neurological disorders in children aged 6 months to 2 years in northern India. Dev Med Child Neurol. 2013; 55: 348-56.
Kumar R, Kapoor SK, Krishnan A. Performance of cause-specific childhood mortality surveillance by health workers using a short verbal autopsy tool. Southeast Asia J Public Health. 2012; 1: 151-158
Kumar R. Anthropometric and behavioral risk factor for noncommunicable diseases: a cluster survey from rural Wardha. Indian J Public Health. 2015; 59: 61.
Kumar R, Singh K, Nagar S, Kumar M, Mehto UK, Rai G, Gupta N. Pollutant Levels at Cooking Place and Their Association with Respiratory Symptoms in Women in a Rural Area of Delhi-NCR. Indian J Chest Dis Allied Sci. 2015; 57: 225-31.
Kumar S, Awasthi S, Jain A, Srivastava RC. Blood zinc levels in children hospitalized with severe pneumonia: a case control study. Indian Pediatr. 2004; 41: 486-91.
Kumar SG, Premarajan KC, Subitha L, Suguna E, Vinayagamoorthy, Kumar V. Prevalence and Pattern of Alcohol Consumption using Alcohol Use Disorders Identification Test (AUDIT) in Rural Tamil Nadu, India. J Clin Diagn Res. 2013; 7: 1637-9.
Kumar V, Datta N, Wadhwa SS, Singhi S. Morbidity and mortality in diarrhea in rural Haryana Indian. Indian J Pediatr. 1985; 52: 455-61.
Kumar V, Garg BS. Global health and infant mortality: application of verbal autopsy tool to categorize infant deaths, ascertain their causes and identify the gaps in health management information system in India. Int J Curr Res Rev. 2014; 5: 87-94.
Kusuma Y, Das P. Hypertension in Orissa, India: a cross-sectional study among some tribal, rural and urban populations. Public Health. 2008; 122: 1120-3.
Kusuma Y, Gupta S, Pandav C. Migration and hypertension: a cross-sectional study among neo-migrants and settled-migrants in Delhi, India. Asia Pac J Public Health. 2009; 21: 497-507.
Kusuma YS, Babu BV, Naidu JM. Blood pressure levels among cross-cultural populations of Visakhapatnam district, Andhra Pradesh, India. Ann Hum Biol. 2002; 29: 502-12.
Lear SA, Hu W, Rangarajan S, Gasevic D, Leong D, Iqbal R, Casanova A, Swaminathan S, Anjana RM, Kumar R, Rosengren A, Wei L, Yang W, Chuangshi W, Huaxing L, Nair S, Diaz R, Swidon H, Gupta R, Mohammadifard N, Lopez-Jaramillo P, Oguz A, Zatonska K, Seron P, Avezum A, Poirier P, Teo K, Yusuf S. The effect of physical activity on mortality and cardiovascular disease in 130â€ˆ000 people from 17 high-income, middle-income, and low-income countries: the PURE study. Lancet. 2017; 390: 2643-2654.
Leavey A, Londeree J, Priyadarshini P, Puppala J, Schechtman KB, Yadama G, Biswas P. Real-Time Particulate and CO Concentrations from Cookstoves in Rural Households in Udaipur; India. Environ Sci Technol. 2015; 49(12): 7423-31.

Li X, Li W, Liu G, Shen X, Tang Y. Association between cigarette smoking and Parkinson’s disease: a meta-analysis. Arch Gerontol Geriatr. 2015; 61:510–6.
Llibre Rodriguez JJ, Ferri CP, Acosta D, Guerra M, Huang Y, Jacob KS, Krishnamoorthy ES, Salas A, Sosa AL, Acosta I, Dewey ME, Gaona C, Jotheeswaran AT, Li S, Rodriguez D, Rodriguez G, Kumar PS, Valhuerdi A, Prince M, 10/66 Dementia Research Group. Prevalence of dementia in Latin America, India, and China: a population-based cross-sectional survey. Lancet. 2008; 372: 464-74.
London School of Hygiene and Tropical Medicine. Indian Migration Study 2005-2007.
Bolt J, Inklaar R, De Jong H, Van Zanden JL. Rebasing ‘Maddison’: new income comparisons and the shape of long-run economic development. GGDC Research Memorandum. 2018; 174: 1-67.
Madras Diabetes Research Foundation & M. V. Diabetes Specialities Centre. India - Chennai Urban Population Study Blood Glucose, Cholesterol, BMI, and Diabetes Incidence Measurements, 1996-2006. [Data shared for this analysis]
Madras Diabetes Research Foundation & M. V. Diabetes Specialities Centre. India - Chennai Urban Rural Epidemiology Study Blood Glucose, Cholesterol, BMI, and Diabetes Incidence Measurements, 2001-2013. [Data shared for this analysis]
Mahanta TG, Joshi R, Mahanta BN, Xavier D. Prevalence of modifiable cardiovascular risk factors among tea garden and general population in Dibrugarh, Assam, India. J Epidemiol Glob Health. 2013; 3: 147–56.
Mahalanabis D, Gupta S, Paul D, Gupta A, Lahiri M, Khaled MA. Risk factors for pneumonia in infants and young children and the role of solid fuel for cooking: a case-control study. Epidemiol Infect. 2002; 129: 65-71.
Mahavarkar SH, Madhu CK, Mule VD. A comparative study of teenage pregnancy. J Obstet Gynaecol. 2008; 28: 604-7.
Mahesh PA, Jayaraj BS, Chaya SK, Lokesh KS, McKay AJ, Prabhakar AK, Pape UJ. Variation in the prevalence of chronic bronchitis among smokers: a cross-sectional study. Int J Tuberc Lung Dis. 2014; 18: 862-9.
Malhotra P, Kumari S, Kumar R, Jain S, Sharma BK. Prevalence and determinants of hypertension in an un-industrialised rural population of North India. J Hum Hypertens. 1999; 13: 467-72.
Mani KS, Rangan G, Srinivas HV, Kalyanasundaram S, Narendran S, Reddy AK. The Yelandur study: a community-based approach to epilepsy in rural South India – epidemiological aspects. Seizure. 1998; 7: 281-8.
Massey D, Masih J, Kulshrestha A, Habil M, Taneja A. Indoor/outdoor relationship of fine particles less than 2.5 mm (PM2.5) in residential homes locations in central Indian region. Build Environ. 2009; 44: 2037-45.
Mathuranath PS, Cherian PJ, Mathew R, Kumar S, George A, Alexander A, Ranjith N, Sarma PS. Dementia in Kerala, South India: prevalence and influence of age, education and gender. Int J Geriatr Psychiatry. 2010; 25: 290-7.
Matsuoka H, Ishii A, Panjaitan W, Sudiranto R. Malaria and glucose-6-phosphate dehydrogenase deficiency in North Sumatra, Indonesia. Southeast Asian J Trop Med Public Health. 1986; 17: 530-6.
McCleary R, Shankle WR, Mulnard RA, Dick MB. Ishihara test performance and dementia. J Neurol Sci. 1996; 142: 93-8.
McClure EM, Pasha O, Goudar SS, Chomba E, Garces A, Tshefu A, Althabe F, Esamai F, Patel A, Wright LL, Moore J, Kodkany BS, Belizan JM, Saleem S, Derman RJ, Carlo WA, Hambidge KM, Buekens P, Liechty EA, Bose C, Koso-Thomas M, Jobe AH, Goldenberg RL; Global Network Investigators. Epidemiology of stillbirth in low-middle income countries: A Global Network Study. Acta Obstet Gynecol Scand. 2011; 90: 1379-85.
Mehta P, Joseph A, Verghese A. An epidemiologic study of psychiatric disorders in a rural area in Tamilnadu. Indian J Psychiatry. 1985; 27: 153-8.
Midha T, Idris MZ, Saran RK, Srivastav AK, Singh SK. Prevalence and determinants of hypertension in the urban and rural population of a north Indian district. East Afr J Public Health. 2009; 6: 268–73.
Ministry of Health and Family Welfare (India). India Family Welfare Statistics 2011. New Delhi, India: Ministry of Health and Family Welfare (India), 2011.
Ministry of Health and Family Welfare, Government of India, International Institute for Population Sciences, Centres for Disease Control and Prevention (CDC), Johns Hopkins Bloomberg School of Public Health, Research Triangle Institute, Inc., World Health Organisation. India Global Adult Tobacco Survey 2009-2010. Atlanta, United States: CDC.
Ministry of Health and Family Welfare, Government of India, United Nations Children's Fund. India Coverage Evaluation Survey 2005.
Ministry of Health and Family Welfare, Government of India, United Nations Children's Fund. India Coverage Evaluation Survey 2007.
Ministry of Health and Family Welfare, Government of India, United Nations Children's Fund. India Coverage Evaluation Survey Data 2009-2010. [Data shared for this analysis]
Ministry of Health and Family Welfare (India), Public Health Foundation of India. India Pentavalent Vaccination Coverage by State 2013-2015.
Ministry of Health and Family Welfare, Government of India, World Health Organization - Regional Office for South-East Asia and Country Office for India, Centres for Disease Control and Prevention. India-Arunachal Pradesh Global Youth Tobacco Survey 2000. New Delhi, India: Ministry of Health and Family Welfare.
Ministry of Health and Family Welfare, Government of India, World Health Organization - Regional Office for South-East Asia and Country Office for India, Centres for Disease Control and Prevention. India-Assam Global Youth Tobacco Survey 2000. New Delhi, India: Ministry of Health and Family Welfare.
Ministry of Health and Family Welfare, Government of India, World Health Organization - Regional Office for South-East Asia and Country Office for India, Centres for Disease Control and Prevention. India-Bihar Global Youth Tobacco Survey 2000. New Delhi, India: Ministry of Health and Family Welfare.
Ministry of Health and Family Welfare, Government of India, World Health Organization - Regional Office for South-East Asia and Country Office for India, Centres for Disease Control and Prevention. India-Goa Global Youth Tobacco Survey 2000. NNew Delhi, India: Ministry of Health and Family Welfare.
Ministry of Health and Family Welfare, Government of India, World Health Organization - Regional Office for South-East Asia and Country Office for India, Centres for Disease Control and Prevention. India-Maharashtra Global Youth Tobacco Survey 2000. New Delhi, India: Ministry of Health and Family Welfare.
Ministry of Health and Family Welfare, Government of India, World Health Organization - Regional Office for South-East Asia and Country Office for India, Centres for Disease Control and Prevention. India-Manipur Global Youth Tobacco Survey 2000. New Delhi, India: Ministry of Health and Family Welfare.

Ministry of Health and Family Welfare, Government of India, World Health Organization - Regional Office for South-East Asia and Country Office for India, Centres for Disease Control and Prevention. India-Meghalaya Global Youth Tobacco Survey 2000. New Delhi, India: Ministry of Health and Family Welfare.
Ministry of Health and Family Welfare, Government of India, World Health Organization - Regional Office for South-East Asia and Country Office for India, Centres for Disease Control and Prevention. India-Mizoram Global Youth Tobacco Survey 2000. New Delhi, India: Ministry of Health and Family Welfare.
Ministry of Health and Family Welfare, Government of India, World Health Organization - Regional Office for South-East Asia and Country Office for India, Centres for Disease Control and Prevention. India-Nagaland Global Youth Tobacco Survey 2000. New Delhi, India: Ministry of Health and Family Welfare.
Ministry of Health and Family Welfare, Government of India, World Health Organization - Regional Office for South-East Asia and Country Office for India, Centres for Disease Control and Prevention. India-Sikkim Global Youth Tobacco Survey 2000. New Delhi, India: Ministry of Health and Family Welfare.
Ministry of Health and Family Welfare, Government of India, World Health Organization - Regional Office for South-East Asia and Country Office for India, Centres for Disease Control and Prevention. India-Tamil Nadu Global Youth Tobacco Survey 2000. New Delhi, India: Ministry of Health and Family Welfare.
Ministry of Health and Family Welfare, Government of India, World Health Organization - Regional Office for South-East Asia and Country Office for India, Centres for Disease Control and Prevention. India-Tripura Global Youth Tobacco Survey 2000. New Delhi, India: Ministry of Health and Family Welfare.
Ministry of Health and Family Welfare, Government of India, World Health Organization - Regional Office for South-East Asia and Country Office for India, Centres for Disease Control and Prevention. India-West Bengal Global Youth Tobacco Survey 2000. New Delhi, India: Ministry of Health and Family Welfare.
Ministry of Health and Family Welfare, Government of India, World Health Organization - Regional Office for South-East Asia and Country Office for India, Centres for Disease Control and Prevention. India-Andra Pradesh Global Youth Tobacco Survey 2001. New Delhi, India: Ministry of Health and Family Welfare.
Ministry of Health and Family Welfare, Government of India, World Health Organization - Regional Office for South-East Asia and Country Office for India, Centres for Disease Control and Prevention. India-Delhi Global Youth Tobacco Survey 2001. New Delhi, India: Ministry of Health and Family Welfare.
Ministry of Health and Family Welfare, Government of India, World Health Organization - Regional Office for South-East Asia and Country Office for India, Centres for Disease Control and Prevention. India-Uttar Pradesh Global Youth Tobacco Survey 2002. New Delhi, India: Ministry of Health and Family Welfare.
Ministry of Health and Family Welfare, Government of India, World Health Organization - Regional Office for South-East Asia and Country Office for India, Centres for Disease Control and Prevention. India-Uttaranchal Global Youth Tobacco Survey 2002. New Delhi, India: Ministry of Health and Family Welfare.
Ministry of Health and Family Welfare, Government of India, World Health Organization - Regional Office for South-East Asia and Country Office for India, Centres for Disease Control and Prevention. India-Karnataka Global Youth Tobacco Survey 2003. New Delhi, India: Ministry of Health and Family Welfare.
Centers for Disease Control and Prevention (CDC) and World Health Organization (WHO). India-Rajastan Global Youth Tobacco Survey 2003. United States: Centers for Disease Control and Prevention (CDC), 2003.
Ministry of Health and Family Welfare, Government of India, World Health Organization - Regional Office for South-East Asia and Country Office for India, Centres for Disease Control and Prevention. India-Gujarat Global Youth Tobacco Survey 2004. New Delhi, India: Ministry of Health and Family Welfare.
Ministry of Health and Family Welfare, Government of India, World Health Organization - Regional Office for South-East Asia and Country Office for India, Centres for Disease Control and Prevention. India-Gujarat with Ahmedabad Global Youth Tobacco Survey 2004. New Delhi, India: Ministry of Health and Family Welfare.
Ministry of Health and Family Welfare, Government of India, World Health Organization - Regional Office for South-East Asia and Country Office for India, Centres for Disease Control and Prevention. India-Haryana Global Youth Tobacco Survey 2004. New Delhi, India: Ministry of Health and Family Welfare.
Ministry of Health and Family Welfare, Government of India, World Health Organization - Regional Office for South-East Asia and Country Office for India, Centres for Disease Control and Prevention. India-Himachal Pradesh Global Youth Tobacco Survey 2004. New Delhi, India: Ministry of Health and Family Welfare.
Ministry of Health and Family Welfare, Government of India, World Health Organization - Regional Office for South-East Asia and Country Office for India, Centres for Disease Control and Prevention. India-Jammu and Kashmir Global Youth Tobacco Survey 2004. New Delhi, India: Ministry of Health and Family Welfare.
Ministry of Health and Family Welfare, Government of India, World Health Organization - Regional Office for South-East Asia and Country Office for India, Centres for Disease Control and Prevention. India-Kerala Global Youth Tobacco Survey 2004. New Delhi, India: Ministry of Health and Family Welfare.
Ministry of Health and Family Welfare, Government of India, World Health Organization - Regional Office for South-East Asia and Country Office for India, Centres for Disease Control and Prevention. India-Madhya Pradesh Global Youth Tobacco Survey 2004. New Delhi, India: Ministry of Health and Family Welfare.
Ministry of Health and Family Welfare, Government of India, World Health Organization - Regional Office for South-East Asia and Country Office for India, Centres for Disease Control and Prevention. India-Punjab Global Youth Tobacco Survey 2004. New Delhi, India: Ministry of Health and Family Welfare.
Ministry of Health and Family Welfare, Government of India, World Health Organization - Regional Office for South-East Asia and Country Office for India, Centres for Disease Control and Prevention. India Global Youth Tobacco Survey 2006. New Delhi, India: Ministry of Health and Family Welfare.
Ministry of Health and Family Welfare, Government of India, World Health Organization - Regional Office for South-East Asia and Country Office for India, Centres for Disease Control and Prevention. India Global Youth Tobacco Survey 2009. New Delhi, India: Ministry of Health and Family Welfare.
Ministry of Health and Family Welfare (India), United Nations Children's Fund (UNICEF). India Evaluation of Routine Immunization Coverage Survey 1997-1998.
Ministry of Health and Family Welfare (India), United Nations Children's Fund (UNICEF). India Evaluation of Routine Immunization Coverage Survey 1998-1999.
Ministry of Statistics and Programme Implementation, Government of India. Consumer Expenditure Survey, National Sample Survey Round 38, January-December 1983. New Delhi, India: Ministry of Statistics and Programme Implementation.

Ministry of Statistics and Programme Implementation, Government of India. Employment and Unemployment Survey, National Sample Survey Round 38, January-December 1983. New Delhi, India: Ministry of Statistics and Programme Implementation.
Ministry of Statistics and Programme Implementation, Government of India. Consumer Expenditure Survey, National Sample Survey Round 43, July 1987-June 1988. New Delhi, India: Ministry of Statistics and Programme Implementation.
Ministry of Statistics and Programme Implementation, Government of India. Employment and Unemployment Survey, National Sample Survey Round 43, July 1987- June 1988. New Delhi, India: Ministry of Statistics and Programme Implementation.
Ministry of Statistics and Programme Implementation, Government of India. Consumer Expenditure Survey, National Sample Survey Round 47, July - December 1991. New Delhi, India: Ministry of Statistics and Programme Implementation.
Ministry of Statistics and Programme Implementation, Government of India. Consumer Expenditure Survey, National Sample Survey Round 49, January-June 1993. New Delhi, India: Ministry of Statistics and Programme Implementation.
Ministry of Statistics and Programme Implementation, Government of India. Consumer Expenditure Survey, National Sample Survey Round 50, July 1993-June 1994. New Delhi, India: Ministry of Statistics and Programme Implementation.
Ministry of Statistics and Programme Implementation, Government of India. Consumer Expenditure Survey, National Sample Survey Round 50, July 1994-June 1995. New Delhi, India: Ministry of Statistics and Programme Implementation.
Ministry of Statistics and Programme Implementation, Government of India. Employment and Unemployment Survey, National Sample Survey Round 50, July 1993- June 1994. New Delhi, India: Ministry of Statistics and Programme Implementation.
Ministry of Statistics and Programme Implementation, Government of India. Employment and Unemployment Survey, National Sample Survey Round 50, July 1994-June 1995. New Delhi, India: Ministry of Statistics and Programme Implementation.
Ministry of Statistics and Programme Implementation, Government of India. Consumer Expenditure Survey, National Sample Survey Round 52, July 1995-June 1996. New Delhi, India: Ministry of Statistics and Programme Implementation.
Ministry of Statistics and Programme Implementation, Government of India. Common Property Resources, Sanitation & Hygiene Services, National Sample Survey Round 54, January - June 1998. New Delhi, India: Ministry of Statistics and Programme Implementation.
Ministry of Statistics and Programme Implementation, Government of India. Consumer Expenditure Survey, National Sample Survey Round 54, January - June 1998. New Delhi, India: Ministry of Statistics and Programme Implementation.
Ministry of Statistics and Programme Implementation, Government of India. Consumer Expenditure Survey, India National Sample Survey Round 55, July 1999-June 2000. New Delhi, India: Ministry of Statistics and Programme Implementation.
Ministry of Statistics and Programme Implementation, Government of India. Employment and Unemployment Survey, India National Sample Survey Round 55, July 1999-June 2000. New Delhi, India: Ministry of Statistics and Programme Implementation.
Ministry of Statistics and Programme Implementation, Government of India. Consumer Expenditure Survey, National Sample Survey Round 60, January-June 2004. New Delhi, India: Ministry of Statistics and Programme Implementation.
Ministry of Statistics and Programme Implementation, Government of India. Employment and Unemployment surveys, National Sample Survey Round 60, January-June 2004. New Delhi, India: Ministry of Statistics and Programme Implementation.
Ministry of Statistics and Programme Implementation, Government of India. Consumer Expenditure Survey, National Sample Survey Round 61, July 2004 - June 2005. New Delhi, India: Ministry of Statistics and Programme Implementation.
Ministry of Statistics and Programme Implementation, Government of India. Employment and Unemployment Survey, National Sample Survey Round 61, July 2004- June 2005. New Delhi, India: Ministry of Statistics and Programme Implementation.
Ministry of Statistics and Programme Implementation, Government of India. Consumer Expenditure Survey, National Sample Survey Round 63, July 2006- June 2007. New Delhi, India: Ministry of Statistics and Programme Implementation
Ministry of Statistics and Programme Implementation, Government of India. Consumer Expenditure Survey, National Sample Survey Round 64, July 2007- June 2008. New Delhi, India: Ministry of Statistics and Programme Implementation.
Ministry of Statistics and Programme Implementation, Government of India. Employment and Unemployment Survey, National Sample Survey Round 64, July 2007- June 2008. New Delhi, India: Ministry of Statistics and Programme Implementation.
Ministry of Statistics and Programme Implementation, Government of India. Consumer Expenditure Survey, National Sample Survey Round 66, July 2009- June 2010. New Delhi, India: Ministry of Statistics and Programme Implementation
Ministry of Statistics and Programme Implementation, Government of India. Consumer Expenditure Survey, National Sample Survey Round 68, July 2011- June 2012. New Delhi, India: Ministry of Statistics and Programme Implementation
Ministry of Statistics and Programme Implementation, Government of India. Household expenditure on services and durable goods survey, National Sample Survey Round 72, July 2014 - June 2015. New Delhi, India: Ministry of Statistics and Programme Implementation.
Ministry of Statistics and Programme Implementation, Government of India. India State Series: Gross State Domestic Product at Factor Cost by Industry of Origin Tables 1980-81 to 1993-94. New Delhi, India: Ministry of Statistics and Programme Implementation.
Ministry of Statistics and Programme Implementation, Government of India. India State Series: Gross State Domestic Product at Factor Cost by Industry of Origin Tables 1993-94 to 2004-05. New Delhi, India: Ministry of Statistics and Programme Implementation.
Ministry of Statistics and Programme Implementation, Government of India. India State Series: Gross State Domestic Product at Factor Cost by Industry of Origin Tables 1999-2000 to 2009-10. New Delhi, India: Ministry of Statistics and Programme Implementation.
Ministry of Statistics and Programme Implementation, Government of India. India State Series: Gross State Domestic Product at Factor Cost by Industry of Origin Tables 2004-05 to 2013-14. New Delhi, India: Ministry of Statistics and Programme Implementation.
Ministry of Statistics and Programme Implementation, Government of India. Participation & Expenditure in Education Survey, National Sample Survey Round 64, July 2007- June 2008. New Delhi, India: Ministry of Statistics and Programme Implementation.
Ministry of Statistics and Programme Implementation, Government of India. Participation in Education Survey, National Sample Survey Round 52, July 1995-June 1996. New Delhi, India: Ministry of Statistics and Programme Implementation.

Ministry of Statistics and Programme Implementation, Government of India. Rapid Survey on Swachhta Status, National Sample Survey Round 72, July 2014-June 2015. New Delhi, India: Ministry of Statistics and Programme Implementation.
Ministry of Statistics and Programme Implementation, Government of India. Rapid Survey on Swachhta Status, National Sample Survey Round 75, July 2017-June 2018. New Delhi, India: Ministry of Statistics and Programme Implementation.
Ministry of Statistics and Programme Implementation, Government of India. Survey on Healthcare, National Sample Survey Round 52, July 1995-June 1996. New Delhi, India: Ministry of Statistics and Programme Implementation.
Ministry of Statistics and Programme Implementation, Government of India. Survey on Literacy and Culture, National Sample Survey Round 47, July - December 1991. New Delhi, India: Ministry of Statistics and Programme Implementation.
Ministry of Statistics and Programme Implementation, Government of India. Survey on Morbidity and Healthcare, National Sample Survey Round 60, January-June 2004. New Delhi, India: Ministry of Statistics and Programme Implementation.
Ministry of Statistics and Programme Implementation, Government of India. Survey on Participation in Education, National Sample Survey Round 42, July 1986- June 1987. New Delhi, India: Ministry of Statistics and Programme Implementation.
Ministry of Women and Child Development, Government of India, United Nations Children's Fund. India Rapid Survey on Children 2013-2014. New Delhi, India: Ministry of Women and Child Development.
Minz S, Balraj V, Lalitha MK, Murali N, Cherian T, Manoharan G, Kadirvan S, Joseph A, Steinhoff MC. Incidence of Haemophilus influenzae type b meningitis in India. Indian J Med Res. 2008; 128: 57-64.
Mir M, Newcombe R. The relationship of dietary salt and blood pressure in three farming communities in Kashmir. J Hum Hypertens. 1988; 2: 241-6.
Mir MA, Mir F, Khosla T, Newcombe R. The relationship of salt intake and arterial blood pressure in salted-tea drinking Kashmiris. Int J Cardiol. 1986; 13: 279-88.
Mohan M, Sperduto RD, Angra SK, Milton RC, Mathur RL, Underwood BA, Jaffery N, Pandya CB, Chhabra VK, Vajpayee RB. India-US case-control study of age-related cataracts. India-US Case-Control Study Group. Arch Ophthalmol. 1989; 107(5): 670-6.
Mohan V, Deepa M, Anjana RM, Lanthorn H, Deepa R. Incidence of diabetes and pre-diabetes in a selected urban south Indian population (CUPS - 19). J Assoc Physicians India. 2008; 56: 152-7.
Mohan V, Deepa M, Farooq S, Datta M, Deepa R. Prevalence, awareness and control of hypertension in Chennai--The Chennai Urban Rural Epidemiology Study (CURES-52). J Assoc Physicians India. 2007; 55: 326-32.
Mohan V, Mathur P, Deepa R, Deepa M, Shukla DK, Menon GR, Anand K, Desai NG, Joshi PP, Mahanta J, Thankappan KR, Shah B. Urban rural differences in prevalence of self-reported diabetes in India--the WHO-ICMR Indian NCD risk factor surveillance. Diabetes Res Clin Pract. 2008; 80: 159-68.
Mondal NK, Roy A, Mukherjee B, Das D, Ray MR. Indoor Air Pollution from Biomass Burning Activates Akt in Airway Cells and Peripheral Blood Lymphocytes: A Study among Premenopausal Women in Rural India. Toxicol Pathol. 2010; 38: 1085-98.
Mony PK, Varghese B, Thomas T. Estimation of perinatal mortality rate for institutional births in Rajasthan state, India, using capture-recapture technique. BMJ Open. 2015; 5: e005966.
Mukhopadhyay R, Sambandam S, Pillarisetti A, Jack D, Mukhopadhyay K, Balakrishnan K, Vaswani M, Bates MN, Kinney PL, Arora N, Smith KR. Cooking practices, air quality, and the acceptability of advanced cookstoves in Haryana, India: an exploratory study to inform large-scale interventions. Glob Health Action. 2012; 5: 1-13.
Murthy J, Vijay S, Ravi Raju C, Thomas J. Acute symptomatic seizures associated with neurocysticercoses: A community based prevalence study and comprehensive rural epilepsy study in South India (CRESSI). Neurology Asia. 2004; 9: 86.
Nadkarni A, Weiss HA, Naik A, Bhat B, Patel V. The six-year outcome of alcohol use disorders in men: A population based study from India. Drug Alcohol Depend. 2016; 162: 107–15.
Nagaraja D, Gururaj G, Girish N, Panda S, Roy AK, Sarma GRK, Srinivasa R. Feasibility study of stroke surveillance: data from Bangalore, India. Indian J Med Res. 2009; 130: 396–403.
Nair MKC, Russell PSS, Mammen P, Abhiram Chandran R, Krishnan R, Nazeema S, Chembagam N, Peter D. ADad 3: the epidemiology of Anxiety Disorders among adolescents in a rural community population in India. Indian J Pediatr. 2013; 80: S144-148.
Nangia V.The central India eye and medical study data set 2006-2008. [Data shared for this analysis]
Nangia V, Jonas JB, Sinha A, Matin A, Kulkarni M, Panda-Jonas S. Ocular axial length and its associations in an adult population of Central Rural India. The Central India Eye and Medical Study [Unpublished data]. Ophthalmology 2010;117(7):1360-6.
National Cancer Registry Programme (India). India - North East Population Based Cancer Registries Report 2005-2006. New Delhi, India: Indian Council of Medical Research (ICMR), 2008.
National Cancer Registry Programme (India). India Consolidated Report of Population Based Cancer Registries 2004-2005. New Delhi, India: Indian Council of Medical Research (ICMR), 2008.
National Cancer Registry Programme (India). India Consolidated Report of Population Based Cancer Registries 2012-2014. New Delhi, India: Indian Council of Medical Research (ICMR), 2016.
National Cancer Registry Programme (India). India Population Based Cancer Registries 2009-2011. New Delhi, India: Indian Council of Medical Research (ICMR), 2013.
National Cancer Registry Programme (India). India Three-Year Report of Population Based Cancer Registries 2006-2008. New Delhi, India: Indian Council of Medical Research (ICMR), 2010.
National Institute of Medical Statistics, Indian Council of Medical Research (ICMR), Integrated Disease Surveillance Programme. Non-Communicable Disease Risk Factors Survey Data 2007-2008. New Delhi, India: ICMR. [Data shared for this analysis]
National Institute of Medical Statistics, Indian Council of Medical Research (ICMR), Integrated Disease Surveillance Programme. Non-Communicable Disease Risk Factors Survey Report 2007-2008. New Delhi, India: ICMR.
National Institute of Mental Health and Neurosciences, World Health Organization (WHO). Report on WHO Collaborative Project on Unrecorded Consumption of Alcohol in Karnataka, India 2001-2002. Available from: http://nimhans.ac.in/cam/sites/default/files/Publications/25.pdf

National Nutrition Monitoring Bureau, National Institute of Nutrition (NIN), Indian Council of Medical Research. India Rural First Repeat Survey of Diet and Nutritional Status Data 1988-1990. [Data shared for this analysis]

National Nutrition Monitoring Bureau, National Institute of Nutrition (NIN), Indian Council of Medical Research. India Rural First Repeat Survey of Diet and Nutritional Status Report 1988-1990. Hyderabad, India: NIN.

National Nutrition Monitoring Bureau, National Institute of Nutrition (NIN), Indian Council of Medical Research. India Rural Second Repeat Survey of Diet and Nutritional Status Data 1996-1997. [Data shared for this analysis]

National Nutrition Monitoring Bureau, National Institute of Nutrition (NIN), Indian Council of Medical Research. India Rural Second Repeat Survey of Diet and Nutritional Status Report 1996-1997. Hyderabad, India: NIN.

National Nutrition Monitoring Bureau, National Institute of Nutrition (NIN), Indian Council of Medical Research. India Rural Survey of Diet and Nutritional Status Data 1994-1995. [Data shared for this analysis]

National Nutrition Monitoring Bureau, National Institute of Nutrition (NIN), Indian Council of Medical Research. India Rural Survey of Diet and Nutritional Status Report 1994-1995. Hyderabad, India: NIN.

National Nutrition Monitoring Bureau, National Institute of Nutrition (NIN), Indian Council of Medical Research. India Rural Survey of Diet and Nutritional Status Data 2000-2001. [Data shared for this analysis]

National Nutrition Monitoring Bureau, National Institute of Nutrition (NIN), Indian Council of Medical Research. India Rural Survey of Diet and Nutritional Status Report 2000-2001. Hyderabad, India: NIN.

National Nutrition Monitoring Bureau, National Institute of Nutrition (NIN), Indian Council of Medical Research. India Rural Survey of Diet and Nutritional Status Data 2004-2006. [Data shared for this analysis]

National Nutrition Monitoring Bureau, National Institute of Nutrition (NIN), Indian Council of Medical Research. India Rural Survey of Diet and Nutritional Status Report 2004-2006. Hyderabad, India: NIN.

National Nutrition Monitoring Bureau, National Institute of Nutrition (NIN), Indian Council of Medical Research. India Rural Third Repeat Survey of Diet and Nutritional Status Data 2011-2012. [Data shared for this analysis]

National Nutrition Monitoring Bureau, National Institute of Nutrition (NIN), Indian Council of Medical Research. India Rural Third Repeat Survey of Diet and Nutritional Status Report 2011-2012. Hyderabad, India: NIN.

National Nutrition Monitoring Bureau, National Institute of Nutrition (NIN), Indian Council of Medical Research. India Survey of Diet and Nutritional Status Data 1990-1992. [Data shared for this analysis]

National Nutrition Monitoring Bureau, National Institute of Nutrition (NIN), Indian Council of Medical Research. India Survey of Diet and Nutritional Status Report 1990-1992. Hyderabad, India: NIN.

National Nutrition Monitoring Bureau, National Institute of Nutrition (NIN), Indian Council of Medical Research. India Tribal First Repeat Survey of Diet and Nutritional Status Data 1998-1999. [Data shared for this analysis]

National Nutrition Monitoring Bureau, National Institute of Nutrition (NIN), Indian Council of Medical Research. India Tribal First Repeat Survey of Diet and Nutritional Status Report 1998-1999. Hyderabad, India: NIN.

National Nutrition Monitoring Bureau, National Institute of Nutrition (NIN), Indian Council of Medical Research. India Tribal Second Repeat Survey of Diet and Nutritional Status Data 2007-2008. [Data shared for this analysis]

National Nutrition Monitoring Bureau, National Institute of Nutrition (NIN), Indian Council of Medical Research. India Tribal Second Repeat Survey of Diet and Nutritional Status Report 2007-2008. Hyderabad, India: NIN.

National Nutrition Monitoring Bureau, National Institute of Nutrition (NIN), Indian Council of Medical Research. India Urban Slums Survey of Diet and Nutritional Status Data 1993-1994. [Data shared for this analysis]

National Nutrition Monitoring Bureau, National Institute of Nutrition (NIN), Indian Council of Medical Research. India Urban Slums Survey of Diet and Nutritional Status Report 1993-1994. Hyderabad, India: NIN.

National Nutrition Monitoring Bureau, National Institute of Nutrition (NIN), Indian Council of Medical Research. India Urban Slums Survey of Diet and Nutritional Status Data 2015-2016. [Data shared for this analysis]

National Nutrition Monitoring Bureau, National Institute of Nutrition (NIN), Indian Council of Medical Research. India Urban Slums Survey of Diet and Nutritional Status Report 2015-2016. Hyderabad, India: NIN.

National Nutrition Monitoring Bureau, National Institute of Nutrition, Indian Council of Medical Research. India National Nutrition Monitoring Bureau Eight States Pooled Data 1991-1992. [Data shared for this analysis]

Naumoff KS. Quantitative Metrics of Exposure and Health for Indoor Air Pollution from Household Biomass Fuels in Guatemala and India [dissertation]. Berkeley, United States: University of California, Berkeley, 2007.

Nazareth Hospital. Inpatient and Discharge Data 2014. Shillong, Meghalaya: Nazareth Hospital. [Data shared for this analysis]

Nazir A, Papita R, Anbalagan VP, Anjana RM, Deepa M, Mohan V. Prevalence of diabetes in Asian Indians based on glycated hemoglobin and fasting and 2-H post-load (75-g) plasma glucose (CURES-120). Diabetes Technol Ther. 2012; 14: 665–8.

Nirmalan PK, Tielsch JM, Katz J, Thulasiraj RD, Krishnadas R, Ramakrishnan R, Robin AL. Relationship between vision impairment and eye disease to vision-specific quality of life and function in rural India: the Aravind Comprehensive Eye Survey. Invest Ophthalmol Vis Sci. 2005; 46(7): 2308-12.

Nizamie SH, Akthar S, Banerjee I, Goyal N. Health care delivery model in epilepsy to reduce treatment gap: World Health Organization study from a rural tribal population of India. Epilepsy Res. 2009; 84: 146–52.

Norboo T, Stobdan T, Tsering N, Angchuk N, Tsering P, Ahmed I, Chorol T, Kumar Sharma V, Reddy P, Singh SB, Kimura Y, Sakamoto R, Fukutomi E, Ishikawa M, Suwa K, Kosaka Y, Nose M, Yamaguchi T, Tsukihara T, Matsubayashi K, Otsuka K, Okumiya K. Prevalence of hypertension at high altitude: cross-sectional survey in Ladakh, Northern India 2007-2011. BMJ Open. 2015; 5: e007026.

Nuffield Department of Population Health, University of Oxford. India - Trivandrum Tobacco Study.

Nuffield Department of Population Health, University of Oxford. India Studies of Smoking and Death.

Nuffield Department of Population Health, University of Oxford. Prospective Study of 500,000 Adults in Chennai, India .
O'Gorman C, Broadley SA. Smoking and multiple sclerosis: evidence for latitudinal and temporal variation. J Neurol. 2014 ;261: 1677–83.
Office of the Chief Registrar of Births and Deaths, Directorate of Economics and Statistics Bangalore Government of Karnataka. India - Karnataka Medical Certification of Cause of Death Report 2014. Bangalore, India.
Office of the Chief Registrar of Births and Deaths, Directorate of Economics and Statistics Bangalore Government of Karnataka. India - Karnataka Medical Certification of Cause of Death Report 2015. Bangalore, India.
Office of the Registrar General & Census Commissioner, Centre for Global Health Research - University of Toronto. India SRS Special Fertility and Mortality Survey 1998. New Delhi, India: Office of the Registrar General & Census Commissioner; 2005.
Office of the Registrar General and Census Commissioner, Ministry of Health and Family Welfare, Government of India, National Institute of Health and Family Welfare, Nutrition Foundation of India, National Institute of Nutrition, Indian Council of Medical Research. India Clinical, Anthropometric and Bio-chemical (CAB) Survey Data 2014 [Biomarker Component of Annual Health Survey]. New Delhi, India: Office of the Registrar General and Census Commissioner.
Office of the Registrar General & Census Commissioner, Ministry of Home Affairs, Government of India. India Annual Health Survey Data 2010-2011. New Delhi, India: Office of the Registrar General & Census Commissioner.
Office of the Registrar General & Census Commissioner, Ministry of Home Affairs, Government of India. India Annual Health Survey Data 2010-2013. New Delhi, India: Office of the Registrar General & Census Commissioner.
Office of the Registrar General & Census Commissioner, Ministry of Home Affairs, Government of India. India Annual Health Survey Data 2011-2012. New Delhi, India: Office of the Registrar General & Census Commissioner.
Office of the Registrar General & Census Commissioner, Ministry of Home Affairs, Government of India. India Annual Health Survey Data 2012-2013. New Delhi, India: Office of the Registrar General & Census Commissioner.
Office of the Registrar General & Census Commissioner, Ministry of Home Affairs, Government of India. India Annual Health Survey Report 2010-2011. New Delhi, India: Office of the Registrar General & Census Commissioner.
Office of the Registrar General & Census Commissioner, Ministry of Home Affairs, Government of India. India Annual Health Survey Report 2010-2013. New Delhi, India: Office of the Registrar General & Census Commissioner.
Office of the Registrar General & Census Commissioner, Ministry of Home Affairs, Government of India. India Annual Health Survey Report 2011-2012. New Delhi, India: Office of the Registrar General & Census Commissioner.
Office of the Registrar General & Census Commissioner, Ministry of Home Affairs, Government of India. India Annual Health Survey Report 2012-2013. New Delhi, India: Office of the Registrar General & Census Commissioner.
Office of the Registrar General & Census Commissioner, Ministry of Home Affairs, Government of India. India Medical Certification of Cause of Death Report 1997. New Delhi, India: Office of the Registrar General & Census Commissioner.
Office of the Registrar General & Census Commissioner, Ministry of Home Affairs, Government of India. India Medical Certification of Cause of Death Report 2005. New Delhi, India: Office of the Registrar General & Census Commissioner.
Office of the Registrar General & Census Commissioner, Ministry of Home Affairs, Government of India. India Medical Certification of Cause of Death Report 2006. New Delhi, India: Office of the Registrar General & Census Commissioner.
Office of the Registrar General & Census Commissioner, Ministry of Home Affairs, Government of India. India Medical Certification of Cause of Death Report 2008. New Delhi, India: Office of the Registrar General & Census Commissioner.
Office of the Registrar General & Census Commissioner, Ministry of Home Affairs, Government of India. India Medical Certification of Cause of Death Report 2009. New Delhi, India: Office of the Registrar General & Census Commissioner; 2014.
Office of the Registrar General & Census Commissioner, Ministry of Home Affairs, Government of India. India Medical Certification of Cause of Death Report 2010. New Delhi, India: Office of the Registrar General & Census Commissioner; 2014.
Office of the Registrar General & Census Commissioner, Ministry of Home Affairs, Government of India. India Medical Certification of Cause of Death Report 2011. New Delhi, India: Office of the Registrar General & Census Commissioner.
Office of the Registrar General & Census Commissioner, Ministry of Home Affairs, Government of India. India Medical Certification of Cause of Death Report 2012. New Delhi, India: Office of the Registrar General & Census Commissioner; 2015.
Office of the Registrar General & Census Commissioner, Ministry of Home Affairs, Government of India. India Medical Certification of Cause of Death Report 2013. New Delhi, India: Office of the Registrar General & Census Commissioner.
Office of the Registrar General & Census Commissioner, Ministry of Home Affairs, Government of India. India Medical Certification of Cause of Death State-Level Tabulations 1990. New Delhi, India: Office of the Registrar General & Census Commissioner.
Office of the Registrar General & Census Commissioner, Ministry of Home Affairs, Government of India. India Medical Certification of Cause of Death State-Level Tabulations 1991. New Delhi, India: Office of the Registrar General & Census Commissioner.
Office of the Registrar General & Census Commissioner, Ministry of Home Affairs, Government of India. India Medical Certification of Cause of Death State-Level Tabulations 1992. New Delhi, India: Office of the Registrar General & Census Commissioner.
Office of the Registrar General & Census Commissioner, Ministry of Home Affairs, Government of India. India Medical Certification of Cause of Death State-Level Tabulations 1993. New Delhi, India: Office of the Registrar General & Census Commissioner.
Office of the Registrar General & Census Commissioner, Ministry of Home Affairs, Government of India. India Medical Certification of Cause of Death State-Level Tabulations 1994. New Delhi, India: Office of the Registrar General & Census Commissioner.
Office of the Registrar General & Census Commissioner, Ministry of Home Affairs, Government of India. India Medical Certification of Cause of Death State-Level Tabulations 1995. New Delhi, India: Office of the Registrar General & Census Commissioner.
Office of the Registrar General & Census Commissioner, Ministry of Home Affairs, Government of India. India Medical Certification of Cause of Death State-Level Tabulations 1996. New Delhi, India: Office of the Registrar General & Census Commissioner.
Office of the Registrar General & Census Commissioner, Ministry of Home Affairs, Government of India. India Medical Certification of Cause of Death State-Level Tabulations 1998. New Delhi, India: Office of the Registrar General & Census Commissioner.

Office of the Registrar General & Census Commissioner, Ministry of Home Affairs, Government of India. India Medical Certification of Cause of Death State-Level Tabulations 1999. New Delhi, India: Office of the Registrar General & Census Commissioner.
Office of the Registrar General & Census Commissioner, Ministry of Home Affairs, Government of India. India Medical Certification of Cause of Death State-Level Tabulations 2000. New Delhi, India: Office of the Registrar General & Census Commissioner.
Office of the Registrar General & Census Commissioner, Ministry of Home Affairs, Government of India. India Medical Certification of Cause of Death State-Level Tabulations 2001. New Delhi, India: Office of the Registrar General & Census Commissioner.
Office of the Registrar General & Census Commissioner, Ministry of Home Affairs, Government of India. India Medical Certification of Cause of Death State-Level Tabulations 2002. New Delhi, India: Office of the Registrar General & Census Commissioner.
Office of the Registrar General & Census Commissioner, Ministry of Home Affairs, Government of India. India Medical Certification of Cause of Death State-Level Tabulations 2003. New Delhi, India: Office of the Registrar General & Census Commissioner.
Office of the Registrar General & Census Commissioner, Ministry of Home Affairs, Government of India. India Medical Certification of Cause of Death State-Level Tabulations 2004. New Delhi, India: Office of the Registrar General & Census Commissioner.
Office of the Registrar General & Census Commissioner, Ministry of Home Affairs, Government of India. India Population and Housing Census 1981. New Delhi, India: Office of the Registrar General & Census Commissioner.
Office of the Registrar General & Census Commissioner, Ministry of Home Affairs, Government of India. India Population and Housing Census 2001. New Delhi, India: Office of the Registrar General & Census Commissioner.
Office of the Registrar General & Census Commissioner, Ministry of Home Affairs, Government of India. India Population and Housing Census 2011. New Delhi, India: Office of the Registrar General & Census Commissioner; 2012.
Office of the Registrar General & Census Commissioner, Ministry of Home Affairs, Government of India. India Sample Registration System Baseline Survey 2014. New Delhi, India: Office of the Registrar General & Census Commissioner.
Office of the Registrar General & Census Commissioner, Ministry of Home Affairs, Government of India. India SRS Statistical Report 1999. New Delhi, India: Office of the Registrar General & Census Commissioner.
Office of the Registrar General & Census Commissioner, Ministry of Home Affairs, Government of India. India SRS Statistical Report 2000. New Delhi, India: Office of the Registrar General & Census Commissioner.
Office of the Registrar General & Census Commissioner, Ministry of Home Affairs, Government of India. India SRS Statistical Report 2001. New Delhi, India: Office of the Registrar General & Census Commissioner; 2005.
Office of the Registrar General & Census Commissioner, Ministry of Home Affairs, Government of India. India SRS Statistical Report 2002. New Delhi, India: Office of the Registrar General & Census Commissioner; 2005.
Office of the Registrar General & Census Commissioner, Ministry of Home Affairs, Government of India. India SRS Statistical Report 2003. New Delhi, India: Office of the Registrar General and Census Commissioner; 2005.
Office of the Registrar General & Census Commissioner, Ministry of Home Affairs, Government of India. India SRS Statistical Report 2004. New Delhi, India: Office of the Registrar General and Census Commissioner; 2006.
Office of the Registrar General & Census Commissioner, Ministry of Home Affairs, Government of India. India SRS Statistical Report 2005. New Delhi, India: Office of the Registrar General and Census Commissioner; 2006.
Office of the Registrar General & Census Commissioner, Ministry of Home Affairs, Government of India. India SRS Statistical Report 2006. New Delhi, India: Office of the Registrar General and Census Commissioner; 2008.
Office of the Registrar General & Census Commissioner, Ministry of Home Affairs, Government of India. India SRS Statistical Report 2007. New Delhi, India: Office of the Registrar General and Census Commissioner; 2008.
Office of the Registrar General & Census Commissioner, Ministry of Home Affairs, Government of India. India SRS Statistical Report 2008. New Delhi, India: Office of the Registrar General and Census Commissioner; 2010.
Office of the Registrar General & Census Commissioner, Ministry of Home Affairs, Government of India. India SRS Statistical Report 2009. New Delhi, India: Office of the Registrar General and Census Commissioner.
Office of the Registrar General & Census Commissioner, Ministry of Home Affairs, Government of India. India SRS Statistical Report 2010. New Delhi, India: Office of the Registrar General and Census Commissioner; 2012.
Office of the Registrar General & Census Commissioner, Ministry of Home Affairs, Government of India. India SRS Statistical Report 2011. New Delhi, India: Office of the Registrar General and Census Commissioner; 2013.
Office of the Registrar General & Census Commissioner, Ministry of Home Affairs, Government of India. India SRS Statistical Report 2012. New Delhi, India: Office of the Registrar General and Census Commissioner; 2013.
Office of the Registrar General & Census Commissioner, Ministry of Home Affairs, Government of India. India SRS Statistical Report 2013. New Delhi, India: Office of the Registrar General and Census Commissioner; 2014.
Office of the Registrar General & Census Commissioner, Ministry of Home Affairs, Government of India. India SRS Statistical Report 2014. New Delhi, India: Office of the Registrar General and Census Commissioner.
Office of the Registrar General & Census Commissioner, Ministry of Home Affairs, Government of India. India SRS Verbal Autopsy 2004-2006. New Delhi, India: Office of the Registrar General and Census Commissioner. [Data shared for this analysis]
Office of the Registrar General & Census Commissioner, Ministry of Home Affairs, Government of India. India SRS Verbal Autopsy 2007-2009. New Delhi, India: Office of the Registrar General and Census Commissioner. [Data shared for this analysis]
Office of the Registrar General & Census Commissioner, Ministry of Home Affairs, Government of India. India SRS Verbal Autopsy 2010-2013. New Delhi, India: Office of the Registrar General and Census Commissioner. [Data shared for this analysis]
Office of the Registrar General & Census Commissioner, Ministry of Home Affairs, Government of India. India SRS Verbal Autopsy Report 2004-2006. New Delhi, India: Office of the Registrar General and Census Commissioner.

Office of the Registrar General & Census Commissioner, Ministry of Home Affairs, Government of India. India SRS Verbal Autopsy Report 2007-2009. New Delhi, India: Office of the Registrar General and Census Commissioner.
Office of the Registrar General & Census Commissioner, Ministry of Home Affairs, Government of India. India SRS Verbal Autopsy Report 2010-2013. New Delhi, India: Office of the Registrar General and Census Commissioner.
Office of the Registrar General & Census Commissioner, Ministry of Home Affairs, Government of India. India Vital Statistics 1980. New Delhi, India: Office of the Registrar General and Census Commissioner.
Office of the Registrar General & Census Commissioner, Ministry of Home Affairs, Government of India. India Vital Statistics 1981. New Delhi, India: Office of the Registrar General and Census Commissioner.
Office of the Registrar General & Census Commissioner, Ministry of Home Affairs, Government of India. India Vital Statistics 1982. New Delhi, India: Office of the Registrar General and Census Commissioner.
Office of the Registrar General & Census Commissioner, Ministry of Home Affairs, Government of India. India Vital Statistics 1983. New Delhi, India: Office of the Registrar General and Census Commissioner.
Office of the Registrar General & Census Commissioner, Ministry of Home Affairs, Government of India. India Vital Statistics 1984. New Delhi, India: Office of the Registrar General and Census Commissioner.
Office of the Registrar General & Census Commissioner, Ministry of Home Affairs, Government of India. India Vital Statistics 1986. New Delhi, India: Office of the Registrar General and Census Commissioner.
Office of the Registrar General & Census Commissioner, Ministry of Home Affairs, Government of India. India Vital Statistics 1987. New Delhi, India: Office of the Registrar General and Census Commissioner.
Office of the Registrar General & Census Commissioner, Ministry of Home Affairs, Government of India. India Vital Statistics 1989. New Delhi, India: Office of the Registrar General and Census Commissioner.
Office of the Registrar General and Census Commissioner (India). India Survey of Causes of Death (Rural) 1980.
Office of the Registrar General and Census Commissioner (India). India Survey of Causes of Death (Rural) 1981.
Office of the Registrar General and Census Commissioner (India). India Survey of Causes of Death (Rural) 1982.
Office of the Registrar General and Census Commissioner (India). India Survey of Causes of Death (Rural) 1983.
Office of the Registrar General and Census Commissioner (India). India Survey of Causes of Death (Rural) 1984.
Office of the Registrar General and Census Commissioner (India). India Survey of Causes of Death (Rural) 1985.
Office of the Registrar General and Census Commissioner (India). India Survey of Causes of Death (Rural) 1986.
Office of the Registrar General and Census Commissioner, Ministry of Health and Family Welfare, Government of India, National Institute of Health and Family Welfare, Nutrition Foundation of India, National Institute of Nutrition, Indian Council of Medical Research. India Clinical, Anthropometric and Bio-chemical (CAB) Survey Data 2014 [Biomarker Component of Annual Health Survey]. New Delhi, India: Office of the Registrar General and Census Commissioner.
Office of the Registrar General and Census Commissioner, Ministry of Health and Family Welfare, Government of India, National Institute of Health and Family Welfare, Nutrition Foundation of India, National Institute of Nutrition, Indian Council of Medical Research. India Clinical, Anthropometric and Bio-chemical (CAB) Survey Report 2014 [Biomarker Component of Annual Health Survey]. New Delhi, India: Office of the Registrar General and Census Commissioner.
Oommen AM, Abraham VJ, George K, Jose VJ. Prevalence of coronary heart disease in rural and urban Vellore: A repeat cross-sectional survey. Indian Heart J. 2016; 68: 473-9.
Oommen AM, Abraham VJ, George K, Jose VJ. Prevalence of risk factors for non-communicable diseases in rural & urban Tamil Nadu. Indian J Med Res. 2016; 144: 460-71.
Pal DK, Das T, Sengupta S. Comparison of key informant and survey methods for ascertainment of childhood epilepsy in West Bengal, India. Int J Epidemiol. 1998; 27: 672–6.
Pandey RM, Gupta R, Misra A, Misra P, Singh V, Agrawal A, Dey S, Rao S, Menon VU, Kamalamma N, Devi KPV, Revathi K, Sharma V. Determinants of urban-rural differences in cardiovascular risk factors in middle-aged women in India: a cross-sectional study. Int J Cardiol. 2013; 163: 157-62.
Pandey S, Singhi P, Bharti B. Prevalence and treatment gap in childhood epilepsy in a north Indian city: a community-based study. J Trop Pediatr. 2014; 60: 118-23.
Pandian JD, Singh G, Kaur P, Bansal R, Paul BS, Singla M, Singh S, Samuel CJ, Verma SJ, Moodbidri P, Mehmi G, Sharma A, Arora OP, Dhanuka AK, Sobti MK, Sehgal H, Kaur M, Grewal SS, Jhawar SS, Shadangi TN, Arora T, Saxena A, Sachdeva G, Gill JS, Brar RS, Gill A, Bakshi SS, Pawar SS, Singh G, Sikka P, Litoria PK, Sharma M. Incidence, short-term outcome, and spatial distribution of stroke patients in Ludhiana, India. Neurology. 2016; 86: 425-33.
Pandian JD. Ludhiana Population Based Stroke Registry 2012-2013 [Data shared for this analysis]
Pandit L, Kundapur R. Prevalence and patterns of demyelinating central nervous system disorders in urban Mangalore, South India. Mult Scler. 2014; 20: 1651-3.
Parkin DM, International Agency for Research on Cancer, International Association of Cancer Registries. Cancer Incidence in Five Continents. Vol. I to VIII. Lyon, France, IARCPress, 2005.
Patandin S, Bots ML, Abel R, Valkenburg HA. Impaired glucose tolerance and diabetes mellitus in a rural population in south India. Diabetes Res Clin Pract. 1994; 24: 47-53.
Patil YP, Shinde RL. Undernutrition among Indian men: a study based on NFHS-3. Am J Men Health. 2014; 8: 492–502.
Pemminati S, Prabha Adhikari MR, Pathak R, Pai MRSM. Prevalence of metabolic syndrome (METS) using IDF 2005 guidelines in a semi urban south Indian (Boloor Diabetes Study) population of Mangalore. J Assoc Physicians India. 2010; 58: 674–7.
Pillai A, Nayak MB, Greenfield TK, Bond JC, Hasin DS, Patel V. Adolescent drinking onset and its adult consequences among men: A population based study from India. J Epidemiol Community Health. 2014; 68: 922–7.

Pillai A, Nayak MB, Greenfield TK, Bond JC, Nadkarni A, Patel V. Patterns of alcohol use, their correlates, and impact in male drinkers: a population-based survey from Goa, India. Soc Psychiatry Psychiatr Epidemiol. 2013; 48: 275–82.

Postgraduate Institute of Medical Education & Research (PGIMER) Chandigarh, Tata Memorial Center. Cancer Incidence and Mortality in S.A.S. Nagar District 2013. Chandigarh, India: PGIMER Chandigarh.

Poovathinal SA, Anitha A, Thomas R, Kaniamattam M, Melempatt N, Anilkumar A, Meena M. Prevalence of autism spectrum disorders in a semiurban community in south India. Ann Epidemiol. 2016; 26: 663–665e8.

Prasad DS, Kabir Z, Dash AK, Das BC. Prevalence and risk factors for diabetes and impaired glucose tolerance in Asian Indians: a community survey from urban eastern India. Diabetes Metab Syndr. 2012; 6: 96-101.

Prasad KN, Prasad A, Gupta RK, Nath K, Pradhan S, Tripathi M, Pandey CM. Neurocysticercosis in patients with active epilepsy from the pig farming community of Lucknow district, north India. Trans R Soc Trop Med Hyg. 2009; 103: 144-50.

Prasad KN, Rao RS, Sujatha A. Birthweight pattern in Karnataka. Indian Pediatr. 1994; 31: 836-9.

Premarajan KC, Danabalan M, Chandrasekar R, Srinivasa DK. Prevalence of psychiatry morbidity in an urban community of Pondicherry. Indian J Psychiatry. 1993; 35: 99-102.

Prince M, Acosta D, Ferri CP, Guerra M, Huang Y, Llibre Rodriguez JJ, Salas A, Sosa AL, Williams JD, Dewey ME, Acosta I, Jotheeswaran AT, Liu Z. Dementia incidence and mortality in middle-income countries, and associations with indicators of cognitive reserve: a 10/66 Dementia Research Group population-based cohort study. Lancet. 2012; 380: 50-8.

Profenno LA, Porsteinsson AP, Faraone SV. Meta-analysis of alzheimer's disease risk with obesity, diabetes, and related disorders. Biological Psychiatry 2010; 67: 505–12.

Public Health Foundation of India. Cause of Death Estimation Study in Bihar 2011-2014.

Public Health Foundation of India. India Cause of Death Estimation Study in Bihar 2011-2014. New Delhi, India: Public Health Foundation of India. [Data shared for this analysis]

Radhakrishnan K, Pandian JD, Santhoshkumar T, Thomas SV, Deetha TD, Sarma PS, Jayachandran D, Mohamed E. Prevalence, knowledge, attitude, and practice of epilepsy in Kerala, South India. Epilepsia. 2000; 41: 1027-35.

Radhakrishnan S, Balamurugan S. Prevalence of diabetes and hypertension among geriatric population in a rural community of Tamilnadu. Indian J Med Sci. 2013; 67: 130-8.

Raghunath R, Tripathi RM, Suseela B, Bhalke S, Shukla VK, Puranik VD. Dietary intake of metals by Mumbai adult population. Sci Total Environ. 2006; 356: 62-8.

Rahmathullah L, Tielsch JM, Thulasiraj RD, Katz J, Coles C, Devi S, John R, Prakash K, Sadanand AV, Edwin N, Kamaraj C. Impact of supplementing newborn infants with vitamin A on early infant mortality: community based randomised trial in southern India. BMJ. 2003; 327: 254.

Raina SK, Kashyap V, Bhardwaj AK, Kumar D, Chander V. Prevalence of autism spectrum disorders among children (1-10 years of age) - findings of a mid-term report from Northwest India. J Postgrad Med. 2015; 61: 243–6.

Raina SK, Razdan S, Nanda R. Prevalence of neurological disorders in children less than 10 years of age in RS Pura town of Jammu and Kashmir. J Pediatr Neurosci. 2011; 6: 103–5.

Rajkumar S, Kumar S, Thara R. Prevalence of dementia in a rural setting: A report from India. Int J Geriatr Psychiatry. 1997; 12: 702-7.

Rajshekhar V, Raghava MV, Prabhakaran V, Oommen A, Muliyil J. Active epilepsy as an index of burden of neurocysticercosis in Vellore district, India. Neurology. 2006; 67: 2135-9.

Ramachandran A, Snehalatha C, Krishnaswamy CV. Incidence of IDDM in children in urban population in southern India. Madras IDDM Registry Group Madras, South India. Diabetes Res Clin Pract. 1996; 34: 79-82.

Ramachandran A, Snehalatha C, Latha E, Manoharan M, Vijay V. Impacts of urbanisation on the lifestyle and on the prevalence of diabetes in native Asian Indian population. Diabetes Res Clin Pract. 1999; 44: 207-13.

Ramachandran A, Mary S, Yamuna A, Murugesan N, Snehalatha C. High prevalence of diabetes and cardiovascular risk factors associated with urbanization in India. Diabetes Care. 2008; 31: 893-8.

Ramachandran A, Snehalatha C, Abdul Khader OM, Joseph TA, Viswanathan M. Prevalence of childhood diabetes in an urban population in south India. Diabetes Res Clin Pract. 1992; 17: 227-31.

Ramachandran A, Snehalatha C, Kapur A, Vijay V, Mohan V, Das AK, Rao PV, Yajnik CS, Prasanna Kumar KM, Nair JD. High prevalence of diabetes and impaired glucose tolerance in India: National Urban Diabetes Survey. Diabetologia. 2001; 44: 1094-101.

Ramachandran P, Fitzwater SP, Aneja S, Verghese VP, Kumar V, Nedunchelian K, Wadhwa N, Veeraraghavan B, Kumar R, Meeran M. Prospective multi-centre sentinel surveillance for Haemophilus influenzae type b and other bacterial meningitis in Indian children. Indian J Med Res. 2013; 137(4): 712-20.

Raman Kutty V, Joseph A, Soman CR. High prevalence of type 2 diabetes in an urban settlement in Kerala, India. Ethn Health. 1999; 4: 231-9.

Rao CR, Kamath VG, Shetty A, Kamath A. A study on the prevalence of type 2 diabetes in coastal Karnataka. Int J Diabetes Dev Ctries. 2010; 30: 80-5.

Rao GN, Kulkarni GB, Gururaj G, Rajesh K, Subbakrishna DK, Steiner TJ, Stovner LJ. The burden of headache disorders in India: methodology and questionnaire validation for a community-based survey in Karnataka State. J Headache Pain. 2012; 13: 543-50.

Rao GN, Kulkarni GB, Gururaj G, Stovner LJ, Steiner TJ. The burden attributable to headache disorders in India: estimates from a community-based study in Karnataka State. J Headache Pain. 2015;16:94.

Rathod SD, Nadkarni A, Bhana A, Shidhaye R. Epidemiological features of alcohol use in rural India: a population-based cross-sectional study. BMJ Open. 2015; 5: e009802.

Ravikumar P, Bhansali A, Ravikiran M, Bhansali S, Walia R, Shanmugasundar G, Thakur JS, Kumar Bhadada S, Dutta P. Prevalence and risk factors of diabetes in a community-based study in North India: The Chandigarh Urban Diabetes Study (CUDS). Diabetes Metab. 2011; 37: 216-21.

Razdan S, Kaul RL, Motta A, Kaul S, Bhatt RK. Prevalence and pattern of major neurological disorders in rural Kashmir (India) in 1986. Neuroepidemiology. 1994; 13(3): 113-9.

Razdan S, Koul RL, Motta A, Kaul S. Cerebrovascular disease in rural Kashmir, India. Stroke. 1989; 20: 1691–3.

Reddaiah VP, Kapoor SK. Socio-biological factors in underfive deaths in a rural area. Indian J Pediatr. 1992; 59: 567-71.
Reddy BSK, Kumar KR, Balakrishnaiah G, Gopal KR, Reddy RR, Reddy LSS, Ahammed YN, Narasimhulu K, Moorthy KK, Babu SS. Potential Source Regions Contributing to Seasonal Variations of Black Carbon Aerosols over Anantapur in Southeast India. Aerosol Air Qual Res. 2012; 12: 344-58.
Reddy KK, Rao AP, Reddy TP. Socioeconomic status and the prevalence of coronary heart disease risk factors. Asia Pac J Clin Nutr. 2002; 11: 98-103.
Respiratory Epidemiology, Occupational Medicine & Public Health, National Heart & Lung Institute, Imperial College London. India - Pune Burden of Lung Disease Initiative Survey 2008-2009.
Respiratory Epidemiology, Occupational Medicine & Public Health, National Heart & Lung Institute, Imperial College London. India - Srinagar Burden of Lung Disease Intiative Survey 2011.
Ritchie GE, Kengne AP, Joshi R, Chow C, Neal B, Patel A, Zoungas S. Comparison of near-patient capillary glucose measurement and a risk assessment questionnaire in screening for type 2 diabetes in a high-risk population in rural India. Diabetes Care. 2011; 34: 44–9.
Sachdeva JS, Singh S, Sidhu BS, Goyal RK, Singh J. An epidemiological study of psychiatric disorders in rural Faridkot (Punjab). Indian J Psychiatry. 1986; 28: 317-23.
Sachdev HPS, Osmond C, Fall CHD, Lakshmy R, Ramji S, Dey Biswas SK, Prabhakaran D, Tandon N, Reddy KS, Barker DJP, Bhargava SK. Predicting adult metabolic syndrome from childhood body mass index: follow-up of the New Delhi birth cohort. Arch Dis Child. 2009; 94: 768-74.
Sadikot SM, Nigam A, Das S, Bajaj S, Zargar AH, Prasannakumar KM, Sosale A, Munichoodappa C, Seshiah V, Singh SK, Jamal A, Sai K, Sadasivrao Y, Murthy SS, Hazra DK, Jain S, Mukherjee S, Bandyopadhay S, Sinha NK, Mishra R, Dora M, Jena B, Patra P, Goenka K. The burden of diabetes and impaired glucose tolerance in India using the WHO 1999 criteria: prevalence of diabetes in India study (PODIS). Diabetes Res Clin Pract. 2004; 66: 301-7.
Saha S P, Bhattachrya S, Roy B K, Basu A, Roy T, Maity B, Das S K. A prospective incidence study of epilepsy in a rural community of West-Bengal, India. Neurology Asia 2008; 13: 41-8.
Saha SP, Bhattacharya S, Das SK, Maity B, Roy T, Raut DK. Epidemiological study of neurological disorders in a rural population of Eastern India. J Indian Med Assoc. 2003; 101: 299-304.
Saha SP, Bhattachrya S, Roy BK, Basu A, Maity B, Das SK. A prospective incidence study of epilepsy in a rural community of West-Bengal, India. Neurol Asia. 2008;8.
Sahu M, Peipert J, Singhal V, Yadama GN, Biswas P. Evaluation of mass and surface area concentration of particle emissions and development of emissions indices for cookstoves in rural India. Environ Sci Technol. 2011; 45: 2428-34.
Saleem S, McClure EM, Goudar SS, Patel A, Esamai F, Garces A, Chomba E, Althabe F, Moore J, Kodkany B, Pasha O, Belizan J, Mayansyan A, Derman RJ, Hibberd PL, Liechty EA, Krebs NF, Hambidge KM, Buekens P, Carlo WA, Wright LL, Koso-Thomas M, Jobe AH, Goldenberg RL, on behalf of the Global Network Maternal Newborn Health Registry Study Investigators. A prospective study of maternal, fetal and neonatal deaths in low- and middle-income countries. Bull World Health Organ. 2014; 92: 605-12.
Samokhvalov AV, Irving H, Mohapatra S, Rehm J. Alcohol consumption, unprovoked seizures, and epilepsy: a systematic review and meta-analysis. Epilepsia. 2010;51: 1177–84.
Samuel P, Antonisamy B, Raghupathy P, Richard J, Fall CHD. Socio-economic status and cardiovascular risk factors in rural and urban areas of Vellore, Tamilnadu, South India. Int J Epidemiol. 2012; 41: 1315-27.
Sangath, Columbia University, Alcohol Research Group, Public Health Institute, University of California San Francisco, London School of Hygiene and Tropical Medicine. Soryacher Asar Ani Hacher Amcho Shodh (SAAHAS) Goa Alcohol Use Study 2004-2008. Goa, India: Sangath.
Sankar J, Singhi P, Bansal A, Ray P, Singhi S. Role of dexamethasone and oral glycerol in reducing hearing and neurological sequelae in children with bacterial meningitis.Indian Pediatr. 2007; 44: 649-56.
Sapkota A, Gajalakshmi V, Jetly DH, Roychowdhury S, Dikshit RP, Brennan P, Hashibe M, Boffetta P. Indoor air pollution from solid fuels and risk of hypopharyngeal/laryngeal and lung cancers: a multicentric case-control study from India. Int J Epidemiol. 2008; 37: 321-8.
Satsangi PG, Yadav S, Pipal AS, Kumbhar N. Characteristics of trace metals in fine (PM2.5) and inhalable (PM10) particles and its health risk assessment along with in-silico approach in indoor environment of India. Atmos Environ. 2014; 384-93.
Sauvaget C, Ramadas K, Thomas G, Thara S, Sankaranarayanan R. Prognosis criteria of casual systolic and diastolic blood pressure values in a prospective study in India. J Epidemiol Community Health. 2010; 64: 366–72.
Savitha MR, Nandeeshwara SB, Pradeep Kumar MJ, ul-Haque F, Raju CK. Modifiable risk factors for acute lower respiratory tract infections. Indian J Pediatr. 2007; 74: 477-82.
Saxena N, Nayar D, Kapil U. Prevalence of underweight, stunting and wasting. Indian Pediatr. 1997; 34: 627-31.
Sekhar KC, Mohan CR, Bolla CSR, Deotale PG, Al Gabbany SAEF. A Study on Verbal Autopsy of Cause of Deaths Among Insured Members in Rural Areas of West Godavari District, India. World J Pharm Res. 2018; 7: 931-9.
Sen B, Nandi DN, Mukherjee SP, Mishra DC, Banerjee G, Sarkar S. Psychiatric morbidity in an urban slum-dwelling community. Indian J Psychiatry. 1984; 26: 185–93.
Shaddick G, Thomas ML. Particulate Matter 2.5 and 10 Surface Monitor Station Expanded Database 2008-2017. [Unpublished].
Shah N, Ramankutty V, Premila PG, Sathy N. Risk factors for severe pneumonia in children in south Kerala: a hospital-based case-control study. J Trop Pediatr. 1994; 40: 201-6.
Shah A, Afzal M. Prevalence of diabetes and hypertension and association with various risk factors among different Muslim populations of Manipur, India. J Diabetes Metab Disord. 2013; 12: 52.
Shah C, Sheth NR, Solanki B, Shah N. To assess the prevalence of impaired glucose tolerance and impaired fasting glucose in Western Indian population. J Assoc Physicians India. 2013; 61: 179–84.
Shah MS, Khalique N, Khan Z. Determinants of childhood mortality. Indian J Prev Soc Med. 2011; 42: 118-22.
Shah PA, Shapoo SF, Koul RK, Khan MA. Prevalence of epilepsy in school-going children (6-18 years) in Kashmir Valley of North-west India. J Indian Med Assoc. 2009; 107: 216-8.
Shaji S, Bose S, Verghese A. Prevalence of dementia in an urban population in Kerala, India. Br J Psychiatry. 2005; 186: 136-40.

Shaji S, Promodu K, Abraham T, Roy KJ, Verghese A. An epidemiological study of dementia in a rural community in Kerala, India. Br J Psychiatry. 1996; 168: 745-9.
Shaji S, Verghese A, Promodu K, George B, Shibu VP. Prevalence of priority psychiatric disorders in a rural area in kerala. Indian J Psychiatry. 1995; 37: 91–6.
Sharma P, Sharma P, Jain S, Prashant K. An integrated statistical approach for evaluating the exceedance of criteria pollutants in the ambient air of megacity Delhi. Atmos Environ. 2013; 70: 7-17.
Shell Foundation. India Shell Foundation Household Air Pollution Estimates 2009.
Shikha B, Harsh S, Narayan G. Infant deaths' audit: Contextual factors contributing to Infant deaths in tribal district-Valsad, Gujarat (India). Res Med Den Sci. 2015; 3: 171-5.
Shivpuri D, Rajesh MS, Jain D. Prevalence and characteristics of migraine among adolescents: a questionnaire survey. Indian Pediatr. 2003; 40: 665-9.
Shrivastava SR, Ghorpade AG. High prevalence of type 2 diabetes melitus and its risk factors among the rural population of Pondicherry, South India. J Res Health Sci. 2014; 14: 258-63.
Singh A, Kaur A. Epilepsy in rural Haryana – prevalence and treatment seeking behaviour. J Indian Med Assoc. 1997; 95: 37-47.
Singh G, Bawa J, Chinna D, Chaudhary A, Saggar K, Modi M, Sander JW. Association between epilepsy and cysticercosis and toxocariasis: a population-based case-control study in a slum in India. Epilepsia. 2012; 53: 2203-8.
Singh RB, Bajaj S, Niaz MA, Rastogi SS, Moshiri M. Prevalence of type 2 diabetes mellitus and risk of hypertension and coronary artery disease in rural and urban population with low rates of obesity. Int J Cardiol. 1998; 66: 65-72.
Singh RB, Fedacko J, Pella D, Macejova Z, Ghosh S, de Amit K, Begom R, Tumbis ZA, Haque M, Vajpeyee SK, de Meester F, Sergey C, Agarwalo R, Muthusamy VV, Five City Study Group, Gupta AK. Prevalence and risk factors for prehypertension and hypertension in five Indian cities. Acta Cardiol. 2011; 66: 29-37.
Singh AK, Mani K, Krishnan A, Aggarwal P, Gupta SK. Prevalence, awareness, treatment and control of diabetes among elderly persons in an urban slum of delhi. Indian J Community Med. 2012; 37: 236-9.
Singh AK, Farag YMK, Mittal BV, Subramanian KK, Reddy SRK, Acharya VN, Almeida AF, Channakeshavamurthy A, Ballal HS, P G, Issacs R, Jasuja S, Kirpalani AL, Kher V, Modi GK, Nainan G, Prakash J, Rana DS, Sreedhara R, Sinha DK, V SB, Sunder S, Sharma RK, Seetharam S, Raju TR, Rajapurkar MM. Epidemiology and risk factors of chronic kidney disease in India - results from the SEEK (Screening and Early Evaluation of Kidney Disease) study. BMC Nephrol. 2013; 114.
Singhal PK, Mathur GP, Mathur S, Singh YD. Mortality patterns in under six children in I.C.D.S. urban slum. Indian Pediatr. 1986; 23: 617-22.
Singhal PK, Mathur GP, Mathur S, Singh YD. Neonatal morbidity and mortality in ICDS Urban Slums. Indian Pediatr. 1990; 27: 485-8.
Singhal PK, Singh M, Paul VK, Deorari AK, Ghorpade MG, Malhotra A. Neonatal hypoglycemia--clinical profile and glucose requirements. Indian Pediatr. 1992; 29: 167-71.
Sohi D, Walia I, Singh A. Prevalence and treatment of epilepsy in a Chandigarh slum. Bull PGI. 1993; 27: 175-8.
Sreenivas V, Prabhakar AK, Badrinath SS, Fernandez T, Roy IS, Sharma T, Shah B. A rural population based case-control study of senile cataract in India. J Epidemiol. 1999; 9: 327-36.
Sridharan SE, Unnikrishnan JP, Sukumaran S, Sylaja PN, Nayak SD, Sarma PS, Radhakrishnan K. Incidence, Types, Risk Factors, and Outcome of Stroke in a Developing Country. Stroke. 2009; 40: 1212-8.
Srinath S, Girimaji SC, Gururaj G, Seshadri S, Subbakrishna DK, Bhola P, Kumar N. Epidemiological study of child and adolescent psychiatric disorders in urban and rural areas of Bangalore, India. Indian J Med Res. 2005; 122: 67-79.
St. John's Medical College Hospital (India). India - Bangalore St. John's Medical College Hospital Inpatient Data 2017.
St. John's National Academy of Health Sciences. Indian Stroke ProspectIve Registry (INSPIRE) Data 2009-2014.
State Bureau of Health Intelligence & Vital Statistics, Directorate of Health Services, Department of Health & Family Welfare, Government of Odisha. India - Odisha Medical Certification of Cause of Death Data 2009. Odisha, India: Department of Health & Family Welfare, Government of Odisha.
State Bureau of Health Intelligence & Vital Statistics, Directorate of Health Services, Department of Health & Family Welfare, Government of Odisha. India - Odisha Medical Certification of Cause of Death Data 2010. Odisha, India: Department of Health & Family Welfare, Government of Odisha
State Bureau of Health Intelligence & Vital Statistics, Directorate of Health Services, Department of Health & Family Welfare, Government of Odisha. India - Odisha Medical Certification of Cause of Death Data 2011. Odisha, India: Department of Health & Family Welfare, Government of Odisha.
State Bureau of Health Intelligence & Vital Statistics, Directorate of Health Services, Department of Health & Family Welfare, Government of Odisha. India - Odisha Medical Certification of Cause of Death Data 2012. Odisha, India: Department of Health & Family Welfare, Government of Odisha.
State Bureau of Health Intelligence & Vital Statistics, Directorate of Health Services, Department of Health & Family Welfare, Government of Odisha. India - Odisha Medical Certification of Cause of Death Data 2013. Odisha, India: Department of Health & Family Welfare, Government of Odisha.
Subramanian SV, Smith GD. Patterns, distribution, and determinants of under- and overnutrition: a population-based study of women in India. Am J Clin Nutr. 2006; 84: 633-40.
Sureka RK, Sureka R. Prevalence of epilepsy in rural Rajasthan--a door-to-door survey. J Assoc Physicians India. 2007; 55: 741-2.
Tamil Nadu Kidney Research Foundation, Kidney Disease Data Centre (KDDC), Chennai International Society of Nephrology (ISN). Chronic Kidney Disease and Cardiovascular Risk Survey 2008-2009. Chennai, India; ISN-KDDC.
Tamil Nadu Kidney Research Foundation, Kidney Disease Data Centre (KDDC), Chennai International Society of Nephrology (ISN). Chronic Kidney Disease and Cardiovascular Risk Survey 2014-2015. Chennai, India; ISN-KDDC.
The INTERSALT Co-operative Research Group. Appendix tables. Centre-specific results by age and sex. J Hum Hypertens 1989;3(5):331-407.
Thomas K, Mukkai Kesavan L, Veeraraghavan B, Jasmine S, Jude J, Shubankar M, Kulkarni P, Steinhoff M. Invasive pneumococcal disease associated with high case fatality in India. J Clin Epidemiol. 2013; 66: 36-43.

Tielsch JM, Katz J, Thulasiraj RD, Coles CL, Sheeladevi S, Yanik EL, Rahmathullah L. Exposure to indoor biomass fuel and tobacco smoke and risk of adverse reproductive outcomes, mortality, respiratory morbidity and growth among newborn infants in south India. Int J Epidemiol. 2009; 38: 1351-63.
Tobacco Sales Data. Personal Correspondence with Christopher Tan, 1997-2012. [Data shared for this analysis]
Trivedi SS, Pasrija S. Teenage pregnancies and their obstetric outcomes. Trop Doct. 2007; 37: 85-8.
Tyrovolas S, Koyanagi A, Garin N, Olaya B, Ayuso-Mateos JL, Miret M, Chatterji S, Tobiasz-Adamczyk B, Koskinen S, Leonardi M, Haro JM. Determinants of the components of arterial pressure among older adults--the role of anthropometric and clinical factors: a multi-continent study. Atherosclerosis. 2015; 238: 240–9.
U.S. Department of Agriculture (USDA). USDA Global Tobacco Database 1960-2005. Washington DC , United States: USDA.
Ughade SN, Zodpey SP, Khanolkar VA. Risk factors for cataract: a case control study. Indian J Ophthalmol. 1998; 46: 221-7.
United Nations Office on Drugs and Crime (UNODC). World Drug Report 2012. Vienna, Austria: UNODC; 2012.
Unnikrishnan JP, Sylaja S, Nayak SD, Radhakrishnan K. India - Trivandrum Stroke Registry 2005. [Unpublished].
Upadhyaya S, Shettyb S, Kumarc SS, Dongred A, Deshmukhe P. Institutionalizing district level infant death review: an experience from southern India. Southeast Asia J Public Health. 2012; 1: 446-56.
Vadivoo S, Gupte MD, Adhikary R, Kohli A, Kangusamy B, Joshua V, Mathai AK, Kumar K, Mainkar M, Goswami P, IBBA Study Team. Appropriateness and execution challenges of three formal size estimation methods for high-risk populations in India. AIDS. 2008; 22: S137-148.
Vas CJ, Pinto C, Panikker D, Noronha S, Deshpande N, Kulkarni L, Sachdeva S. Prevalence of Dementia in an Urban Indian Population. Int Psychogeriatr. 2001; 13: 439-50.
Vijayakumar G, Arun R, Kutty VR. High prevalence of type 2 diabetes mellitus and other metabolic disorders in rural Central Kerala. J Assoc Physicians India. 2009; 563-7.
Vijayaraghavan K, Rao DH. Diet & nutrition situation in rural India. Indian J Med Res. 1998; 108:243-53.
Vogel JP, Souza JP, Mori R, Morisaki N, Lumbiganon P, Laopaiboon M, Ortiz-Panozo E, Hernandez B, Pérez-Cuevas R, Roy M, Mittal S, Cecatti JG, Tunçalp Ö, Gülmezoglu AM; WHO Multicountry Survey on Maternal and Newborn Health Research Network. Maternal complications and perinatal mortality: findings of the World Health Organization Multicountry Survey on Maternal and Newborn Health. BJOG. 2014; 121: 76-88.
Wadia NH, Bhatia K. Multiple sclerosis is prevalent in the Zoroastrians (Parsis) of India. Ann Neurol. 1990; 28: 177-9.
Wang H-Y, Leena KB, Plymoth A, Hergens M-P, Yin L, Shenoy KT, Ye W. Prevalence of gastro-esophageal reflux disease and its risk factors in a community-based population in southern India. BMC Gastroenterol. 2016; 16: 36.
Wayse V, Yousafzai A, Mogale K, Filteau S. Association of subclinical vitamin D deficiency with severe acute lower respiratory infection in Indian children under 5 y. Eur J Clin Nutr. 2004; 58: 563-7.
World Bank. Survey of Living Conditions 1997-1998, Uttar Pradesh and Bihar. Washington D.C., United States: World Bank.
World Bank. World Development Indicators - Gross Domestic Product (GDP). Washington DC, United States of America: World Bank.
World Health Organization (WHO). Global Database on Child Growth and Malnutrition - Historical. Geneva, Switzerland: WHO.
World Health Organization (WHO). WHO Global Health Observatory - Recorded Alcohol Per Capita Consumption 1960-1979. Geneva, Switzerland: World Health Organization (WHO).
World Health Organization (WHO). WHO Global Health Observatory - Recorded Alcohol Per Capita Consumption 1980-1999. Geneva, Switzerland: World Health Organization (WHO).
World Health Organization (WHO). WHO Global Health Observatory - Recorded Alcohol Per Capita Consumption 2000-2009 by country. Geneva, Switzerland: World Health Organization (WHO).
World Health Organization (WHO). WHO Global Infobase - Tobacco Use Prevalence. Geneva, Switzerland: WHO.
World Health Organization (WHO). WHO Global Survey on Maternal and Perinatal Health 2004-2008. Geneva, Switzerland: WHO.
World Health Organization (WHO). WHO Household Energy Database 1974-2008. Geneva, Switzerland: World Health Organization (WHO), 2010.
World Health Organization (WHO), United Nations Children's Fund (UNICEF). WHO and UNICEF Reported Administrative Data for Immunization Coverage Time Series.
World Health Organization. Study on Global Ageing and Adult Health (SAGE) Pilot Study 2005 Data from the Data Archive of Social Research on Aging. Los Altos, United States: Sociometrics Corporation.
Wylie BJ, Coull BA, Hamer DH, Singh MP, Jack D, Yeboah-Antwi K, Sabin L, Singh N, MacLeod WB. Impact of biomass fuels on pregnancy outcomes in central East India. Environ Health. 2014; 13: 1.
Zaman FA, Borang A. Prevalence of diabetes mellitus amongst rural hilly population of North Eastern India and its relationship with associated risk factors and related co-morbidities. J Nat Sci Biol Med. 2014; 5: 383-8.
Zargar AH, Khan AK, Masoodi SR, Laway BA, Wani AI, Bashir MI, Dar FA. Prevalence of type 2 diabetes mellitus and impaired glucose tolerance in the Kashmir Valley of the Indian subcontinent. Diabetes Res Clin Pract. 2000; 47: 135-46.
Zhang J, Chen C, Hua S, Liao H, Wang M, Xiong Y, Cao F. An updated meta-analysis of cohort studies: diabetes and risk of Alzheimer's disease. Diabetes Res Clin Pract. 2017; 124: 41-47.
Zhong G, Wang Y, Zhang Y, Guo JJ, Zhao Y. Smoking is associated with an increased risk of dementia: a meta-analysis of prospective cohort studies with investigation of potential effect modifiers. PLoS One 2015;10: e0118333
Zodpey SP, Ughade SN. Exposure to cheaper cooking fuels and risk of age-related cataract in women. Indian J Occup Environ Med. 1999; 3: 159-61.

3. Socio-demographic Index of the states of India, 2019

States of India	SDI in 2019
Bihar	0.45
Madhya Pradesh	0.50
Jharkhand	0.50
Rajasthan	0.51
Uttar Pradesh	0.51
Chhattisgarh	0.52
Odisha	0.54
Andhra Pradesh	0.54
Assam	0.54
West Bengal	0.55
Tripura	0.55
Arunachal Pradesh	0.55
Meghalaya	0.55
Telangana	0.56
Karnataka	0.57
Manipur	0.59
Jammu & Kashmir and Ladakh	0.60
Haryana	0.60
Gujarat	0.60
Mizoram	0.61
Tamil Nadu	0.61
Nagaland	0.62
Sikkim	0.62
Uttarakhand	0.62
Punjab	0.62
Maharashtra	0.62
Himachal Pradesh	0.63
Kerala	0.66
Other small union territories	0.67
Delhi	0.71
Goa	0.72

SDI as computed by GBD in 2019 as described elsewhere (Lancet 2020; 396: 1135-39).
SDI= Socio-demographic Index.

4. Correlation between SDI of the states of India and the DALY rates of non-communicable, communicable, and injury-related neurological disorders, 2019

Neurological disorders	Correlation between SDI of states of India and					
	Crude DALY rate			Age-standardised DALY rate		
	r*	r-square	p-value	r*	r-square	p-value
Non-communicable neurological disorders	-0.04	0.002	0.826	-0.32	0.10	0.078
Communicable neurological disorders	-0.56	0.31	0.001	-0.52	0.28	0.002
Injury-related neurological disorders	0.61	0.37	<0.0001	0.42	0.18	0.019

SDI= Socio-demographic Index.
r = Pearson correlation coefficient.
*Following categories were used for the strength of correlation: 0.00-0.19 as very weak, 0.20-0.39 as weak, 0.40-0.59 as moderate, 0.60-0.79 as strong, and 0.80-1 as very strong (Swinscow TDV, Campbell MJ, editors. Statistics at Square One. London: BMJ Publishing Group. 1997).

5. Crude DALY rates of non-communicable, communicable, and injury-related neurological disorders in the states of India, 2019

States of India*	DALYs per 100,000 (95% uncertainty interval)		
	Non-communicable neurological disorders	Communicable neurological disorders	Injury-related neurological disorders
**India**	2,754 (2,143-3,615)	369 (305-493)	198 (146-260)
Bihar	2,265 (1,686-3,094)	364 (271-534)	156 (113-204)
Madhya Pradesh	2,656 (2,043-3,511)	446 (346-615)	181 (133-237)
Jharkhand	2,234 (1,628-3,058)	304 (223-451)	179 (131-236)
Rajasthan	2,264 (1,625-3,132)	374 (282-545)	182 (132-240)
Uttar Pradesh	2,220 (1,629-3,100)	615 (496-775)	168 (122-219)
Chhattisgarh	3,925 (3,154-4,855)	281 (207-421)	186 (137-244)
Odisha	3,661 (2,914-4,652)	374 (284-485)	198 (146-259)
Andhra Pradesh	2,968 (2,255-3,974)	330 (270-402)	218 (162-285)
Assam	3,562 (2,884-4,507)	276 (193-504)	153 (112-199)
West Bengal	3,792 (2,983-4,762)	249 (191-396)	204 (149-268)
Tripura	3,765 (2,902-4,763)	153 (102-324)	198 (145-262)
Arunachal Pradesh	2,132 (1,544-3,012)	290 (220-434)	156 (115-206)
Meghalaya	2,242 (1,613-3,090)	309 (223-458)	141 (103-184)
Telangana	2,659 (1,974-3,561)	271 (211-379)	243 (178-319)
Karnataka	3,182 (2,453-4,098)	316 (253-438)	208 (152-274)
Manipur	2,963 (2,240-3,882)	204 (145-353)	207 (151-275)
Jammu & Kashmir and Ladakh	2,355 (1,751-3,207)	185 (133-339)	263 (182-375)
Haryana	2,358 (1,708-3,250)	313 (243-454)	221 (163-291)
Gujarat	2,560 (1,898-3,448)	395 (323-507)	207 (152-273)
Mizoram	1,878 (1,247-2,854)	343 (262-457)	171 (124-225)
Tamil Nadu	2,729 (2,069-3,666)	350 (292-413)	283 (208-372)
Nagaland	2,710 (2,024-3,628)	299 (219-462)	161 (117-209)
Sikkim	2,212 (1,517-3,166)	185 (139-263)	210 (153-278)
Uttarakhand	2,678 (1,984-3,639)	408 (329-521)	225 (165-296)
Punjab	2,765 (2,094-3,658)	229 (175-363)	249 (183-327)
Maharashtra	3,010 (2,331-3,942)	249 (190-393)	202 (149-265)
Himachal Pradesh	2,659 (1,970-3,641)	189 (145-285)	238 (176-312)
Kerala	3,381 (2,665-4,362)	184 (137-315)	271 (199-356)
Other small union territories	2,490 (1,797-3,420)	170 (123-307)	245 (179-323)
Delhi	2,167 (1,538-3,018)	226 (168-365)	203 (149-269)
Goa	3,220 (2,405-4,231)	135 (90-270)	265 (194-352)

*The states are listed in increasing order of Socio-demographic Index in 2019.

6. Number of deaths due to neurological disorders in India, 2019

Neurological disorders	Number of deaths (95% uncertainty interval)			Percentage of total neurological disorders deaths (95% uncertainty interval)		
	Both sexes	Males	Females	Both sexes	Males	Females
**Non-communicable disorders**						
Stroke	699,100 (593,800-806,600)	363,200 (294,000-443,400)	335,800 (264,600-412,800)	68.0 (54.6-75.3)	69.5 (58.1-76.0)	66.4 (52.1-5.1)
Epilepsy	32,700 (26,800-39,200)	17,100 (14,000-21,200)	15,600 (11,100-19,900)	3.2 (2.5-3.8)	3.3 (2.6-3.9)	3.1 (2.1-4.0)
Alzheimer's disease and other dementias	129,000 (31,200-360,000)	55,800 (12,600-163,400)	73,200 (17,800-199,700)	12.0 (3.2-29.1)	10.3 (2.7-25.7)	13.8 (3.7-32.4)
Brain and CNS cancer	23,700 (18,600-28,900)	13,300 (8,900-17,300)	10,400 (8,000-13,000)	2.3 (1.7-2.8)	2.5 (1.7-3.3)	2.1 (1.5-2.5)
Parkinson's disease	45,300 (38,600-52,800)	25,600 (20,500-30,900)	19,800 (15,600-24,600)	4.4 (3.5-5.1)	4.9 (3.8-5.7)	3.9 (3.0-4.7)
Multiple sclerosis	2,310 (1,860-2,930)	1,020 (750-1,340)	1,290 (950-1,890)	0.2 (0.2-0.3)	0.2 (0.1-0.3)	0.3 (0.2-0.4)
Motor neuron diseases	1,600 (1,220-1990)	900 (640-1,200)	700 (530-950)	0.2 (0.1-0.2)	0.2 (0.1-0.2)	0.1 (0.1-0.2)
Other neurological disorders	5,840 (4,210-8,280)	2,580 (1,580-4,390)	3,260 (2,440-4,220)	0.6 (0.4-0.8)	0.5 (0.3-0.8)	0.6 (0.5-0.8)
**Communicable disorders**						
Encephalitis	51,900 (40,400-85,000)	24,300 (18,200-46,100)	27,600 (20,500-45,300)	5.0 (3.7-8.1)	4.6 (3.4-8.3)	5.5 (3.8-8.8)
Meningitis	34,700 (29,700-40,000)	16,700 (13,800-20,700)	18,000 (14,900-21,300)	3.4 (2.7-4.0)	3.2 (2.5-4.0)	3.6 (2.7-4.4)
Tetanus	7,330 (4,920-11,020)	3,920 (2,160-6,360)	3,410 (2,240-5,220)	0.7 (0.4-1.1)	0.8 (0.4-1.3)	0.7 (0.4-1.1)

7. Prevalence or incidence rate of neurological disorders in the states of India, 2019

States of India*	Prevalence per 100,000 (95% uncertainty interval)							
	Headache disorders	Epilepsy	Cerebral palsy	Alzheimer's disease and other dementias	Brain and CNS cancer	Parkinson's disease	Multiple sclerosis	Motor neuron diseases
**India**	**35,060 (32,254-37,903)**	**726 (604-853)**	**1,210 (1,054-1,396)**	**266 (225-306)**	**3.5 (2.7-4.4)**	**55 (46-66)**	**7.7 (6.0-9.4)**	**1.8 (1.4-2.3)**
Bihar	33,287 (30,477-36,091)	617 (445-817)	1,239 (1,051-1,455)	203 (172-234)	3.2 (2.4-4.3)	42 (35-50)	6.9 (5.4-8.5)	1.7 (1.3-2.1)
Madhya Pradesh	34,444 (31,583-37,322)	660 (490-845)	1,092 (934-1,291)	227 (194-264)	3.4 (2.6-4.4)	47 (39-56)	7.3 (5.7-9.0)	1.7 (1.3-2.1)
Jharkhand	34,595 (31,791-37,501)	767 (567-978)	1,270 (1,106-1,486)	244 (207-283)	2.6 (1.9-3.6)	50 (41-59)	7.3 (5.7-9.0)	1.7 (1.4-2.2)
Rajasthan	34,748 (31,951-37,665)	643 (475-826)	1,144 (979-1,340)	231 (196-267)	3.5 (2.7-4.5)	46 (38-55)	7.9 (6.2-9.8)	1.9 (1.5-2.4)
Uttar Pradesh	33,970 (31,160-36,831)	669 (491-866)	1,127 (968-1,315)	195 (166-225)	3.3 (2.6-4.5)	43 (35-51)	7.7 (6.0-9.4)	1.7 (1.3-2.1)
Chhattisgarh	34,765 (31,954-37,633)	810 (587-1,038)	1,111 (949-1,300)	227 (193-264)	2.9 (2.2-3.8)	51 (42-60)	7.2 (5.5-8.9)	1.7 (1.3-2.1)
Odisha	35,441 (32,638-38,238)	814 (568-1,046)	1,083 (926-1,271)	292 (249-338)	3.4 (2.6-4.6)	62 (51-74)	7.6 (5.9-9.4)	1.6 (1.3-2.1)
Andhra Pradesh	36,400 (33,591-39,291)	822 (604-1,051)	1,126 (983-1,300)	368 (312-428)	3.3 (2.4-4.4)	72 (59-86)	6.7 (5.3-8.3)	1.7 (1.4-2.2)
Assam	34,652 (31,782-37,518)	680 (489-885)	1,147 (980-1,331)	192 (163-222)	2.9 (2.1-4.5)	42 (34-50)	8.4 (6.6-10.3)	1.7 (1.4-2.2)
West Bengal	36,392 (33,495-39,300)	467 (343-613)	1,234 (1,070-1,424)	251 (214-291)	3.4 (2.4-4.4)	57 (47-69)	8.7 (6.8-10.6)	1.9 (1.5-2.4)
Tripura	36,481 (33,553-39,397)	696 (511-898)	1,298 (1,120-1,499)	265 (225-307)	2.3 (1.7-3.6)	52 (42-62)	8.5 (6.6-10.3)	1.9 (1.5-2.4)
Arunachal Pradesh	34,096 (31,241-37,037)	729 (537-952)	1,459 (1,254-1,708)	149 (127-171)	2.9 (2.1-4.5)	31 (26-37)	7.8 (6.1-9.8)	2.0 (1.5-2.5)
Meghalaya	34,093 (31,227-37,054)	751 (552-964)	1,340 (1,147-1,578)	164 (140-189)	2.5 (1.8-4.0)	32 (26-38)	7.3 (5.6-9.2)	1.9 (1.5-2.4)
Telangana	36,262 (33,407-39,127)	874 (667-1,090)	1,187 (1,027-1,385)	272 (233-317)	3.5 (2.5-4.7)	58 (47-70)	6.9 (5.4-8.7)	1.8 (1.4-2.3)
Karnataka	33,820 (30,967-36,640)	905 (657-1,189)	1,222 (1,062-1,419)	318 (270-368)	3.9 (2.8-4.8)	64 (52-77)	6.2 (4.8-7.7)	1.7 (1.4-2.2)
Manipur	35,364 (32,478-38,231)	647 (473-819)	1,358 (1,176-1,578)	217 (184-250)	2.7 (1.9-3.9)	43 (35-51)	8.4 (6.6-10.3)	1.9 (1.5-2.4)
Jammu & Kashmir and Ladakh	35,587 (32,705-38,525)	597 (439-774)	1,343 (1,161-1,565)	247 (211-286)	3.6 (2.7-4.7)	46 (37-57)	10.6 (8.4-13.1)	2.2 (1.8-2.8)
Haryana	35,458 (32,644-38,295)	825 (611-1,028)	1,237 (1,068-1,445)	286 (244-328)	3.4 (2.6-4.3)	56 (47-67)	9.6 (7.5-11.8)	2.1 (1.7-2.7)
Gujarat	35,308 (32,472-38,127)	855 (640-1,074)	1,180 (1,025-1,369)	252 (214-292)	3.4 (2.7-4.6)	56 (46-68)	8.0 (6.3-9.8)	1.9 (1.5-2.4)
Mizoram	34,559 (31,696-37,405)	714 (534-904)	1,325 (1,144-1,543)	213 (183-245)	3.3 (2.3-4.4)	39 (32-46)	7.7 (6.0-9.5)	1.9 (1.5-2.4)
Tamil Nadu	36,607 (33,809-39,467)	769 (579-968)	1,214 (1,052-1,402)	366 (312-421)	3.9 (2.9-5.0)	73 (60-88)	6.3 (4.9-7.8)	1.7 (1.3-2.1)
Nagaland	34,401 (31,546-37,344)	756 (554-951)	1,475 (1,269-1,736)	198 (168-229)	3.1 (2.2-4.5)	41 (34-48)	7.7 (6.1-9.5)	2.0 (1.5-2.5)
Sikkim	37,137 (34,133-40,142)	800 (591-1,027)	1,306 (1,134-1,516)	247 (210-285)	2.7 (1.9-4.8)	51 (42-61)	9.5 (7.5-11.7)	2.3 (1.8-2.9)
Uttarakhand	35,659 (32,828-38,531)	911 (682-1,171)	1,363 (1,182-1,583)	256 (217-296)	3.6 (2.8-5.0)	57 (46-68)	10.5 (8.3-12.9)	2.0 (1.6-2.5)
Punjab	36,226 (33,408-39,107)	865 (650-1,104)	1,278 (1,115-1,491)	356 (304-409)	4.0 (3.1-5.3)	74 (61-88)	11.4 (9.0-14)	2.2 (1.7-2.7)
Maharashtra	36,130 (33,266-38,927)	784 (585-995)	1,380 (1,195-1,609)	323 (274-373)	4.2 (2.9-5.2)	69 (57-82)	7.5 (5.8-9.3)	1.9 (1.5-2.4)
Himachal Pradesh	36,373 (33,538-39,200)	828 (599-1,054)	1,508 (1,276-1,764)	359 (305-415)	4.0 (2.8-5.1)	68 (56-81)	11.9 (9.5-14.5)	2.2 (1.8-2.8)
Kerala	36,279 (33,537-39,014)	865 (641-1,087)	1,500 (1,309-1,731)	564 (482-646)	5.3 (3.0-6.7)	96 (79-115)	6.6 (5.1-8.2)	1.9 (1.5-2.4)
Other small union territories	37,315 (34,342-40,276)	820 (600-1,032)	1,367 (1,176-1,584)	255 (217-294)	3.5 (2.3-4.6)	56 (47-67)	6.9 (5.3-8.6)	2.0 (1.6-2.5)
Delhi	35,851 (32,938-38,713)	818 (567-1,078)	1,275 (1,101-1,483)	201 (168-235)	4.8 (2.6-6.5)	51 (43-61)	10.1 (7.9-12.5)	2.2 (1.8-2.8)
Goa	36,911 (34,120-39,758)	958 (696-1,210)	1,336 (1,166-1,550)	414 (352-475)	4.4 (2.6-5.8)	86 (71-102)	7.8 (6.1-9.5)	2.1 (1.7-2.6)

States of India*	Incidence per 100,000 (95% uncertainty interval)					
	Stroke	Encephalitis	Meningitis	Tetanus	Traumatic brain injuries	Spinal cord injuries
**India**	**93 (83-104)**	**44 (40-48)**	**40 (33-47)**	**1.19 (0.78-1.88)**	**537 (459-623)**	**9.7 (7.5-12.5)**
Bihar	71 (63-80)	44 (39-49)	43 (34-55)	1.39 (0.87-2.27)	450 (386-520)	8.0 (6.2-10.1)
Madhya Pradesh	87 (78-98)	42 (37-46)	48 (39-57)	0.62 (0.33-1.37)	513 (439-593)	9.0 (6.9-11.6)
Jharkhand	78 (69-87)	44 (39-48)	38 (30-45)	1.17 (0.73-1.89)	479 (410-554)	8.5 (6.6-11.0)
Rajasthan	74 (66-83)	40 (36-45)	42 (34-50)	0.99 (0.58-1.70)	522 (439-616)	8.9 (6.7-11.8)
Uttar Pradesh	64 (57-72)	43 (39-48)	51 (42-61)	3.93 (2.04-6.81)	503 (429-592)	8.8 (6.7-11.4)
Chhattisgarh	124 (112-140)	42 (37-46)	38 (30-46)	0.98 (0.59-1.68)	526 (451-607)	9.6 (7.4-12.2)
Odisha	123 (110-140)	50 (45-54)	35 (29-42)	0.72 (0.43-1.19)	516 (443-597)	9.5 (7.3-12.1)
Andhra Pradesh	109 (97-122)	52 (47-56)	34 (28-40)	0.27 (0.15-0.61)	566 (483-658)	10.7 (8.2-13.8)
Assam	110 (98-124)	39 (35-44)	36 (29-44)	0.83 (0.49-1.62)	411 (348-479)	7.2 (5.5-9.3)
West Bengal	145 (129-163)	41 (37-45)	34 (27-40)	0.53 (0.33-0.85)	507 (433-588)	9.3 (7.2-12.0)
Tripura	134 (120-150)	38 (34-42)	33 (27-40)	0.23 (0.11-0.73)	477 (407-552)	8.5 (6.6-11.1)
Arunachal Pradesh	65 (58-73)	40 (36-45)	40 (32-49)	0.15 (0.04-0.83)	461 (394-538)	8.1 (6.2-10.3)
Meghalaya	69 (62-78)	39 (35-44)	44 (36-54)	0.34 (0.17-0.84)	415 (354-484)	7.1 (5.5-9.1)
Telangana	88 (78-98)	42 (38-46)	34 (28-41)	0.22 (0.12-0.53)	643 (547-748)	11.4 (8.6-14.9)
Karnataka	109 (97-123)	51 (46-55)	36 (30-42)	0.14 (0.05-0.68)	557 (475-650)	10.5 (8.1-13.4)
Manipur	104 (92-116)	41 (36-45)	35 (28-42)	0.67 (0.38-1.20)	506 (431-591)	8.5 (6.4-11.5)
Jammu & Kashmir and Ladakh	77 (68-86)	35 (31-39)	35 (29-42)	0.16 (0.08-0.50)	592 (504-693)	11.1 (8.2-14.9)
Haryana	79 (71-89)	41 (37-45)	38 (31-44)	0.57 (0.32-0.96)	601 (513-698)	10.5 (8.0-13.7)
Gujarat	83 (74-95)	45 (40-49)	42 (34-50)	0.43 (0.27-0.84)	559 (478-647)	10.1 (7.7-13.0)
Mizoram	54 (48-61)	45 (40-50)	45 (37-54)	0.17 (0.06-0.67)	478 (408-554)	8.2 (6.3-10.7)
Tamil Nadu	100 (89-113)	53 (48-58)	26 (21-32)	0.08 (0.02-0.44)	740 (633-858)	14.0 (10.7-18.1)
Nagaland	89 (80-99)	40 (35-44)	42 (34-51)	1.42 (0.63-2.64)	454 (388-528)	7.8 (6.0-10.1)
Sikkim	79 (71-89)	35 (31-38)	32 (26-38)	0.07 (0.03-0.36)	554 (473-645)	9.8 (7.5-12.6)
Uttarakhand	84 (75-95)	41 (37-45)	44 (36-52)	2.57 (0.86-4.46)	636 (542-734)	11.2 (8.5-14.6)
Punjab	101 (90-113)	43 (38-47)	33 (27-39)	0.38 (0.24-0.68)	627 (535-729)	11.0 (8.2-14.5)
Maharashtra	103 (92-117)	41 (37-46)	36 (29-43)	0.39 (0.24-0.66)	524 (445-614)	9.8 (7.5-12.6)
Himachal Pradesh	95 (85-107)	39 (35-43)	28 (22-33)	0.49 (0.21-1.13)	597 (512-692)	10.8 (8.3-13.9)
Kerala	152 (135-172)	49 (45-54)	34 (28-40)	0.23 (0.11-0.43)	636 (545-738)	12.6 (9.6-16.2)
Other small union territories	84 (75-95)	38 (34-42)	31 (25-37)	0.08 (0.04-0.35)	627 (535-729)	11.5 (8.8-14.8)
Delhi	71 (63-79)	37 (33-42)	38 (31-45)	0.40 (0.17-0.71)	528 (450-613)	9.1 (6.9-12.0)
Goa	121 (108-137)	43 (39-48)	31 (25-38)	0.37 (0.09-0.93)	636 (544-740)	12.3 (9.4-15.9)

*The states are listed in increasing order of Socio-demographic Index in 2019

8. Correlation between SDI of the states of India and the prevalence or incidence and DALY rates of neurological disorders, 2019

Neurological disorders	Correlation between SDI of states of India and											
	Crude prevalence			Age-standardised prevalence			Crude DALY rate			Age-standardised DALY rate		
**Non-communicable disorders**	**r***	**r-square**	**p-value**	**r***	**r-square**	**p-value**	**r***	**r-square**	**p-value**	**r***	**r-square**	**p-value**
Headache disorders	0.63	0.39	<0.0001	0.023	0.0005	0.902	0.68	0.46	<0.0001	0.060	0.004	0.747
Epilepsy	0.55	0.30	0.002	0.54	0.29	0.002	0.12	0.02	0.553	0.42	0.17	0.024
Cerebral palsy	0.56	0.31	0.001	0.66	0.43	<0.0001	0.56	0.32	0.001	0.70	0.49	<0.0001
Alzheimer's disease and other dementias	0.44	0.19	0.013	0.09	0.01	0.615	0.42	0.18	0.019	-0.16	0.02	0.398
Brain and CNS cancer	0.61	0.37	<0.0001	0.64	0.41	<0.0001	0.23	0.05	0.207	0.12	0.01	0.521
Parkinson's disease	0.49	0.24	0.005	0.41	0.16	0.024	0.46	0.21	0.009	-0.04	0.002	0.825
Multiple sclerosis	0.32	0.10	0.081	-0.02	0.0005	0.908	0.24	0.06	0.201	-0.21	0.04	0.268
Motor neuron diseases	0.67	0.45	<0.0001	0.62	0.38	<0.0001	0.75	0.57	<0.0001	0.49	0.24	0.005
	**Crude incidence rate**			**Age-standardised incidence rate**			**Crude DALY rate**			**Age-standardised DALY rate**		
**Non-communicable disorders**	**r***	**r-square**	**p-value**	**r***	**r-square**	**p-value**	**r***	**r-square**	**p-value**	**r***	**r-square**	**p-value**
Stroke	0.11	0.01	0.558	-0.27	0.07	0.141	-0.17	0.03	0.370	-0.36	0.13	0.048
**Communicable disorders**												
Encephalitis	-0.12	0.01	0.529	-0.27	0.07	0.144	-0.55	0.30	0.002	-0.52	0.27	0.003
Meningitis	-0.45	0.20	0.011	-0.21	0.04	0.260	-0.42	0.18	0.018	-0.38	0.14	0.036
Tetanus	-0.33	0.11	0.067	-0.31	0.10	0.086	-0.36	0.13	0.046	-0.33	0.11	0.067
**Injuries**												
Traumatic brain injuries	0.54	0.29	0.002	0.40	0.16	0.027	0.62	0.39	<0.0001	0.49	0.24	0.005
Spinal cord injuries	0.53	0.28	0.002	0.39	0.15	0.032	0.48	0.23	0.006	0.24	0.06	0.201

SDI= Socio-demographic Index.
r = Pearson correlation coefficient.
*Following categories were used for the strength of correlation: 0.00-0.19 as very weak, 0.20-0.39 as weak, 0.40-0.59 as moderate, 0.60-0.79 as strong, and 0.80-1 as very strong (Swinscow TDV, Campbell MJ, editors. Statistics at Square One. London: BMJ Publishing Group. 1997).

9. Age-specific prevalence of select neurological disorders by sex in India, 2019

Age group (years)	Prevalence per 100,000 (95% uncertainty interval)							
	Migraine		Tension-type headache		Epilepsy		Cerebral palsy	
	Males	Females	Males	Females	Males	Females	Males	Females
**Under 5**	0	0	0	0	654 (452-864)	597 (409-806)	1,834 (1,570-2,153)	1,740 (1,500-2,029)
**5 to 9**	1,830 (1,141-2,771)	3,273 (2,056-4,895)	7,769 (4,935-11,042)	8,508 (5,462-12,228)	775 (549-1044)	647 (453-882)	1,484 (1,281-1,730)	1,374 (1,194-1,594)
**10 to 14**	9,006 (6,302-12,649)	15,438 (10,937-21,502)	26,510 (19,013-35,636)	28,382 (20,431-38,329)	820 (577-1075)	680 (471-907)	1,442 (1,245-1,677)	1,336 (1,165-1,543)
**15 to 19**	13,650 (10,140-17,623)	21,996 (16,753-28,580)	31,221 (21,489-43,280)	32,530 (22,404-45,349)	821 (582-1060)	685 (477-900)	1,384 (1,200-1,605)	1,284 (1,121-1,478)
**20 to 24**	15,411 (12,041-19,422)	23,846 (18,809-29,428)	32,731 (22,473-44,335)	33,279 (22,871-45,511)	803 (577-1028)	678 (474-882)	1,332 (1,156-1,546)	1,245 (1,089-1,431)
**25 to 29**	16,539 (13,091-20,617)	25,261 (20,285-30,913)	34,464 (24,487-46,534)	34,037 (24,222-45,775)	775 (565-985)	655 (460-856)	1,309 (1,139-1,516)	1,236 (1,081-1,421)
**30 to 34**	17,301 (13,624-21,710)	26,325 (21,054-33,016)	34,819 (23,393-46,724)	33,703 (22,991-45,919)	725 (507-940)	613 (424-812)	1,269 (1,104-1,464)	1,216 (1,066-1,395)
**35 to 39**	17,521 (14,189-21,744)	27,071 (22,150-33,155)	367,16 (26,535-49,480)	35,745 (25,479-47,580)	680 (484-875)	577 (396-770)	1,216 (1,057-1,402)	1,191 (1,044-1,362)
**40 to 44**	17,850 (14,613-22,437)	28,225 (23,260-34,454)	33,690 (23,336-45,377)	33,060 (23,301-44,285)	648 (449-845)	557 (368-754)	1,145 (997-1,316)	1,154 (1,013-1,316)
**45 to 49**	16,646 (13,286-20,604)	26,808 (21,522-33,007)	30,395 (20,491-43,652)	29,453 (20,491-42,170)	640 (453-816)	573 (394-761)	1,058 (921-1,212)	1,105 (971-1,260)
**50 to 54**	14,976 (11,857-18,607)	24,129 (19,180-29,910)	31,828 (22,032-43,465)	30,168 (21,022-41,707)	656 (477-847)	623 (436-814)	953 (827-1,094)	1,038 (913-1,178)
**55 to 59**	13,220 (10,401-16,569)	21,136 (16,686-26,641)	25,978 (17,670-35,409)	24,190 (16,692-32,971)	718 (534-928)	709 (518-927)	830 (716-950)	929 (812-1,057)
**60 to 64**	11,463 (9,006-14,363)	18,119 (14,185-22,767)	28,245 (20,214-37,880)	26,084 (18,545-34,991)	819 (609-1,086)	829 (613-1,084)	695 (592-804)	811 (704-922)
**65 to 69**	9,701 (7,291-12,172)	15,110 (11,432-18,934)	30,535 (20,260-42,995)	29,305 (19,400-41,179)	961 (687-1,286)	972 (722-1,270)	576 (482-679)	666 (572-762)
**70 to 74**	7,936 (6,091-10,251)	12,166 (9,350-15,653)	32,835 (23,558-44,694)	33,827 (24,655-45,761)	1,132 (807-1,551)	1,134 (828-1,521)	497 (410-595)	555 (466-648)
**75 to 79**	6,568 (5,049-8,553)	9,951 (7,550-12,906)	32,676 (23,256-44,768)	34,430 (24,547-45,957)	1,382 (972-1,842)	1,300 (923-1,725)	463 (368-567)	485 (397-580)
**80 plus**	5,219 (4,043-6,695)	7,811 (6,023-10,032)	26,936 (19,712-35,087)	28,633 (21,211-37,635)	1,677 (1,203-2,200)	1,463 (1,073-1,917)	161 (120-205)	170 (132-212)

Age group (years)	Prevalence per 100,000 (95% uncertainty interval)											
	Alzheimer's disease and other dementias		Brain and CNS cancer		Parkinson's disease		Multiple sclerosis		Motor neuron diseases		Other neurological disorders	
	Males	Females	Males	Females	Males	Females	Males	Females	Males	Females	Males	Females
**Under 5**	0	0	9.2 (4.8-14.3)	6.8 (4.7-9.7)	0	0	0	0	0.7 (0.5-1.1)	0.6 (0.3-0.9)	0.6 (0.3-1.0)	0.4 (0.2-0.6)
**5 to 9**	0	0	2.8 (1.7-4.0)	2.9 (2.0-4.2)	0	0	0.05 (0.01-0.11)	0.07 (0.02-0.15)	1.3 (0.9-1.8)	1.0 (0.7-1.5)	0.7 (0.3-1.3)	0.4 (0.2-0.7)
**10 to 14**	0	0	1.9 (1.2-2.6)	2.1 (1.5-2.8)	0	0	0.4 (0.2-0.7)	0.6 (0.3-1.0)	1.7 (1.1-2.4)	1.4 (0.9-2.0)	0.7 (0.3-1.3)	0.4 (0.1-0.7)
**15 to 19**	0	0	2.0 (1.3-2.9)	2.6 (2.0-3.5)	0	0	1.2 (0.7-2.0)	2.1 (1.3-3.2)	2.1 (1.4-3.0)	1.6 (1.1-2.4)	0.8 (0.4-1.3)	0.3 (0.2-0.6)
**20 to 24**	0	0	2.0 (1.3-2.7)	2.5 (1.8-3.5)	0.3 (0.1-0.6)	0.2 (0.1-0.4)	2.7 (1.8-4.1)	4.8 (3.3-7.0)	2.4 (1.7-3.4)	1.8 (1.3-2.7)	0.8 (0.4-1.4)	0.4 (0.2-0.7)
**25 to 29**	0	0	2.4 (1.6-3.3)	2.7 (1.8-3.7)	1.9 (0.6-3.8)	1.3 (0.4-2.8)	4.9 (3.3-7.7)	8.7 (5.9-13.1)	2.6 (1.8-3.7)	2.0 (1.4-2.9)	0.7 (0.4-1.2)	0.4 (0.2-0.8)
**30 to 34**	0	0	2.6 (1.8-3.5)	2.3 (1.6-3.0)	5.3 (2.5-9.8)	3.9 (1.7-7.3)	7.4 (5.1-10.4)	12.8 (9.0-17.7)	2.7 (1.6-3.9)	2.2 (1.3-3.3)	0.7 (0.3-1.2)	0.5 (0.2-1.0)
**35 to 39**	0	0	3.0 (2.0-4.1)	2.2 (1.5-2.9)	11.8 (7.3-17.7)	8.5 (5.0-13.4)	9.8 (6.9-13.0)	16.7 (12.0-21.5)	2.5 (1.5-3.9)	2.2 (1.3-3.4)	0.6 (0.3-1.1)	0.6 (0.3-1.0)
**40 to 44**	5.7 (2.8-9.3)	6.9 (3.5-11.1)	2.9 (1.9-3.9)	1.9 (1.4-2.6)	22.5 (14.3-32.5)	16.3 (9.8-24.7)	11.9 (8.8-15.3)	19.7 (14.9-24.9)	2.2 (1.2-3.7)	2.1 (1.2-3.5)	0.7 (0.3-1.3)	0.6 (0.3-1.2)
**45 to 49**	42.5 (27.7-60.5)	52.6 (34.7-74.6)	4.0 (2.6-5.6)	2.7 (1.9-3.6)	41.0 (29.2-54.4)	30.4 (20.9-41.6)	13.3 (9.9-16.6)	21.7 (16.7-26.5)	2.0 (1.1-3.1)	2.0 (1.2-3.2)	0.8 (0.3-1.4)	0.6 (0.3-1.1)
**50 to 54**	125.4 (90.4-169.2)	161.2 (117.1-215.8)	5.1 (3.1-7.2)	4.0 (2.7-5.4)	69.9 (51.1-92.5)	53.2 (37.6-70.7)	13.8 (10.4-17.3)	22.5 (17.6-27.5)	1.7 (0.8-2.9)	1.8 (0.9-3.0)	0.9 (0.4-1.7)	0.6 (0.3-1.2)
**55 to 59**	276.5 (209.3-353.3)	370.2 (280.5-470.2)	7.0 (4.5-9.6)	4.9 (3.5-6.5)	117.3 (89.4-154.7)	92.1 (69.0-121.4)	13.2 (10.1-16.7)	21.9 (17.3-27.0)	1.6 (0.8-2.5)	1.6 (0.8-2.7)	1.1 (0.5-2.0)	0.7 (0.3-1.3)
**60 to 64**	566.6 (436.3-729.0)	801.3 (621.6-1023.6)	7.3 (4.6-9.9)	5.8 (4.2-7.4)	197.5 (147.8-266.7)	157.8 (118.2-212.7)	11.7 (9.0-14.9)	20.2 (15.9-24.8)	1.6 (0.9-2.5)	1.6 (0.9-2.5)	1.2 (0.5-2.4)	0.9 (0.4-1.7)
**65 to 69**	1,065.4 (818.7-1,361.8)	1,527.2 (1,187.7-1,911.6)	7.6 (4.6-10.3)	6.2 (4.4-7.8)	356.0 (262.8-471.9)	282.6 (211.0-371.8)	10.0 (7.5-12.7)	18.2 (14.3-22.4)	1.6 (0.9-2.6)	1.6 (0.9-2.6)	1.3 (0.6-2.3)	1.2 (0.6-2.3)
**70 to 74**	2,001.5 (1,560.7-2,557.4)	2,583.5 (2,041.9-3,246.5)	6.8 (4.2-9.2)	6.1 (4.4-7.7)	600.6 (437.7-804.6)	466.4 (345.3-620.5)	8.3 (6.3-10.7)	16.0 (12.5-20.0)	1.6 (0.9-2.5)	1.5 (0.9-2.5)	1.2 (0.6-2.4)	1.5 (0.7-2.9)
**75 to 79**	3,853.1 (3,005.5-4,902.6)	4,293.8 (3,400.0-5,408.1)	5.3 (3.3-7.2)	5.2 (3.7-6.6)	946.8 (701.7-1,193.6)	697.4 (523.5-882.6)	6.7 (5.1-8.9)	13.8 (10.8-17.6)	1.5 (0.8-2.5)	1.5 (0.8-2.5)	1.1 (0.6-1.9)	1.7 (0.9-3.0)
**80 plus**	9,215.6 (7,250.3-11,413.2)	9,312.1 (7,435.5-11,415.3)	4.1 (2.5-5.4)	3.5 (2.3-4.5)	1,215.2 (862.3-1,609.0)	867.3 (619.8-1,141.1)	4.9 (3.6-6.7)	10.9 (8.5-14.2)	1.8 (1.1-2.8)	1.8 (1.1-2.8)	1.1 (0.5-1.9)	1.8 (0.9-3.2)

10. Age-specific incidence rate of select neurological disorders by sex in India, 2019

Age group (years)	Incidence per 100,000 (95% uncertainty interval)							
	Stroke		Encephalitis		Meningitis		Tetanus	
	Males	Females	Males	Females	Males	Females	Males	Females
**Under 5**	21 (13-32)	25 (16-37)	104 (83-130)	114 (90-141)	183 (131-251)	209 (152-288)	9.5 (4.5-17.7)	9.0 (4.5-16.6)
**5 to 9**	17 (10-27)	22 (13-33)	42 (29-56)	48 (35-64)	50 (29-83)	53 (31-87)	0.2 (0.1-0.6)	0.1 (0.1-0.4)
**10 to 14**	14 (8-23)	17 (11-28)	25 (18-33)	28 (21-38)	25 (14-41)	28 (15-45)	0.1 (0.1-0.4)	0.2 (0.1-0.4)
**15 to 19**	12 (7-17)	14 (9-21)	18 (14-24)	21 (16-27)	19 (11-33)	23 (14-39)	0.2 (0.1-0.5)	0.2 (0.1-0.5)
**20 to 24**	13 (9-19)	15 (10-22)	16 (11-22)	17 (12-23)	17 (9-33)	24 (12-45)	0.2 (0.1-0.5)	0.3 (0.1-0.7)
**25 to 29**	18 (12-27)	20 (13-30)	17 (12-23)	16 (11-22)	18 (11-28)	24 (15-37)	0.3 (0.1-0.6)	0.2 (0.1-0.5)
**30 to 34**	30 (23-40)	31 (23-41)	20 (15-25)	17 (13-23)	20 (12-30)	22 (14-34)	0.3 (0.1-0.7)	0.3 (0.1-0.6)
**35 to 39**	50 (38-66)	49 (36-65)	23 (17-29)	21 (15-28)	17 (10-28)	19 (11-31)	0.6 (0.2-1.1)	0.3 (0.1-0.6)
**40 to 44**	84 (68-103)	82 (67-101)	28 (21-35)	27 (20-35)	17 (10-26)	18 (11-27)	0.6 (0.2-1.3)	0.3 (0.2-0.7)
**45 to 49**	133 (100-171)	131 (99-168)	34 (26-42)	36 (27-45)	18 (11-28)	21 (12-32)	0.7 (0.3-1.5)	0.4 (0.2-0.9)
**50 to 54**	202 (164-247)	195 (158-236)	43 (35-53)	47 (38-58)	20 (13-30)	23 (15-36)	0.8 (0.3-1.7)	0.7 (0.3-1.4)
**55 to 59**	291 (223-379)	271 (205-354)	56 (42-72)	62 (48-78)	21 (12-34)	24 (13-39)	0.9 (0.4-2.0)	0.5 (0.2-1.1)
**60 to 64**	358 (286-448)	342 (272-430)	76 (61-93)	82 (67-99)	23 (14-34)	26 (16-39)	0.9 (0.4-2.2)	0.6 (0.3-1.4)
**65 to 69**	404 (306-523)	408 (308-526)	104 (81-129)	106 (85-129)	25 (15-39)	29 (17-44)	1.0 (0.4-2.3)	0.8 (0.4-1.8)
**70 to 74**	456 (363-579)	475 (377-604)	136 (110-165)	130 (107-157)	31 (21-46)	36 (24-53)	1.6 (0.6-3.4)	2.0 (0.9-4.3)
**75 to 79**	518 (399-656)	549 (423-693)	172 (136-213)	154 (126-185)	43 (24-68)	47 (27-75)	1.7 (0.6-3.5)	1.9 (0.9-3.9)
**80 plus**	786 (634-945)	809 (655-969)	282 (239-329)	220 (187-255)	61 (43-83)	72 (51-97)	3.7 (1.3-6.9)	2.2 (1.1-4.5)

Age group (years)	Incidence per 100,000 (95% uncertainty interval)			
	Traumatic brain injuries		Spinal cord injuries	
	Males	Females	Males	Females
**Under 5**	159 (126-197)	121 (93-157)	2.4 (1.9-2.9)	2.2 (1.7-2.8)
**5 to 9**	177 (137-226)	154 (115-203)	2.6 (2.0-3.3)	2.6 (1.9-3.6)
**10 to 14**	297 (221-396)	212 (158-280)	4.1 (2.9-5.7)	3.7 (2.6-5.0)
**15 to 19**	549 (381-809)	277 (205-377)	7.4 (4.9-11.2)	5.2 (3.7-7.4)
**20 to 24**	795 (566-1,147)	316 (236-443)	10.9 (7.1-16.4)	6.3 (4.4-9.2)
**25 to 29**	857 (586-1,185)	327 (235-445)	12.1 (7.9-18.5)	6.3 (4.4-9.3)
**30 to 34**	855 (587-1,224)	364 (255-508)	12.6 (8.4-19.0)	6.7 (4.5-10.0)
**35 to 39**	874 (632-1,179)	426 (302-582)	13.3 (8.8-19.7)	7.7 (5.1-11.6)
**40 to 44**	867 (597-1,212)	455 (314-638)	13.6 (8.9-20.2)	8.3 (5.5-12.7)
**45 to 49**	836 (591-1,151)	469 (332-643)	13.7 (9.1- 20.4)	9.1 (6.1-13.7)
**50 to 54**	809 (579-1,114)	526 (378-720)	13.9 (9.4-20.6)	11.0 (7.4-16.0)
**55 to 59**	803 (573-1,066)	627 (444-829)	14.6 (9.7- 21.1)	14.2 (9.3-20.9)
**60 to 64**	832 (591-1,152)	759 (548-1,019)	15.9 (10.7-22.9)	18.8 (12.4-27.5)
**65 to 69**	928 (679-1,239)	954 (680-1,314)	18.7 (12.9-26.8)	25.9 (17.0-37.4)
**70 to 74**	1,083 (799-1,458)	1,199 (826-1,655)	23.1 (15.6- 33.1)	34.9 (22.0-53.2)
**75 to 79**	1,315 (941-1,759)	1,608 (1,073-2,348)	28.9 (18.9-43.1)	48.5 (28.3-78.0)
**80 plus**	1,492 (1,165-1,907)	2,107 (1,479-2,964)	33.9 (23.4-48.1)	64.2 (38.9-101.5)

11. DALYs of neurological disorders by age group in India, 2019

Age group (years)	DALYs (95% uncertainty interval)						
	Stroke	Headache disorders	Epilepsy	Cerebral palsy	Alzheimer's disease and other dementias	Brain and CNS cancer	Parkinson's disease
**Under 5**	61,095 (80,376-46,812)	0	483,918 (340,741-650,223)	323,514 (218,932-452,501)	0	152,525 (209,990-107,000)	0
**5 to 9**	39,102 (49,836-30,237)	113,317 (294,923-2,516)	416,419 (280,114-58,6507)	303,925 (207,090-428,745)	0	77,556 (102,260-57,321)	0
**10 to 14**	61,790 (76,994-48,058)	614,778 (1,485,452-31,872)	489,421 (341,379-675,101)	309,676 (21,0285-433,643)	0	63,584 (81,864-47,863)	0
**15 to 19**	112,688 (135,712-91,836)	934,732 (2,248,659-98,586)	551,875 (399,406-738,837)	293,557 (201,821-407,504)	0	40,357 (52,046-31,797)	0
**20 to 24**	189,315 (222,518-158,474)	994,570 (2,297,219-127,794)	532,823 (389,152-707,433)	268,557 (184,051-372,695)	0	43,461 (56,171-33,912)	202 (269-142)
**25 to 29**	219,046 (254,542-187,993)	967,864 (2,163,482-139,506)	448,710 (325,409-605,677)	240,731 (165,606-333,451)	0	50,172 (63,462-40,122)	517 (879-291)
**30 to 34**	336,316 (388,878-287,734)	943,454 (2,099,583-152,080)	419,450 (302,661-561,163)	216,394 (148,783-300,450)	0	58,547 (73,989-45,597)	1,191 (1,997-689)
**35 to 39**	519,634 (606,338-447,236)	878,245 (1,969,305-140,278)	347,344 (245,289-468,534)	186,656 (128,493-257,418)	0	65,989 (84,040-50,929)	2,376 (3,487-1,549)
**40 to 44**	714,736 (837,898-607,301)	778,200 (1,707,200-142,375)	261,070 (173,343-362,862)	149,245 (103,235-205,208)	5,053 (15,927-1,118)	65,499 (83,022-49,036)	8,160 (10,187-6,457)
**45 to 49**	1,016,498 (1,192,369-856,238)	648,750 (1,432,899-139,047)	222,940 (150,648-305,319)	117,772 (81,571-162,495)	25,851 (72,740-6,988)	66,075 (83,267-49,416)	15,234 (18,662-12,309)
**50 to 54**	1,575,146 (1,863,058-1,299,160)	503,180 (1,093,091-119,667)	204,773 (145,944-273,076)	86,174 (59,602-117,623)	47,328 (12,2701-15,498)	69,705 (90,327-52,056)	25,477 (30,936-20,910)
**55 to 59**	1,995,898 (2,354,992-1,638,409)	370,346 (797,921-96,172)	187,919 (135,488-250,413)	58,658 (40,811-80,248)	7,7104 (19,5236-28,474)	70,269 (88,784-52,407)	35,431 (43,042-29,131)
**60 to 64**	2,474,918 (2,856,635-2,102,663)	277,510 (578,568-78,647)	170,216 (123,899-227,936)	37,333 (26,041-50,870)	12,6321 (30,4524-50,489)	64,909 (81,081-49,372)	56,936 (67,630-47,043)
**65 to 69**	2,673,010 (3,073,563-2,305,540)	195,920 (399,705-59,987)	156,515 (115,869-213,329)	19,875 (14,045-26,631)	198,689 (484,173-79,756)	56,140 (69,546-42,377)	99,557 (117,685-82,736)
**70 to 74**	2,188,945 (2,519,351-1,889,095)	110,806 (233,051-34,409)	122,680 (89,460-165,000)	8,114 (5,713-10,735)	277,601 (665,378-118,405)	36,425 (44,380-27,312)	155,178 (180,885-132,041)
**75 to 79**	1,649,874 (1,912,145-1,410,557)	57,676 (119,891-17,234)	83,894 (57,669-114,278)	2,885 (2,058-3,809)	341,849 (845,907-143,769)	20,654 (25,355-15,394)	185,690 (215,563-158,541)
**80 plus**	1,504,316 (1,751,097-1,253,983)	39,736 (82,844-12,554)	94,385 (67,293-12,4499)	1,031 (742-1,362)	1,025,745 (2,524,234-390,192)	11,379 (13,836-8,283)	227,024 (267,162-194,400)

Age group (years)	DALYs (95% uncertainty interval)							
	Multiple sclerosis	Motor neuron diseases	Other non-communicable neurological disorders	Encephalitis	Meningitis	Tetanus	Traumatic brain injuries	Spinal cord injuries
**Under 5**	0	4,115 (5,845-2,830)	47,589 (29,266-69,305)	621,712 (1,087,131-418,201)	917,190 (1,214,847-664,817)	409,878 (674,295-228,501)	4,461 (2,867-6,327)	2,625 (1,810-3,476)
**5 to 9**	47 (87-21)	933 (1,212-706)	53,955 (32,593-80,928)	270,812 (497,609-171,897)	194,748 (266,006-143,717)	7,363 (16,789-3,826)	10,325 (6,746-14,670)	8,853 (6,215-11,728)
**10 to 14**	230 (383-127)	1,226 (1,556-944)	77,252 (45,940-122,394)	202,989 (352,313-140,310)	149,248 (186,774-116,402)	5,897 (11,945-3,416)	22,064 (14,926-30,934)	18,066 (12,406-24,389)
**15 to 19**	925 (1,374-613)	1,502 (1,896-1,177)	70,020 (44,048-105,520)	130,125 (229,432-94,244)	121,194 (147,768-100,121)	7,412 (15,199-4,585)	43,936 (29,678-61,311)	32,013 (22,404-41,721)
**20 to 24**	3,604 (5,307-2,535)	1,598 (1,998-1,265)	48,808 (31,547-71,337)	141,689 (251,714-104,266)	105,430 (128,280-87,566)	8,849 (17,225-5,223)	74,844 (51,091-105,463)	51,091 (36,039-65,132)
**25 to 29**	5,991 (8,201-4,408)	1,361 (1,730-1,065)	32,587 (21,794-46,614)	97,966 (1732,43-72,074)	92,385 (110,749-76,191)	8,066 (15,136-5,150)	105,822 (72,796-144,769)	68,469 (48,834-88,928)
**30 to 34**	9,605 (12,496-7,499)	1,774 (2,225-1,400)	27,599 (19,230-39,142)	100,845 (173,032-75,942)	85,263 (102,367-71,445)	8,787 (15,347-5,670)	138,836 (95,734-194,317)	85,765 (61,121-109,403)
**35 to 39**	13,399 (17,003-10,543)	2,665 (3,383-2,081)	29,396 (21,033-40,360)	95,241 (166,307-71,638)	76,736 (93,491-63,507)	9,528 (16,317-5,871)	162,627 (112,741-223,247)	93,939 (66,270-120,431)
**40 to 44**	15,389 (19,293-12,215)	3,634 (4,723-2,761)	28,428 (20,596-38,324)	86,784 (145,589-64,672)	73,186 (88,332-59,736)	8,899 (14,867-5,215)	178,048 (124,503-243,191)	94,738 (67,426-121,140)
**45 to 49**	15,305 (19,022-12,138)	5,650 (7,389-4,138)	30,393 (22,664-40,435)	76,165 (134,453-56,429)	74,561 (91,377-60,409)	8,732 (14,347-5,247)	187,426 (131,677-256,847)	91,832 (67,034-117,596)
**50 to 54**	14,057 (18,163-11,132)	6,655 (8,878-4,803)	28,049 (20,912-36,846)	72,577 (131,354-52,285)	74,119 (91,873-59,192)	8,737 (15,249-5,158)	184,949 (130,552-251,330)	81,993 (59,420-104,009)
**55 to 59**	12,158 (15,535-9,597)	8,080 (10,660-6,038)	26,149 (20,048-34,390)	78,365 (139,207-56,875)	64,248 (79,290-51,748)	6,473 (12,046-3,850)	176,492 (123,855-241,675)	68,572 (50,654-86,940)
**60 to 64**	9,006 (11,487-7,080)	7,298 (9,426-5,449)	22,265 (17,341-28,211)	90,028 (154,113-67,226)	46,318 (56,454-37,786)	5,322 (9,855-3,311)	167,859 (119,695-226,951)	59,057 (43,036-73,903)
**65 to 69**	6,153 (7,576-5,004)	6,477 (8,217-4,981)	18,539 (14,704-23,557)	98,033 (161,068-74,395)	39,455 (47,846-32,040)	4,296 (7,443-2,674)	150,412 (106,512-202,724)	50,912 (37,457-64,111)
**70 to 74**	3,701 (4,605-2,980)	2,939 (3,864-2,218)	12,827 (10,226-16,130)	95,446 (150,327-73,167)	31,455 (38,776-25,621)	4,972 (8,431-2,994)	113,407 (80,365-151,479)	36,683 (26,735-46,267)
**75 to 79**	2,005 (2,472-1,625)	1,416 (1,844-1,068)	8,483 (6,753-10,642)	78,260 (116,790-60,486)	23,017 (27,880-18,785)	2,511 (4,129-1,474)	79,229 (56,324-106,840)	24,247 (17,642-31,219)
**80 plus**	1,419 (1,720-1,148)	611 (794-466)	6,723 (5,323-8,304)	85,308 (113,135-67,638)	23,427 (27,921-18,926)	2,160 (3,710-1,203)	69,572 (49,042-94,965)	18,594 (12,801-25,266)

12. Percentage contribution of communicable, non-communicable, and injury-related disorders to total neurological DALYs in each age group in India, 2019

Age group (years)	Non-communicable neurological disorders	Communicable neurological disorders	Injury-related neurological disorders
Under 5	36.1%	63.7%	0.2%
5 to 9	67.1%	31.6%	1.3%
10 to 14	80.2%	17.8%	2.0%
15 to 19	85.7%	11.1%	3.2%
20 to 24	84.5%	10.4%	5.1%
25 to 29	84.1%	8.5%	7.4%
30 to 34	82.8%	8.0%	9.2%
35 to 39	82.4%	7.3%	10.3%
40 to 44	82.1%	6.8%	11.0%
45 to 49	83.1%	6.1%	10.7%
50 to 54	85.8%	5.2%	8.9%
55 to 59	87.8%	4.6%	7.6%
60 to 64	89.8%	3.9%	6.3%
65 to 69	90.9%	3.8%	5.3%
70 to 74	91.2%	4.1%	4.7%
75 to 79	91.9%	4.1%	4.0%
80 plus	93.6%	3.6%	2.8%
